# Supplementary material for: Islet Gene View—a tool to facilitate islet research
Source: Life Sci Alliance. 2022 Aug 10;5(12):e202201376. doi: 10.26508/lsa.202201376 (PMC9366203; doi:10.26508/lsa.202201376)
Supplement: Supplementary file 1 [file LSA-2022-01376_TableS1.docx]

Supplementary table 1: Clinical characteristics of the organ donors.

| **Variable** | **n** | **Mean (± SD)** | **Min** | **Max** |
| --- | --- | --- | --- | --- |
| Age | 187 | 59±10 | 24 | 81 |
| BMI | 188 | 26.4±3.8 | 17.6 | 40.1 |
| HbA1c | 169 | 5.9±0.7 | 4.3 | 10 |
| Female / Male | 70 / 118 |  |  |  |
| **Non-T2D** | 155 |  |  |  |
| Age | 154 | 58.3±10.3 | 24 | 76 |
| BMI | 155 | 26.3±3.5 | 18 | 40.1 |
| HbA1c | 137 | 5.7±0.5 | 4.3 | 8 |
| Female / Male | 59/96 |  |  |  |
| **T2D** | 33 |  |  |  |
| Age | 33 | 60.9±10.3 | 41 | 81 |
| BMI | 33 | 28.2±4.5 | 20.1 | 34.9 |
| HbA1c | 32 | 6.8±0.9 | 5.7 | 10 |
| Female / Male | 11 / 22 |  |  |  |
