# Supplementary material for: Islet Gene View—a tool to facilitate islet research
Source: Life Sci Alliance. 2022 Aug 10;5(12):e202201376. doi: 10.26508/lsa.202201376 (PMC9366203; doi:10.26508/lsa.202201376)
Supplement: Supplementary file 2 [file LSA-2022-01376_TableS2.docx]

Supplementary table 2: Genes with genome-wide significant (FDR<=0.05) differential expression between T2D and non-T2D donor islets.

| **HGNC symbol** | **genes** | **logFC** | **P.Value** | **FDR** |
| --- | --- | --- | --- | --- |
| OPRD1 | ENSG00000116329 | -1.33 | 1.31E-11 | 1.85E-07 |
| SERPINE2 | ENSG00000135919 | 0.60 | 5.05E-10 | 3.56E-06 |
| PLA1A | ENSG00000144837 | -0.97 | 2.00E-09 | 9.42E-06 |
| MAP2K6 | ENSG00000108984 | -0.54 | 8.71E-09 | 3.07E-05 |
| SFRP1 | ENSG00000104332 | 1.10 | 1.44E-08 | 3.97E-05 |
| GRAMD3 | ENSG00000155324 | -0.34 | 1.69E-08 | 3.97E-05 |
| RASGRP1 | ENSG00000172575 | -0.78 | 2.01E-08 | 4.06E-05 |
| KCNAB2 | ENSG00000069424 | 0.58 | 3.32E-08 | 5.68E-05 |
| SFRP4 | ENSG00000106483 | 1.95 | 3.62E-08 | 5.68E-05 |
| RASD2 | ENSG00000100302 | 1.16 | 4.28E-08 | 6.04E-05 |
| SMOC2 | ENSG00000112562 | 1.92 | 5.44E-08 | 6.98E-05 |
| ARG2 | ENSG00000081181 | -0.76 | 6.70E-08 | 7.88E-05 |
| CLMP | ENSG00000166250 | 0.89 | 1.37E-07 | 1.39E-04 |
| GLRA1 | ENSG00000145888 | -1.40 | 1.38E-07 | 1.39E-04 |
| CCL22 | ENSG00000102962 | 1.77 | 1.61E-07 | 1.51E-04 |
| CRH | ENSG00000147571 | 1.00 | 2.22E-07 | 1.96E-04 |
| SYNDIG1 | ENSG00000101463 | 0.79 | 2.73E-07 | 2.27E-04 |
| CD5 | ENSG00000110448 | 0.92 | 8.30E-07 | 6.05E-04 |
| APOD | ENSG00000189058 | 1.29 | 8.58E-07 | 6.05E-04 |
| KAZN | ENSG00000189337 | 0.48 | 9.81E-07 | 6.59E-04 |
| *SLC2A2* | ENSG00000163581 | -1.13 | 1.09E-06 | 6.98E-04 |
| *HHATL* | ENSG00000010282 | -1.04 | 1.18E-06 | 7.21E-04 |
| *APOLD1* | ENSG00000178878 | 0.74 | 1.26E-06 | 7.41E-04 |
| *PTGDS* | ENSG00000107317 | 1.43 | 1.35E-06 | 7.64E-04 |
| *VASH2* | ENSG00000143494 | 0.65 | 1.47E-06 | 7.93E-04 |
| *FSTL4* | ENSG00000053108 | 0.62 | 1.54E-06 | 7.93E-04 |
| *MIS18BP1* | ENSG00000129534 | -0.30 | 1.57E-06 | 7.93E-04 |
| *TAC1* | ENSG00000006128 | 1.09 | 1.74E-06 | 8.44E-04 |
| *BMX* | ENSG00000102010 | 1.21 | 2.76E-06 | 1.30E-03 |
| *FFAR4* | ENSG00000186188 | -0.85 | 3.20E-06 | 1.46E-03 |
| *CHRDL1* | ENSG00000101938 | 1.09 | 6.08E-06 | 2.68E-03 |
| *MFAP4* | ENSG00000166482 | 1.04 | 6.28E-06 | 2.68E-03 |
| *FST* | ENSG00000134363 | 0.71 | 7.22E-06 | 2.99E-03 |
| *ORC6* | ENSG00000091651 | -0.50 | 7.82E-06 | 3.01E-03 |
| *FGF7* | ENSG00000140285 | 0.96 | 7.98E-06 | 3.01E-03 |
| *SCAI* | ENSG00000173611 | -0.32 | 8.08E-06 | 3.01E-03 |
| *SSTR5-AS1* | ENSG00000261713 | 0.83 | 8.11E-06 | 3.01E-03 |
| *IL1RL1* | ENSG00000115602 | 1.33 | 9.53E-06 | 3.45E-03 |
| *TMEM158* | ENSG00000249992 | 0.75 | 1.01E-05 | 3.55E-03 |
| *RP11-395G23.3* | ENSG00000254615 | 0.51 | 1.13E-05 | 3.90E-03 |
| *PTGER4* | ENSG00000171522 | 0.45 | 1.17E-05 | 3.94E-03 |
| *PPP1R1A* | ENSG00000135447 | -0.73 | 1.30E-05 | 4.25E-03 |
| *PODN* | ENSG00000174348 | 0.87 | 1.33E-05 | 4.25E-03 |
| *BMP6* | ENSG00000153162 | 0.80 | 1.38E-05 | 4.25E-03 |
| *CHL1* | ENSG00000134121 | -1.02 | 1.38E-05 | 4.25E-03 |
| *MPP1* | ENSG00000130830 | -0.25 | 1.73E-05 | 5.08E-03 |
| *ARL4C* | ENSG00000188042 | 0.40 | 1.73E-05 | 5.08E-03 |
| *NR2F1-AS1* | ENSG00000237187 | -0.86 | 1.76E-05 | 5.08E-03 |
| *SPON1* | ENSG00000262655 | 0.74 | 1.96E-05 | 5.54E-03 |
| *PPFIBP2* | ENSG00000166387 | -0.48 | 2.07E-05 | 5.65E-03 |
| *MIA2* | ENSG00000150526 | -0.50 | 2.09E-05 | 5.65E-03 |
| *C1QTNF1* | ENSG00000173918 | 0.76 | 2.12E-05 | 5.65E-03 |
| *SH2D2A* | ENSG00000027869 | 0.75 | 2.48E-05 | 6.34E-03 |
| *GREM2* | ENSG00000180875 | -1.06 | 2.50E-05 | 6.34E-03 |
| *FGFBP1* | ENSG00000137440 | 1.26 | 2.52E-05 | 6.34E-03 |
| *CTSF* | ENSG00000174080 | 0.42 | 2.59E-05 | 6.41E-03 |
| *AASS* | ENSG00000008311 | -0.38 | 2.71E-05 | 6.58E-03 |
| *PBLD* | ENSG00000108187 | -0.55 | 2.89E-05 | 6.92E-03 |
| *HS6ST2* | ENSG00000171004 | -0.53 | 3.05E-05 | 7.17E-03 |
| *SLC22A15* | ENSG00000163393 | -0.37 | 3.16E-05 | 7.29E-03 |
| *HSPB6* | ENSG00000004776 | 0.89 | 3.20E-05 | 7.29E-03 |
| *MEDAG* | ENSG00000102802 | 0.98 | 3.50E-05 | 7.63E-03 |
| *CTSV* | ENSG00000136943 | -0.58 | 3.51E-05 | 7.63E-03 |
| *CLCF1* | ENSG00000175505 | 0.59 | 3.51E-05 | 7.63E-03 |
| *RAB39A* | ENSG00000179331 | -0.48 | 3.59E-05 | 7.67E-03 |
| *CAPN13* | ENSG00000162949 | -0.72 | 3.90E-05 | 7.93E-03 |
| *CXCL5* | ENSG00000163735 | 0.94 | 3.94E-05 | 7.93E-03 |
| *SPIRE1* | ENSG00000134278 | -0.32 | 4.01E-05 | 7.93E-03 |
| *RRAGD* | ENSG00000025039 | -0.37 | 4.02E-05 | 7.93E-03 |
| *TUBB6* | ENSG00000176014 | 0.62 | 4.03E-05 | 7.93E-03 |
| *PTGES* | ENSG00000148344 | 0.80 | 4.07E-05 | 7.93E-03 |
| *CPXM2* | ENSG00000121898 | 0.75 | 4.12E-05 | 7.93E-03 |
| *PCDHB15* | ENSG00000113248 | 0.36 | 4.16E-05 | 7.93E-03 |
| *UNC5D* | ENSG00000156687 | -0.81 | 4.50E-05 | 8.46E-03 |
| *CALCA* | ENSG00000110680 | 0.75 | 4.70E-05 | 8.61E-03 |
| *DUSP5* | ENSG00000138166 | 0.49 | 4.74E-05 | 8.61E-03 |
| *RP5-1033H22.2* | ENSG00000224093 | -0.50 | 4.80E-05 | 8.61E-03 |
| *FBLN1* | ENSG00000077942 | 0.96 | 4.82E-05 | 8.61E-03 |
| *MAMLD1* | ENSG00000013619 | 0.45 | 4.93E-05 | 8.70E-03 |
| *ANKRD36BP2* | ENSG00000230006 | -0.74 | 5.10E-05 | 8.84E-03 |
| *IL6* | ENSG00000136244 | 1.10 | 5.14E-05 | 8.84E-03 |
| *SERPINF1* | ENSG00000132386 | 0.83 | 5.30E-05 | 9.01E-03 |
| *PTPN3* | ENSG00000070159 | -0.35 | 5.40E-05 | 9.06E-03 |
| *IAPP* | ENSG00000121351 | -1.14 | 5.65E-05 | 9.38E-03 |
| *VSTM2L* | ENSG00000132821 | 0.71 | 5.78E-05 | 9.47E-03 |
| *ZADH2* | ENSG00000180011 | -0.21 | 5.84E-05 | 9.47E-03 |
| *NT5E* | ENSG00000135318 | 0.56 | 5.92E-05 | 9.49E-03 |
| *GPC3* | ENSG00000147257 | 0.96 | 6.11E-05 | 9.67E-03 |
| *FJX1* | ENSG00000179431 | 0.67 | 6.17E-05 | 9.67E-03 |
| *S100A6* | ENSG00000197956 | 0.80 | 6.25E-05 | 9.70E-03 |
| *NEBL* | ENSG00000078114 | -0.39 | 6.37E-05 | 9.77E-03 |
| *FANCI* | ENSG00000140525 | -0.46 | 6.80E-05 | 1.02E-02 |
| *ATP1A1-AS1* | ENSG00000203865 | -0.32 | 6.80E-05 | 1.02E-02 |
| *DPT* | ENSG00000143196 | 0.97 | 7.71E-05 | 1.15E-02 |
| *C4orf19* | ENSG00000154274 | -0.37 | 7.96E-05 | 1.17E-02 |
| *IL33* | ENSG00000137033 | 0.89 | 8.25E-05 | 1.20E-02 |
| *COBLL1* | ENSG00000082438 | -0.26 | 8.48E-05 | 1.22E-02 |
| *MMP10* | ENSG00000166670 | 0.97 | 8.64E-05 | 1.23E-02 |
| *GAD1* | ENSG00000128683 | 0.55 | 9.28E-05 | 1.31E-02 |
| *KIAA1107* | ENSG00000069712 | -0.38 | 9.40E-05 | 1.31E-02 |
| *APLNR* | ENSG00000134817 | 1.01 | 9.95E-05 | 1.37E-02 |
| *TFCP2L1* | ENSG00000115112 | -0.38 | 1.00E-04 | 1.37E-02 |
| *ERICH5* | ENSG00000177459 | -0.35 | 1.02E-04 | 1.38E-02 |
| *ACRBP* | ENSG00000111644 | -0.45 | 1.07E-04 | 1.42E-02 |
| *CORO2B* | ENSG00000103647 | 0.38 | 1.07E-04 | 1.42E-02 |
| *IL1R2* | ENSG00000115590 | 0.90 | 1.09E-04 | 1.44E-02 |
| *CKAP2* | ENSG00000136108 | -0.33 | 1.18E-04 | 1.53E-02 |
| *ELN* | ENSG00000049540 | 0.80 | 1.18E-04 | 1.53E-02 |
| *CST2* | ENSG00000170369 | 1.04 | 1.20E-04 | 1.53E-02 |
| *NAV3* | ENSG00000067798 | 0.44 | 1.20E-04 | 1.53E-02 |
| *INPP5F* | ENSG00000198825 | -0.35 | 1.25E-04 | 1.56E-02 |
| *SEPP1* | ENSG00000250722 | -0.49 | 1.26E-04 | 1.56E-02 |
| *RCBTB1* | ENSG00000136144 | -0.24 | 1.28E-04 | 1.56E-02 |
| *SRGN* | ENSG00000122862 | 0.60 | 1.30E-04 | 1.56E-02 |
| *FOSL1* | ENSG00000175592 | 0.98 | 1.30E-04 | 1.56E-02 |
| *BHMT2* | ENSG00000132840 | 0.41 | 1.31E-04 | 1.56E-02 |
| *COTL1* | ENSG00000103187 | 0.42 | 1.32E-04 | 1.56E-02 |
| *SOX6* | ENSG00000110693 | -0.37 | 1.33E-04 | 1.56E-02 |
| *CALY* | ENSG00000130643 | 0.69 | 1.33E-04 | 1.56E-02 |
| *NR4A3* | ENSG00000119508 | 0.63 | 1.46E-04 | 1.70E-02 |
| *SIRPA* | ENSG00000198053 | 0.40 | 1.53E-04 | 1.76E-02 |
| *RP11-1277A3.2* | ENSG00000246596 | -0.52 | 1.56E-04 | 1.76E-02 |
| *LPAR1* | ENSG00000198121 | 0.46 | 1.58E-04 | 1.76E-02 |
| *MRTO4* | ENSG00000053372 | 0.36 | 1.58E-04 | 1.76E-02 |
| *SERPINB9* | ENSG00000170542 | 0.41 | 1.58E-04 | 1.76E-02 |
| *DPYSL3* | ENSG00000113657 | 0.37 | 1.59E-04 | 1.76E-02 |
| *TBC1D4* | ENSG00000136111 | -0.32 | 1.66E-04 | 1.82E-02 |
| *COMMD9* | ENSG00000110442 | -0.21 | 1.69E-04 | 1.82E-02 |
| *FAM105A* | ENSG00000145569 | -0.53 | 1.69E-04 | 1.82E-02 |
| *ARHGAP6* | ENSG00000047648 | 0.54 | 1.72E-04 | 1.82E-02 |
| *ITGA9-AS1* | ENSG00000235257 | -0.43 | 1.72E-04 | 1.82E-02 |
| *FXYD2* | ENSG00000137731 | -0.64 | 1.73E-04 | 1.82E-02 |
| *TMEM27* | ENSG00000147003 | -0.42 | 1.74E-04 | 1.82E-02 |
| *PTX3* | ENSG00000163661 | 0.75 | 1.76E-04 | 1.82E-02 |
| *LAT2* | ENSG00000086730 | 0.39 | 1.77E-04 | 1.82E-02 |
| *IL18R1* | ENSG00000115604 | 0.58 | 1.77E-04 | 1.82E-02 |
| *DHFR* | ENSG00000228716 | -0.50 | 1.79E-04 | 1.82E-02 |
| *CDC20* | ENSG00000117399 | -0.76 | 1.81E-04 | 1.83E-02 |
| *FCN3* | ENSG00000142748 | 1.17 | 1.82E-04 | 1.83E-02 |
| *KB-1732A1.1* | ENSG00000253669 | 0.53 | 1.84E-04 | 1.84E-02 |
| *TNFRSF11A* | ENSG00000141655 | 0.32 | 1.88E-04 | 1.87E-02 |
| *KIAA0020* | ENSG00000080608 | 0.24 | 1.94E-04 | 1.91E-02 |
| *RRP12* | ENSG00000052749 | 0.40 | 1.97E-04 | 1.93E-02 |
| *ACP2* | ENSG00000134575 | 0.27 | 2.00E-04 | 1.94E-02 |
| *NCS1* | ENSG00000107130 | 0.41 | 2.06E-04 | 1.99E-02 |
| *DCN* | ENSG00000011465 | 0.69 | 2.14E-04 | 2.05E-02 |
| *IL11* | ENSG00000095752 | 0.79 | 2.17E-04 | 2.05E-02 |
| *GRPEL1* | ENSG00000109519 | 0.22 | 2.17E-04 | 2.05E-02 |
| *KCNC1* | ENSG00000129159 | 0.49 | 2.24E-04 | 2.11E-02 |
| *F10* | ENSG00000126218 | 0.54 | 2.27E-04 | 2.12E-02 |
| *SASS6* | ENSG00000156876 | -0.26 | 2.36E-04 | 2.19E-02 |
| *BTBD3* | ENSG00000132640 | -0.29 | 2.42E-04 | 2.23E-02 |
| *ARSJ* | ENSG00000180801 | 0.37 | 2.46E-04 | 2.26E-02 |
| *RRP9* | ENSG00000114767 | 0.41 | 2.56E-04 | 2.32E-02 |
| *ROR2* | ENSG00000169071 | 0.54 | 2.57E-04 | 2.32E-02 |
| *GDPD1* | ENSG00000153982 | -0.33 | 2.62E-04 | 2.33E-02 |
| *CHST15* | ENSG00000182022 | 0.38 | 2.62E-04 | 2.33E-02 |
| *GNAL* | ENSG00000141404 | 0.42 | 2.63E-04 | 2.33E-02 |
| *KLHL12* | ENSG00000117153 | -0.18 | 2.66E-04 | 2.34E-02 |
| *NCOA3* | ENSG00000124151 | -0.21 | 2.67E-04 | 2.34E-02 |
| *SLC38A5* | ENSG00000017483 | 0.66 | 2.71E-04 | 2.34E-02 |
| *ANKRD13A* | ENSG00000076513 | -0.22 | 2.73E-04 | 2.34E-02 |
| *GLP1R* | ENSG00000112164 | -0.66 | 2.73E-04 | 2.34E-02 |
| *HIATL2* | ENSG00000196312 | -0.27 | 2.87E-04 | 2.45E-02 |
| *BMPR1B* | ENSG00000138696 | 0.48 | 2.94E-04 | 2.50E-02 |
| *CELP* | ENSG00000170827 | 1.16 | 3.00E-04 | 2.54E-02 |
| *SLC34A2* | ENSG00000157765 | 0.63 | 3.04E-04 | 2.55E-02 |
| *RAB3IP* | ENSG00000127328 | -0.18 | 3.10E-04 | 2.57E-02 |
| *GPAM* | ENSG00000119927 | -0.25 | 3.11E-04 | 2.57E-02 |
| *AIFM2* | ENSG00000042286 | 0.28 | 3.12E-04 | 2.57E-02 |
| *MST1R* | ENSG00000164078 | 0.69 | 3.16E-04 | 2.59E-02 |
| *MGAT4A* | ENSG00000071073 | -0.28 | 3.22E-04 | 2.62E-02 |
| *PIEZO2* | ENSG00000154864 | -1.09 | 3.28E-04 | 2.65E-02 |
| *EDNRB* | ENSG00000136160 | 0.72 | 3.29E-04 | 2.65E-02 |
| *PARK2* | ENSG00000185345 | -0.35 | 3.31E-04 | 2.65E-02 |
| *CCDC86* | ENSG00000110104 | 0.38 | 3.36E-04 | 2.65E-02 |
| *SLC35D3* | ENSG00000182747 | 0.57 | 3.37E-04 | 2.65E-02 |
| *KLF12* | ENSG00000118922 | -0.32 | 3.38E-04 | 2.65E-02 |
| *ADPGK* | ENSG00000159322 | 0.17 | 3.38E-04 | 2.65E-02 |
| *VPS11* | ENSG00000160695 | -0.23 | 3.40E-04 | 2.65E-02 |
| *TUBB4B* | ENSG00000188229 | 0.55 | 3.43E-04 | 2.66E-02 |
| *PDE4B* | ENSG00000184588 | 0.55 | 3.47E-04 | 2.67E-02 |
| *CMSS1* | ENSG00000184220 | 0.33 | 3.50E-04 | 2.68E-02 |
| *S100A10* | ENSG00000197747 | 0.60 | 3.55E-04 | 2.70E-02 |
| *CHST2* | ENSG00000175040 | 0.52 | 3.58E-04 | 2.70E-02 |
| *USP43* | ENSG00000154914 | 0.39 | 3.58E-04 | 2.70E-02 |
| *LDLRAD3* | ENSG00000179241 | 0.33 | 3.67E-04 | 2.75E-02 |
| *SPARCL1* | ENSG00000152583 | 0.87 | 3.77E-04 | 2.81E-02 |
| *TWIST2* | ENSG00000233608 | 0.67 | 3.79E-04 | 2.82E-02 |
| *LAMC2* | ENSG00000058085 | 0.71 | 3.84E-04 | 2.82E-02 |
| *MALL* | ENSG00000144063 | 0.68 | 3.84E-04 | 2.82E-02 |
| *ITGBL1* | ENSG00000198542 | 0.84 | 3.87E-04 | 2.83E-02 |
| *BCL2L1* | ENSG00000171552 | 0.29 | 3.94E-04 | 2.85E-02 |
| *PRC1* | ENSG00000198901 | -0.56 | 3.94E-04 | 2.85E-02 |
| *KCNJ16* | ENSG00000153822 | -0.80 | 4.02E-04 | 2.89E-02 |
| *SULT2B1* | ENSG00000088002 | 0.93 | 4.14E-04 | 2.97E-02 |
| *DACH2* | ENSG00000126733 | -0.44 | 4.19E-04 | 2.99E-02 |
| *GRWD1* | ENSG00000105447 | 0.32 | 4.22E-04 | 3.00E-02 |
| *RRS1* | ENSG00000179041 | 0.40 | 4.39E-04 | 3.10E-02 |
| *PAQR5* | ENSG00000137819 | 0.50 | 4.44E-04 | 3.12E-02 |
| *ZNRF1* | ENSG00000186187 | 0.34 | 4.50E-04 | 3.14E-02 |
| *NRG1* | ENSG00000157168 | 0.43 | 4.61E-04 | 3.21E-02 |
| *TMEM237* | ENSG00000155755 | -0.23 | 4.66E-04 | 3.22E-02 |
| *BPIFC* | ENSG00000184459 | -0.74 | 4.69E-04 | 3.23E-02 |
| *THBS2* | ENSG00000186340 | 0.61 | 4.75E-04 | 3.25E-02 |
| *TRIQK* | ENSG00000205133 | -0.27 | 4.79E-04 | 3.26E-02 |
| *RP11-693N9.2* | ENSG00000235505 | -0.77 | 4.84E-04 | 3.28E-02 |
| *CDKN1A* | ENSG00000124762 | 0.39 | 4.88E-04 | 3.30E-02 |
| *POLD2* | ENSG00000106628 | 0.36 | 4.94E-04 | 3.31E-02 |
| *DIXDC1* | ENSG00000150764 | -0.24 | 4.95E-04 | 3.31E-02 |
| *TPX2* | ENSG00000088325 | -0.71 | 5.03E-04 | 3.35E-02 |
| *RP11-96H19.1* | ENSG00000257261 | -0.49 | 5.13E-04 | 3.37E-02 |
| *ADRA2C* | ENSG00000184160 | 0.81 | 5.14E-04 | 3.37E-02 |
| *SOD3* | ENSG00000109610 | 0.65 | 5.15E-04 | 3.37E-02 |
| *INPP1* | ENSG00000151689 | 0.17 | 5.15E-04 | 3.37E-02 |
| *PDE1A* | ENSG00000115252 | 0.50 | 5.19E-04 | 3.38E-02 |
| *ARNTL* | ENSG00000133794 | -0.20 | 5.25E-04 | 3.40E-02 |
| *HCN4* | ENSG00000138622 | -0.46 | 5.39E-04 | 3.47E-02 |
| *EPHB2* | ENSG00000133216 | 0.46 | 5.41E-04 | 3.47E-02 |
| *HMOX1* | ENSG00000100292 | 0.90 | 5.43E-04 | 3.47E-02 |
| *ABCC8* | ENSG00000006071 | -0.71 | 5.46E-04 | 3.47E-02 |
| *CTDSPL* | ENSG00000144677 | -0.23 | 5.49E-04 | 3.47E-02 |
| *PTTG1IP* | ENSG00000183255 | 0.24 | 5.58E-04 | 3.52E-02 |
| *CBLC* | ENSG00000142273 | 0.51 | 5.63E-04 | 3.53E-02 |
| *ABAT* | ENSG00000183044 | -0.39 | 5.70E-04 | 3.54E-02 |
| *SNRNP48* | ENSG00000168566 | -0.15 | 5.70E-04 | 3.54E-02 |
| *APC* | ENSG00000134982 | -0.24 | 5.79E-04 | 3.58E-02 |
| *HEXB* | ENSG00000049860 | -0.20 | 5.89E-04 | 3.63E-02 |
| *GAP43* | ENSG00000172020 | 0.50 | 6.10E-04 | 3.74E-02 |
| *LINC01091* | ENSG00000249464 | -0.50 | 6.24E-04 | 3.81E-02 |
| *CDK1* | ENSG00000170312 | -0.81 | 6.28E-04 | 3.82E-02 |
| *FAM8A1* | ENSG00000137414 | -0.20 | 6.42E-04 | 3.89E-02 |
| *IL1R1* | ENSG00000115594 | 0.33 | 6.49E-04 | 3.91E-02 |
| *NDRG3* | ENSG00000101079 | -0.22 | 6.53E-04 | 3.92E-02 |
| *COMP* | ENSG00000105664 | 0.86 | 6.58E-04 | 3.92E-02 |
| *ABI3BP* | ENSG00000154175 | 0.48 | 6.58E-04 | 3.92E-02 |
| *DENND2A* | ENSG00000146966 | -0.36 | 6.61E-04 | 3.92E-02 |
| *SH3BGRL* | ENSG00000131171 | -0.23 | 6.66E-04 | 3.93E-02 |
| *LYAR* | ENSG00000145220 | 0.34 | 6.96E-04 | 4.08E-02 |
| *SCD* | ENSG00000099194 | -0.37 | 6.96E-04 | 4.08E-02 |
| *NECAB2* | ENSG00000103154 | 0.51 | 7.06E-04 | 4.10E-02 |
| *LIF* | ENSG00000128342 | 0.64 | 7.11E-04 | 4.10E-02 |
| *ENTPD6* | ENSG00000197586 | 0.27 | 7.12E-04 | 4.10E-02 |
| *CMTM7* | ENSG00000153551 | 0.37 | 7.13E-04 | 4.10E-02 |
| *ATP6V1A* | ENSG00000114573 | -0.20 | 7.25E-04 | 4.13E-02 |
| *C7* | ENSG00000112936 | 1.25 | 7.25E-04 | 4.13E-02 |
| *BEST3* | ENSG00000127325 | -0.67 | 7.26E-04 | 4.13E-02 |
| *DKK3* | ENSG00000050165 | 0.59 | 7.36E-04 | 4.17E-02 |
| *NOP16* | ENSG00000048162 | 0.36 | 7.41E-04 | 4.17E-02 |
| *ANXA5* | ENSG00000164111 | 0.28 | 7.43E-04 | 4.17E-02 |
| *PDGFRA* | ENSG00000134853 | 0.61 | 7.45E-04 | 4.17E-02 |
| *FAM122C* | ENSG00000156500 | -0.30 | 7.47E-04 | 4.17E-02 |
| *FBLN5* | ENSG00000140092 | 0.43 | 7.64E-04 | 4.24E-02 |
| *GNG7* | ENSG00000176533 | -0.42 | 7.68E-04 | 4.25E-02 |
| *MYC* | ENSG00000136997 | 0.41 | 7.83E-04 | 4.31E-02 |
| *MESDC2* | ENSG00000117899 | 0.12 | 7.92E-04 | 4.32E-02 |
| *PID1* | ENSG00000153823 | 0.46 | 7.92E-04 | 4.32E-02 |
| *HYKK* | ENSG00000188266 | -0.27 | 7.94E-04 | 4.32E-02 |
| *NUDT7* | ENSG00000140876 | -0.37 | 8.01E-04 | 4.34E-02 |
| *RACGAP1* | ENSG00000161800 | -0.37 | 8.24E-04 | 4.44E-02 |
| *C4orf33* | ENSG00000151470 | -0.27 | 8.24E-04 | 4.44E-02 |
| *COG6* | ENSG00000133103 | -0.27 | 8.38E-04 | 4.49E-02 |
| *GLB1L2* | ENSG00000149328 | 0.24 | 8.50E-04 | 4.54E-02 |
| *IL22RA1* | ENSG00000142677 | 0.70 | 8.59E-04 | 4.57E-02 |
| *AC004381.6* | ENSG00000005189 | -0.34 | 8.61E-04 | 4.57E-02 |
| *ACADSB* | ENSG00000196177 | -0.27 | 8.77E-04 | 4.63E-02 |
| *HMGA1* | ENSG00000137309 | 0.72 | 8.80E-04 | 4.63E-02 |
| *GCNT4* | ENSG00000176928 | -0.35 | 8.93E-04 | 4.67E-02 |
| *ANKRD50* | ENSG00000151458 | -0.28 | 8.94E-04 | 4.67E-02 |
| *RP11-680F8.1* | ENSG00000256802 | 0.55 | 9.00E-04 | 4.69E-02 |
| *S100A16* | ENSG00000188643 | 0.54 | 9.07E-04 | 4.70E-02 |
| *LINC01099* | ENSG00000251504 | -0.82 | 9.09E-04 | 4.70E-02 |
| *SNRPA* | ENSG00000077312 | 0.34 | 9.16E-04 | 4.72E-02 |
| *BAG1* | ENSG00000107262 | 0.24 | 9.21E-04 | 4.73E-02 |
| *ATP4A* | ENSG00000105675 | 1.25 | 9.35E-04 | 4.78E-02 |
| *DNAJB4* | ENSG00000162616 | -0.31 | 9.40E-04 | 4.78E-02 |
| *NUDT12* | ENSG00000112874 | -0.21 | 9.42E-04 | 4.78E-02 |
| *SGOL2* | ENSG00000163535 | -0.34 | 9.45E-04 | 4.78E-02 |
| *KBTBD3* | ENSG00000182359 | -0.29 | 9.52E-04 | 4.80E-02 |
| *ITGA11* | ENSG00000137809 | -1.22 | 9.62E-04 | 4.83E-02 |
| *SLC4A8* | ENSG00000050438 | -0.45 | 9.76E-04 | 4.88E-02 |
| *KIAA1109* | ENSG00000138688 | -0.28 | 1.01E-03 | 5.00E-02 |
| *FBXL20* | ENSG00000108306 | -0.28 | 1.01E-03 | 5.00E-02 |
| *SEPT9* | ENSG00000184640 | 0.36 | 1.01E-03 | 5.00E-02 |
