# Supplementary material for: Islet Gene View—a tool to facilitate islet research
Source: Life Sci Alliance. 2022 Aug 10;5(12):e202201376. doi: 10.26508/lsa.202201376 (PMC9366203; doi:10.26508/lsa.202201376)
Supplement: Supplementary file 3 [file LSA-2022-01376_TableS3.docx]

Supplementary table 3: Correlation of differentially expressed genes (T2D vs non-T2D, from supplementary table 2) with HbA1c levels.

| **HGNC symbol** | **beta** | **average_expression** | **t** | **p_value** | **adjusted_pvalue** |
| --- | --- | --- | --- | --- | --- |
| *OPRD1* | -0.695 | 3.411 | -6.045 | 9.55E-09 | 8.11E-05 |
| *SLC2A2* | -0.720 | 2.996 | -6.008 | 1.15E-08 | 8.11E-05 |
| *PLA1A* | -0.487 | 1.596 | -5.348 | 2.90E-07 | 1.13E-03 |
| *CPXM2* | 0.549 | 1.641 | 5.328 | 3.19E-07 | 1.13E-03 |
| *SERPINE2* | 0.289 | 6.498 | 5.213 | 5.45E-07 | 1.53E-03 |
| *HHATL* | -0.603 | 1.068 | -5.147 | 7.39E-07 | 1.53E-03 |
| *PPP1R1A* | -0.485 | 7.472 | -5.142 | 7.58E-07 | 1.53E-03 |
| *SFRP1* | 0.575 | 3.540 | 5.061 | 1.10E-06 | 1.93E-03 |
| *RASGRP1* | -0.354 | 4.053 | -4.756 | 4.27E-06 | 6.69E-03 |
| *VASH2* | 0.353 | 1.425 | 4.524 | 1.15E-05 | 1.48E-02 |
| *GLRA1* | -0.658 | 2.434 | -4.523 | 1.15E-05 | 1.48E-02 |
| *BHMT2* | 0.281 | 3.178 | 4.501 | 1.27E-05 | 1.49E-02 |
| *HS6ST2* | -0.307 | 3.337 | -4.364 | 2.24E-05 | 2.43E-02 |
| *GRAMD3* | -0.151 | 4.968 | -4.319 | 2.68E-05 | 2.47E-02 |
| *CALCA* | 0.436 | 0.457 | 4.297 | 2.93E-05 | 2.47E-02 |
| *PTPN3* | -0.200 | 6.993 | -4.294 | 2.97E-05 | 2.47E-02 |
| *FFAR4* | -0.436 | 3.377 | -4.228 | 3.88E-05 | 3.04E-02 |
| *CHL1* | -0.543 | 3.511 | -4.207 | 4.22E-05 | 3.09E-02 |
| *SPON1* | 0.408 | 5.706 | 4.152 | 5.27E-05 | 3.54E-02 |
| *SRGN* | 0.355 | 4.552 | 4.078 | 7.04E-05 | 4.52E-02 |
| *TBC1D4* | -0.191 | 6.164 | -4.045 | 8.00E-05 | 4.85E-02 |
| *DKK3* | 0.394 | 5.477 | 4.036 | 8.29E-05 | 4.85E-02 |
| *CTSV* | -0.310 | 1.636 | -4.026 | 8.60E-05 | 4.85E-02 |
| *UNC5D* | -0.443 | 3.399 | -3.964 | 1.09E-04 | 5.93E-02 |
| *CST2* | 0.618 | -0.462 | 3.897 | 1.41E-04 | 7.38E-02 |
| *CAPN13* | -0.390 | 5.092 | -3.815 | 1.92E-04 | 9.67E-02 |
| *RRAGD* | -0.194 | 7.065 | -3.789 | 2.11E-04 | 1.03E-01 |
| *APOLD1* | 0.351 | 3.372 | 3.748 | 2.46E-04 | 1.16E-01 |
| *RP5-1033H22.2* | -0.256 | 2.104 | -3.685 | 3.09E-04 | 1.36E-01 |
| *GAD1* | 0.317 | 2.175 | 3.670 | 3.26E-04 | 1.36E-01 |
| *PDGFRA* | 0.377 | 4.403 | 3.633 | 3.73E-04 | 1.46E-01 |
| *FGF7* | 0.451 | 2.996 | 3.600 | 4.20E-04 | 1.60E-01 |
| *PCDHB15* | 0.187 | 2.239 | 3.536 | 5.27E-04 | 1.93E-01 |
| *GLP1R* | -0.362 | 4.998 | -3.524 | 5.49E-04 | 1.93E-01 |
| *PPFIBP2* | -0.226 | 3.881 | -3.514 | 5.69E-04 | 1.93E-01 |
| *ERICH5* | -0.187 | 4.544 | -3.511 | 5.76E-04 | 1.93E-01 |
| *THBS2* | 0.349 | 6.956 | 3.483 | 6.33E-04 | 2.08E-01 |
| *DACH2* | -0.251 | 2.728 | -3.457 | 6.93E-04 | 2.17E-01 |
| *TMEM27* | -0.218 | 3.325 | -3.431 | 7.58E-04 | 2.23E-01 |
| *KCNAB2* | 0.225 | 2.719 | 3.430 | 7.60E-04 | 2.23E-01 |
| *APOD* | 0.537 | 3.651 | 3.417 | 7.95E-04 | 2.29E-01 |
| *CLMP* | 0.346 | 3.941 | 3.397 | 8.52E-04 | 2.36E-01 |
| *BPIFC* | -0.395 | 1.715 | -3.354 | 9.86E-04 | 2.63E-01 |
| *NT5E* | 0.275 | 4.875 | 3.338 | 1.04E-03 | 2.71E-01 |
| *MMP10* | 0.485 | 5.019 | 3.328 | 1.08E-03 | 2.71E-01 |
| *SLC4A8* | -0.258 | 5.519 | -3.315 | 1.12E-03 | 2.73E-01 |
| *HCN4* | -0.258 | 3.082 | -3.305 | 1.16E-03 | 2.76E-01 |
| *GREM2* | -0.490 | 1.636 | -3.301 | 1.18E-03 | 2.76E-01 |
| *IL1RL1* | 0.561 | 3.821 | 3.294 | 1.21E-03 | 2.76E-01 |
| *DPYSL3* | 0.182 | 6.037 | 3.292 | 1.21E-03 | 2.76E-01 |
| *TFCP2L1* | -0.190 | 4.420 | -3.237 | 1.46E-03 | 3.00E-01 |
| *RP11-395G23.3* | 0.229 | 1.306 | 3.235 | 1.47E-03 | 3.00E-01 |
| *SMOC2* | 0.709 | 1.035 | 3.228 | 1.50E-03 | 3.02E-01 |
| *ARG2* | -0.260 | 4.647 | -3.218 | 1.55E-03 | 3.04E-01 |
| *CCL22* | 0.594 | 1.024 | 3.211 | 1.59E-03 | 3.04E-01 |
| *IL11* | 0.396 | 7.599 | 3.199 | 1.65E-03 | 3.04E-01 |
| *MAP2K6* | -0.180 | 1.924 | -3.196 | 1.67E-03 | 3.04E-01 |
| *RAB39A* | -0.220 | 2.491 | -3.191 | 1.70E-03 | 3.04E-01 |
| *BTBD3* | -0.142 | 7.046 | -3.148 | 1.95E-03 | 3.31E-01 |
| *MIA2* | -0.211 | 0.679 | -3.139 | 2.00E-03 | 3.36E-01 |
| *IL33* | 0.409 | 5.029 | 3.131 | 2.06E-03 | 3.42E-01 |
| *IL1R2* | 0.422 | 1.072 | 3.091 | 2.34E-03 | 3.61E-01 |
| *LPAR1* | 0.219 | 3.414 | 3.085 | 2.38E-03 | 3.61E-01 |
| *C4orf19* | -0.168 | 4.501 | -3.082 | 2.41E-03 | 3.61E-01 |
| *IAPP* | -0.497 | 10.140 | -3.080 | 2.42E-03 | 3.61E-01 |
| *SPIRE1* | -0.139 | 6.265 | -3.072 | 2.49E-03 | 3.66E-01 |
| *RP11-1277A3.2* | -0.247 | 0.143 | -3.066 | 2.53E-03 | 3.66E-01 |
| *ZADH2* | -0.094 | 5.847 | -3.064 | 2.54E-03 | 3.66E-01 |
| *SYNDIG1* | 0.292 | 2.322 | 3.062 | 2.57E-03 | 3.66E-01 |
| *SH2D2A* | 0.318 | 1.089 | 3.020 | 2.93E-03 | 4.09E-01 |
| *SERPINF1* | 0.365 | 4.024 | 3.011 | 3.01E-03 | 4.09E-01 |
| *CHRDL1* | 0.433 | 1.503 | 2.994 | 3.17E-03 | 4.26E-01 |
| *NEBL* | -0.168 | 6.721 | -2.984 | 3.27E-03 | 4.35E-01 |
| *FSTL4* | 0.236 | 4.003 | 2.956 | 3.57E-03 | 4.45E-01 |
| *TAC1* | 0.408 | 1.505 | 2.955 | 3.58E-03 | 4.45E-01 |
| *MESDC2* | 0.059 | 6.209 | 2.950 | 3.63E-03 | 4.45E-01 |
| *CORO2B* | 0.172 | 3.970 | 2.944 | 3.70E-03 | 4.45E-01 |
| *GAP43* | 0.256 | 2.160 | 2.942 | 3.72E-03 | 4.45E-01 |
| *SOX6* | -0.161 | 4.316 | -2.923 | 3.95E-03 | 4.48E-01 |
| *PDE1A* | 0.248 | 2.176 | 2.921 | 3.97E-03 | 4.48E-01 |
| *ARL4C* | 0.159 | 5.903 | 2.916 | 4.04E-03 | 4.52E-01 |
| *ATP6V1A* | -0.092 | 7.471 | -2.870 | 4.64E-03 | 4.85E-01 |
| *KAZN* | 0.173 | 2.531 | 2.854 | 4.87E-03 | 5.01E-01 |
| *COBLL1* | -0.110 | 6.162 | -2.850 | 4.92E-03 | 5.03E-01 |
| *SCAI* | -0.121 | 3.982 | -2.840 | 5.07E-03 | 5.11E-01 |
| *PARK2* | -0.163 | 2.400 | -2.827 | 5.28E-03 | 5.22E-01 |
| *ACRBP* | -0.199 | 1.807 | -2.820 | 5.38E-03 | 5.22E-01 |
| *FXYD2* | -0.291 | 2.897 | -2.815 | 5.47E-03 | 5.22E-01 |
| *ROR2* | 0.255 | 2.142 | 2.814 | 5.49E-03 | 5.22E-01 |
| *SSTR5-AS1* | 0.334 | 3.268 | 2.799 | 5.74E-03 | 5.32E-01 |
| *MEDAG* | 0.391 | 5.102 | 2.785 | 5.97E-03 | 5.37E-01 |
| *GCNT4* | -0.168 | 4.914 | -2.782 | 6.03E-03 | 5.37E-01 |
| *MPP1* | -0.097 | 5.247 | -2.765 | 6.33E-03 | 5.55E-01 |
| *GNAL* | 0.198 | 2.410 | 2.764 | 6.36E-03 | 5.55E-01 |
| *SFRP4* | 0.612 | 1.269 | 2.760 | 6.42E-03 | 5.55E-01 |
| *DHFR* | -0.208 | 2.552 | -2.758 | 6.47E-03 | 5.55E-01 |
| *DCN* | 0.296 | 5.395 | 2.729 | 7.04E-03 | 5.80E-01 |
| *NRG1* | 0.199 | 2.260 | 2.716 | 7.32E-03 | 5.86E-01 |
| *FAM105A* | -0.221 | 6.001 | -2.699 | 7.67E-03 | 5.93E-01 |
| *INPP5F* | -0.145 | 6.740 | -2.690 | 7.88E-03 | 6.04E-01 |
| *CHST15* | 0.163 | 4.684 | 2.688 | 7.92E-03 | 6.04E-01 |
| *ORC6* | -0.180 | 0.502 | -2.676 | 8.21E-03 | 6.04E-01 |
| *IL1R1* | 0.145 | 7.334 | 2.663 | 8.50E-03 | 6.04E-01 |
| *IL6* | 0.428 | 3.502 | 2.663 | 8.50E-03 | 6.04E-01 |
| *BEST3* | -0.302 | 1.652 | -2.662 | 8.54E-03 | 6.04E-01 |
| *GLB1L2* | 0.114 | 3.903 | 2.651 | 8.80E-03 | 6.04E-01 |
| *RASD2* | 0.350 | 2.553 | 2.649 | 8.86E-03 | 6.04E-01 |
| *CRH* | 0.335 | 2.112 | 2.645 | 8.95E-03 | 6.04E-01 |
| *AASS* | -0.139 | 3.133 | -2.639 | 9.10E-03 | 6.04E-01 |
| *APC* | -0.102 | 6.691 | -2.639 | 9.11E-03 | 6.04E-01 |
| *RAB3IP* | -0.075 | 6.314 | -2.633 | 9.27E-03 | 6.06E-01 |
| *ARHGAP6* | 0.214 | 1.875 | 2.571 | 1.10E-02 | 6.83E-01 |
| *ARNTL* | -0.086 | 4.654 | -2.564 | 1.12E-02 | 6.90E-01 |
| *LIF* | 0.278 | 7.234 | 2.524 | 1.25E-02 | 7.16E-01 |
| *CTDSPL* | -0.098 | 5.530 | -2.517 | 1.28E-02 | 7.16E-01 |
| *GNG7* | -0.188 | 3.791 | -2.510 | 1.30E-02 | 7.18E-01 |
| *PODN* | 0.314 | 2.298 | 2.509 | 1.31E-02 | 7.18E-01 |
| *ABCC8* | -0.292 | 8.709 | -2.503 | 1.33E-02 | 7.18E-01 |
| *KIAA0020* | 0.094 | 5.388 | 2.501 | 1.34E-02 | 7.18E-01 |
| *FBLN1* | 0.361 | 3.918 | 2.498 | 1.35E-02 | 7.18E-01 |
| *GPC3* | 0.352 | 1.644 | 2.493 | 1.37E-02 | 7.18E-01 |
| *PBLD* | -0.190 | 3.744 | -2.490 | 1.38E-02 | 7.18E-01 |
| *C1QTNF1* | 0.271 | 2.263 | 2.485 | 1.39E-02 | 7.23E-01 |
| *NCOA3* | -0.078 | 6.500 | -2.471 | 1.45E-02 | 7.34E-01 |
| *IL18R1* | 0.229 | 1.892 | 2.455 | 1.51E-02 | 7.46E-01 |
| *ANKRD36BP2* | -0.272 | 1.260 | -2.448 | 1.54E-02 | 7.46E-01 |
| *SLC22A15* | -0.128 | 4.289 | -2.427 | 1.63E-02 | 7.60E-01 |
| *FANCI* | -0.167 | 3.322 | -2.426 | 1.63E-02 | 7.60E-01 |
| *CDKN1A* | 0.151 | 8.177 | 2.416 | 1.68E-02 | 7.72E-01 |
| *ANKRD50* | -0.109 | 5.413 | -2.414 | 1.68E-02 | 7.72E-01 |
| *BMX* | 0.373 | 1.245 | 2.411 | 1.70E-02 | 7.73E-01 |
| *PTGDS* | 0.460 | 1.658 | 2.396 | 1.77E-02 | 7.80E-01 |
| *ANKRD13A* | -0.082 | 6.236 | -2.393 | 1.78E-02 | 7.80E-01 |
| *SPARCL1* | 0.341 | 4.674 | 2.345 | 2.02E-02 | 8.17E-01 |
| *MGAT4A* | -0.104 | 7.247 | -2.342 | 2.03E-02 | 8.20E-01 |
| *F10* | 0.202 | 4.837 | 2.339 | 2.05E-02 | 8.20E-01 |
| *LINC01091* | -0.198 | 0.800 | -2.313 | 2.19E-02 | 8.46E-01 |
| *MIS18BP1* | -0.087 | 4.393 | -2.302 | 2.26E-02 | 8.46E-01 |
| *COMMD9* | -0.073 | 5.248 | -2.298 | 2.28E-02 | 8.46E-01 |
| *KLHL12* | -0.064 | 6.014 | -2.289 | 2.33E-02 | 8.57E-01 |
| *KB-1732A1.1* | 0.195 | 0.882 | 2.272 | 2.44E-02 | 8.63E-01 |
| *ABAT* | -0.143 | 5.687 | -2.268 | 2.46E-02 | 8.63E-01 |
| *BMP6* | 0.255 | 1.660 | 2.265 | 2.48E-02 | 8.63E-01 |
| *ATP1A1-AS1* | -0.105 | 1.731 | -2.264 | 2.49E-02 | 8.63E-01 |
| *TUBB6* | 0.198 | 5.584 | 2.264 | 2.49E-02 | 8.63E-01 |
| *HYKK* | -0.108 | 1.527 | -2.243 | 2.62E-02 | 8.77E-01 |
| *APLNR* | 0.345 | 1.974 | 2.242 | 2.63E-02 | 8.77E-01 |
| *TWIST2* | 0.257 | 0.931 | 2.236 | 2.67E-02 | 8.82E-01 |
| *ACADSB* | -0.105 | 5.103 | -2.230 | 2.71E-02 | 8.87E-01 |
| *CTSF* | 0.126 | 4.165 | 2.229 | 2.72E-02 | 8.87E-01 |
| *TMEM158* | 0.238 | 3.369 | 2.225 | 2.74E-02 | 8.90E-01 |
| *PTGES* | 0.265 | 3.397 | 2.216 | 2.81E-02 | 8.98E-01 |
| *C7* | 0.459 | 6.123 | 2.213 | 2.82E-02 | 8.98E-01 |
| *MFAP4* | 0.315 | 3.174 | 2.194 | 2.96E-02 | 9.15E-01 |
| *CKAP2* | -0.113 | 3.729 | -2.190 | 2.99E-02 | 9.22E-01 |
| *GPAM* | -0.087 | 4.159 | -2.168 | 3.16E-02 | 9.47E-01 |
| *KIAA1109* | -0.103 | 7.182 | -2.151 | 3.29E-02 | 9.54E-01 |
| *NR4A3* | 0.216 | 3.219 | 2.135 | 3.42E-02 | 9.64E-01 |
| *SOD3* | 0.241 | 3.685 | 2.131 | 3.45E-02 | 9.69E-01 |
| *CALY* | 0.244 | 2.910 | 2.121 | 3.54E-02 | 9.81E-01 |
| *FGFBP1* | 0.376 | 3.724 | 2.108 | 3.65E-02 | 9.81E-01 |
| *ITGBL1* | 0.306 | 0.645 | 2.080 | 3.91E-02 | 9.87E-01 |
| *COG6* | -0.096 | 5.813 | -2.072 | 3.98E-02 | 9.92E-01 |
| *KIAA1107* | -0.120 | 4.151 | -2.067 | 4.03E-02 | 9.92E-01 |
| *EDNRB* | 0.231 | 6.176 | 2.065 | 4.04E-02 | 9.92E-01 |
| *S100A6* | 0.229 | 8.074 | 2.051 | 4.18E-02 | 1.00E+00 |
| *MRTO4* | 0.115 | 5.171 | 2.050 | 4.19E-02 | 1.00E+00 |
| *ADPGK* | 0.053 | 5.487 | 2.030 | 4.39E-02 | 1.00E+00 |
| *PID1* | 0.170 | 3.398 | 2.022 | 4.48E-02 | 1.00E+00 |
| *DENND2A* | -0.124 | 3.648 | -2.019 | 4.51E-02 | 1.00E+00 |
| *LDLRAD3* | 0.113 | 5.036 | 2.017 | 4.53E-02 | 1.00E+00 |
| *CMSS1* | 0.110 | 4.499 | 2.014 | 4.56E-02 | 1.00E+00 |
| *ANXA5* | 0.096 | 8.985 | 2.006 | 4.65E-02 | 1.00E+00 |
| *LAT2* | 0.127 | 2.171 | 1.985 | 4.88E-02 | 1.00E+00 |
| *VPS11* | -0.072 | 4.975 | -1.965 | 5.11E-02 | 1.00E+00 |
| *CHST2* | 0.177 | 3.182 | 1.959 | 5.17E-02 | 1.00E+00 |
| *KCNJ16* | -0.245 | 3.978 | -1.958 | 5.19E-02 | 1.00E+00 |
| *LINC01099* | -0.280 | 2.381 | -1.955 | 5.23E-02 | 1.00E+00 |
| *CDC20* | -0.228 | 0.548 | -1.949 | 5.29E-02 | 1.00E+00 |
| *RACGAP1* | -0.121 | 3.402 | -1.940 | 5.41E-02 | 1.00E+00 |
| *PAQR5* | 0.158 | 4.345 | 1.927 | 5.56E-02 | 1.00E+00 |
| *CD5* | 0.234 | 1.733 | 1.912 | 5.76E-02 | 1.00E+00 |
| *DIXDC1* | -0.076 | 4.517 | -1.886 | 6.11E-02 | 1.00E+00 |
| *LAMC2* | 0.222 | 8.859 | 1.881 | 6.17E-02 | 1.00E+00 |
| *SERPINB9* | 0.117 | 4.083 | 1.860 | 6.47E-02 | 1.00E+00 |
| *ACP2* | 0.081 | 5.209 | 1.860 | 6.47E-02 | 1.00E+00 |
| *DPT* | 0.272 | 3.144 | 1.855 | 6.53E-02 | 1.00E+00 |
| *ITGA9-AS1* | -0.125 | 0.722 | -1.841 | 6.74E-02 | 1.00E+00 |
| *PTX3* | 0.214 | 1.577 | 1.816 | 7.12E-02 | 1.00E+00 |
| *PTTG1IP* | 0.070 | 8.172 | 1.815 | 7.14E-02 | 1.00E+00 |
| *PDE4B* | 0.167 | 4.618 | 1.813 | 7.16E-02 | 1.00E+00 |
| *CLCF1* | 0.157 | 2.863 | 1.811 | 7.19E-02 | 1.00E+00 |
| *AIFM2* | 0.086 | 4.745 | 1.810 | 7.21E-02 | 1.00E+00 |
| *RP11-680F8.1* | 0.177 | -0.875 | 1.781 | 7.68E-02 | 1.00E+00 |
| *SLC38A5* | 0.186 | 2.825 | 1.756 | 8.09E-02 | 1.00E+00 |
| *RP11-693N9.2* | -0.220 | -0.825 | -1.749 | 8.21E-02 | 1.00E+00 |
| *RCBTB1* | -0.064 | 6.202 | -1.728 | 8.58E-02 | 1.00E+00 |
| *ABI3BP* | 0.145 | 4.237 | 1.699 | 9.11E-02 | 1.00E+00 |
| *DNAJB4* | -0.091 | 5.002 | -1.699 | 9.12E-02 | 1.00E+00 |
| *HSPB6* | 0.227 | 1.464 | 1.694 | 9.22E-02 | 1.00E+00 |
| *SCD* | -0.105 | 9.693 | -1.675 | 9.58E-02 | 1.00E+00 |
| *FAM8A1* | -0.056 | 6.203 | -1.674 | 9.61E-02 | 1.00E+00 |
| *MALL* | 0.195 | 3.482 | 1.661 | 9.87E-02 | 1.00E+00 |
| *SLC35D3* | 0.168 | 1.263 | 1.656 | 9.96E-02 | 1.00E+00 |
| *PIEZO2* | -0.271 | 2.290 | -1.652 | 1.00E-01 | 1.00E+00 |
| *CXCL5* | 0.229 | 4.151 | 1.628 | 1.05E-01 | 1.00E+00 |
| *EPHB2* | 0.127 | 5.309 | 1.619 | 1.07E-01 | 1.00E+00 |
| *ARSJ* | 0.096 | 3.529 | 1.597 | 1.12E-01 | 1.00E+00 |
| *FCN3* | 0.320 | 1.186 | 1.589 | 1.14E-01 | 1.00E+00 |
| *POLD2* | 0.095 | 5.857 | 1.588 | 1.14E-01 | 1.00E+00 |
| *SGOL2* | -0.092 | 2.220 | -1.583 | 1.15E-01 | 1.00E+00 |
| *COMP* | 0.257 | 1.493 | 1.575 | 1.17E-01 | 1.00E+00 |
| *NAV3* | 0.112 | 3.407 | 1.568 | 1.19E-01 | 1.00E+00 |
| *SIRPA* | 0.098 | 5.214 | 1.563 | 1.20E-01 | 1.00E+00 |
| *MYC* | 0.111 | 5.110 | 1.533 | 1.27E-01 | 1.00E+00 |
| *DUSP5* | 0.109 | 6.364 | 1.532 | 1.28E-01 | 1.00E+00 |
| *GRPEL1* | 0.054 | 5.552 | 1.524 | 1.29E-01 | 1.00E+00 |
| *NR2F1-AS1* | -0.177 | 2.145 | -1.519 | 1.31E-01 | 1.00E+00 |
| *NDRG3* | -0.056 | 5.769 | -1.513 | 1.32E-01 | 1.00E+00 |
| *SNRNP48* | -0.040 | 5.414 | -1.510 | 1.33E-01 | 1.00E+00 |
| *NUDT12* | -0.055 | 5.804 | -1.504 | 1.35E-01 | 1.00E+00 |
| *TNFRSF11A* | 0.076 | 5.164 | 1.499 | 1.36E-01 | 1.00E+00 |
| *PTGER4* | 0.095 | 2.621 | 1.494 | 1.37E-01 | 1.00E+00 |
| *MAMLD1* | 0.105 | 4.346 | 1.491 | 1.38E-01 | 1.00E+00 |
| *NECAB2* | 0.140 | 2.528 | 1.489 | 1.38E-01 | 1.00E+00 |
| *RRS1* | 0.105 | 3.715 | 1.484 | 1.40E-01 | 1.00E+00 |
| *CDK1* | -0.200 | 0.711 | -1.479 | 1.41E-01 | 1.00E+00 |
| *AC004381.6* | -0.090 | 1.472 | -1.472 | 1.43E-01 | 1.00E+00 |
| *TPX2* | -0.170 | 1.488 | -1.462 | 1.46E-01 | 1.00E+00 |
| *FST* | 0.134 | 1.201 | 1.460 | 1.46E-01 | 1.00E+00 |
| *S100A10* | 0.141 | 6.846 | 1.460 | 1.46E-01 | 1.00E+00 |
| *SLC34A2* | 0.148 | 5.321 | 1.448 | 1.49E-01 | 1.00E+00 |
| *FBLN5* | 0.111 | 3.715 | 1.440 | 1.52E-01 | 1.00E+00 |
| *BAG1* | 0.060 | 6.125 | 1.438 | 1.52E-01 | 1.00E+00 |
| *ATP4A* | 0.315 | 0.554 | 1.428 | 1.55E-01 | 1.00E+00 |
| *CMTM7* | 0.089 | 3.984 | 1.424 | 1.56E-01 | 1.00E+00 |
| *HEXB* | -0.049 | 7.816 | -1.421 | 1.57E-01 | 1.00E+00 |
| *LYAR* | 0.083 | 3.690 | 1.384 | 1.68E-01 | 1.00E+00 |
| *NCS1* | 0.095 | 4.995 | 1.380 | 1.69E-01 | 1.00E+00 |
| *RRP12* | 0.088 | 5.288 | 1.374 | 1.71E-01 | 1.00E+00 |
| *TRIQK* | -0.063 | 6.105 | -1.368 | 1.73E-01 | 1.00E+00 |
| *HMOX1* | 0.199 | 7.516 | 1.364 | 1.75E-01 | 1.00E+00 |
| *BCL2L1* | 0.065 | 6.853 | 1.362 | 1.75E-01 | 1.00E+00 |
| *INPP1* | 0.039 | 5.308 | 1.354 | 1.78E-01 | 1.00E+00 |
| *PRC1* | -0.123 | 2.174 | -1.332 | 1.85E-01 | 1.00E+00 |
| *SASS6* | -0.056 | 2.359 | -1.322 | 1.88E-01 | 1.00E+00 |
| *COTL1* | 0.084 | 6.048 | 1.300 | 1.95E-01 | 1.00E+00 |
| *FOSL1* | 0.205 | 4.141 | 1.274 | 2.04E-01 | 1.00E+00 |
| *KCNC1* | 0.107 | 2.921 | 1.269 | 2.06E-01 | 1.00E+00 |
| *CBLC* | 0.114 | 2.347 | 1.235 | 2.19E-01 | 1.00E+00 |
| *USP43* | 0.079 | 3.058 | 1.219 | 2.25E-01 | 1.00E+00 |
| *TMEM237* | -0.049 | 3.271 | -1.208 | 2.29E-01 | 1.00E+00 |
| *VSTM2L* | 0.140 | 3.269 | 1.201 | 2.32E-01 | 1.00E+00 |
| *SH3BGRL* | -0.046 | 6.183 | -1.201 | 2.32E-01 | 1.00E+00 |
| *IL22RA1* | 0.149 | 3.662 | 1.189 | 2.36E-01 | 1.00E+00 |
| *HIATL2* | -0.051 | 2.189 | -1.155 | 2.50E-01 | 1.00E+00 |
| *SEPP1* | -0.085 | 4.403 | -1.154 | 2.50E-01 | 1.00E+00 |
| *FJX1* | 0.120 | 2.611 | 1.153 | 2.51E-01 | 1.00E+00 |
| *NUDT7* | -0.071 | 1.511 | -1.151 | 2.51E-01 | 1.00E+00 |
| *SNRPA* | 0.070 | 4.748 | 1.140 | 2.56E-01 | 1.00E+00 |
| *C4orf33* | -0.051 | 3.599 | -1.067 | 2.88E-01 | 1.00E+00 |
| *S100A16* | 0.102 | 6.026 | 1.064 | 2.89E-01 | 1.00E+00 |
| *NOP16* | 0.068 | 3.582 | 1.063 | 2.89E-01 | 1.00E+00 |
| *FBXL20* | -0.053 | 4.461 | -1.054 | 2.93E-01 | 1.00E+00 |
| *KLF12* | -0.054 | 4.276 | -1.045 | 2.98E-01 | 1.00E+00 |
| *SEPT9* | 0.066 | 6.872 | 1.033 | 3.03E-01 | 1.00E+00 |
| *KBTBD3* | -0.053 | 3.819 | -1.022 | 3.08E-01 | 1.00E+00 |
| *ITGA11* | -0.198 | 3.440 | -0.985 | 3.26E-01 | 1.00E+00 |
| *CCDC86* | 0.063 | 4.586 | 0.966 | 3.36E-01 | 1.00E+00 |
| *ELN* | 0.123 | 1.124 | 0.965 | 3.36E-01 | 1.00E+00 |
| *ENTPD6* | 0.043 | 5.961 | 0.952 | 3.42E-01 | 1.00E+00 |
| *RP11-96H19.1* | -0.076 | -0.005 | -0.944 | 3.46E-01 | 1.00E+00 |
| *RRP9* | 0.063 | 3.736 | 0.944 | 3.47E-01 | 1.00E+00 |
| *FAM122C* | -0.049 | 0.444 | -0.941 | 3.48E-01 | 1.00E+00 |
| *MST1R* | 0.110 | 2.730 | 0.940 | 3.49E-01 | 1.00E+00 |
| *BMPR1B* | 0.074 | 2.379 | 0.911 | 3.64E-01 | 1.00E+00 |
| *ADRA2C* | 0.133 | 0.617 | 0.903 | 3.68E-01 | 1.00E+00 |
| *ZNRF1* | 0.049 | 4.099 | 0.836 | 4.04E-01 | 1.00E+00 |
| *SULT2B1* | 0.130 | 0.504 | 0.792 | 4.30E-01 | 1.00E+00 |
| *TUBB4B* | 0.065 | 7.241 | 0.738 | 4.61E-01 | 1.00E+00 |
| *GDPD1* | -0.040 | 2.207 | -0.737 | 4.62E-01 | 1.00E+00 |
| *HMGA1* | 0.088 | 6.826 | 0.720 | 4.72E-01 | 1.00E+00 |
| *GRWD1* | 0.036 | 4.778 | 0.656 | 5.13E-01 | 1.00E+00 |
| *CELP* | 0.109 | -0.925 | 0.557 | 5.78E-01 | 1.00E+00 |
| MARK1* | -0.150061228 | 5.045222 | -4.14114 | 5.49E-05 | 0.043623 |
| REEP1* | 0.211946353 | 3.814101 | 4.123065 | 5.89E-05 | 0.043623 |

**significantly correlated with HbA1c, but not significantly differentially expressed between T2D and non T2D donor islets*
