# Supplementary material for: Islet Gene View—a tool to facilitate islet research
Source: Life Sci Alliance. 2022 Aug 10;5(12):e202201376. doi: 10.26508/lsa.202201376 (PMC9366203; doi:10.26508/lsa.202201376)
Supplement: Supplementary file 4 [file LSA-2022-01376_TableS4.docx]

Supplementary table 4: Top 5 positively or negatively correlated genes for each secretory gene.

| **gene** | **direction** | **symbol** | **rho** | **FDR** | **P_empirical** |
| --- | --- | --- | --- | --- | --- |
| INS | Positive | *TFEB* | 0.94 | 6.92E-90 | 2.10E-05 |
|  |  | *IRF2BP1* | 0.94 | 1.55E-87 | 2.60E-05 |
|  |  | *SNHG19* | 0.94 | 8.00E-86 | 3.50E-05 |
|  |  | *SURF2* | 0.93 | 1.81E-80 | 6.60E-05 |
|  |  | *C15orf59* | 0.93 | 1.86E-80 | 6.60E-05 |
|  | Negative | *HADHA* | -0.88 | 2.29E-61 | 4.00E-06 |
|  |  | *EXT2* | -0.88 | 3.50E-60 | 6.00E-06 |
|  |  | *ERGIC3* | -0.87 | 3.67E-58 | 8.00E-06 |
|  |  | *RP11-544M22.13* | -0.87 | 1.01E-57 | 8.00E-06 |
|  |  | *ACOX1* | -0.87 | 3.12E-57 | 1.00E-05 |
| GCG | Positive | *TUSC3* | 0.80 | 9.01E-41 | 5.02E-03 |
|  |  | *ENPP2* | 0.79 | 6.90E-40 | 5.41E-03 |
|  |  | *GLT1D1* | 0.78 | 1.72E-37 | 6.64E-03 |
|  |  | *SLC38A4* | 0.77 | 2.80E-36 | 7.35E-03 |
|  |  | *CPQ* | 0.77 | 8.26E-36 | 7.64E-03 |
|  | Negative | *ITPKC* | -0.73 | 1.79E-30 | 2.47E-03 |
|  |  | *ABTB2* | -0.72 | 4.14E-29 | 3.07E-03 |
|  |  | *PPARD* | -0.70 | 4.15E-27 | 4.27E-03 |
|  |  | *TEAD4* | -0.70 | 1.06E-26 | 4.55E-03 |
|  |  | *TUBB6* | -0.69 | 3.47E-26 | 4.96E-03 |
| SST | Positive | *CUTA* | 0.77 | 1.31E-35 | 7.93E-03 |
|  |  | *PSD* | 0.76 | 7.47E-35 | 8.44E-03 |
|  |  | *FBXL16* | 0.76 | 1.37E-34 | 8.63E-03 |
|  |  | *PTPRN2* | 0.75 | 1.59E-33 | 9.46E-03 |
|  |  | *LY6H* | 0.75 | 4.50E-33 | 9.86E-03 |
|  | Negative | *CASP4* | -0.72 | 8.16E-29 | 3.34E-03 |
|  |  | *EIF4A1* | -0.70 | 0 | 4.33E-03 |
|  |  | *MRRF* | -0.69 | 2.22E-26 | 4.99E-03 |
|  |  | *EIF4E2* | -0.68 | 1.76E-25 | 5.78E-03 |
|  |  | *RALB* | -0.67 | 2.09E-24 | 6.89E-03 |
| PPY | Positive | *CARTPT* | 0.60 | 8.78E-16 | 4.07E-02 |
|  |  | *KCNG1* | 0.44 | 1.29E-06 | 1.12E-01 |
|  |  | *MAPK13* | 0.41 | 2.01E-05 | 1.31E-01 |
|  |  | *ACOT7* | 0.40 | 2.55E-05 | 1.35E-01 |
|  |  | *ERCC1* | 0.38 | 1.50E-04 | 1.52E-01 |
|  | Negative | *WIPF3* | -0.40 | 4.39E-05 | 9.68E-02 |
|  |  | *RP11-147L13.11* | -0.36 | 2.41E-05 | 1.20E-01 |
|  |  | *KLHL5* | -0.34 | 3.73E-04 | 1.34E-01 |
|  |  | *KCNAB1* | -0.34 | 3.94E-04 | 1.35E-01 |
|  |  | *MAP4K3* | -0.32 | 6.21E-04 | 1.45E-01 |
| IAPP | Positive | *PCSK1* | 0.82 | 0 | 3.71E-03 |
|  |  | *RNF14* | 0.78 | 6.94E-38 | 6.51E-03 |
|  |  | *VDAC3* | 0.78 | 1.93E-37 | 6.78E-03 |
|  |  | *B3GALNT1* | 0.77 | 4.89E-36 | 7.60E-03 |
|  |  | *PRDX3* | 0.77 | 0 | 7.68E-03 |
|  | Negative | *MESDC1* | -0.78 | 1.89E-037 | 7.27E-04 |
|  |  | *PIM3* | -0.76 | 3.84E-35 | 1.10E-03 |
|  |  | *ZNF598* | -0.76 | 1.68E-34 | 1.25E-03 |
|  |  | *AXIN1* | -0.76 | 1.92E-34 | 1.26E-03 |
|  |  | *ZNF503* | -0.76 | 2.56E-34 | 1.29E-03 |
| GHRL | Positive | *CNIH2* | 0.42 | 1.01E-05 | 1.22E-01 |
|  |  | *D4S234E* | 0.39 | 1.27E-04 | 1.46E-01 |
|  |  | *ZSCAN16-AS1* | 0.38 | 1.38E-04 | 1.49E-01 |
|  |  | *RAB3C* | 0.38 | 1.38E-04 | 1.51E-01 |
|  |  | *C6orf57* | 0.38 | 1.38E-04 | 1.52E-01 |
|  | Negative | *PTPN12* | -0.39 | 1.17E-04 | 9.87E-02 |
|  |  | *CYTH1* | -0.39 | 1.27E-04 | 1.03E-01 |
|  |  | *RP11-253E3.3* | -0.36 | 2.97E-04 | 1.16E-01 |
|  |  | *TET3* | -0.36 | 3.69E-04 | 1.19E-01 |
|  |  | *RAPGEF2* | -0.36 | 3.69E-04 | 1.20E-01 |
