# Supplementary material for: Islet Gene View—a tool to facilitate islet research
Source: Life Sci Alliance. 2022 Aug 10;5(12):e202201376. doi: 10.26508/lsa.202201376 (PMC9366203; doi:10.26508/lsa.202201376)
Supplement: Supplementary file 5 [file LSA-2022-01376_TableS5.docx]

Supplementary table 5: Coexpression of differentially expressed genes (T2D vs non-T2D, from supplementary table 2) with INS, GCG, IAPP, SST, GHRL expression. Significant correlations ( Bonferroni p INS_SPP < 3.33E-06) are presented in bold.

| **genes** | **HGNC** | **INS_rho** | **INS_empP** | **INS_SPP** | **GCG_rho** | **GCG_empP** | **GCG_SPP** | **IAPP_rho** | **IAPP_empP** | **IAPP_SPP** | **SST_rho** | **SST_empP** | **SST_SPP** | **PPY_rho** | **PPY_empP** | **PPY_SPP** | **GHRL_rho** | **GHRL_empP** | **GHRL_SPP** |
| --- | --- | --- | --- | --- | --- | --- | --- | --- | --- | --- | --- | --- | --- | --- | --- | --- | --- | --- | --- |
| ENSG00000004776 | *HSPB6* | 0.54 | 6.27E-02 | **1.83E-15** | -0.30 | 1.62E-01 | 2.80E-05 | -0.39 | 9.74E-02 | **2.03E-08** | 0.24 | 2.73E-01 | 1.16E-03 | 0.07 | 4.51E-01 | 3.40E-01 | -0.04 | 4.19E-01 | 5.57E-01 |
| ENSG00000005189 | *AC004381.6* | -0.23 | 2.26E-01 | 1.84E-03 | 0.48 | 9.08E-02 | **4.21E-12** | 0.26 | 2.49E-01 | 3.00E-04 | 0.05 | 4.73E-01 | 4.90E-01 | -0.14 | 3.16E-01 | 6.40E-02 | 0.19 | 3.23E-01 | 1.08E-02 |
| ENSG00000006071 | *ABCC8* | -0.09 | 3.62E-01 | 2.02E-01 | 0.46 | 1.01E-01 | **3.38E-11** | 0.45 | 1.08E-01 | **1.34E-10** | 0.26 | 2.45E-01 | 2.42E-04 | 0.06 | 4.63E-01 | 4.21E-01 | 0.25 | 2.58E-01 | 5.09E-04 |
| ENSG00000006128 | *TAC1* | 0.02 | 5.08E-01 | 7.83E-01 | -0.19 | 2.64E-01 | 1.06E-02 | -0.02 | 4.43E-01 | 7.66E-01 | -0.13 | 3.18E-01 | 6.81E-02 | 0.04 | 4.88E-01 | 6.08E-01 | 0.05 | 4.71E-01 | 4.78E-01 |
| ENSG00000008311 | *AASS* | -0.65 | 9.80E-03 | **6.87E-24** | 0.27 | 2.41E-01 | 1.85E-04 | 0.44 | 1.14E-01 | **3.66E-10** | -0.34 | 1.30E-01 | **1.36E-06** | -0.10 | 3.59E-01 | 1.88E-01 | -0.02 | 4.47E-01 | 8.03E-01 |
| ENSG00000010282 | *HHATL* | 0.21 | 2.97E-01 | 3.67E-03 | 0.19 | 3.15E-01 | 7.83E-03 | 0.29 | 2.24E-01 | 5.88E-05 | 0.39 | 1.43E-01 | **2.73E-08** | 0.05 | 4.75E-01 | 5.06E-01 | 0.26 | 2.51E-01 | 3.38E-04 |
| ENSG00000011465 | *DCN* | 0.06 | 4.64E-01 | 4.29E-01 | -0.10 | 3.51E-01 | 1.57E-01 | -0.18 | 2.67E-01 | 1.17E-02 | -0.16 | 2.90E-01 | 2.78E-02 | 0.03 | 5.02E-01 | 7.29E-01 | -0.11 | 3.49E-01 | 1.50E-01 |
| ENSG00000013619 | *MAMLD1* | 0.54 | 6.00E-02 | **6.94E-16** | -0.35 | 1.27E-01 | **1.01E-06** | -0.54 | 3.21E-02 | **6.73E-16** | 0.27 | 2.38E-01 | 1.52E-04 | 0.01 | 5.15E-01 | 8.44E-01 | -0.12 | 3.37E-01 | 1.12E-01 |
| ENSG00000017483 | *SLC38A5* | -0.27 | 1.86E-01 | 1.71E-04 | -0.46 | 6.54E-02 | **4.90E-11** | -0.06 | 3.95E-01 | 3.83E-01 | -0.52 | 4.02E-02 | **2.19E-14** | 0.02 | 5.04E-01 | 7.49E-01 | -0.17 | 2.78E-01 | 1.82E-02 |
| ENSG00000025039 | *RRAGD* | 0.24 | 2.69E-01 | 9.18E-04 | 0.21 | 3.01E-01 | 4.31E-03 | 0.03 | 4.91E-01 | 6.36E-01 | 0.47 | 9.74E-02 | **1.71E-11** | -0.06 | 3.96E-01 | 3.88E-01 | 0.19 | 3.18E-01 | 8.82E-03 |
| ENSG00000027869 | *SH2D2A* | 0.41 | 1.28E-01 | **3.60E-09** | -0.30 | 1.59E-01 | 2.22E-05 | -0.43 | 8.05E-02 | **1.20E-09** | 0.14 | 3.77E-01 | 6.44E-02 | 0.20 | 3.04E-01 | 4.97E-03 | -0.11 | 3.38E-01 | 1.17E-01 |
| ENSG00000042286 | *AIFM2* | 0.41 | 1.30E-01 | **4.63E-09** | -0.56 | 2.66E-02 | **3.45E-17** | -0.44 | 7.27E-02 | **2.49E-10** | 0.10 | 4.19E-01 | 1.81E-01 | 0.23 | 2.81E-01 | 1.73E-03 | -0.18 | 2.68E-01 | 1.26E-02 |
| ENSG00000047648 | *ARHGAP6* | -0.18 | 2.65E-01 | 1.11E-02 | -0.12 | 3.33E-01 | 1.02E-01 | 0.00 | 4.67E-01 | 9.85E-01 | -0.25 | 2.07E-01 | 6.33E-04 | -0.01 | 4.59E-01 | 9.10E-01 | -0.15 | 2.98E-01 | 3.61E-02 |
| ENSG00000048162 | *NOP16* | 0.51 | 7.38E-02 | **6.09E-14** | -0.65 | 1.03E-02 | **1.59E-23** | -0.47 | 5.77E-02 | **7.09E-12** | 0.05 | 4.68E-01 | 4.56E-01 | 0.25 | 2.60E-01 | 5.64E-04 | -0.14 | 3.07E-01 | 4.85E-02 |
| ENSG00000049540 | *ELN* | 0.65 | 2.59E-02 | **3.01E-24** | -0.56 | 2.91E-02 | **1.38E-16** | -0.69 | 5.52E-03 | **1.22E-27** | 0.20 | 3.06E-01 | 5.30E-03 | 0.17 | 3.40E-01 | 2.01E-02 | -0.16 | 2.93E-01 | 3.10E-02 |
| ENSG00000049860 | *HEXB* | -0.48 | 5.48E-02 | **3.14E-12** | 0.52 | 7.07E-02 | **2.42E-14** | 0.57 | 4.84E-02 | **6.66E-18** | -0.02 | 4.43E-01 | 7.62E-01 | -0.20 | 2.55E-01 | 7.13E-03 | 0.20 | 3.08E-01 | 5.90E-03 |
| ENSG00000050165 | *DKK3* | -0.33 | 1.41E-01 | 4.13E-06 | 0.48 | 8.71E-02 | **1.82E-12** | 0.26 | 2.53E-01 | 3.70E-04 | -0.09 | 3.63E-01 | 2.05E-01 | -0.16 | 2.94E-01 | 3.17E-02 | 0.12 | 3.89E-01 | 8.82E-02 |
| ENSG00000050438 | *SLC4A8* | -0.06 | 3.94E-01 | 3.76E-01 | 0.61 | 3.72E-02 | **1.52E-20** | 0.40 | 1.37E-01 | **1.24E-08** | 0.38 | 1.50E-01 | **6.68E-08** | -0.11 | 3.48E-01 | 1.47E-01 | 0.26 | 2.47E-01 | 2.69E-04 |
| ENSG00000052749 | *RRP12* | 0.74 | 1.14E-02 | **1.96E-33** | -0.56 | 2.80E-02 | **7.51E-17** | -0.59 | 2.06E-02 | **6.56E-19** | 0.39 | 1.47E-01 | **4.69E-08** | 0.19 | 3.20E-01 | 9.44E-03 | -0.10 | 3.60E-01 | 1.94E-01 |
| ENSG00000053108 | *FSTL4* | 0.27 | 2.39E-01 | 1.60E-04 | 0.20 | 3.13E-01 | 7.30E-03 | 0.06 | 4.65E-01 | 4.32E-01 | 0.32 | 1.94E-01 | 5.98E-06 | 0.01 | 5.22E-01 | 9.15E-01 | 0.14 | 3.75E-01 | 6.01E-02 |
| ENSG00000053372 | *MRTO4* | -0.12 | 3.35E-01 | 1.06E-01 | -0.41 | 8.77E-02 | **4.25E-09** | -0.02 | 4.43E-01 | 7.62E-01 | -0.33 | 1.38E-01 | **3.23E-06** | 0.13 | 3.80E-01 | 6.94E-02 | -0.11 | 3.38E-01 | 1.16E-01 |
| ENSG00000058085 | *LAMC2* | -0.13 | 3.20E-01 | 7.21E-02 | -0.55 | 3.04E-02 | **2.91E-16** | -0.17 | 2.77E-01 | 1.78E-02 | -0.40 | 9.58E-02 | **1.59E-08** | 0.02 | 5.06E-01 | 7.64E-01 | -0.20 | 2.49E-01 | 5.48E-03 |
| ENSG00000067798 | *NAV3* | 0.32 | 1.96E-01 | 6.87E-06 | -0.23 | 2.19E-01 | 1.30E-03 | -0.32 | 1.44E-01 | 5.46E-06 | 0.16 | 3.55E-01 | 3.25E-02 | 0.01 | 5.24E-01 | 9.24E-01 | -0.04 | 4.24E-01 | 6.03E-01 |
| ENSG00000069424 | *KCNAB2* | 0.60 | 4.09E-02 | **1.40E-19** | -0.02 | 4.47E-01 | 8.02E-01 | -0.37 | 1.11E-01 | **1.41E-07** | 0.50 | 8.16E-02 | **4.80E-13** | 0.16 | 3.52E-01 | 3.00E-02 | 0.11 | 4.08E-01 | 1.43E-01 |
| ENSG00000069712 | *KIAA1107* | -0.17 | 2.76E-01 | 1.68E-02 | 0.48 | 9.12E-02 | **4.60E-12** | 0.44 | 1.12E-01 | **2.88E-10** | 0.17 | 3.37E-01 | 1.80E-02 | -0.13 | 3.18E-01 | 6.79E-02 | 0.26 | 2.46E-01 | 2.50E-04 |
| ENSG00000070159 | *PTPN3* | -0.26 | 1.99E-01 | 3.91E-04 | 0.21 | 2.97E-01 | 3.60E-03 | 0.40 | 1.39E-01 | **1.64E-08** | 0.01 | 5.15E-01 | 8.46E-01 | -0.03 | 4.30E-01 | 6.50E-01 | 0.10 | 4.14E-01 | 1.64E-01 |
| ENSG00000071073 | *MGAT4A* | -0.27 | 1.83E-01 | 1.34E-04 | 0.47 | 9.73E-02 | **1.67E-11** | 0.54 | 6.22E-02 | **1.51E-15** | 0.04 | 4.89E-01 | 6.17E-01 | -0.13 | 3.19E-01 | 7.00E-02 | 0.26 | 2.54E-01 | 3.94E-04 |
| ENSG00000076513 | *ANKRD13A* | -0.26 | 1.95E-01 | 2.96E-04 | 0.09 | 4.25E-01 | 2.08E-01 | 0.09 | 4.23E-01 | 1.98E-01 | -0.05 | 4.17E-01 | 5.39E-01 | -0.11 | 3.42E-01 | 1.28E-01 | 0.00 | 4.67E-01 | 9.85E-01 |
| ENSG00000077312 | *SNRPA* | 0.47 | 9.40E-02 | **8.45E-12** | -0.54 | 3.24E-02 | **7.78E-16** | -0.53 | 3.52E-02 | **2.75E-15** | 0.10 | 4.13E-01 | 1.61E-01 | 0.12 | 3.91E-01 | 9.29E-02 | -0.15 | 2.96E-01 | 3.42E-02 |
| ENSG00000077942 | *FBLN1* | 0.60 | 3.96E-02 | **6.75E-20** | -0.45 | 6.68E-02 | **6.70E-11** | -0.57 | 2.48E-02 | **1.19E-17** | 0.15 | 3.62E-01 | 4.12E-02 | 0.20 | 3.04E-01 | 4.89E-03 | -0.16 | 2.89E-01 | 2.68E-02 |
| ENSG00000078114 | *NEBL* | -0.04 | 4.26E-01 | 6.15E-01 | 0.51 | 7.61E-02 | **1.15E-13** | 0.28 | 2.31E-01 | 9.71E-05 | 0.31 | 2.07E-01 | 1.69E-05 | -0.10 | 3.54E-01 | 1.70E-01 | 0.18 | 3.34E-01 | 1.62E-02 |
| ENSG00000080608 | *KIAA0020* | -0.27 | 1.83E-01 | 1.34E-04 | -0.30 | 1.65E-01 | 3.58E-05 | 0.09 | 4.31E-01 | 2.33E-01 | -0.35 | 1.24E-01 | **7.01E-07** | -0.02 | 4.41E-01 | 7.43E-01 | -0.09 | 3.66E-01 | 2.22E-01 |
| ENSG00000081181 | *ARG2* | -0.04 | 4.21E-01 | 5.74E-01 | 0.49 | 8.44E-02 | **9.50E-13** | 0.33 | 1.91E-01 | 4.34E-06 | 0.30 | 2.12E-01 | 2.56E-05 | -0.08 | 3.81E-01 | 2.98E-01 | 0.16 | 3.48E-01 | 2.65E-02 |
| ENSG00000082438 | *COBLL1* | -0.51 | 4.43E-02 | **1.02E-13** | 0.07 | 4.51E-01 | 3.39E-01 | 0.34 | 1.81E-01 | **1.84E-06** | -0.28 | 1.76E-01 | 8.01E-05 | -0.11 | 3.39E-01 | 1.19E-01 | -0.08 | 3.74E-01 | 2.56E-01 |
| ENSG00000086730 | *LAT2* | 0.48 | 9.19E-02 | **5.39E-12** | 0.12 | 3.89E-01 | 8.83E-02 | -0.26 | 1.92E-01 | 2.54E-04 | 0.43 | 1.15E-01 | **4.81E-10** | 0.19 | 3.19E-01 | 9.13E-03 | 0.08 | 4.35E-01 | 2.54E-01 |
| ENSG00000088002 | *SULT2B1* | 0.16 | 3.52E-01 | 3.04E-02 | -0.46 | 6.55E-02 | **4.92E-11** | -0.24 | 2.16E-01 | 1.10E-03 | -0.05 | 4.13E-01 | 5.11E-01 | 0.21 | 2.95E-01 | 3.36E-03 | -0.03 | 4.37E-01 | 7.10E-01 |
| ENSG00000088325 | *TPX2* | -0.44 | 7.23E-02 | **2.25E-10** | 0.47 | 9.68E-02 | **1.50E-11** | 0.37 | 1.56E-01 | **1.38E-07** | -0.10 | 3.53E-01 | 1.64E-01 | -0.13 | 3.24E-01 | 8.04E-02 | 0.12 | 3.95E-01 | 1.03E-01 |
| ENSG00000091651 | *ORC6* | 0.09 | 4.27E-01 | 2.15E-01 | 0.00 | 5.28E-01 | 9.66E-01 | -0.18 | 2.66E-01 | 1.13E-02 | 0.15 | 3.64E-01 | 4.39E-02 | 0.09 | 4.23E-01 | 1.98E-01 | -0.01 | 4.52E-01 | 8.43E-01 |
| ENSG00000095752 | *IL11* | 0.34 | 1.81E-01 | **1.74E-06** | -0.42 | 8.38E-02 | **2.16E-09** | -0.47 | 5.85E-02 | **8.84E-12** | -0.04 | 4.27E-01 | 6.26E-01 | 0.06 | 4.59E-01 | 3.94E-01 | -0.24 | 2.14E-01 | 9.77E-04 |
| ENSG00000099194 | *SCD* | -0.14 | 3.16E-01 | 6.30E-02 | 0.56 | 5.41E-02 | **7.43E-17** | 0.35 | 1.75E-01 | **1.02E-06** | 0.20 | 3.11E-01 | 6.73E-03 | -0.15 | 3.01E-01 | 3.98E-02 | 0.18 | 3.33E-01 | 1.58E-02 |
| ENSG00000100292 | *HMOX1* | 0.42 | 1.21E-01 | **1.85E-09** | -0.36 | 1.20E-01 | **5.16E-07** | -0.33 | 1.40E-01 | 4.26E-06 | 0.17 | 3.37E-01 | 1.82E-02 | 0.14 | 3.73E-01 | 5.71E-02 | -0.11 | 3.48E-01 | 1.48E-01 |
| ENSG00000100302 | *RASD2* | 0.45 | 1.05E-01 | **8.57E-11** | -0.43 | 7.84E-02 | **7.83E-10** | -0.51 | 4.18E-02 | **4.17E-14** | 0.11 | 4.02E-01 | 1.22E-01 | 0.15 | 3.64E-01 | 4.40E-02 | -0.15 | 2.98E-01 | 3.67E-02 |
| ENSG00000101079 | *NDRG3* | -0.68 | 6.30E-03 | **8.84E-27** | 0.58 | 4.72E-02 | **3.75E-18** | 0.64 | 2.85E-02 | **3.08E-23** | -0.19 | 2.59E-01 | 8.38E-03 | -0.17 | 2.84E-01 | 2.26E-02 | 0.12 | 3.94E-01 | 1.00E-01 |
| ENSG00000101463 | *SYNDIG1* | 0.44 | 1.10E-01 | **1.84E-10** | -0.01 | 4.60E-01 | 9.23E-01 | -0.33 | 1.39E-01 | 3.47E-06 | 0.36 | 1.67E-01 | **4.62E-07** | 0.08 | 4.38E-01 | 2.70E-01 | 0.03 | 5.02E-01 | 7.30E-01 |
| ENSG00000101938 | *CHRDL1* | 0.18 | 3.29E-01 | 1.36E-02 | -0.31 | 1.57E-01 | 1.84E-05 | -0.34 | 1.33E-01 | **1.80E-06** | -0.17 | 2.83E-01 | 2.18E-02 | -0.01 | 4.56E-01 | 8.81E-01 | -0.15 | 3.02E-01 | 4.11E-02 |
| ENSG00000102010 | *BMX* | 0.14 | 3.71E-01 | 5.38E-02 | -0.30 | 1.66E-01 | 3.79E-05 | -0.16 | 2.87E-01 | 2.47E-02 | -0.06 | 4.04E-01 | 4.44E-01 | 0.07 | 4.48E-01 | 3.26E-01 | -0.07 | 3.90E-01 | 3.52E-01 |
| ENSG00000102802 | *MEDAG* | 0.27 | 2.40E-01 | 1.69E-04 | -0.45 | 6.78E-02 | **8.61E-11** | -0.42 | 8.13E-02 | **1.40E-09** | -0.16 | 2.93E-01 | 3.05E-02 | -0.01 | 4.62E-01 | 9.38E-01 | -0.23 | 2.20E-01 | 1.31E-03 |
| ENSG00000102962 | *CCL22* | -0.23 | 2.24E-01 | 1.65E-03 | -0.18 | 2.68E-01 | 1.24E-02 | 0.01 | 5.17E-01 | 8.63E-01 | -0.28 | 1.76E-01 | 8.31E-05 | 0.00 | 4.65E-01 | 9.65E-01 | -0.04 | 4.26E-01 | 6.13E-01 |
| ENSG00000103154 | *NECAB2* | 0.74 | 1.05E-02 | **1.93E-34** | 0.00 | 5.26E-01 | 9.47E-01 | -0.39 | 9.81E-02 | **2.24E-08** | 0.70 | 1.73E-02 | **1.54E-28** | 0.11 | 4.11E-01 | 1.50E-01 | 0.13 | 3.79E-01 | 6.76E-02 |
| ENSG00000103187 | *COTL1* | 0.50 | 8.14E-02 | **4.61E-13** | -0.33 | 1.42E-01 | 4.72E-06 | -0.38 | 1.09E-01 | **1.10E-07** | 0.31 | 2.03E-01 | 1.29E-05 | 0.16 | 3.46E-01 | 2.45E-02 | -0.02 | 4.49E-01 | 8.16E-01 |
| ENSG00000103647 | *CORO2B* | 0.12 | 3.90E-01 | 9.07E-02 | -0.05 | 4.09E-01 | 4.82E-01 | -0.09 | 3.70E-01 | 2.40E-01 | 0.08 | 4.39E-01 | 2.76E-01 | 0.14 | 3.68E-01 | 4.96E-02 | 0.01 | 5.21E-01 | 9.05E-01 |
| ENSG00000104332 | *SFRP1* | -0.12 | 3.31E-01 | 9.61E-02 | -0.11 | 3.43E-01 | 1.30E-01 | -0.12 | 3.36E-01 | 1.11E-01 | -0.28 | 1.80E-01 | 1.10E-04 | -0.04 | 4.18E-01 | 5.46E-01 | -0.12 | 3.32E-01 | 9.91E-02 |
| ENSG00000105447 | *GRWD1* | 0.68 | 2.08E-02 | **1.65E-26** | -0.51 | 4.40E-02 | **9.40E-14** | -0.53 | 3.55E-02 | **3.18E-15** | 0.34 | 1.83E-01 | **2.23E-06** | 0.31 | 2.02E-01 | 1.16E-05 | -0.04 | 4.22E-01 | 5.83E-01 |
| ENSG00000105664 | *COMP* | 0.57 | 4.97E-02 | **1.17E-17** | -0.25 | 2.07E-01 | 6.28E-04 | -0.52 | 3.93E-02 | **1.60E-14** | 0.24 | 2.71E-01 | 1.05E-03 | 0.11 | 4.04E-01 | 1.28E-01 | -0.09 | 3.67E-01 | 2.23E-01 |
| ENSG00000105675 | *ATP4A* | 0.18 | 3.27E-01 | 1.24E-02 | -0.54 | 3.33E-02 | **1.16E-15** | -0.36 | 1.17E-01 | **3.20E-07** | -0.12 | 3.36E-01 | 1.10E-01 | 0.14 | 3.68E-01 | 4.97E-02 | -0.12 | 3.36E-01 | 1.09E-01 |
| ENSG00000106483 | *SFRP4* | 0.37 | 1.59E-01 | **1.80E-07** | -0.38 | 1.09E-01 | **1.08E-07** | -0.46 | 6.15E-02 | **1.88E-11** | 0.00 | 4.66E-01 | 9.79E-01 | 0.06 | 4.65E-01 | 4.31E-01 | -0.21 | 2.43E-01 | 4.31E-03 |
| ENSG00000106628 | *POLD2* | 0.41 | 1.28E-01 | **3.67E-09** | -0.32 | 1.44E-01 | 5.59E-06 | -0.27 | 1.86E-01 | 1.66E-04 | 0.20 | 3.12E-01 | 6.94E-03 | 0.32 | 1.94E-01 | 5.82E-06 | -0.06 | 4.02E-01 | 4.28E-01 |
| ENSG00000107130 | *NCS1* | 0.65 | 2.71E-02 | **9.05E-24** | -0.51 | 4.17E-02 | **4.03E-14** | -0.57 | 2.47E-02 | **1.09E-17** | 0.26 | 2.46E-01 | 2.44E-04 | 0.19 | 3.17E-01 | 8.47E-03 | -0.09 | 3.65E-01 | 2.17E-01 |
| ENSG00000107262 | *BAG1* | 0.35 | 1.73E-01 | **7.97E-07** | -0.35 | 1.27E-01 | **9.47E-07** | -0.26 | 1.92E-01 | 2.55E-04 | 0.25 | 2.56E-01 | 4.60E-04 | 0.23 | 2.74E-01 | 1.21E-03 | 0.09 | 4.24E-01 | 2.02E-01 |
| ENSG00000107317 | *PTGDS* | 0.77 | 7.80E-03 | **6.95E-38** | -0.49 | 5.06E-02 | **8.64E-13** | -0.66 | 8.48E-03 | **6.88E-25** | 0.29 | 2.19E-01 | 4.24E-05 | 0.24 | 2.72E-01 | 1.11E-03 | -0.11 | 3.47E-01 | 1.44E-01 |
| ENSG00000108187 | *PBLD* | -0.61 | 1.61E-02 | **1.36E-20** | 0.38 | 1.51E-01 | **7.09E-08** | 0.49 | 8.38E-02 | **8.05E-13** | -0.31 | 1.53E-01 | 1.26E-05 | -0.19 | 2.63E-01 | 1.01E-02 | 0.04 | 4.85E-01 | 5.83E-01 |
| ENSG00000108306 | *FBXL20* | -0.62 | 1.47E-02 | **3.57E-21** | 0.28 | 2.29E-01 | 8.39E-05 | 0.37 | 1.55E-01 | **1.16E-07** | -0.35 | 1.23E-01 | **6.27E-07** | -0.15 | 3.04E-01 | 4.37E-02 | 0.00 | 4.66E-01 | 9.72E-01 |
| ENSG00000108984 | *MAP2K6* | -0.34 | 1.34E-01 | **1.98E-06** | 0.46 | 1.02E-01 | **4.80E-11** | 0.57 | 4.98E-02 | **1.21E-17** | -0.07 | 3.90E-01 | 3.51E-01 | 0.01 | 5.15E-01 | 8.50E-01 | 0.22 | 2.91E-01 | 2.70E-03 |
| ENSG00000109519 | *GRPEL1* | -0.24 | 2.09E-01 | 7.06E-04 | -0.24 | 2.15E-01 | 1.04E-03 | 0.01 | 5.20E-01 | 8.90E-01 | -0.32 | 1.47E-01 | 7.66E-06 | -0.04 | 4.17E-01 | 5.45E-01 | -0.09 | 3.62E-01 | 2.00E-01 |
| ENSG00000109610 | *SOD3* | 0.83 | 3.18E-03 | **3.14E-48** | -0.48 | 5.38E-02 | **2.35E-12** | -0.68 | 6.04E-03 | **4.79E-27** | 0.43 | 1.17E-01 | **6.36E-10** | 0.19 | 3.22E-01 | 1.04E-02 | -0.16 | 2.95E-01 | 3.27E-02 |
| ENSG00000110104 | *CCDC86* | 0.78 | 6.44E-03 | **3.76E-40** | -0.33 | 1.39E-01 | 3.43E-06 | -0.55 | 3.16E-02 | **5.24E-16** | 0.54 | 6.07E-02 | **9.06E-16** | 0.20 | 3.11E-01 | 6.54E-03 | 0.03 | 5.02E-01 | 7.29E-01 |
| ENSG00000110442 | *COMMD9* | -0.33 | 1.37E-01 | **2.79E-06** | 0.37 | 1.62E-01 | **2.59E-07** | 0.49 | 8.32E-02 | **7.13E-13** | -0.01 | 4.52E-01 | 8.49E-01 | 0.07 | 4.52E-01 | 3.51E-01 | 0.22 | 2.87E-01 | 2.24E-03 |
| ENSG00000110448 | *CD5* | 0.23 | 2.81E-01 | 1.74E-03 | 0.09 | 4.29E-01 | 2.27E-01 | 0.05 | 4.71E-01 | 4.77E-01 | 0.31 | 2.04E-01 | 1.39E-05 | 0.10 | 4.20E-01 | 1.86E-01 | 0.19 | 3.15E-01 | 7.95E-03 |
| ENSG00000110680 | *CALCA* | 0.47 | 9.63E-02 | **1.35E-11** | -0.06 | 4.00E-01 | 4.18E-01 | -0.37 | 1.13E-01 | **1.98E-07** | 0.31 | 2.02E-01 | 1.16E-05 | 0.04 | 4.85E-01 | 5.88E-01 | -0.08 | 3.83E-01 | 3.06E-01 |
| ENSG00000110693 | *SOX6* | -0.55 | 2.98E-02 | **2.06E-16** | 0.54 | 6.36E-02 | **2.49E-15** | 0.52 | 7.24E-02 | **3.97E-14** | -0.18 | 2.72E-01 | 1.43E-02 | -0.19 | 2.62E-01 | 9.79E-03 | 0.08 | 4.40E-01 | 2.79E-01 |
| ENSG00000111644 | *ACRBP* | 0.05 | 4.79E-01 | 5.37E-01 | 0.42 | 1.25E-01 | **2.15E-09** | 0.28 | 2.34E-01 | 1.17E-04 | 0.32 | 1.98E-01 | 8.35E-06 | -0.06 | 4.01E-01 | 4.23E-01 | 0.23 | 2.79E-01 | 1.53E-03 |
| ENSG00000112164 | *GLP1R* | -0.22 | 2.35E-01 | 2.85E-03 | 0.49 | 8.43E-02 | **9.17E-13** | 0.60 | 3.92E-02 | **5.05E-20** | 0.21 | 2.97E-01 | 3.60E-03 | -0.03 | 4.33E-01 | 6.73E-01 | 0.27 | 2.40E-01 | 1.72E-04 |
| ENSG00000112562 | *SMOC2* | 0.35 | 1.75E-01 | **1.03E-06** | -0.39 | 1.03E-01 | **4.61E-08** | -0.41 | 8.64E-02 | **3.44E-09** | -0.01 | 4.57E-01 | 8.90E-01 | 0.01 | 5.21E-01 | 9.06E-01 | -0.14 | 3.13E-01 | 5.91E-02 |
| ENSG00000112874 | *NUDT12* | -0.51 | 4.20E-02 | **4.51E-14** | 0.54 | 6.04E-02 | **8.31E-16** | 0.65 | 2.59E-02 | **2.95E-24** | -0.16 | 2.90E-01 | 2.83E-02 | -0.20 | 2.46E-01 | 4.81E-03 | 0.19 | 3.16E-01 | 8.15E-03 |
| ENSG00000112936 | *C7* | 0.28 | 2.36E-01 | 1.30E-04 | -0.34 | 1.32E-01 | **1.72E-06** | -0.34 | 1.29E-01 | **1.28E-06** | -0.06 | 3.95E-01 | 3.80E-01 | 0.02 | 5.04E-01 | 7.46E-01 | -0.17 | 2.83E-01 | 2.20E-02 |
| ENSG00000113248 | *PCDHB15* | 0.20 | 3.09E-01 | 6.22E-03 | 0.18 | 3.27E-01 | 1.26E-02 | -0.05 | 4.07E-01 | 4.63E-01 | 0.23 | 2.80E-01 | 1.66E-03 | -0.01 | 4.57E-01 | 8.94E-01 | 0.01 | 5.21E-01 | 8.99E-01 |
| ENSG00000113657 | *DPYSL3* | 0.22 | 2.87E-01 | 2.28E-03 | 0.09 | 4.24E-01 | 2.01E-01 | -0.23 | 2.25E-01 | 1.78E-03 | 0.19 | 3.20E-01 | 9.41E-03 | 0.12 | 3.94E-01 | 9.96E-02 | -0.04 | 4.20E-01 | 5.65E-01 |
| ENSG00000114573 | *ATP6V1A* | -0.54 | 3.23E-02 | **7.63E-16** | 0.47 | 9.33E-02 | **7.22E-12** | 0.67 | 2.19E-02 | **5.42E-26** | -0.15 | 3.00E-01 | 3.95E-02 | -0.19 | 2.56E-01 | 7.58E-03 | 0.13 | 3.78E-01 | 6.63E-02 |
| ENSG00000114767 | *RRP9* | 0.54 | 6.06E-02 | **8.64E-16** | -0.62 | 1.52E-02 | **5.62E-21** | -0.43 | 7.82E-02 | **7.57E-10** | 0.17 | 3.37E-01 | 1.82E-02 | 0.33 | 1.90E-01 | 4.09E-06 | -0.03 | 4.33E-01 | 6.73E-01 |
| ENSG00000115112 | *TFCP2L1* | 0.18 | 3.26E-01 | 1.22E-02 | 0.21 | 3.01E-01 | 4.22E-03 | 0.17 | 3.36E-01 | 1.74E-02 | 0.40 | 1.40E-01 | **1.84E-08** | -0.03 | 4.36E-01 | 6.98E-01 | 0.17 | 3.41E-01 | 2.06E-02 |
| ENSG00000115252 | *PDE1A* | -0.35 | 1.29E-01 | **1.24E-06** | 0.25 | 2.58E-01 | 4.96E-04 | 0.29 | 2.21E-01 | 4.90E-05 | -0.27 | 1.88E-01 | 1.94E-04 | -0.01 | 4.62E-01 | 9.42E-01 | 0.02 | 5.14E-01 | 8.33E-01 |
| ENSG00000115590 | *IL1R2* | 0.17 | 3.36E-01 | 1.72E-02 | -0.48 | 5.31E-02 | **1.89E-12** | -0.28 | 1.76E-01 | 8.08E-05 | -0.16 | 2.95E-01 | 3.34E-02 | 0.10 | 4.13E-01 | 1.58E-01 | -0.12 | 3.30E-01 | 9.50E-02 |
| ENSG00000115594 | *IL1R1* | -0.17 | 2.81E-01 | 2.00E-02 | -0.30 | 1.65E-01 | 3.51E-05 | -0.22 | 2.30E-01 | 2.22E-03 | -0.41 | 8.98E-02 | **6.01E-09** | -0.15 | 3.02E-01 | 4.16E-02 | -0.32 | 1.50E-01 | 9.61E-06 |
| ENSG00000115602 | *IL1RL1* | 0.09 | 4.30E-01 | 2.28E-01 | -0.34 | 1.34E-01 | **2.02E-06** | -0.17 | 2.83E-01 | 2.18E-02 | -0.17 | 2.78E-01 | 1.82E-02 | 0.03 | 4.94E-01 | 6.60E-01 | -0.09 | 3.68E-01 | 2.30E-01 |
| ENSG00000115604 | *IL18R1* | 0.30 | 2.10E-01 | 2.16E-05 | -0.34 | 1.36E-01 | **2.47E-06** | -0.44 | 7.54E-02 | **4.29E-10** | -0.02 | 4.42E-01 | 7.57E-01 | -0.02 | 4.43E-01 | 7.66E-01 | -0.18 | 2.73E-01 | 1.51E-02 |
| ENSG00000116329 | *OPRD1* | 0.25 | 2.64E-01 | 7.02E-04 | 0.22 | 2.89E-01 | 2.49E-03 | 0.08 | 4.40E-01 | 2.82E-01 | 0.42 | 1.24E-01 | **1.93E-09** | -0.03 | 4.39E-01 | 7.30E-01 | 0.14 | 3.77E-01 | 6.40E-02 |
| ENSG00000117153 | *KLHL12* | -0.51 | 4.21E-02 | **4.72E-14** | 0.49 | 8.35E-02 | **7.57E-13** | 0.54 | 5.98E-02 | **6.66E-16** | -0.14 | 3.09E-01 | 5.14E-02 | -0.19 | 2.62E-01 | 9.57E-03 | 0.16 | 3.51E-01 | 2.93E-02 |
| ENSG00000117399 | *CDC20* | -0.23 | 2.23E-01 | 1.55E-03 | 0.31 | 2.08E-01 | 1.86E-05 | 0.22 | 2.85E-01 | 2.10E-03 | -0.01 | 4.57E-01 | 8.96E-01 | 0.01 | 5.18E-01 | 8.70E-01 | 0.07 | 4.50E-01 | 3.36E-01 |
| ENSG00000117899 | *MESDC2* | -0.34 | 1.35E-01 | **2.39E-06** | -0.10 | 3.54E-01 | 1.71E-01 | 0.10 | 4.14E-01 | 1.63E-01 | -0.29 | 1.73E-01 | 6.61E-05 | -0.06 | 4.00E-01 | 4.15E-01 | -0.14 | 3.07E-01 | 4.83E-02 |
| ENSG00000118922 | *KLF12* | -0.45 | 6.97E-02 | **1.32E-10** | 0.64 | 2.94E-02 | **6.36E-23** | 0.53 | 6.72E-02 | **8.21E-15** | 0.00 | 4.68E-01 | 9.94E-01 | -0.17 | 2.83E-01 | 2.20E-02 | 0.11 | 4.00E-01 | 1.17E-01 |
| ENSG00000119508 | *NR4A3* | -0.12 | 3.30E-01 | 9.30E-02 | -0.11 | 3.42E-01 | 1.29E-01 | -0.09 | 3.66E-01 | 2.19E-01 | -0.17 | 2.75E-01 | 1.64E-02 | 0.01 | 5.23E-01 | 9.18E-01 | -0.18 | 2.71E-01 | 1.37E-02 |
| ENSG00000119927 | *GPAM* | -0.52 | 3.83E-02 | **1.07E-14** | 0.31 | 2.08E-01 | 1.79E-05 | 0.51 | 7.54E-02 | **9.44E-14** | -0.20 | 2.46E-01 | 4.95E-03 | -0.09 | 3.69E-01 | 2.32E-01 | 0.08 | 4.35E-01 | 2.53E-01 |
| ENSG00000121351 | *IAPP* | -0.58 | 2.22E-02 | **0.00E+00** | 0.46 | 1.03E-01 | **6.80E-11** | 1.00 | 1.00E-06 | **0.00E+00** | -0.16 | 2.88E-01 | 2.60E-02 | -0.07 | 3.84E-01 | 3.15E-01 | 0.26 | 2.46E-01 | 2.45E-04 |
| ENSG00000121898 | *CPXM2* | -0.03 | 4.29E-01 | 6.45E-01 | 0.22 | 2.87E-01 | 2.29E-03 | -0.09 | 3.70E-01 | 2.38E-01 | 0.00 | 4.67E-01 | 9.89E-01 | 0.01 | 5.15E-01 | 8.43E-01 | -0.06 | 4.01E-01 | 4.22E-01 |
| ENSG00000122862 | *SRGN* | -0.27 | 1.88E-01 | 1.99E-04 | 0.02 | 5.11E-01 | 8.07E-01 | 0.11 | 4.03E-01 | 1.27E-01 | -0.35 | 1.24E-01 | **7.16E-07** | -0.08 | 3.78E-01 | 2.80E-01 | -0.13 | 3.22E-01 | 7.55E-02 |
| ENSG00000124151 | *NCOA3* | -0.55 | 2.97E-02 | **1.98E-16** | 0.35 | 1.71E-01 | **6.64E-07** | 0.51 | 7.33E-02 | **5.26E-14** | -0.20 | 2.47E-01 | 5.03E-03 | -0.18 | 2.68E-01 | 1.26E-02 | 0.05 | 4.75E-01 | 5.03E-01 |
| ENSG00000124762 | *CDKN1A* | 0.31 | 2.08E-01 | 2.04E-05 | -0.39 | 9.79E-02 | **2.90E-08** | -0.27 | 1.88E-01 | 1.98E-04 | 0.05 | 4.70E-01 | 4.71E-01 | 0.29 | 2.24E-01 | 6.49E-05 | -0.13 | 3.27E-01 | 8.66E-02 |
| ENSG00000126218 | *F10* | 0.63 | 3.13E-02 | **2.81E-22** | 0.20 | 3.05E-01 | 5.13E-03 | -0.35 | 1.28E-01 | **1.12E-06** | 0.56 | 5.49E-02 | **1.03E-16** | 0.06 | 4.66E-01 | 4.42E-01 | 0.13 | 3.86E-01 | 8.28E-02 |
| ENSG00000126733 | *DACH2* | -0.21 | 2.44E-01 | 4.47E-03 | 0.44 | 1.12E-01 | **2.82E-10** | 0.59 | 4.18E-02 | **2.31E-19** | 0.17 | 3.43E-01 | 2.25E-02 | -0.09 | 3.71E-01 | 2.42E-01 | 0.23 | 2.78E-01 | 1.50E-03 |
| ENSG00000127325 | *BEST3* | -0.27 | 1.90E-01 | 2.13E-04 | 0.49 | 8.61E-02 | **1.44E-12** | 0.66 | 2.35E-02 | **3.13E-25** | 0.03 | 4.96E-01 | 6.79E-01 | -0.08 | 3.73E-01 | 2.51E-01 | 0.24 | 2.65E-01 | 7.44E-04 |
| ENSG00000127328 | *RAB3IP* | 0.32 | 1.98E-01 | 8.15E-06 | -0.03 | 4.35E-01 | 6.94E-01 | -0.15 | 2.98E-01 | 3.67E-02 | 0.34 | 1.82E-01 | **2.04E-06** | 0.02 | 5.08E-01 | 7.78E-01 | 0.19 | 3.19E-01 | 9.16E-03 |
| ENSG00000128342 | *LIF* | 0.33 | 1.86E-01 | **2.96E-06** | -0.51 | 4.44E-02 | **1.07E-13** | -0.56 | 2.78E-02 | **6.60E-17** | -0.09 | 3.70E-01 | 2.38E-01 | 0.08 | 4.41E-01 | 2.85E-01 | -0.29 | 1.67E-01 | 4.26E-05 |
| ENSG00000128683 | *GAD1* | 0.37 | 1.57E-01 | **1.42E-07** | -0.01 | 4.55E-01 | 8.71E-01 | -0.32 | 1.47E-01 | 7.76E-06 | 0.16 | 3.52E-01 | 2.99E-02 | -0.02 | 4.41E-01 | 7.49E-01 | -0.03 | 4.31E-01 | 6.57E-01 |
| ENSG00000129159 | *KCNC1* | 0.41 | 1.29E-01 | **4.06E-09** | 0.25 | 2.60E-01 | 5.69E-04 | -0.20 | 2.54E-01 | 6.84E-03 | 0.50 | 7.93E-02 | **2.68E-13** | 0.05 | 4.73E-01 | 4.90E-01 | 0.08 | 4.37E-01 | 2.64E-01 |
| ENSG00000129534 | *MIS18BP1* | 0.25 | 2.56E-01 | 4.52E-04 | 0.17 | 3.35E-01 | 1.69E-02 | -0.10 | 3.53E-01 | 1.65E-01 | 0.32 | 1.94E-01 | 6.13E-06 | 0.03 | 5.01E-01 | 7.20E-01 | 0.04 | 4.87E-01 | 6.01E-01 |
| ENSG00000130643 | *CALY* | 0.87 | 1.05E-03 | **2.09E-59** | -0.12 | 3.35E-01 | 1.07E-01 | -0.55 | 2.95E-02 | **1.70E-16** | 0.64 | 2.79E-02 | **1.78E-23** | 0.21 | 2.96E-01 | 3.42E-03 | 0.06 | 4.67E-01 | 4.48E-01 |
| ENSG00000130830 | *MPP1* | -0.42 | 8.47E-02 | **2.54E-09** | 0.42 | 1.27E-01 | **3.15E-09** | 0.63 | 3.15E-02 | **3.15E-22** | -0.08 | 3.72E-01 | 2.48E-01 | -0.07 | 3.86E-01 | 3.23E-01 | 0.14 | 3.70E-01 | 5.22E-02 |
| ENSG00000131171 | *SH3BGRL* | -0.47 | 5.85E-02 | **8.88E-12** | 0.74 | 1.11E-02 | **8.33E-34** | 0.61 | 3.74E-02 | **1.73E-20** | -0.05 | 4.07E-01 | 4.61E-01 | -0.23 | 2.25E-01 | 1.77E-03 | 0.23 | 2.79E-01 | 1.58E-03 |
| ENSG00000132386 | *SERPINF1* | 0.47 | 9.52E-02 | **1.09E-11** | -0.43 | 7.73E-02 | **6.32E-10** | -0.54 | 3.36E-02 | **1.40E-15** | 0.04 | 4.83E-01 | 5.72E-01 | 0.07 | 4.48E-01 | 3.23E-01 | -0.20 | 2.52E-01 | 6.28E-03 |
| ENSG00000132640 | *BTBD3* | -0.13 | 3.25E-01 | 8.24E-02 | 0.55 | 5.63E-02 | **1.83E-16** | 0.39 | 1.42E-01 | **2.47E-08** | 0.21 | 2.96E-01 | 3.42E-03 | -0.17 | 2.84E-01 | 2.24E-02 | 0.24 | 2.69E-01 | 9.34E-04 |
| ENSG00000132821 | *VSTM2L* | 0.85 | 1.67E-03 | **8.10E-55** | -0.24 | 2.17E-01 | 1.11E-03 | -0.60 | 1.76E-02 | **5.57E-20** | 0.59 | 4.39E-02 | **7.07E-19** | 0.19 | 3.20E-01 | 9.61E-03 | 0.01 | 5.14E-01 | 8.41E-01 |
| ENSG00000132840 | *BHMT2* | -0.03 | 4.36E-01 | 7.06E-01 | 0.22 | 2.93E-01 | 3.00E-03 | 0.09 | 4.31E-01 | 2.33E-01 | 0.00 | 5.27E-01 | 9.60E-01 | 0.02 | 5.09E-01 | 7.90E-01 | -0.03 | 4.38E-01 | 7.24E-01 |
| ENSG00000133103 | *COG6* | -0.52 | 3.83E-02 | **1.05E-14** | 0.54 | 6.21E-02 | **1.46E-15** | 0.66 | 2.38E-02 | **4.10E-25** | -0.12 | 3.34E-01 | 1.05E-01 | -0.08 | 3.72E-01 | 2.50E-01 | 0.17 | 3.35E-01 | 1.65E-02 |
| ENSG00000133216 | *EPHB2* | 0.00 | 4.67E-01 | 9.86E-01 | -0.61 | 1.58E-02 | **1.04E-20** | -0.27 | 1.87E-01 | 1.81E-04 | -0.30 | 1.62E-01 | 2.66E-05 | 0.15 | 3.60E-01 | 3.83E-02 | -0.25 | 2.03E-01 | 4.89E-04 |
| ENSG00000133794 | *ARNTL* | -0.33 | 1.42E-01 | 4.49E-06 | 0.27 | 2.39E-01 | 1.65E-04 | 0.21 | 2.98E-01 | 3.82E-03 | -0.15 | 2.96E-01 | 3.43E-02 | -0.17 | 2.81E-01 | 2.05E-02 | -0.07 | 3.86E-01 | 3.25E-01 |
| ENSG00000134121 | *CHL1* | -0.13 | 3.22E-01 | 7.48E-02 | 0.41 | 1.32E-01 | **6.20E-09** | 0.45 | 1.07E-01 | **1.09E-10** | 0.21 | 3.01E-01 | 4.24E-03 | -0.13 | 3.26E-01 | 8.54E-02 | 0.26 | 2.46E-01 | 2.44E-04 |
| ENSG00000134278 | *SPIRE1* | 0.14 | 3.69E-01 | 5.13E-02 | 0.17 | 3.43E-01 | 2.23E-02 | -0.08 | 3.76E-01 | 2.68E-01 | 0.26 | 2.45E-01 | 2.42E-04 | -0.12 | 3.36E-01 | 1.09E-01 | -0.02 | 4.40E-01 | 7.35E-01 |
| ENSG00000134363 | *FST* | -0.38 | 1.06E-01 | **6.86E-08** | -0.24 | 2.12E-01 | 8.65E-04 | 0.06 | 4.59E-01 | 3.97E-01 | -0.37 | 1.12E-01 | **1.59E-07** | 0.02 | 5.05E-01 | 7.56E-01 | -0.14 | 3.10E-01 | 5.36E-02 |
| ENSG00000134575 | *ACP2* | 0.28 | 2.33E-01 | 1.13E-04 | -0.16 | 2.87E-01 | 2.49E-02 | -0.19 | 2.63E-01 | 9.92E-03 | 0.24 | 2.72E-01 | 1.06E-03 | 0.19 | 3.16E-01 | 8.00E-03 | -0.03 | 4.33E-01 | 6.73E-01 |
| ENSG00000134817 | *APLNR* | -0.18 | 2.71E-01 | 1.37E-02 | -0.16 | 2.92E-01 | 3.02E-02 | 0.04 | 4.82E-01 | 5.64E-01 | -0.27 | 1.89E-01 | 2.04E-04 | 0.02 | 5.03E-01 | 7.42E-01 | -0.05 | 4.14E-01 | 5.18E-01 |
| ENSG00000134853 | *PDGFRA* | -0.25 | 2.07E-01 | 6.26E-04 | 0.03 | 4.92E-01 | 6.43E-01 | -0.02 | 4.44E-01 | 7.73E-01 | -0.28 | 1.80E-01 | 1.07E-04 | -0.08 | 3.77E-01 | 2.74E-01 | -0.14 | 3.08E-01 | 5.00E-02 |
| ENSG00000134982 | *APC* | -0.10 | 3.54E-01 | 1.68E-01 | 0.35 | 1.74E-01 | **9.16E-07** | 0.28 | 2.28E-01 | 7.92E-05 | 0.20 | 3.13E-01 | 7.14E-03 | -0.12 | 3.30E-01 | 9.32E-02 | 0.12 | 3.92E-01 | 9.50E-02 |
| ENSG00000135318 | *NT5E* | -0.07 | 3.91E-01 | 3.59E-01 | -0.12 | 3.29E-01 | 9.18E-02 | -0.16 | 2.92E-01 | 2.98E-02 | -0.21 | 2.38E-01 | 3.34E-03 | -0.02 | 4.42E-01 | 7.60E-01 | -0.15 | 3.05E-01 | 4.58E-02 |
| ENSG00000135447 | *PPP1R1A* | 0.12 | 3.90E-01 | 9.04E-02 | 0.45 | 1.07E-01 | **1.16E-10** | 0.45 | 1.06E-01 | **8.82E-11** | 0.51 | 7.51E-02 | **8.74E-14** | 0.06 | 4.66E-01 | 4.44E-01 | 0.35 | 1.73E-01 | **7.85E-07** |
| ENSG00000135919 | *SERPINE2* | -0.06 | 3.98E-01 | 4.01E-01 | 0.16 | 3.50E-01 | 2.81E-02 | -0.02 | 4.42E-01 | 7.52E-01 | -0.05 | 4.16E-01 | 5.34E-01 | -0.06 | 3.96E-01 | 3.90E-01 | 0.01 | 5.25E-01 | 9.39E-01 |
| ENSG00000136108 | *CKAP2* | -0.19 | 2.59E-01 | 8.60E-03 | 0.53 | 6.40E-02 | **2.87E-15** | 0.30 | 2.13E-01 | 2.66E-05 | 0.11 | 4.06E-01 | 1.36E-01 | -0.11 | 3.42E-01 | 1.29E-01 | 0.20 | 3.08E-01 | 5.96E-03 |
| ENSG00000136111 | *TBC1D4* | 0.13 | 3.88E-01 | 8.53E-02 | 0.13 | 3.80E-01 | 6.90E-02 | 0.18 | 3.32E-01 | 1.52E-02 | 0.37 | 1.55E-01 | **1.22E-07** | -0.01 | 4.59E-01 | 9.10E-01 | 0.16 | 3.45E-01 | 2.40E-02 |
| ENSG00000136144 | *RCBTB1* | -0.14 | 3.06E-01 | 4.76E-02 | 0.52 | 6.86E-02 | **1.28E-14** | 0.33 | 1.91E-01 | 4.66E-06 | 0.12 | 3.89E-01 | 8.87E-02 | -0.19 | 2.63E-01 | 9.99E-03 | 0.19 | 3.23E-01 | 1.07E-02 |
| ENSG00000136160 | *EDNRB* | 0.23 | 2.82E-01 | 1.85E-03 | -0.25 | 2.08E-01 | 6.78E-04 | -0.28 | 1.81E-01 | 1.19E-04 | 0.01 | 5.18E-01 | 8.76E-01 | 0.09 | 4.29E-01 | 2.26E-01 | -0.13 | 3.18E-01 | 6.83E-02 |
| ENSG00000136244 | *IL6* | -0.03 | 4.31E-01 | 6.54E-01 | -0.41 | 8.85E-02 | **4.84E-09** | -0.27 | 1.90E-01 | 2.16E-04 | -0.37 | 1.15E-01 | **2.43E-07** | -0.01 | 4.52E-01 | 8.50E-01 | -0.25 | 2.06E-01 | 6.15E-04 |
| ENSG00000136943 | *CTSV* | -0.16 | 2.89E-01 | 2.66E-02 | 0.07 | 4.53E-01 | 3.54E-01 | 0.34 | 1.80E-01 | **1.60E-06** | 0.01 | 5.25E-01 | 9.38E-01 | -0.06 | 4.03E-01 | 4.38E-01 | 0.13 | 3.82E-01 | 7.41E-02 |
| ENSG00000136997 | *MYC* | 0.18 | 3.30E-01 | 1.38E-02 | -0.66 | 8.62E-03 | **9.06E-25** | -0.45 | 6.78E-02 | **8.58E-11** | -0.18 | 2.68E-01 | 1.22E-02 | 0.12 | 3.93E-01 | 9.87E-02 | -0.26 | 1.99E-01 | 3.85E-04 |
| ENSG00000137033 | *IL33* | -0.19 | 2.57E-01 | 7.82E-03 | -0.18 | 2.72E-01 | 1.43E-02 | 0.00 | 4.68E-01 | 9.94E-01 | -0.34 | 1.36E-01 | **2.56E-06** | 0.02 | 5.13E-01 | 8.27E-01 | -0.12 | 3.35E-01 | 1.06E-01 |
| ENSG00000137309 | *HMGA1* | 0.68 | 2.09E-02 | **1.68E-26** | -0.66 | 8.83E-03 | **1.32E-24** | -0.64 | 1.12E-02 | **5.54E-23** | 0.23 | 2.83E-01 | 1.85E-03 | 0.24 | 2.65E-01 | 7.30E-04 | -0.12 | 3.31E-01 | 9.59E-02 |
| ENSG00000137414 | *FAM8A1* | -0.28 | 1.78E-01 | 9.30E-05 | 0.53 | 6.42E-02 | **3.09E-15** | 0.37 | 1.60E-01 | **2.20E-07** | 0.02 | 5.11E-01 | 8.07E-01 | -0.22 | 2.27E-01 | 1.96E-03 | 0.07 | 4.53E-01 | 3.54E-01 |
| ENSG00000137440 | *FGFBP1* | -0.12 | 3.31E-01 | 9.57E-02 | -0.42 | 8.28E-02 | **1.80E-09** | -0.03 | 4.32E-01 | 6.65E-01 | -0.28 | 1.77E-01 | 9.01E-05 | 0.05 | 4.79E-01 | 5.37E-01 | -0.12 | 3.29E-01 | 9.03E-02 |
| ENSG00000137731 | *FXYD2* | 0.56 | 5.56E-02 | **0.00E+00** | -0.16 | 2.95E-01 | 3.35E-02 | -0.29 | 1.74E-01 | 7.78E-05 | 0.41 | 1.29E-01 | **5.51E-09** | 0.21 | 2.94E-01 | 3.24E-03 | 0.13 | 3.78E-01 | 6.58E-02 |
| ENSG00000137809 | *ITGA11* | -0.37 | 1.12E-01 | **1.68E-07** | 0.50 | 7.86E-02 | **2.24E-13** | 0.36 | 1.63E-01 | **2.80E-07** | -0.02 | 4.43E-01 | 7.67E-01 | -0.05 | 4.15E-01 | 5.22E-01 | 0.06 | 4.60E-01 | 4.01E-01 |
| ENSG00000137819 | *PAQR5* | -0.56 | 2.83E-02 | **9.13E-17** | -0.30 | 1.63E-01 | 3.02E-05 | 0.15 | 3.65E-01 | 4.51E-02 | -0.63 | 1.31E-02 | **6.15E-22** | -0.10 | 3.51E-01 | 1.57E-01 | -0.18 | 2.68E-01 | 1.22E-02 |
| ENSG00000138166 | *DUSP5* | 0.06 | 4.67E-01 | 4.46E-01 | -0.51 | 4.49E-02 | **1.27E-13** | -0.33 | 1.40E-01 | 3.82E-06 | -0.18 | 2.72E-01 | 1.43E-02 | 0.06 | 4.59E-01 | 3.96E-01 | -0.17 | 2.85E-01 | 2.36E-02 |
| ENSG00000138622 | *HCN4* | 0.30 | 2.11E-01 | 2.43E-05 | 0.13 | 3.85E-01 | 7.93E-02 | 0.08 | 4.44E-01 | 3.03E-01 | 0.51 | 7.28E-02 | **4.47E-14** | 0.02 | 5.04E-01 | 7.45E-01 | 0.12 | 3.97E-01 | 1.07E-01 |
| ENSG00000138688 | *KIAA1109* | -0.16 | 2.89E-01 | 2.72E-02 | 0.32 | 1.98E-01 | 8.46E-06 | 0.09 | 4.27E-01 | 2.15E-01 | 0.08 | 4.35E-01 | 2.55E-01 | -0.16 | 2.91E-01 | 2.89E-02 | -0.06 | 3.98E-01 | 4.00E-01 |
| ENSG00000138696 | *BMPR1B* | -0.35 | 1.28E-01 | **1.06E-06** | -0.34 | 1.34E-01 | **2.00E-06** | 0.00 | 4.63E-01 | 9.48E-01 | -0.51 | 4.46E-02 | **1.13E-13** | -0.01 | 4.54E-01 | 8.63E-01 | -0.14 | 3.11E-01 | 5.46E-02 |
| ENSG00000140092 | *FBLN5* | -0.29 | 1.70E-01 | 5.12E-05 | -0.22 | 2.36E-01 | 3.04E-03 | -0.15 | 2.97E-01 | 3.48E-02 | -0.41 | 8.99E-02 | **6.12E-09** | -0.17 | 2.82E-01 | 2.14E-02 | -0.25 | 2.04E-01 | 5.45E-04 |
| ENSG00000140285 | *FGF7* | -0.36 | 1.21E-01 | **4.73E-07** | -0.02 | 4.47E-01 | 7.99E-01 | 0.16 | 3.50E-01 | 2.84E-02 | -0.37 | 1.14E-01 | **2.12E-07** | -0.02 | 4.45E-01 | 7.80E-01 | -0.09 | 3.71E-01 | 2.43E-01 |
| ENSG00000140525 | *FANCI* | -0.39 | 1.01E-01 | **3.73E-08** | 0.31 | 2.03E-01 | 1.23E-05 | 0.20 | 3.09E-01 | 6.04E-03 | -0.15 | 2.99E-01 | 3.79E-02 | -0.18 | 2.70E-01 | 1.35E-02 | -0.04 | 4.17E-01 | 5.40E-01 |
| ENSG00000140876 | *NUDT7* | -0.52 | 3.92E-02 | **1.49E-14** | 0.51 | 7.27E-02 | **4.41E-14** | 0.67 | 2.18E-02 | **5.16E-26** | -0.12 | 3.34E-01 | 1.04E-01 | -0.15 | 3.00E-01 | 3.93E-02 | 0.15 | 3.66E-01 | 4.63E-02 |
| ENSG00000141404 | *GNAL* | -0.06 | 4.03E-01 | 4.37E-01 | 0.22 | 2.91E-01 | 2.70E-03 | 0.13 | 3.85E-01 | 7.99E-02 | -0.05 | 4.15E-01 | 5.25E-01 | -0.10 | 3.56E-01 | 1.76E-01 | 0.08 | 4.44E-01 | 3.04E-01 |
| ENSG00000141655 | *TNFRSF11A* | 0.20 | 3.13E-01 | 7.21E-03 | -0.02 | 4.41E-01 | 7.49E-01 | 0.00 | 4.67E-01 | 9.89E-01 | 0.16 | 3.47E-01 | 2.52E-02 | -0.03 | 4.36E-01 | 6.98E-01 | -0.04 | 4.20E-01 | 5.70E-01 |
| ENSG00000142273 | *CBLC* | 0.67 | 2.32E-02 | **2.25E-25** | -0.51 | 4.34E-02 | **7.59E-14** | -0.63 | 1.27E-02 | **3.61E-22** | 0.31 | 2.08E-01 | 1.90E-05 | 0.27 | 2.42E-01 | 2.01E-04 | -0.05 | 4.06E-01 | 4.54E-01 |
| ENSG00000142677 | *IL22RA1* | -0.14 | 3.11E-01 | 5.47E-02 | -0.47 | 5.92E-02 | **1.06E-11** | -0.12 | 3.33E-01 | 1.01E-01 | -0.39 | 1.02E-01 | **4.12E-08** | 0.15 | 3.66E-01 | 4.60E-02 | -0.09 | 3.71E-01 | 2.45E-01 |
| ENSG00000142748 | *FCN3* | 0.47 | 9.75E-02 | **1.77E-11** | -0.29 | 1.71E-01 | 5.58E-05 | -0.36 | 1.19E-01 | **4.00E-07** | 0.17 | 3.44E-01 | 2.25E-02 | 0.01 | 5.22E-01 | 9.12E-01 | -0.03 | 4.32E-01 | 6.70E-01 |
| ENSG00000143196 | *DPT* | 0.19 | 3.23E-01 | 1.08E-02 | -0.29 | 1.68E-01 | 4.59E-05 | -0.28 | 1.77E-01 | 8.57E-05 | -0.12 | 3.36E-01 | 1.10E-01 | 0.05 | 4.69E-01 | 4.64E-01 | -0.15 | 3.00E-01 | 3.84E-02 |
| ENSG00000143494 | *VASH2* | 0.26 | 2.48E-01 | 2.77E-04 | -0.17 | 2.76E-01 | 1.66E-02 | -0.31 | 1.53E-01 | 1.28E-05 | 0.10 | 4.21E-01 | 1.91E-01 | 0.08 | 4.42E-01 | 2.91E-01 | -0.03 | 4.39E-01 | 7.27E-01 |
| ENSG00000144063 | *MALL* | 0.02 | 5.12E-01 | 8.18E-01 | -0.53 | 3.52E-02 | **2.81E-15** | -0.26 | 1.96E-01 | 3.29E-04 | -0.21 | 2.39E-01 | 3.58E-03 | 0.10 | 4.13E-01 | 1.60E-01 | -0.12 | 3.37E-01 | 1.13E-01 |
| ENSG00000144677 | *CTDSPL* | -0.48 | 5.32E-02 | **1.96E-12** | 0.34 | 1.84E-01 | **2.38E-06** | 0.41 | 1.29E-01 | **3.84E-09** | -0.15 | 3.03E-01 | 4.34E-02 | -0.11 | 3.46E-01 | 1.39E-01 | 0.09 | 4.26E-01 | 2.10E-01 |
| ENSG00000144837 | *PLA1A* | -0.09 | 3.65E-01 | 2.14E-01 | 0.34 | 1.79E-01 | **1.43E-06** | 0.41 | 1.32E-01 | **6.11E-09** | 0.11 | 4.04E-01 | 1.28E-01 | -0.05 | 4.16E-01 | 5.36E-01 | 0.14 | 3.68E-01 | 4.88E-02 |
| ENSG00000145220 | *LYAR* | 0.18 | 3.29E-01 | 1.36E-02 | -0.46 | 6.17E-02 | **1.98E-11** | -0.30 | 1.61E-01 | 2.58E-05 | -0.08 | 3.82E-01 | 3.04E-01 | 0.08 | 4.44E-01 | 3.03E-01 | -0.13 | 3.24E-01 | 7.94E-02 |
| ENSG00000145569 | *FAM105A* | -0.10 | 3.59E-01 | 1.87E-01 | 0.60 | 3.95E-02 | **6.26E-20** | 0.58 | 4.67E-02 | **2.95E-18** | 0.35 | 1.73E-01 | **8.13E-07** | -0.14 | 3.14E-01 | 5.96E-02 | 0.34 | 1.78E-01 | **1.34E-06** |
| ENSG00000145888 | *GLRA1* | -0.21 | 2.45E-01 | 4.66E-03 | 0.51 | 7.57E-02 | **1.04E-13** | 0.48 | 9.01E-02 | **3.56E-12** | 0.12 | 3.98E-01 | 1.10E-01 | -0.06 | 3.99E-01 | 4.06E-01 | 0.19 | 3.14E-01 | 7.60E-03 |
| ENSG00000146966 | *DENND2A* | 0.10 | 4.20E-01 | 1.88E-01 | 0.42 | 1.25E-01 | **2.36E-09** | 0.11 | 4.02E-01 | 1.24E-01 | 0.32 | 1.98E-01 | 8.28E-06 | 0.06 | 4.63E-01 | 4.19E-01 | 0.15 | 3.57E-01 | 3.52E-02 |
| ENSG00000147003 | *TMEM27* | -0.30 | 1.65E-01 | 3.57E-05 | 0.14 | 3.72E-01 | 5.49E-02 | 0.42 | 1.24E-01 | **1.86E-09** | -0.09 | 3.61E-01 | 1.95E-01 | -0.01 | 4.60E-01 | 9.20E-01 | 0.17 | 3.35E-01 | 1.69E-02 |
| ENSG00000147257 | *GPC3* | 0.07 | 4.53E-01 | 3.58E-01 | -0.37 | 1.14E-01 | **2.15E-07** | -0.28 | 1.76E-01 | 8.04E-05 | -0.25 | 2.06E-01 | 5.88E-04 | 0.00 | 4.66E-01 | 9.80E-01 | -0.19 | 2.60E-01 | 8.80E-03 |
| ENSG00000147571 | *CRH* | 0.51 | 7.45E-02 | **7.43E-14** | 0.07 | 4.47E-01 | 3.21E-01 | -0.32 | 1.46E-01 | 6.99E-06 | 0.51 | 7.64E-02 | **1.25E-13** | 0.03 | 4.99E-01 | 7.01E-01 | 0.18 | 3.26E-01 | 1.18E-02 |
| ENSG00000148344 | *PTGES* | 0.17 | 3.36E-01 | 1.74E-02 | -0.40 | 9.17E-02 | **8.30E-09** | -0.40 | 9.37E-02 | **1.14E-08** | -0.10 | 3.52E-01 | 1.61E-01 | 0.10 | 4.13E-01 | 1.60E-01 | -0.24 | 2.16E-01 | 1.06E-03 |
| ENSG00000149328 | *GLB1L2* | -0.24 | 2.12E-01 | 8.62E-04 | 0.28 | 2.31E-01 | 9.38E-05 | 0.21 | 2.96E-01 | 3.43E-03 | -0.03 | 4.32E-01 | 6.67E-01 | 0.01 | 5.23E-01 | 9.23E-01 | 0.11 | 4.09E-01 | 1.45E-01 |
| ENSG00000150526 | *MIA2* | -0.35 | 1.26E-01 | **8.69E-07** | 0.72 | 1.42E-02 | **7.80E-31** | 0.51 | 7.26E-02 | **4.28E-14** | 0.10 | 4.12E-01 | 1.55E-01 | -0.16 | 2.92E-01 | 2.96E-02 | 0.19 | 3.17E-01 | 8.38E-03 |
| ENSG00000150764 | *DIXDC1* | -0.40 | 9.46E-02 | **1.31E-08** | 0.52 | 7.10E-02 | **2.65E-14** | 0.55 | 5.57E-02 | **1.43E-16** | -0.04 | 4.20E-01 | 5.66E-01 | -0.24 | 2.17E-01 | 1.16E-03 | 0.10 | 4.21E-01 | 1.92E-01 |
| ENSG00000151458 | *ANKRD50* | -0.76 | 1.23E-03 | **1.12E-36** | 0.18 | 3.32E-01 | 1.50E-02 | 0.51 | 7.35E-02 | **5.53E-14** | -0.49 | 4.96E-02 | **6.23E-13** | -0.19 | 2.59E-01 | 8.57E-03 | -0.09 | 3.67E-01 | 2.23E-01 |
| ENSG00000151470 | *C4orf33* | -0.48 | 5.53E-02 | **3.63E-12** | 0.51 | 7.41E-02 | **6.55E-14** | 0.59 | 4.24E-02 | **3.24E-19** | -0.15 | 2.96E-01 | 3.42E-02 | -0.13 | 3.23E-01 | 7.82E-02 | 0.14 | 3.73E-01 | 5.75E-02 |
| ENSG00000151689 | *INPP1* | 0.01 | 5.21E-01 | 8.98E-01 | -0.18 | 2.75E-01 | 1.62E-02 | -0.08 | 3.75E-01 | 2.66E-01 | -0.08 | 3.73E-01 | 2.56E-01 | -0.02 | 4.47E-01 | 7.96E-01 | -0.04 | 4.20E-01 | 5.69E-01 |
| ENSG00000152583 | *SPARCL1* | 0.29 | 2.27E-01 | 7.10E-05 | -0.28 | 1.76E-01 | 8.34E-05 | -0.30 | 1.59E-01 | 2.15E-05 | -0.02 | 4.48E-01 | 8.07E-01 | 0.02 | 5.12E-01 | 8.16E-01 | -0.12 | 3.34E-01 | 1.04E-01 |
| ENSG00000153162 | *BMP6* | 0.14 | 3.76E-01 | 6.21E-02 | -0.35 | 1.28E-01 | **1.11E-06** | -0.28 | 1.81E-01 | 1.15E-04 | -0.14 | 3.09E-01 | 5.15E-02 | 0.12 | 3.92E-01 | 9.58E-02 | -0.19 | 2.59E-01 | 8.59E-03 |
| ENSG00000153551 | *CMTM7* | 0.28 | 2.31E-01 | 9.52E-05 | -0.64 | 1.16E-02 | **9.60E-23** | -0.42 | 8.29E-02 | **1.83E-09** | 0.01 | 5.23E-01 | 9.15E-01 | 0.20 | 3.09E-01 | 6.06E-03 | -0.09 | 3.66E-01 | 2.21E-01 |
| ENSG00000153822 | *KCNJ16* | -0.68 | 6.35E-03 | **9.90E-27** | 0.35 | 1.76E-01 | **1.12E-06** | 0.69 | 1.82E-02 | **5.42E-28** | -0.35 | 1.29E-01 | **1.23E-06** | -0.14 | 3.06E-01 | 4.77E-02 | 0.16 | 3.51E-01 | 2.86E-02 |
| ENSG00000153823 | *PID1* | -0.04 | 4.27E-01 | 6.24E-01 | -0.22 | 2.28E-01 | 2.00E-03 | -0.14 | 3.09E-01 | 5.20E-02 | -0.23 | 2.18E-01 | 1.20E-03 | 0.07 | 4.55E-01 | 3.68E-01 | -0.14 | 3.15E-01 | 6.12E-02 |
| ENSG00000153982 | *GDPD1* | -0.51 | 4.35E-02 | **7.88E-14** | 0.45 | 1.09E-01 | **1.55E-10** | 0.58 | 4.78E-02 | **5.06E-18** | -0.20 | 2.48E-01 | 5.21E-03 | -0.13 | 3.27E-01 | 8.55E-02 | 0.11 | 4.10E-01 | 1.48E-01 |
| ENSG00000154175 | *ABI3BP* | 0.04 | 4.88E-01 | 6.07E-01 | -0.18 | 2.75E-01 | 1.60E-02 | -0.32 | 1.44E-01 | 5.77E-06 | -0.09 | 3.64E-01 | 2.10E-01 | -0.01 | 4.58E-01 | 9.06E-01 | -0.22 | 2.31E-01 | 2.35E-03 |
| ENSG00000154274 | *C4orf19* | -0.56 | 2.66E-02 | **3.47E-17** | 0.04 | 4.88E-01 | 6.13E-01 | 0.33 | 1.85E-01 | **2.66E-06** | -0.45 | 6.59E-02 | **5.43E-11** | -0.10 | 3.50E-01 | 1.56E-01 | -0.01 | 4.59E-01 | 9.13E-01 |
| ENSG00000154864 | *PIEZO2* | -0.38 | 1.06E-01 | **7.60E-08** | 0.42 | 1.23E-01 | **1.60E-09** | 0.39 | 1.47E-01 | **4.38E-08** | -0.06 | 3.97E-01 | 3.93E-01 | -0.04 | 4.26E-01 | 6.19E-01 | 0.05 | 4.75E-01 | 5.10E-01 |
| ENSG00000154914 | *USP43* | 0.34 | 1.80E-01 | **1.65E-06** | -0.67 | 6.85E-03 | **3.00E-26** | -0.57 | 2.54E-02 | **1.65E-17** | -0.09 | 3.63E-01 | 2.08E-01 | 0.16 | 3.52E-01 | 3.04E-02 | -0.23 | 2.27E-01 | 1.91E-03 |
| ENSG00000155324 | *GRAMD3* | -0.24 | 2.15E-01 | 9.99E-04 | 0.56 | 5.47E-02 | **9.62E-17** | 0.52 | 7.10E-02 | **2.64E-14** | 0.18 | 3.32E-01 | 1.50E-02 | 0.01 | 5.14E-01 | 8.40E-01 | 0.26 | 2.51E-01 | 3.35E-04 |
| ENSG00000155755 | *TMEM237* | -0.48 | 5.64E-02 | **4.99E-12** | 0.48 | 9.01E-02 | **3.56E-12** | 0.60 | 4.14E-02 | **1.86E-19** | -0.10 | 3.55E-01 | 1.72E-01 | -0.09 | 3.68E-01 | 2.30E-01 | 0.19 | 3.20E-01 | 9.66E-03 |
| ENSG00000156500 | *FAM122C* | 0.12 | 3.89E-01 | 8.84E-02 | -0.09 | 3.63E-01 | 2.07E-01 | -0.07 | 3.91E-01 | 3.53E-01 | 0.06 | 4.68E-01 | 4.52E-01 | -0.05 | 4.13E-01 | 5.08E-01 | -0.01 | 4.59E-01 | 9.13E-01 |
| ENSG00000156687 | *UNC5D* | -0.22 | 2.30E-01 | 2.31E-03 | 0.36 | 1.62E-01 | **2.66E-07** | 0.58 | 4.71E-02 | **3.59E-18** | 0.14 | 3.74E-01 | 5.90E-02 | -0.01 | 4.51E-01 | 8.39E-01 | 0.20 | 3.08E-01 | 5.82E-03 |
| ENSG00000156876 | *SASS6* | -0.22 | 2.28E-01 | 1.99E-03 | 0.16 | 3.48E-01 | 2.66E-02 | 0.23 | 2.82E-01 | 1.80E-03 | -0.02 | 4.46E-01 | 7.88E-01 | -0.03 | 4.30E-01 | 6.49E-01 | 0.10 | 4.17E-01 | 1.74E-01 |
| ENSG00000157168 | *NRG1* | -0.36 | 1.21E-01 | **4.70E-07** | 0.24 | 2.70E-01 | 9.84E-04 | 0.25 | 2.62E-01 | 6.50E-04 | -0.08 | 3.72E-01 | 2.47E-01 | 0.04 | 4.86E-01 | 5.96E-01 | 0.13 | 3.85E-01 | 7.98E-02 |
| ENSG00000157765 | *SLC34A2* | -0.35 | 1.25E-01 | **7.89E-07** | -0.27 | 1.84E-01 | 1.50E-04 | -0.02 | 4.45E-01 | 7.83E-01 | -0.44 | 7.25E-02 | **2.34E-10** | -0.10 | 3.55E-01 | 1.72E-01 | -0.19 | 2.60E-01 | 8.77E-03 |
| ENSG00000159322 | *ADPGK* | -0.08 | 3.80E-01 | 2.90E-01 | -0.48 | 5.65E-02 | **5.09E-12** | -0.17 | 2.76E-01 | 1.70E-02 | -0.26 | 1.99E-01 | 4.04E-04 | 0.09 | 4.32E-01 | 2.40E-01 | -0.18 | 2.74E-01 | 1.54E-02 |
| ENSG00000160695 | *VPS11* | -0.22 | 2.34E-01 | 2.71E-03 | 0.36 | 1.69E-01 | **5.58E-07** | 0.31 | 2.05E-01 | 1.42E-05 | 0.09 | 4.25E-01 | 2.08E-01 | 0.10 | 4.16E-01 | 1.70E-01 | 0.11 | 4.05E-01 | 1.32E-01 |
| ENSG00000161800 | *RACGAP1* | -0.70 | 4.18E-03 | **2.14E-29** | 0.50 | 8.02E-02 | **3.37E-13** | 0.66 | 2.44E-02 | **7.85E-25** | -0.28 | 1.82E-01 | 1.31E-04 | -0.13 | 3.27E-01 | 8.59E-02 | 0.14 | 3.76E-01 | 6.17E-02 |
| ENSG00000162616 | *DNAJB4* | -0.66 | 7.92E-03 | **2.47E-25** | 0.35 | 1.75E-01 | **1.02E-06** | 0.58 | 4.65E-02 | **2.72E-18** | -0.38 | 1.06E-01 | **7.03E-08** | -0.10 | 3.54E-01 | 1.69E-01 | 0.03 | 4.95E-01 | 6.70E-01 |
| ENSG00000162949 | *CAPN13* | -0.04 | 4.17E-01 | 5.43E-01 | 0.33 | 1.87E-01 | **3.24E-06** | 0.44 | 1.15E-01 | **4.26E-10** | 0.20 | 3.12E-01 | 6.82E-03 | 0.02 | 5.10E-01 | 8.03E-01 | 0.17 | 3.39E-01 | 1.94E-02 |
| ENSG00000163393 | *SLC22A15* | -0.14 | 3.11E-01 | 5.46E-02 | 0.58 | 4.54E-02 | **1.57E-18** | 0.35 | 1.74E-01 | **9.12E-07** | 0.27 | 2.45E-01 | 2.29E-04 | -0.06 | 3.99E-01 | 4.09E-01 | 0.22 | 2.93E-01 | 3.04E-03 |
| ENSG00000163535 | *SGOL2* | -0.28 | 1.82E-01 | 1.24E-04 | 0.19 | 3.20E-01 | 9.52E-03 | 0.23 | 2.80E-01 | 1.66E-03 | -0.07 | 3.91E-01 | 3.53E-01 | -0.04 | 4.21E-01 | 5.71E-01 | 0.08 | 4.42E-01 | 2.91E-01 |
| ENSG00000163581 | *SLC2A2* | -0.47 | 5.80E-02 | **7.56E-12** | 0.38 | 1.48E-01 | **5.02E-08** | 0.75 | 1.02E-02 | **9.41E-35** | -0.10 | 3.59E-01 | 1.91E-01 | -0.03 | 4.32E-01 | 6.70E-01 | 0.21 | 3.03E-01 | 4.70E-03 |
| ENSG00000163661 | *PTX3* | -0.24 | 2.13E-01 | 9.17E-04 | -0.41 | 9.09E-02 | **7.20E-09** | -0.10 | 3.60E-01 | 1.92E-01 | -0.38 | 1.08E-01 | **9.91E-08** | -0.07 | 3.93E-01 | 3.66E-01 | -0.12 | 3.28E-01 | 8.82E-02 |
| ENSG00000163735 | *CXCL5* | -0.02 | 4.41E-01 | 7.44E-01 | -0.34 | 1.33E-01 | **1.77E-06** | -0.21 | 2.38E-01 | 3.35E-03 | -0.23 | 2.25E-01 | 1.75E-03 | 0.00 | 4.65E-01 | 9.72E-01 | -0.14 | 3.10E-01 | 5.38E-02 |
| ENSG00000164078 | *MST1R* | 0.07 | 4.49E-01 | 3.28E-01 | -0.49 | 5.20E-02 | **1.32E-12** | -0.22 | 2.29E-01 | 2.20E-03 | -0.10 | 3.56E-01 | 1.79E-01 | 0.23 | 2.82E-01 | 1.79E-03 | -0.06 | 3.98E-01 | 4.02E-01 |
| ENSG00000164111 | *ANXA5* | -0.47 | 5.91E-02 | **1.02E-11** | -0.28 | 1.82E-01 | 1.23E-04 | 0.17 | 3.36E-01 | 1.73E-02 | -0.48 | 5.67E-02 | **5.41E-12** | -0.06 | 4.02E-01 | 4.28E-01 | -0.08 | 3.79E-01 | 2.84E-01 |
| ENSG00000166250 | *CLMP* | -0.13 | 3.21E-01 | 7.38E-02 | -0.26 | 1.93E-01 | 2.64E-04 | -0.10 | 3.55E-01 | 1.74E-01 | -0.35 | 1.25E-01 | **7.96E-07** | 0.01 | 5.21E-01 | 9.00E-01 | -0.16 | 2.86E-01 | 2.40E-02 |
| ENSG00000166387 | *PPFIBP2* | -0.42 | 8.13E-02 | **1.38E-09** | 0.25 | 2.62E-01 | 6.23E-04 | 0.33 | 1.87E-01 | **3.12E-06** | -0.13 | 3.23E-01 | 7.83E-02 | -0.05 | 4.06E-01 | 4.58E-01 | 0.10 | 4.21E-01 | 1.89E-01 |
| ENSG00000166482 | *MFAP4* | 0.28 | 2.33E-01 | 1.09E-04 | -0.36 | 1.19E-01 | **4.09E-07** | -0.38 | 1.09E-01 | **1.05E-07** | -0.06 | 3.99E-01 | 4.11E-01 | 0.10 | 4.20E-01 | 1.86E-01 | -0.15 | 3.03E-01 | 4.32E-02 |
| ENSG00000166670 | *MMP10* | -0.20 | 2.54E-01 | 6.77E-03 | -0.25 | 2.03E-01 | 5.02E-04 | -0.04 | 4.22E-01 | 5.87E-01 | -0.32 | 1.44E-01 | 5.50E-06 | 0.01 | 5.22E-01 | 9.14E-01 | -0.11 | 3.39E-01 | 1.18E-01 |
| ENSG00000168566 | *SNRNP48* | -0.15 | 3.06E-01 | 4.68E-02 | 0.23 | 2.76E-01 | 1.32E-03 | 0.10 | 4.18E-01 | 1.80E-01 | -0.02 | 4.46E-01 | 7.88E-01 | -0.03 | 4.33E-01 | 6.80E-01 | 0.03 | 5.01E-01 | 7.17E-01 |
| ENSG00000169071 | *ROR2* | 0.68 | 2.04E-02 | **9.18E-27** | -0.23 | 2.24E-01 | 1.65E-03 | -0.59 | 1.92E-02 | **2.26E-19** | 0.47 | 9.75E-02 | **1.75E-11** | 0.19 | 3.18E-01 | 8.96E-03 | -0.05 | 4.08E-01 | 4.71E-01 |
| ENSG00000170312 | *CDK1* | -0.44 | 7.27E-02 | **2.47E-10** | 0.55 | 5.88E-02 | **4.43E-16** | 0.49 | 8.27E-02 | **6.26E-13** | -0.01 | 4.56E-01 | 8.87E-01 | -0.05 | 4.08E-01 | 4.70E-01 | 0.17 | 3.39E-01 | 1.94E-02 |
| ENSG00000170369 | *CST2* | 0.48 | 8.71E-02 | **1.80E-12** | -0.33 | 1.42E-01 | 4.92E-06 | -0.47 | 6.06E-02 | **1.51E-11** | 0.10 | 4.19E-01 | 1.81E-01 | 0.02 | 5.07E-01 | 7.77E-01 | -0.24 | 2.17E-01 | 1.12E-03 |
| ENSG00000170542 | *SERPINB9* | -0.30 | 1.64E-01 | 3.30E-05 | -0.38 | 1.04E-01 | **5.80E-08** | -0.02 | 4.48E-01 | 8.06E-01 | -0.41 | 9.06E-02 | **6.83E-09** | 0.03 | 5.00E-01 | 7.12E-01 | -0.17 | 2.83E-01 | 2.18E-02 |
| ENSG00000170827 | *CELP* | 0.01 | 5.17E-01 | 8.63E-01 | -0.38 | 1.04E-01 | **5.33E-08** | -0.24 | 2.10E-01 | 7.62E-04 | -0.26 | 1.95E-01 | 3.14E-04 | 0.02 | 5.10E-01 | 8.00E-01 | -0.10 | 3.57E-01 | 1.79E-01 |
| ENSG00000171004 | *HS6ST2* | -0.22 | 2.32E-01 | 2.54E-03 | 0.39 | 1.44E-01 | **3.00E-08** | 0.49 | 8.29E-02 | **6.58E-13** | 0.06 | 4.65E-01 | 4.38E-01 | -0.12 | 3.33E-01 | 1.03E-01 | 0.25 | 2.60E-01 | 5.62E-04 |
| ENSG00000171522 | *PTGER4* | 0.12 | 3.95E-01 | 1.03E-01 | -0.01 | 4.59E-01 | 9.12E-01 | -0.21 | 2.37E-01 | 3.24E-03 | 0.06 | 4.58E-01 | 3.86E-01 | -0.01 | 4.55E-01 | 8.75E-01 | -0.02 | 4.49E-01 | 8.18E-01 |
| ENSG00000171552 | *BCL2L1* | 0.42 | 1.23E-01 | **1.78E-09** | -0.49 | 5.04E-02 | **8.02E-13** | -0.50 | 4.73E-02 | **2.94E-13** | 0.07 | 4.56E-01 | 3.75E-01 | 0.18 | 3.26E-01 | 1.20E-02 | -0.12 | 3.30E-01 | 9.42E-02 |
| ENSG00000172020 | *GAP43* | 0.04 | 4.86E-01 | 5.90E-01 | 0.19 | 3.13E-01 | 7.34E-03 | 0.21 | 2.94E-01 | 3.22E-03 | 0.11 | 4.00E-01 | 1.17E-01 | -0.01 | 4.56E-01 | 8.79E-01 | 0.12 | 3.92E-01 | 9.57E-02 |
| ENSG00000172575 | *RASGRP1* | -0.48 | 5.34E-02 | **2.06E-12** | 0.44 | 1.09E-01 | **1.69E-10** | 0.64 | 2.83E-02 | **2.45E-23** | 0.00 | 5.26E-01 | 9.50E-01 | -0.18 | 2.71E-01 | 1.41E-02 | 0.16 | 3.55E-01 | 3.28E-02 |
| ENSG00000173611 | *SCAI* | -0.33 | 1.43E-01 | 4.95E-06 | 0.45 | 1.07E-01 | **1.05E-10** | 0.59 | 4.27E-02 | **3.78E-19** | 0.04 | 4.85E-01 | 5.88E-01 | -0.10 | 3.59E-01 | 1.88E-01 | 0.26 | 2.52E-01 | 3.66E-04 |
| ENSG00000173918 | *C1QTNF1* | 0.57 | 5.11E-02 | **2.08E-17** | -0.56 | 2.78E-02 | **6.68E-17** | -0.65 | 9.70E-03 | **5.80E-24** | 0.13 | 3.85E-01 | 8.03E-02 | 0.14 | 3.68E-01 | 4.88E-02 | -0.24 | 2.09E-01 | 7.21E-04 |
| ENSG00000174080 | *CTSF* | 0.23 | 2.75E-01 | 1.25E-03 | 0.27 | 2.44E-01 | 2.26E-04 | -0.08 | 3.77E-01 | 2.77E-01 | 0.35 | 1.75E-01 | **9.99E-07** | -0.01 | 4.62E-01 | 9.39E-01 | 0.20 | 3.05E-01 | 5.14E-03 |
| ENSG00000174348 | *PODN* | 0.66 | 2.53E-02 | **1.75E-24** | -0.29 | 1.70E-01 | 5.02E-05 | -0.54 | 3.49E-02 | **2.42E-15** | 0.26 | 2.46E-01 | 2.47E-04 | 0.22 | 2.84E-01 | 1.94E-03 | -0.05 | 4.13E-01 | 5.10E-01 |
| ENSG00000175040 | *CHST2* | 0.80 | 4.64E-03 | **5.51E-44** | -0.39 | 9.98E-02 | **2.91E-08** | -0.65 | 9.21E-03 | **2.57E-24** | 0.44 | 1.10E-01 | **1.99E-10** | 0.21 | 2.94E-01 | 3.19E-03 | -0.09 | 3.67E-01 | 2.24E-01 |
| ENSG00000175505 | *CLCF1* | 0.46 | 9.85E-02 | **2.17E-11** | -0.68 | 6.66E-03 | **1.98E-26** | -0.61 | 1.60E-02 | **1.27E-20** | 0.00 | 5.28E-01 | 9.65E-01 | 0.21 | 2.96E-01 | 3.43E-03 | -0.21 | 2.39E-01 | 3.54E-03 |
| ENSG00000175592 | *FOSL1* | 0.48 | 8.96E-02 | **3.22E-12** | -0.60 | 1.90E-02 | **1.76E-19** | -0.53 | 3.56E-02 | **3.33E-15** | 0.04 | 4.82E-01 | 5.59E-01 | 0.17 | 3.43E-01 | 2.19E-02 | -0.17 | 2.79E-01 | 1.92E-02 |
| ENSG00000176014 | *TUBB6* | 0.56 | 5.30E-02 | **4.83E-17** | -0.69 | 4.96E-03 | **2.55E-28** | -0.64 | 1.04E-02 | **1.86E-23** | 0.07 | 4.48E-01 | 3.26E-01 | 0.20 | 3.12E-01 | 6.98E-03 | -0.23 | 2.26E-01 | 1.84E-03 |
| ENSG00000176533 | *GNG7* | 0.03 | 5.00E-01 | 7.11E-01 | 0.45 | 1.08E-01 | **1.30E-10** | 0.35 | 1.72E-01 | **7.24E-07** | 0.39 | 1.47E-01 | **4.83E-08** | 0.03 | 4.97E-01 | 6.86E-01 | 0.25 | 2.63E-01 | 6.55E-04 |
| ENSG00000176928 | *GCNT4* | -0.44 | 7.24E-02 | **2.29E-10** | 0.40 | 1.38E-01 | **1.41E-08** | 0.68 | 2.02E-02 | **7.72E-27** | -0.07 | 3.93E-01 | 3.69E-01 | -0.07 | 3.93E-01 | 3.67E-01 | 0.28 | 2.29E-01 | 8.51E-05 |
| ENSG00000177459 | *ERICH5* | -0.42 | 8.15E-02 | **1.45E-09** | 0.13 | 3.88E-01 | 8.60E-02 | 0.47 | 9.53E-02 | **1.11E-11** | -0.14 | 3.11E-01 | 5.52E-02 | -0.01 | 4.54E-01 | 8.65E-01 | 0.15 | 3.62E-01 | 4.09E-02 |
| ENSG00000178878 | *APOLD1* | 0.15 | 3.61E-01 | 3.95E-02 | -0.05 | 4.08E-01 | 4.68E-01 | -0.19 | 2.62E-01 | 9.82E-03 | 0.06 | 4.60E-01 | 3.99E-01 | -0.03 | 4.39E-01 | 7.25E-01 | -0.02 | 4.40E-01 | 7.40E-01 |
| ENSG00000179041 | *RRS1* | 0.60 | 4.17E-02 | **2.16E-19** | -0.66 | 8.50E-03 | **7.30E-25** | -0.63 | 1.22E-02 | **2.01E-22** | 0.18 | 3.31E-01 | 1.47E-02 | 0.20 | 3.10E-01 | 6.45E-03 | -0.16 | 2.89E-01 | 2.71E-02 |
| ENSG00000179241 | *LDLRAD3* | 0.32 | 1.99E-01 | 8.68E-06 | 0.07 | 4.55E-01 | 3.67E-01 | -0.21 | 2.37E-01 | 3.21E-03 | 0.30 | 2.17E-01 | 3.75E-05 | -0.03 | 4.38E-01 | 7.18E-01 | 0.03 | 5.00E-01 | 7.13E-01 |
| ENSG00000179331 | *RAB39A* | -0.05 | 4.07E-01 | 4.66E-01 | 0.52 | 7.19E-02 | **3.46E-14** | 0.38 | 1.54E-01 | **1.02E-07** | 0.33 | 1.88E-01 | 3.38E-06 | 0.00 | 4.65E-01 | 9.65E-01 | 0.22 | 2.93E-01 | 2.99E-03 |
| ENSG00000179431 | *FJX1* | 0.61 | 3.77E-02 | **2.14E-20** | -0.52 | 3.88E-02 | **1.28E-14** | -0.59 | 1.96E-02 | **3.06E-19** | 0.24 | 2.64E-01 | 7.22E-04 | 0.21 | 2.97E-01 | 3.58E-03 | -0.10 | 3.56E-01 | 1.76E-01 |
| ENSG00000180011 | *ZADH2* | -0.20 | 2.47E-01 | 5.17E-03 | 0.46 | 1.01E-01 | **3.88E-11** | 0.29 | 2.26E-01 | 6.94E-05 | 0.12 | 3.91E-01 | 9.26E-02 | -0.06 | 4.04E-01 | 4.42E-01 | 0.18 | 3.30E-01 | 1.40E-02 |
| ENSG00000180801 | *ARSJ* | -0.41 | 8.95E-02 | **5.68E-09** | -0.23 | 2.19E-01 | 1.29E-03 | 0.11 | 4.11E-01 | 1.51E-01 | -0.49 | 5.18E-02 | **1.25E-12** | 0.07 | 4.48E-01 | 3.26E-01 | -0.08 | 3.73E-01 | 2.51E-01 |
| ENSG00000180875 | *GREM2* | -0.02 | 4.45E-01 | 7.79E-01 | 0.20 | 3.10E-01 | 6.25E-03 | 0.17 | 3.43E-01 | 2.18E-02 | 0.15 | 3.58E-01 | 3.61E-02 | -0.05 | 4.14E-01 | 5.20E-01 | 0.21 | 2.97E-01 | 3.68E-03 |
| ENSG00000182022 | *CHST15* | -0.28 | 1.75E-01 | 7.44E-05 | -0.37 | 1.16E-01 | **2.59E-07** | 0.00 | 4.64E-01 | 9.57E-01 | -0.37 | 1.12E-01 | **1.64E-07** | 0.05 | 4.79E-01 | 5.36E-01 | -0.24 | 2.13E-01 | 9.09E-04 |
| ENSG00000182359 | *KBTBD3* | 0.22 | 2.88E-01 | 2.42E-03 | 0.29 | 2.21E-01 | 4.71E-05 | 0.10 | 4.15E-01 | 1.67E-01 | 0.30 | 2.12E-01 | 2.54E-05 | -0.05 | 4.08E-01 | 4.73E-01 | 0.19 | 3.18E-01 | 8.68E-03 |
| ENSG00000182747 | *SLC35D3* | 0.71 | 1.51E-02 | **4.06E-30** | -0.08 | 3.74E-01 | 2.60E-01 | -0.32 | 1.46E-01 | 6.84E-06 | 0.55 | 5.84E-02 | **3.90E-16** | 0.13 | 3.86E-01 | 8.11E-02 | 0.08 | 4.40E-01 | 2.79E-01 |
| ENSG00000183044 | *ABAT* | -0.27 | 1.87E-01 | 1.77E-04 | 0.55 | 5.71E-02 | **2.41E-16** | 0.58 | 4.82E-02 | **6.00E-18** | 0.10 | 4.17E-01 | 1.75E-01 | -0.03 | 4.35E-01 | 6.97E-01 | 0.26 | 2.53E-01 | 3.88E-04 |
| ENSG00000183255 | *PTTG1IP* | 0.10 | 4.15E-01 | 1.67E-01 | -0.42 | 8.41E-02 | **2.28E-09** | -0.26 | 1.97E-01 | 3.42E-04 | -0.03 | 4.33E-01 | 6.74E-01 | 0.18 | 3.30E-01 | 1.42E-02 | -0.07 | 3.86E-01 | 3.27E-01 |
| ENSG00000184160 | *ADRA2C* | 0.75 | 1.00E-02 | **6.38E-35** | -0.51 | 4.33E-02 | **7.13E-14** | -0.72 | 3.01E-03 | **1.91E-31** | 0.31 | 2.02E-01 | 1.12E-05 | 0.18 | 3.26E-01 | 1.22E-02 | -0.16 | 2.95E-01 | 3.36E-02 |
| ENSG00000184220 | *CMSS1* | -0.26 | 1.98E-01 | 3.67E-04 | -0.12 | 3.34E-01 | 1.04E-01 | 0.21 | 2.98E-01 | 3.83E-03 | -0.33 | 1.36E-01 | **2.66E-06** | 0.06 | 4.64E-01 | 4.26E-01 | -0.02 | 4.47E-01 | 8.04E-01 |
| ENSG00000184459 | *BPIFC* | -0.28 | 1.81E-01 | 1.18E-04 | 0.38 | 1.55E-01 | **1.13E-07** | 0.61 | 3.85E-02 | **3.42E-20** | -0.01 | 4.56E-01 | 8.83E-01 | -0.12 | 3.38E-01 | 1.16E-01 | 0.18 | 3.25E-01 | 1.16E-02 |
| ENSG00000184588 | *PDE4B* | 0.17 | 3.42E-01 | 2.12E-02 | -0.18 | 2.71E-01 | 1.41E-02 | -0.23 | 2.25E-01 | 1.76E-03 | -0.03 | 4.39E-01 | 7.32E-01 | 0.01 | 5.22E-01 | 9.11E-01 | -0.12 | 3.28E-01 | 8.82E-02 |
| ENSG00000184640 | *SEPT9* | -0.20 | 2.46E-01 | 4.93E-03 | -0.39 | 9.92E-02 | **2.68E-08** | -0.23 | 2.18E-01 | 1.21E-03 | -0.38 | 1.06E-01 | **6.80E-08** | 0.04 | 4.84E-01 | 5.75E-01 | -0.27 | 1.85E-01 | 1.62E-04 |
| ENSG00000185345 | *PARK2* | -0.29 | 1.72E-01 | 6.20E-05 | 0.43 | 1.21E-01 | **1.17E-09** | 0.34 | 1.84E-01 | **2.43E-06** | 0.13 | 3.81E-01 | 7.18E-02 | -0.09 | 3.69E-01 | 2.34E-01 | 0.18 | 3.33E-01 | 1.56E-02 |
| ENSG00000186187 | *ZNRF1* | 0.73 | 1.18E-02 | **5.34E-33** | -0.37 | 1.15E-01 | **2.39E-07** | -0.70 | 4.47E-03 | **5.53E-29** | 0.46 | 1.02E-01 | **4.82E-11** | 0.16 | 3.46E-01 | 2.46E-02 | -0.03 | 4.34E-01 | 6.87E-01 |
| ENSG00000186188 | *FFAR4* | -0.06 | 4.00E-01 | 4.12E-01 | 0.38 | 1.49E-01 | **5.98E-08** | 0.41 | 1.33E-01 | **6.86E-09** | 0.39 | 1.45E-01 | **3.43E-08** | -0.03 | 4.35E-01 | 6.93E-01 | 0.21 | 3.01E-01 | 4.21E-03 |
| ENSG00000186340 | *THBS2* | 0.09 | 4.32E-01 | 2.37E-01 | -0.15 | 2.96E-01 | 3.44E-02 | -0.30 | 1.64E-01 | 3.17E-05 | -0.08 | 3.76E-01 | 2.72E-01 | 0.06 | 4.63E-01 | 4.24E-01 | -0.21 | 2.37E-01 | 3.17E-03 |
| ENSG00000188042 | *ARL4C* | 0.47 | 9.63E-02 | **1.37E-11** | -0.42 | 8.44E-02 | **2.40E-09** | -0.59 | 2.02E-02 | **4.89E-19** | 0.15 | 3.59E-01 | 3.80E-02 | -0.01 | 4.53E-01 | 8.52E-01 | -0.15 | 3.05E-01 | 4.60E-02 |
| ENSG00000188229 | *TUBB4B* | 0.54 | 6.05E-02 | **8.53E-16** | -0.66 | 8.36E-03 | **5.42E-25** | -0.51 | 4.26E-02 | **5.61E-14** | 0.16 | 3.47E-01 | 2.55E-02 | 0.33 | 1.91E-01 | 4.46E-06 | -0.08 | 3.82E-01 | 3.00E-01 |
| ENSG00000188266 | *HYKK* | 0.03 | 4.94E-01 | 6.62E-01 | 0.17 | 3.45E-01 | 2.35E-02 | 0.10 | 4.19E-01 | 1.81E-01 | 0.14 | 3.76E-01 | 6.25E-02 | 0.05 | 4.69E-01 | 4.63E-01 | 0.13 | 3.87E-01 | 8.37E-02 |
| ENSG00000188643 | *S100A16* | 0.33 | 1.91E-01 | 4.72E-06 | -0.60 | 1.78E-02 | **6.33E-20** | -0.39 | 9.77E-02 | **2.11E-08** | -0.04 | 4.26E-01 | 6.12E-01 | 0.20 | 3.07E-01 | 5.70E-03 | -0.11 | 3.43E-01 | 1.31E-01 |
| ENSG00000189058 | *APOD* | 0.18 | 3.29E-01 | 1.32E-02 | -0.36 | 1.16E-01 | **2.72E-07** | -0.37 | 1.15E-01 | **2.49E-07** | -0.14 | 3.13E-01 | 5.80E-02 | 0.03 | 4.92E-01 | 6.45E-01 | -0.18 | 2.72E-01 | 1.43E-02 |
| ENSG00000189337 | *KAZN* | 0.48 | 8.98E-02 | **3.38E-12** | -0.36 | 1.16E-01 | **2.61E-07** | -0.51 | 4.34E-02 | **7.58E-14** | 0.20 | 3.12E-01 | 6.98E-03 | 0.10 | 4.19E-01 | 1.83E-01 | -0.13 | 3.20E-01 | 7.10E-02 |
| ENSG00000196177 | *ACADSB* | -0.39 | 9.75E-02 | **2.07E-08** | 0.45 | 1.06E-01 | **1.03E-10** | 0.55 | 5.93E-02 | **5.37E-16** | -0.04 | 4.20E-01 | 5.68E-01 | -0.20 | 2.53E-01 | 6.46E-03 | 0.11 | 4.04E-01 | 1.29E-01 |
| ENSG00000196312 | *HIATL2* | 0.71 | 1.45E-02 | **1.40E-30** | -0.05 | 4.07E-01 | 4.66E-01 | -0.35 | 1.27E-01 | **9.58E-07** | 0.59 | 4.23E-02 | **3.02E-19** | 0.14 | 3.75E-01 | 6.01E-02 | 0.12 | 3.91E-01 | 9.36E-02 |
| ENSG00000197586 | *ENTPD6* | 0.65 | 2.75E-02 | **1.25E-23** | -0.51 | 4.17E-02 | **3.99E-14** | -0.60 | 1.80E-02 | **7.29E-20** | 0.35 | 1.76E-01 | **1.09E-06** | 0.19 | 3.13E-01 | 7.34E-03 | -0.09 | 3.71E-01 | 2.43E-01 |
| ENSG00000197747 | *S100A10* | -0.11 | 3.45E-01 | 1.38E-01 | -0.57 | 2.63E-02 | **0.00E+00** | -0.17 | 2.78E-01 | 1.81E-02 | -0.31 | 1.56E-01 | 1.81E-05 | 0.07 | 4.51E-01 | 3.42E-01 | -0.10 | 3.51E-01 | 1.58E-01 |
| ENSG00000197956 | *S100A6* | 0.33 | 1.88E-01 | 3.95E-06 | -0.54 | 3.40E-02 | **0.00E+00** | -0.37 | 1.13E-01 | **2.25E-07** | 0.00 | 5.29E-01 | 9.78E-01 | 0.23 | 2.74E-01 | 1.27E-03 | -0.05 | 4.10E-01 | 4.83E-01 |
| ENSG00000198053 | *SIRPA* | -0.01 | 4.61E-01 | 9.31E-01 | -0.58 | 2.24E-02 | **2.41E-18** | -0.38 | 1.09E-01 | **1.09E-07** | -0.28 | 1.75E-01 | 7.64E-05 | 0.16 | 3.54E-01 | 3.16E-02 | -0.30 | 1.66E-01 | 3.91E-05 |
| ENSG00000198121 | *LPAR1* | -0.56 | 2.68E-02 | **3.79E-17** | 0.17 | 3.44E-01 | 2.27E-02 | 0.29 | 2.25E-01 | 6.23E-05 | -0.44 | 7.13E-02 | **1.85E-10** | -0.11 | 3.46E-01 | 1.40E-01 | -0.08 | 3.75E-01 | 2.66E-01 |
| ENSG00000198542 | *ITGBL1* | 0.22 | 2.93E-01 | 2.95E-03 | -0.23 | 2.22E-01 | 1.54E-03 | -0.31 | 1.52E-01 | 1.15E-05 | -0.12 | 3.37E-01 | 1.11E-01 | -0.06 | 3.99E-01 | 4.12E-01 | -0.16 | 2.90E-01 | 2.81E-02 |
| ENSG00000198825 | *INPP5F* | -0.24 | 2.17E-01 | 1.12E-03 | 0.60 | 3.97E-02 | **7.20E-20** | 0.57 | 5.10E-02 | **2.02E-17** | 0.13 | 3.83E-01 | 7.62E-02 | -0.07 | 3.90E-01 | 3.50E-01 | 0.24 | 2.71E-01 | 1.04E-03 |
| ENSG00000198901 | *PRC1* | -0.20 | 2.49E-01 | 5.44E-03 | 0.28 | 2.31E-01 | 9.87E-05 | 0.08 | 4.38E-01 | 2.69E-01 | -0.04 | 4.18E-01 | 5.47E-01 | -0.08 | 3.72E-01 | 2.46E-01 | 0.01 | 5.17E-01 | 8.65E-01 |
| ENSG00000203865 | *ATP1A1-AS1* | 0.14 | 3.70E-01 | 5.25E-02 | 0.32 | 1.98E-01 | 8.12E-06 | 0.21 | 3.00E-01 | 4.15E-03 | 0.34 | 1.81E-01 | **1.76E-06** | 0.10 | 4.16E-01 | 1.69E-01 | 0.19 | 3.15E-01 | 7.70E-03 |
| ENSG00000205133 | *TRIQK* | -0.50 | 4.78E-02 | **3.52E-13** | 0.67 | 2.14E-02 | **2.94E-26** | 0.69 | 1.86E-02 | **9.02E-28** | -0.10 | 3.60E-01 | 1.94E-01 | -0.17 | 2.81E-01 | 2.02E-02 | 0.26 | 2.53E-01 | 3.77E-04 |
| ENSG00000224093 | *RP5-1033H22.2* | 0.15 | 3.58E-01 | 3.61E-02 | 0.24 | 2.65E-01 | 7.46E-04 | 0.39 | 1.46E-01 | **3.94E-08** | 0.34 | 1.81E-01 | **1.81E-06** | 0.12 | 3.91E-01 | 9.28E-02 | 0.26 | 2.50E-01 | 3.19E-04 |
| ENSG00000228716 | *DHFR* | -0.48 | 5.56E-02 | **3.98E-12** | 0.39 | 1.45E-01 | **3.48E-08** | 0.44 | 1.12E-01 | **2.68E-10** | -0.16 | 2.87E-01 | 2.55E-02 | -0.06 | 3.94E-01 | 3.76E-01 | 0.11 | 4.10E-01 | 1.49E-01 |
| ENSG00000230006 | *ANKRD36BP2* | 0.35 | 1.72E-01 | **7.17E-07** | 0.33 | 1.87E-01 | **3.26E-06** | -0.02 | 4.49E-01 | 8.20E-01 | 0.56 | 5.39E-02 | **6.67E-17** | -0.01 | 4.60E-01 | 9.19E-01 | 0.27 | 2.39E-01 | 1.64E-04 |
| ENSG00000233608 | *TWIST2* | 0.66 | 2.51E-02 | **1.49E-24** | -0.36 | 1.16E-01 | **2.70E-07** | -0.60 | 1.76E-02 | **5.52E-20** | 0.31 | 2.09E-01 | 2.01E-05 | 0.16 | 3.49E-01 | 2.72E-02 | -0.14 | 3.16E-01 | 6.28E-02 |
| ENSG00000235257 | *ITGA9-AS1* | -0.32 | 1.49E-01 | 8.99E-06 | 0.53 | 6.38E-02 | **2.65E-15** | 0.56 | 5.37E-02 | **6.21E-17** | 0.14 | 3.70E-01 | 5.33E-02 | 0.01 | 5.18E-01 | 8.73E-01 | 0.25 | 2.61E-01 | 6.10E-04 |
| ENSG00000235505 | *RP11-693N9.2* | -0.35 | 1.26E-01 | **8.95E-07** | 0.12 | 3.90E-01 | 9.13E-02 | 0.22 | 2.92E-01 | 2.92E-03 | -0.26 | 1.98E-01 | 3.65E-04 | 0.00 | 4.63E-01 | 9.51E-01 | 0.01 | 5.15E-01 | 8.46E-01 |
| ENSG00000237187 | *NR2F1-AS1* | 0.09 | 4.30E-01 | 2.30E-01 | 0.21 | 3.00E-01 | 4.09E-03 | 0.03 | 5.02E-01 | 7.28E-01 | 0.15 | 3.56E-01 | 3.44E-02 | 0.01 | 5.22E-01 | 9.11E-01 | 0.03 | 5.02E-01 | 7.26E-01 |
| ENSG00000246596 | *RP11-1277A3.2* | 0.17 | 3.44E-01 | 2.27E-02 | 0.23 | 2.75E-01 | 1.24E-03 | -0.04 | 4.27E-01 | 6.20E-01 | 0.32 | 2.00E-01 | 9.58E-06 | -0.04 | 4.27E-01 | 6.26E-01 | 0.09 | 4.31E-01 | 2.34E-01 |
| ENSG00000249464 | *LINC01091* | -0.09 | 3.68E-01 | 2.27E-01 | 0.51 | 7.67E-02 | **1.35E-13** | 0.40 | 1.37E-01 | **1.24E-08** | 0.14 | 3.75E-01 | 6.04E-02 | -0.15 | 3.01E-01 | 4.08E-02 | 0.22 | 2.92E-01 | 2.89E-03 |
| ENSG00000249992 | *TMEM158* | 0.59 | 4.40E-02 | **7.37E-19** | -0.51 | 4.49E-02 | **1.27E-13** | -0.63 | 1.26E-02 | **3.37E-22** | 0.22 | 2.85E-01 | 2.06E-03 | 0.12 | 3.99E-01 | 1.14E-01 | -0.15 | 3.04E-01 | 4.46E-02 |
| ENSG00000250722 | *SEPP1* | -0.62 | 1.48E-02 | **0.00E+00** | 0.54 | 6.35E-02 | **0.00E+00** | 0.55 | 5.71E-02 | **0.00E+00** | -0.30 | 1.63E-01 | 3.15E-05 | -0.19 | 2.63E-01 | 1.03E-02 | 0.09 | 4.32E-01 | 2.41E-01 |
| ENSG00000251504 | *LINC01099* | 0.05 | 4.73E-01 | 4.94E-01 | 0.38 | 1.52E-01 | **8.02E-08** | 0.30 | 2.18E-01 | 3.94E-05 | 0.25 | 2.61E-01 | 5.87E-04 | -0.06 | 3.96E-01 | 3.88E-01 | 0.20 | 3.09E-01 | 6.22E-03 |
| ENSG00000253669 | *KB-1732A1.1* | 0.00 | 5.29E-01 | 9.77E-01 | -0.25 | 2.00E-01 | 4.30E-04 | -0.16 | 2.92E-01 | 2.97E-02 | -0.15 | 3.00E-01 | 3.86E-02 | 0.01 | 5.19E-01 | 8.84E-01 | -0.06 | 3.98E-01 | 4.03E-01 |
| ENSG00000254615 | *RP11-395G23.3* | 0.42 | 1.21E-01 | **1.28E-09** | -0.39 | 9.81E-02 | **2.27E-08** | -0.48 | 5.43E-02 | **2.70E-12** | 0.02 | 5.04E-01 | 7.43E-01 | 0.10 | 4.14E-01 | 1.65E-01 | -0.15 | 3.02E-01 | 4.11E-02 |
| ENSG00000256802 | *RP11-680F8.1* | 0.24 | 2.69E-01 | 9.11E-04 | -0.39 | 1.01E-01 | **3.71E-08** | -0.35 | 1.24E-01 | **7.16E-07** | 0.05 | 4.76E-01 | 5.11E-01 | 0.10 | 4.12E-01 | 1.57E-01 | -0.06 | 4.03E-01 | 4.36E-01 |
| ENSG00000257261 | *RP11-96H19.1* | -0.49 | 5.23E-02 | **1.47E-12** | 0.38 | 1.55E-01 | **1.14E-07** | 0.44 | 1.09E-01 | **1.64E-10** | -0.23 | 2.20E-01 | 1.31E-03 | -0.10 | 3.60E-01 | 1.92E-01 | 0.07 | 4.56E-01 | 3.72E-01 |
| ENSG00000261713 | *SSTR5-AS1* | 0.76 | 8.49E-03 | **8.16E-37** | -0.13 | 3.22E-01 | 7.52E-02 | -0.33 | 1.41E-01 | 4.37E-06 | 0.57 | 5.04E-02 | **1.58E-17** | 0.15 | 3.66E-01 | 4.68E-02 | 0.06 | 4.59E-01 | 3.95E-01 |
| ENSG00000262655 | *SPON1* | -0.10 | 3.60E-01 | 1.92E-01 | -0.10 | 3.52E-01 | 1.63E-01 | -0.17 | 2.84E-01 | 2.25E-02 | -0.21 | 2.41E-01 | 3.86E-03 | -0.03 | 4.34E-01 | 6.88E-01 | -0.20 | 2.55E-01 | 7.30E-03 |
