# Supplementary material for: Islet Gene View—a tool to facilitate islet research
Source: Life Sci Alliance. 2022 Aug 10;5(12):e202201376. doi: 10.26508/lsa.202201376 (PMC9366203; doi:10.26508/lsa.202201376)
Supplement: Supplementary file 6 [file LSA-2022-01376_TableS6.docx]

Supplementary table 6. Cell enrichment of exocrine cells from Segelstolpe et al compared with DE genes correlated with purity.

| **genes** | **logFC_Purity** | **AveExpr_Purity** | **t_Purity** | **P.Value_Purity** | **adj.P.Val_Purity** | **B_Purity** | **HGNC symbol** | **Cell type enrichment from single cell data (Segelstolpe et al)** |
| --- | --- | --- | --- | --- | --- | --- | --- | --- |
| ENSG00000108187 | -0.01 | 3.79 | -3.55 | 4.92E-04 | 1.54E-03 | -2.45E+00 | PBLD | acinar cells |
| ENSG00000154274 | -0.02 | 4.53 | -8.01 | 1.18E-13 | 3.59E-12 | 1.90E+01 | C4orf19 | acinar cells |
| ENSG00000136997 | -0.01 | 5.07 | -5.31 | 3.03E-07 | 1.87E-06 | 4.45E+00 | MYC | acinar cells |
| ENSG00000017483 | -0.03 | 2.81 | -8.06 | 9.14E-14 | 2.91E-12 | 1.95E+01 | SLC38A5 | acinar cells |
| ENSG00000164078 | -0.03 | 2.69 | -6.79 | 1.48E-10 | 1.89E-09 | 1.22E+01 | MST1R | acinar cells |
| ENSG00000134363 | -0.03 | 1.16 | -7.83 | 3.68E-13 | 9.18E-12 | 1.84E+01 | FST | acinar cells |
| ENSG00000132386 | 0.00 | 4.00 | -1.07 | 2.85E-01 | 3.88E-01 | -8.03E+00 | SERPINF1 | acinar cells |
| ENSG00000153822 | -0.01 | 4.05 | -1.64 | 1.02E-01 | 1.66E-01 | -7.27E+00 | KCNJ16 | ductal cells |
| ENSG00000177459 | 0.00 | 4.55 | -2.43 | 1.62E-02 | 3.44E-02 | -5.77E+00 | ERICH5 | ductal cells |
| ENSG00000184640 | -0.01 | 6.85 | -4.78 | 3.55E-06 | 1.76E-05 | 1.95E+00 | SEPT9 | ductal cells |
| ENSG00000153551 | -0.02 | 3.94 | -6.99 | 4.74E-11 | 6.80E-10 | 1.32E+01 | CMTM7 | ductal cells |
| ENSG00000182022 | -0.01 | 4.68 | -5.07 | 9.61E-07 | 5.34E-06 | 3.39E+00 | CHST15 | ductal cells |
| ENSG00000198053 | -0.02 | 5.21 | -6.97 | 5.33E-11 | 7.57E-10 | 1.29E+01 | SIRPA | ductal cells |
| ENSG00000188042 | 0.00 | 5.88 | -1.30 | 1.94E-01 | 2.85E-01 | -7.98E+00 | ARL4C | ductal cells |
| ENSG00000133216 | -0.02 | 5.32 | -7.59 | 1.51E-12 | 3.18E-11 | 1.64E+01 | EPHB2 | ductal cells |
| ENSG00000137819 | -0.03 | 4.37 | -8.68 | 2.01E-15 | 1.09E-13 | 2.31E+01 | PAQR5 | ductal cells |
| ENSG00000188643 | -0.01 | 5.98 | -4.03 | 8.05E-05 | 3.01E-04 | -1.01E+00 | S100A16 | ductal cells |
| ENSG00000176014 | -0.01 | 5.54 | -3.94 | 1.17E-04 | 4.20E-04 | -1.32E+00 | TUBB6 | ductal cells |
| ENSG00000157765 | -0.03 | 5.38 | -7.62 | 1.25E-12 | 2.72E-11 | 1.66E+01 | SLC34A2 | ductal cells |
| ENSG00000109610 | -0.01 | 3.60 | -1.42 | 1.58E-01 | 2.40E-01 | -7.56E+00 | SOD3 | ductal cells |
| ENSG00000179431 | -0.01 | 2.54 | -2.10 | 3.72E-02 | 7.00E-02 | -6.20E+00 | FJX1 | ductal cells |
| ENSG00000144063 | -0.02 | 3.50 | -5.48 | 1.38E-07 | 9.15E-07 | 5.43E+00 | MALL | ductal cells |
| ENSG00000173918 | -0.01 | 2.21 | -3.51 | 5.54E-04 | 1.72E-03 | -2.35E+00 | C1QTNF1 | ductal cells |
| ENSG00000137440 | -0.02 | 3.71 | -3.65 | 3.36E-04 | 1.10E-03 | -2.11E+00 | FGFBP1 | ductal cells |
