# Supplementary material for: Islet Gene View—a tool to facilitate islet research
Source: Life Sci Alliance. 2022 Aug 10;5(12):e202201376. doi: 10.26508/lsa.202201376 (PMC9366203; doi:10.26508/lsa.202201376)
Supplement: Supplementary file 7 [file LSA-2022-01376_TableS7.docx]

Supplementary table 7. Look up of DEGs in Segerstolpe et al for expression in pancreatic stellate cells. Differential expression analysis between Pancreatic Stellate Cells (PSCs, n = 54) and endothelial cells (n = 16) showed the expression of 29 genes from the DEG list of genes to be higher in pancreatic stellate cells whereas 4 genes were significantly upregulated in endothelial cells. Genes with corrected abs(Z-score)>=1.96 were further ranked in descending order by the expression fold change (mle, which equalled log2FC). Positive signs of the third column (mle) indicate that the gene was up-regulated in PSCs, whereas a negative sign corresponds to genes up-regulated in endothelial cells.

| Definition of columns (according to the documentation in the SCDE package): | | |
| --- | --- | --- |
| 1 | gene ID | gene RefSeq id |
| 2 | lb | lower bound of the 95 interval for the expression fold change on log_2_ scale |
| 3 | mle | maximum likelihood estimate of the 95 interval for the expression fold change on log_2_ scale |
| 4 | ub | upper bound of the 95 interval for the expression fold change on log_2_ scale |
| 5 | ce | conservative estimate of the expression-fold change |
| 6 | Z | uncorrected Z-score of expression difference |
| 7 | cZ | expression fold difference corrected for multiple hypothesis testing using BH procedure |

| **rank** | **geneID** | **lb** | **mle** | **ub** | **ce** | **Z** | **cZ** |
| --- | --- | --- | --- | --- | --- | --- | --- |
| 7 | THBS2 | 7.616 | 10.243 | 12.376 | 7.616 | 7.145 | 6.346 |
| 8 | DCN | 7.551 | 10.144 | 12.278 | 7.551 | 7.145 | 6.346 |
| 23 | SERPINE2 | 5.482 | 8.273 | 10.472 | 5.482 | 7.064 | 6.298 |
| 25 | IL11 | 4.957 | 8.010 | 10.341 | 4.957 | 5.974 | 5.156 |
| 35 | SERPINF1 | 5.088 | 7.649 | 10.013 | 5.088 | 6.828 | 6.052 |
| 43 | COMP | 4.268 | 7.288 | 9.553 | 4.268 | 5.732 | 4.897 |
| 56 | PTGDS | 3.349 | 6.796 | 9.783 | 3.349 | 4.728 | 3.819 |
| 66 | ITGA11 | 3.874 | 6.467 | 8.568 | 3.874 | 5.735 | 4.899 |
| 76 | MFAP4 | 3.447 | 6.270 | 8.503 | 3.447 | 4.677 | 3.765 |
| 80 | SPON1 | 3.644 | 6.139 | 8.306 | 3.644 | 5.636 | 4.795 |
| 98 | IL6 | 2.200 | 5.876 | 8.437 | 2.200 | 3.399 | 2.342 |
| 101 | LIF | 2.922 | 5.745 | 7.912 | 2.922 | 4.392 | 3.454 |
| 114 | PDGFRA | 2.659 | 5.450 | 7.616 | 2.659 | 4.350 | 3.411 |
| 137 | FBLN1 | 2.495 | 5.154 | 7.222 | 2.495 | 4.227 | 3.276 |
| 142 | PTGES | 2.167 | 5.056 | 7.255 | 2.167 | 3.828 | 2.822 |
| 180 | PDE1A | 2.003 | 4.629 | 6.730 | 2.003 | 3.844 | 2.841 |
| 215 | PIEZO2 | 1.871 | 4.333 | 6.467 | 1.871 | 3.709 | 2.687 |
| 222 | C7 | 1.346 | 4.301 | 6.533 | 1.346 | 3.068 | 1.973 |
| 233 | SCD | 2.134 | 4.169 | 6.073 | 2.134 | 4.275 | 3.326 |
| 237 | NR2F1-AS1 | 1.444 | 4.136 | 6.237 | 1.444 | 3.266 | 2.190 |
| 242 | SOD3 | 1.477 | 4.104 | 6.270 | 1.477 | 3.258 | 2.181 |
| 254 | PODN | 1.444 | 4.005 | 6.139 | 1.444 | 3.307 | 2.236 |
| 256 | ITGBL1 | 1.346 | 4.005 | 6.139 | 1.346 | 3.142 | 2.051 |
| 286 | DKK3 | 1.970 | 3.710 | 5.679 | 1.970 | 4.416 | 3.478 |
| 303 | IL1R1 | 1.510 | 3.480 | 5.417 | 1.510 | 3.543 | 2.501 |
| 308 | MYC | 1.576 | 3.381 | 5.450 | 1.576 | 3.745 | 2.727 |
| 323 | AIFM2 | 1.313 | 3.217 | 5.384 | 1.313 | 3.378 | 2.317 |
| 349 | ARL4C | 1.116 | 2.955 | 5.253 | 1.116 | 3.174 | 2.089 |
| 406 | ATP6V1A | 0.854 | 2.396 | 4.366 | 0.854 | 3.083 | 1.989 |
| 471 | BTBD3 | -4.727 | -2.955 | -1.149 | -1.149 | -3.313 | -2.242 |
| 500 | SRGN | -4.990 | -3.217 | -1.740 | -1.740 | -4.500 | -3.571 |
| 502 | GNG7 | -5.154 | -3.250 | -1.543 | -1.543 | -3.509 | -2.463 |
| 576 | SERPINB9 | -5.909 | -4.071 | -2.364 | -2.364 | -4.674 | -3.763 |
