# Supplementary material for: Islet Gene View—a tool to facilitate islet research
Source: Life Sci Alliance. 2022 Aug 10;5(12):e202201376. doi: 10.26508/lsa.202201376 (PMC9366203; doi:10.26508/lsa.202201376)
Supplement: Supplementary file 8 [file LSA-2022-01376_TableS8.docx]

Supplementary table 8. List of lead eQTLs per gene for the differentially expressed genes (T2D v non-T2D from Supplementary table 2) in islets (gene level quantifications).

| **Gene ID** | **HGNC symbol** | **Rsid_SNP** | **Position_SNP** | **Beta** | **T** | **P** | **FDR** |
| --- | --- | --- | --- | --- | --- | --- | --- |
| ENSG00000251504 | *LINC01099* | rs1676165 | 4:178948305 | -0.69 | -6.76 | 1.69E-10 | 3.33E-07 |
| ENSG00000251504 | *LINC01099* | rs1711382 | 4:178947569 | -0.69 | -6.75 | 1.78E-10 | 3.49E-07 |
| ENSG00000151470 | *C4orf33* | rs3113489 | 4:129961179 | -0.74 | -6.71 | 2.22E-10 | 4.30E-07 |
| ENSG00000251504 | *LINC01099* | rs11727108 | 4:178909910 | -0.67 | -6.71 | 2.26E-10 | 4.37E-07 |
| ENSG00000251504 | *LINC01099* | rs10006596 | 4:178910307 | -0.67 | -6.71 | 2.27E-10 | 4.38E-07 |
| ENSG00000251504 | *LINC01099* | rs1616320 | 4:178944090 | -0.68 | -6.70 | 2.46E-10 | 4.73E-07 |
| ENSG00000251504 | *LINC01099* | rs1676204 | 4:178935073 | -0.68 | -6.67 | 2.85E-10 | 5.41E-07 |
| ENSG00000251504 | *LINC01099* | rs28676428 | 4:178903141 | -0.67 | -6.66 | 2.96E-10 | 5.61E-07 |
| ENSG00000251504 | *LINC01099* | rs1676163 | 4:178945059 | -0.68 | -6.63 | 3.44E-10 | 6.45E-07 |
| ENSG00000251504 | *LINC01099* | rs35337595 | 4:178931611 | -0.67 | -6.63 | 3.45E-10 | 6.46E-07 |
| ENSG00000251504 | *LINC01099* | rs4690589 | 4:178908200 | -0.66 | -6.63 | 3.61E-10 | 6.74E-07 |
| ENSG00000251504 | *LINC01099* | rs4690588 | 4:178907903 | -0.66 | -6.63 | 3.62E-10 | 6.76E-07 |
| ENSG00000251504 | *LINC01099* | rs34496992 | 4:178911339 | -0.66 | -6.62 | 3.64E-10 | 6.79E-07 |
| ENSG00000251504 | *LINC01099* | rs4521292 | 4:178928693 | -0.67 | -6.62 | 3.75E-10 | 6.99E-07 |
| ENSG00000251504 | *LINC01099* | rs62340139 | 4:178903608 | -0.66 | -6.61 | 3.89E-10 | 7.23E-07 |
| ENSG00000251504 | *LINC01099* | rs11735257 | 4:178948866 | -0.68 | -6.61 | 3.93E-10 | 7.30E-07 |
| ENSG00000251504 | *LINC01099* | rs6818024 | 4:178912494 | -0.65 | -6.57 | 4.77E-10 | 8.73E-07 |
| ENSG00000251504 | *LINC01099* | rs34323828 | 4:178916487 | -0.67 | -6.57 | 4.92E-10 | 8.98E-07 |
| ENSG00000251504 | *LINC01099* | rs2378809 | 4:178928832 | -0.66 | -6.56 | 5.26E-10 | 9.56E-07 |
| ENSG00000251504 | *LINC01099* | rs2054381 | 4:178944245 | -0.67 | -6.56 | 5.29E-10 | 9.60E-07 |
| ENSG00000251504 | *LINC01099* | rs2054380 | 4:178943989 | -0.67 | -6.55 | 5.34E-10 | 9.70E-07 |
| ENSG00000251504 | *LINC01099* | rs35469245 | 4:178936479 | -0.66 | -6.53 | 6.09E-10 | 1.10E-06 |
| ENSG00000251504 | *LINC01099* | rs1200380479 | 4:178937436 | -0.66 | -6.52 | 6.28E-10 | 1.13E-06 |
| ENSG00000251504 | *LINC01099* | rs13127448 | 4:178937182 | -0.66 | -6.52 | 6.29E-10 | 1.13E-06 |
| ENSG00000251504 | *LINC01099* | rs13126982 | 4:178936860 | -0.66 | -6.52 | 6.38E-10 | 1.14E-06 |
| ENSG00000251504 | *LINC01099* | rs13120885 | 4:178936431 | -0.66 | -6.52 | 6.44E-10 | 1.16E-06 |
| ENSG00000251504 | *LINC01099* | rs4690591 | 4:178917059 | -0.67 | -6.52 | 6.46E-10 | 1.16E-06 |
| ENSG00000251504 | *LINC01099* | rs4143150 | 4:178934649 | -0.66 | -6.51 | 6.71E-10 | 1.20E-06 |
| ENSG00000251504 | *LINC01099* | rs13104862 | 4:178933815 | -0.66 | -6.51 | 6.85E-10 | 1.22E-06 |
| ENSG00000251504 | *LINC01099* | rs75677930 | 4:178935924 | -0.71 | -6.50 | 7.03E-10 | 1.25E-06 |
| ENSG00000251504 | *LINC01099* | rs35934543 | 4:178914462 | -0.65 | -6.50 | 7.24E-10 | 1.29E-06 |
| ENSG00000251504 | *LINC01099* | rs11733799 | 4:178930079 | -0.66 | -6.49 | 7.50E-10 | 1.33E-06 |
| ENSG00000251504 | *LINC01099* | rs13143474 | 4:178926789 | -0.65 | -6.47 | 8.33E-10 | 1.47E-06 |
| ENSG00000251504 | *LINC01099* | rs34861677 | 4:178925837 | -0.65 | -6.47 | 8.64E-10 | 1.52E-06 |
| ENSG00000251504 | *LINC01099* | rs34770190 | 4:178925777 | -0.65 | -6.47 | 8.66E-10 | 1.52E-06 |
| ENSG00000251504 | *LINC01099* | rs147331430 | 4:178920812 | -0.65 | -6.46 | 8.73E-10 | 1.53E-06 |
| ENSG00000251504 | *LINC01099* | rs36098045 | 4:178925406 | -0.65 | -6.45 | 9.18E-10 | 1.60E-06 |
| ENSG00000251504 | *LINC01099* | rs6837651 | 4:178928296 | -0.65 | -6.45 | 9.51E-10 | 1.65E-06 |
| ENSG00000251504 | *LINC01099* | rs13150515 | 4:178927692 | -0.65 | -6.44 | 9.71E-10 | 1.69E-06 |
| ENSG00000251504 | *LINC01099* | rs4690593 | 4:178926483 | -0.65 | -6.44 | 1.01E-09 | 1.75E-06 |
| ENSG00000251504 | *LINC01099* | rs4690594 | 4:178926583 | -0.65 | -6.44 | 1.01E-09 | 1.76E-06 |
| ENSG00000251504 | *LINC01099* | rs1599606 | 4:178926190 | -0.65 | -6.43 | 1.05E-09 | 1.80E-06 |
| ENSG00000251504 | *LINC01099* | rs1462516 | 4:178923095 | -0.64 | -6.41 | 1.16E-09 | 1.98E-06 |
| ENSG00000251504 | *LINC01099* | rs1462514 | 4:178922921 | -0.64 | -6.41 | 1.16E-09 | 1.99E-06 |
| ENSG00000251504 | *LINC01099* | rs11452069 | 4:178922709 | -0.64 | -6.41 | 1.17E-09 | 2.00E-06 |
| ENSG00000251504 | *LINC01099* | rs4690400 | 4:178913816 | -0.64 | -6.37 | 1.42E-09 | 2.35E-06 |
| ENSG00000136108 | *CKAP2* | rs1355322882 | 13:53040041 | -0.59 | -6.35 | 1.58E-09 | 2.59E-06 |
| ENSG00000251504 | *LINC01099* | rs74567288 | 4:178935926 | -0.68 | -6.30 | 2.12E-09 | 3.38E-06 |
| ENSG00000251504 | *LINC01099* | rs141226782 | 4:178926130 | -0.65 | -6.28 | 2.32E-09 | 3.68E-06 |
| ENSG00000251504 | *LINC01099* | rs1423848195 | 4:178926129 | -0.65 | -6.28 | 2.32E-09 | 3.68E-06 |
| ENSG00000228716 | *DHFR* | rs201268703 | 5:79912885 | -0.62 | -6.21 | 3.37E-09 | 5.09E-06 |
| ENSG00000151470 | *C4orf33* | rs4394010 | 4:129894649 | 0.76 | 6.15 | 4.64E-09 | 6.82E-06 |
| ENSG00000151470 | *C4orf33* | rs390260 | 4:130036591 | -0.75 | -6.15 | 4.75E-09 | 6.96E-06 |
| ENSG00000151470 | *C4orf33* | rs487233 | 4:130036142 | -0.75 | -6.15 | 4.75E-09 | 6.96E-06 |
| ENSG00000151470 | *C4orf33* | rs389376 | 4:130036179 | -0.75 | -6.15 | 4.75E-09 | 6.96E-06 |
| ENSG00000151470 | *C4orf33* | rs487501 | 4:130036191 | -0.75 | -6.15 | 4.75E-09 | 6.96E-06 |
| ENSG00000151470 | *C4orf33* | rs445294 | 4:130035889 | -0.75 | -6.15 | 4.75E-09 | 6.96E-06 |
| ENSG00000151470 | *C4orf33* | rs426521 | 4:130035755 | -0.75 | -6.15 | 4.75E-09 | 6.96E-06 |
| ENSG00000151470 | *C4orf33* | rs189235 | 4:130035370 | -0.75 | -6.15 | 4.75E-09 | 6.96E-06 |
| ENSG00000151470 | *C4orf33* | rs1699380 | 4:130034581 | -0.75 | -6.15 | 4.75E-09 | 6.96E-06 |
| ENSG00000151470 | *C4orf33* | rs391396 | 4:130037653 | -0.75 | -6.15 | 4.75E-09 | 6.96E-06 |
| ENSG00000151470 | *C4orf33* | rs474794 | 4:130038674 | -0.75 | -6.15 | 4.75E-09 | 6.96E-06 |
| ENSG00000151470 | *C4orf33* | rs337277 | 4:130030652 | -0.75 | -6.15 | 4.75E-09 | 6.96E-06 |
| ENSG00000151470 | *C4orf33* | rs1709419 | 4:130038913 | -0.75 | -6.15 | 4.75E-09 | 6.96E-06 |
| ENSG00000151470 | *C4orf33* | rs337270 | 4:130042232 | -0.75 | -6.15 | 4.75E-09 | 6.96E-06 |
| ENSG00000151470 | *C4orf33* | rs517659 | 4:130045321 | -0.75 | -6.15 | 4.75E-09 | 6.96E-06 |
| ENSG00000151470 | *C4orf33* | rs395864 | 4:130050091 | -0.75 | -6.15 | 4.75E-09 | 6.96E-06 |
| ENSG00000151470 | *C4orf33* | rs337275 | 4:130054469 | -0.75 | -6.15 | 4.75E-09 | 6.96E-06 |
| ENSG00000151470 | *C4orf33* | rs337274 | 4:130056680 | -0.75 | -6.15 | 4.75E-09 | 6.96E-06 |
| ENSG00000151470 | *C4orf33* | rs1757923 | 4:130058670 | -0.75 | -6.15 | 4.75E-09 | 6.96E-06 |
| ENSG00000151470 | *C4orf33* | rs1699387 | 4:130031498 | -0.75 | -6.15 | 4.75E-09 | 6.96E-06 |
| ENSG00000151470 | *C4orf33* | rs474667 | 4:130038714 | -0.75 | -6.15 | 4.75E-09 | 6.96E-06 |
| ENSG00000151470 | *C4orf33* | rs1709421 | 4:130058979 | -0.75 | -6.14 | 4.78E-09 | 7.01E-06 |
| ENSG00000151470 | *C4orf33* | rs57501568 | 4:129888787 | 0.76 | 6.11 | 5.79E-09 | 8.37E-06 |
| ENSG00000151470 | *C4orf33* | rs337278 | 4:130029879 | -0.75 | -6.10 | 6.15E-09 | 8.85E-06 |
| ENSG00000251504 | *LINC01099* | rs1711390 | 4:178933641 | -0.58 | -6.08 | 6.57E-09 | 9.41E-06 |
| ENSG00000136108 | *CKAP2* | rs9568732 | 13:52993412 | -0.55 | -6.07 | 6.99E-09 | 9.95E-06 |
| ENSG00000151470 | *C4orf33* | rs200575504 | 4:130024726 | -0.76 | -6.07 | 7.00E-09 | 9.96E-06 |
| ENSG00000251504 | *LINC01099* | rs2889996 | 4:178929121 | -0.58 | -6.07 | 7.03E-09 | 1.00E-05 |
| ENSG00000251504 | *LINC01099* | rs2378808 | 4:178922743 | -0.56 | -6.05 | 7.61E-09 | 1.07E-05 |
| ENSG00000251504 | *LINC01099* | rs1564086 | 4:178924933 | -0.56 | -6.05 | 7.73E-09 | 1.09E-05 |
| ENSG00000251504 | *LINC01099* | rs11947286 | 4:178923759 | -0.56 | -6.05 | 7.94E-09 | 1.11E-05 |
| ENSG00000151470 | *C4orf33* | rs201427408 | 4:130024725 | -0.75 | -6.05 | 7.94E-09 | 1.12E-05 |
| ENSG00000136108 | *CKAP2* | rs58530254 | 13:53043731 | -0.58 | -6.04 | 8.07E-09 | 1.13E-05 |
| ENSG00000251504 | *LINC01099* | rs1462513 | 4:178915050 | -0.56 | -6.04 | 8.17E-09 | 1.14E-05 |
| ENSG00000251504 | *LINC01099* | rs2126505 | 4:178949353 | -0.66 | -6.04 | 8.17E-09 | 1.14E-05 |
| ENSG00000151470 | *C4orf33* | rs337263 | 4:130033665 | -0.75 | -6.02 | 9.18E-09 | 1.27E-05 |
| ENSG00000251504 | *LINC01099* | rs1711383 | 4:178944653 | -0.56 | -6.01 | 9.38E-09 | 1.30E-05 |
| ENSG00000136108 | *CKAP2* | rs7990581 | 13:53019433 | -0.55 | -6.01 | 9.45E-09 | 1.31E-05 |
| ENSG00000151470 | *C4orf33* | rs1699384 | 4:129987071 | -0.74 | -6.01 | 9.70E-09 | 1.34E-05 |
| ENSG00000251504 | *LINC01099* | rs11726935 | 4:178948678 | -0.55 | -6.00 | 9.98E-09 | 1.37E-05 |
| ENSG00000136108 | *CKAP2* | rs11148252 | 13:53009048 | -0.57 | -5.98 | 1.10E-08 | 1.50E-05 |
| ENSG00000136108 | *CKAP2* | rs4886018 | 13:52990717 | -0.57 | -5.98 | 1.10E-08 | 1.50E-05 |
| ENSG00000151470 | *C4orf33* | rs13130762 | 4:130022875 | -0.73 | -5.98 | 1.11E-08 | 1.51E-05 |
| ENSG00000151470 | *C4orf33* | rs1699391 | 4:130005096 | -0.73 | -5.98 | 1.13E-08 | 1.53E-05 |
| ENSG00000151470 | *C4orf33* | rs1699394 | 4:130019312 | -0.73 | -5.98 | 1.14E-08 | 1.54E-05 |
| ENSG00000151470 | *C4orf33* | rs1037147 | 4:130025873 | -0.73 | -5.98 | 1.14E-08 | 1.54E-05 |
| ENSG00000151470 | *C4orf33* | rs2655310 | 4:130017919 | -0.73 | -5.98 | 1.14E-08 | 1.55E-05 |
| ENSG00000151470 | *C4orf33* | rs1756012 | 4:130018971 | -0.73 | -5.98 | 1.14E-08 | 1.55E-05 |
| ENSG00000151470 | *C4orf33* | rs1699393 | 4:130019352 | -0.73 | -5.98 | 1.14E-08 | 1.55E-05 |
| ENSG00000151470 | *C4orf33* | rs199741783 | 4:130020042 | -0.73 | -5.98 | 1.14E-08 | 1.55E-05 |
| ENSG00000151470 | *C4orf33* | rs1757925 | 4:130020791 | -0.73 | -5.98 | 1.15E-08 | 1.55E-05 |
| ENSG00000151470 | *C4orf33* | rs1757927 | 4:130022069 | -0.73 | -5.98 | 1.15E-08 | 1.55E-05 |
| ENSG00000151470 | *C4orf33* | rs1757928 | 4:130022161 | -0.73 | -5.98 | 1.15E-08 | 1.55E-05 |
| ENSG00000228716 | *DHFR* | rs1461057472 | 5:79912895 | -0.61 | -5.98 | 1.15E-08 | 1.55E-05 |
| ENSG00000151470 | *C4orf33* | rs1699392 | 4:130022347 | -0.73 | -5.98 | 1.15E-08 | 1.55E-05 |
| ENSG00000151470 | *C4orf33* | rs3105369 | 4:130022448 | -0.73 | -5.98 | 1.15E-08 | 1.55E-05 |
| ENSG00000151470 | *C4orf33* | rs1757930 | 4:130022356 | -0.73 | -5.98 | 1.15E-08 | 1.55E-05 |
| ENSG00000151470 | *C4orf33* | rs1757935 | 4:130023759 | -0.73 | -5.98 | 1.15E-08 | 1.56E-05 |
| ENSG00000151470 | *C4orf33* | rs1030831 | 4:130025037 | -0.73 | -5.97 | 1.15E-08 | 1.56E-05 |
| ENSG00000251504 | *LINC01099* | rs11933040 | 4:178936076 | -0.57 | -5.97 | 1.19E-08 | 1.59E-05 |
| ENSG00000151470 | *C4orf33* | rs1757924 | 4:130019599 | -0.73 | -5.96 | 1.21E-08 | 1.61E-05 |
| ENSG00000136108 | *CKAP2* | rs9568734 | 13:53004222 | -0.54 | -5.95 | 1.34E-08 | 1.76E-05 |
| ENSG00000151470 | *C4orf33* | rs1699379 | 4:129992337 | -0.73 | -5.93 | 1.46E-08 | 1.90E-05 |
| ENSG00000151470 | *C4orf33* | rs1699385 | 4:129990164 | -0.73 | -5.93 | 1.46E-08 | 1.90E-05 |
| ENSG00000151470 | *C4orf33* | rs1699389 | 4:130004079 | -0.73 | -5.93 | 1.46E-08 | 1.90E-05 |
| ENSG00000151470 | *C4orf33* | rs58689608 | 4:129991464 | -0.73 | -5.93 | 1.46E-08 | 1.90E-05 |
| ENSG00000151470 | *C4orf33* | rs1699388 | 4:130003237 | -0.73 | -5.93 | 1.46E-08 | 1.90E-05 |
| ENSG00000151470 | *C4orf33* | rs1374491 | 4:129968621 | -0.73 | -5.93 | 1.46E-08 | 1.91E-05 |
| ENSG00000151470 | *C4orf33* | rs2083634 | 4:129976478 | -0.73 | -5.93 | 1.47E-08 | 1.91E-05 |
| ENSG00000151470 | *C4orf33* | rs13109003 | 4:129975969 | -0.73 | -5.93 | 1.47E-08 | 1.91E-05 |
| ENSG00000151470 | *C4orf33* | rs4975193 | 4:129980630 | -0.73 | -5.93 | 1.47E-08 | 1.91E-05 |
| ENSG00000151470 | *C4orf33* | rs4975194 | 4:129982377 | -0.73 | -5.93 | 1.47E-08 | 1.91E-05 |
| ENSG00000151470 | *C4orf33* | rs3099899 | 4:129917368 | -0.73 | -5.92 | 1.51E-08 | 1.96E-05 |
| ENSG00000151470 | *C4orf33* | rs4466058 | 4:129923879 | -0.73 | -5.92 | 1.51E-08 | 1.96E-05 |
| ENSG00000136108 | *CKAP2* | rs3892337 | 13:53029485 | -0.54 | -5.92 | 1.56E-08 | 2.01E-05 |
| ENSG00000136108 | *CKAP2* | rs9536079 | 13:53030565 | -0.54 | -5.91 | 1.57E-08 | 2.03E-05 |
| ENSG00000136108 | *CKAP2* | rs7321964 | 13:53040822 | -0.54 | -5.91 | 1.57E-08 | 2.03E-05 |
| ENSG00000151470 | *C4orf33* | rs66921209 | 4:129868556 | 0.73 | 5.90 | 1.67E-08 | 2.14E-05 |
| ENSG00000228716 | *DHFR* | rs6453527 | 5:80139372 | 0.61 | 5.89 | 1.79E-08 | 2.28E-05 |
| ENSG00000251504 | *LINC01099* | rs11721689 | 4:178930142 | -0.56 | -5.89 | 1.79E-08 | 2.28E-05 |
| ENSG00000136108 | *CKAP2* | rs11620062 | 13:52994026 | -0.56 | -5.88 | 1.89E-08 | 2.40E-05 |
| ENSG00000136108 | *CKAP2* | rs7328653 | 13:53032735 | -0.54 | -5.88 | 1.90E-08 | 2.41E-05 |
| ENSG00000151470 | *C4orf33* | rs1002969776 | 4:129988405 | -0.73 | -5.87 | 1.95E-08 | 2.47E-05 |
| ENSG00000151470 | *C4orf33* | rs280604 | 4:129921170 | -0.71 | -5.86 | 2.02E-08 | 2.55E-05 |
| ENSG00000151470 | *C4orf33* | rs10002332 | 4:129875301 | 0.72 | 5.86 | 2.06E-08 | 2.59E-05 |
| ENSG00000151470 | *C4orf33* | rs12171333 | 4:129879711 | 0.72 | 5.86 | 2.06E-08 | 2.60E-05 |
| ENSG00000151470 | *C4orf33* | rs35006492 | 4:129878342 | 0.72 | 5.86 | 2.07E-08 | 2.60E-05 |
| ENSG00000151470 | *C4orf33* | rs2198042 | 4:129885499 | 0.72 | 5.85 | 2.12E-08 | 2.67E-05 |
| ENSG00000151470 | *C4orf33* | rs7655841 | 4:129890790 | 0.72 | 5.85 | 2.13E-08 | 2.67E-05 |
| ENSG00000151470 | *C4orf33* | rs397995376 | 4:129911111 | -0.72 | -5.85 | 2.20E-08 | 2.75E-05 |
| ENSG00000151470 | *C4orf33* | rs280603 | 4:129915063 | -0.72 | -5.85 | 2.22E-08 | 2.77E-05 |
| ENSG00000151470 | *C4orf33* | rs1041359014 | 4:130024727 | -0.72 | -5.83 | 2.47E-08 | 3.05E-05 |
| ENSG00000251504 | *LINC01099* | rs141897901 | 4:178941582 | -0.61 | -5.82 | 2.50E-08 | 3.10E-05 |
| ENSG00000136108 | *CKAP2* | rs1173916816 | 13:53043727 | -0.69 | -5.80 | 2.86E-08 | 3.49E-05 |
| ENSG00000136108 | *CKAP2* | rs9526927 | 13:53051627 | -0.54 | -5.79 | 2.91E-08 | 3.55E-05 |
| ENSG00000257261 | *RP11-96H19.1* | rs1443358762 | 12:46844823 | 0.68 | 5.78 | 3.05E-08 | 3.70E-05 |
| ENSG00000169071 | *ROR2* | rs9774945 | 9:94710847 | 0.56 | 5.77 | 3.33E-08 | 4.00E-05 |
| ENSG00000169071 | *ROR2* | rs11464558 | 9:94678627 | 0.58 | 5.76 | 3.37E-08 | 4.05E-05 |
| ENSG00000169071 | *ROR2* | rs4595185 | 9:94516377 | -0.57 | -5.76 | 3.45E-08 | 4.14E-05 |
| ENSG00000169071 | *ROR2* | rs11278540 | 9:94635486 | 0.57 | 5.75 | 3.66E-08 | 4.37E-05 |
| ENSG00000169071 | *ROR2* | rs10992159 | 9:94677777 | 0.57 | 5.74 | 3.74E-08 | 4.45E-05 |
| ENSG00000169071 | *ROR2* | rs4275276 | 9:94662604 | 0.57 | 5.73 | 3.92E-08 | 4.65E-05 |
| ENSG00000169071 | *ROR2* | rs4430151 | 9:94662474 | 0.57 | 5.73 | 3.92E-08 | 4.65E-05 |
| ENSG00000169071 | *ROR2* | rs62565679 | 9:94662092 | 0.57 | 5.73 | 3.93E-08 | 4.65E-05 |
| ENSG00000169071 | *ROR2* | rs6479383 | 9:94679355 | 0.57 | 5.73 | 4.05E-08 | 4.78E-05 |
| ENSG00000136108 | *CKAP2* | rs4286007 | 13:53022518 | -0.54 | -5.72 | 4.25E-08 | 5.01E-05 |
| ENSG00000169071 | *ROR2* | rs1881391 | 9:94692698 | 0.55 | 5.71 | 4.39E-08 | 5.16E-05 |
| ENSG00000136108 | *CKAP2* | rs11618716 | 13:53046836 | -0.54 | -5.71 | 4.46E-08 | 5.23E-05 |
| ENSG00000169071 | *ROR2* | rs186792620 | 9:94671199 | 0.56 | 5.70 | 4.53E-08 | 5.30E-05 |
| ENSG00000169071 | *ROR2* | rs10659451 | 9:94671200 | 0.56 | 5.70 | 4.53E-08 | 5.30E-05 |
| ENSG00000228716 | *DHFR* | rs372069 | 5:79931007 | -0.54 | -5.70 | 4.56E-08 | 5.33E-05 |
| ENSG00000228716 | *DHFR* | rs525054 | 5:79932099 | -0.54 | -5.70 | 4.56E-08 | 5.34E-05 |
| ENSG00000151470 | *C4orf33* | rs2597836 | 4:129904130 | -0.70 | -5.70 | 4.62E-08 | 5.40E-05 |
| ENSG00000228716 | *DHFR* | rs245379 | 5:80131628 | 0.60 | 5.70 | 4.62E-08 | 5.41E-05 |
| ENSG00000169071 | *ROR2* | rs6479387 | 9:94695011 | 0.54 | 5.70 | 4.64E-08 | 5.42E-05 |
| ENSG00000228716 | *DHFR* | rs2434329 | 5:79944196 | -0.54 | -5.70 | 4.66E-08 | 5.44E-05 |
| ENSG00000228716 | *DHFR* | rs861365 | 5:79915807 | -0.54 | -5.70 | 4.66E-08 | 5.44E-05 |
| ENSG00000228716 | *DHFR* | rs384925 | 5:79914633 | -0.54 | -5.70 | 4.66E-08 | 5.45E-05 |
| ENSG00000228716 | *DHFR* | rs1105524 | 5:79950512 | -0.54 | -5.70 | 4.67E-08 | 5.45E-05 |
| ENSG00000151470 | *C4orf33* | rs1854773 | 4:130008092 | -0.69 | -5.70 | 4.67E-08 | 5.45E-05 |
| ENSG00000228716 | *DHFR* | rs34359040 | 5:79942316 | -0.54 | -5.70 | 4.67E-08 | 5.46E-05 |
| ENSG00000151470 | *C4orf33* | rs2034498 | 4:129892905 | 0.71 | 5.70 | 4.73E-08 | 5.52E-05 |
| ENSG00000169071 | *ROR2* | rs10124760 | 9:94691555 | 0.54 | 5.69 | 4.84E-08 | 5.63E-05 |
| ENSG00000251504 | *LINC01099* | rs62339460 | 4:178951641 | -0.62 | -5.69 | 4.89E-08 | 5.69E-05 |
| ENSG00000251504 | *LINC01099* | rs1466411 | 4:178950814 | -0.62 | -5.69 | 4.98E-08 | 5.78E-05 |
| ENSG00000251504 | *LINC01099* | rs1466412 | 4:178950615 | -0.62 | -5.68 | 5.00E-08 | 5.80E-05 |
| ENSG00000251504 | *LINC01099* | rs11721667 | 4:178969298 | -0.62 | -5.68 | 5.14E-08 | 5.95E-05 |
| ENSG00000251504 | *LINC01099* | rs192922451 | 4:178939747 | -0.60 | -5.68 | 5.17E-08 | 5.98E-05 |
| ENSG00000169071 | *ROR2* | rs7850309 | 9:94711649 | 0.56 | 5.68 | 5.24E-08 | 6.06E-05 |
| ENSG00000136108 | *CKAP2* | rs201579381 | 13:52909775 | 0.53 | 5.67 | 5.40E-08 | 6.23E-05 |
| ENSG00000151470 | *C4orf33* | rs576492 | 4:130045592 | -0.70 | -5.66 | 5.63E-08 | 6.47E-05 |
| ENSG00000251504 | *LINC01099* | rs966108 | 4:178969993 | -0.62 | -5.66 | 5.65E-08 | 6.49E-05 |
| ENSG00000251504 | *LINC01099* | rs7681806 | 4:178968678 | -0.62 | -5.66 | 5.69E-08 | 6.53E-05 |
| ENSG00000251504 | *LINC01099* | rs77510205 | 4:178968505 | -0.62 | -5.66 | 5.70E-08 | 6.54E-05 |
| ENSG00000251504 | *LINC01099* | rs140587707 | 4:178966372 | -0.62 | -5.66 | 5.77E-08 | 6.62E-05 |
| ENSG00000251504 | *LINC01099* | rs10520400 | 4:178966254 | -0.62 | -5.66 | 5.78E-08 | 6.62E-05 |
| ENSG00000251504 | *LINC01099* | rs11733691 | 4:178959777 | -0.62 | -5.65 | 5.98E-08 | 6.84E-05 |
| ENSG00000251504 | *LINC01099* | rs62339517 | 4:178962042 | -0.62 | -5.65 | 5.98E-08 | 6.84E-05 |
| ENSG00000251504 | *LINC01099* | rs10564854 | 4:178964605 | -0.62 | -5.64 | 6.38E-08 | 7.26E-05 |
| ENSG00000228716 | *DHFR* | rs1643665 | 5:79910843 | -0.54 | -5.63 | 6.43E-08 | 7.30E-05 |
| ENSG00000228716 | *DHFR* | rs147163404 | 5:80130457 | 0.58 | 5.63 | 6.48E-08 | 7.37E-05 |
| ENSG00000251504 | *LINC01099* | rs966109 | 4:178970124 | -0.62 | -5.63 | 6.52E-08 | 7.41E-05 |
| ENSG00000169071 | *ROR2* | rs7869182 | 9:94497042 | -0.52 | -5.63 | 6.53E-08 | 7.41E-05 |
| ENSG00000251504 | *LINC01099* | rs34775126 | 4:178969669 | -0.62 | -5.63 | 6.54E-08 | 7.42E-05 |
| ENSG00000251504 | *LINC01099* | rs6821159 | 4:178968052 | -0.62 | -5.63 | 6.60E-08 | 7.49E-05 |
| ENSG00000251504 | *LINC01099* | rs6820216 | 4:178967785 | -0.62 | -5.63 | 6.61E-08 | 7.50E-05 |
| ENSG00000251504 | *LINC01099* | rs62339524 | 4:178967369 | -0.62 | -5.63 | 6.63E-08 | 7.52E-05 |
| ENSG00000251504 | *LINC01099* | rs62339523 | 4:178967235 | -0.62 | -5.63 | 6.64E-08 | 7.53E-05 |
| ENSG00000251504 | *LINC01099* | rs41514145 | 4:178965658 | -0.62 | -5.63 | 6.71E-08 | 7.60E-05 |
| ENSG00000251504 | *LINC01099* | rs1676176 | 4:178964441 | -0.52 | -5.62 | 6.75E-08 | 7.65E-05 |
| ENSG00000251504 | *LINC01099* | rs1425001846 | 4:178961959 | -0.63 | -5.62 | 6.80E-08 | 7.69E-05 |
| ENSG00000251504 | *LINC01099* | rs56898809 | 4:178961738 | -0.61 | -5.62 | 6.85E-08 | 7.75E-05 |
| ENSG00000251504 | *LINC01099* | rs2045759 | 4:178962431 | -0.61 | -5.62 | 6.92E-08 | 7.82E-05 |
| ENSG00000251504 | *LINC01099* | rs2045757 | 4:178962336 | -0.61 | -5.62 | 6.92E-08 | 7.83E-05 |
| ENSG00000251504 | *LINC01099* | rs1023907 | 4:178961069 | -0.61 | -5.62 | 6.92E-08 | 7.83E-05 |
| ENSG00000251504 | *LINC01099* | rs2010754 | 4:178960519 | -0.61 | -5.62 | 6.92E-08 | 7.83E-05 |
| ENSG00000251504 | *LINC01099* | rs1037963 | 4:178960085 | -0.61 | -5.62 | 6.92E-08 | 7.83E-05 |
| ENSG00000251504 | *LINC01099* | rs1037964 | 4:178960206 | -0.61 | -5.62 | 6.92E-08 | 7.83E-05 |
| ENSG00000251504 | *LINC01099* | rs1037965 | 4:178960219 | -0.61 | -5.62 | 6.92E-08 | 7.83E-05 |
| ENSG00000251504 | *LINC01099* | rs55900761 | 4:178957400 | -0.61 | -5.62 | 6.93E-08 | 7.83E-05 |
| ENSG00000251504 | *LINC01099* | rs62339526 | 4:178970269 | -0.62 | -5.62 | 7.06E-08 | 7.96E-05 |
| ENSG00000151470 | *C4orf33* | rs6534707 | 4:129970673 | -0.70 | -5.61 | 7.14E-08 | 8.05E-05 |
| ENSG00000171522 | *PTGER4* | rs6885315 | 5:40429542 | 0.51 | 5.61 | 7.22E-08 | 8.13E-05 |
| ENSG00000251504 | *LINC01099* | rs1350946 | 4:178954527 | -0.61 | -5.61 | 7.25E-08 | 8.16E-05 |
| ENSG00000136108 | *CKAP2* | rs6561662 | 13:52909774 | 0.53 | 5.61 | 7.37E-08 | 8.29E-05 |
| ENSG00000251504 | *LINC01099* | rs1711369 | 4:178963944 | -0.51 | -5.60 | 7.68E-08 | 8.61E-05 |
| ENSG00000151470 | *C4orf33* | rs5861877 | 4:130034753 | -0.66 | -5.60 | 7.70E-08 | 8.63E-05 |
| ENSG00000171522 | *PTGER4* | rs6880934 | 5:40429192 | 0.51 | 5.60 | 7.71E-08 | 8.65E-05 |
| ENSG00000169071 | *ROR2* | rs10739925 | 9:94835310 | 0.54 | 5.59 | 7.82E-08 | 8.75E-05 |
| ENSG00000228716 | *DHFR* | rs1028780078 | 5:80106201 | 0.59 | 5.58 | 8.44E-08 | 9.40E-05 |
| ENSG00000228716 | *DHFR* | rs3073786 | 5:80105201 | 0.59 | 5.58 | 8.47E-08 | 9.43E-05 |
| ENSG00000151470 | *C4orf33* | rs280599 | 4:129945525 | -0.69 | -5.58 | 8.55E-08 | 9.52E-05 |
| ENSG00000149328 | *GLB1L2* | rs11608146 | 11:134242327 | 0.54 | 5.57 | 8.65E-08 | 9.62E-05 |
| ENSG00000228716 | *DHFR* | rs245364 | 5:80139382 | 0.59 | 5.57 | 8.79E-08 | 9.76E-05 |
| ENSG00000251504 | *LINC01099* | rs2045758 | 4:178962421 | -0.61 | -5.57 | 9.00E-08 | 9.98E-05 |
| ENSG00000251504 | *LINC01099* | rs2615591 | 4:178951563 | -0.51 | -5.56 | 9.08E-08 | 1.01E-04 |
| ENSG00000251504 | *LINC01099* | rs1676169 | 4:178951714 | -0.51 | -5.56 | 9.09E-08 | 1.01E-04 |
| ENSG00000251504 | *LINC01099* | rs1023909 | 4:178961304 | -0.51 | -5.56 | 9.10E-08 | 1.01E-04 |
| ENSG00000251504 | *LINC01099* | rs2054379 | 4:178943184 | -0.59 | -5.56 | 9.39E-08 | 1.04E-04 |
| ENSG00000251504 | *LINC01099* | rs4374579 | 4:178942172 | -0.59 | -5.55 | 9.58E-08 | 1.06E-04 |
| ENSG00000171522 | *PTGER4* | rs202019078 | 5:40401688 | 0.51 | 5.55 | 9.78E-08 | 1.08E-04 |
| ENSG00000149328 | *GLB1L2* | rs3133072 | 11:134196629 | -0.52 | -5.55 | 9.81E-08 | 1.08E-04 |
| ENSG00000251504 | *LINC01099* | rs143020738 | 4:178961198 | -0.61 | -5.55 | 9.86E-08 | 1.08E-04 |
| ENSG00000251504 | *LINC01099* | rs1192274693 | 4:178916404 | -0.61 | -5.54 | 1.02E-07 | 1.12E-04 |
| ENSG00000251504 | *LINC01099* | rs62341572 | 4:178937808 | -0.59 | -5.54 | 1.02E-07 | 1.12E-04 |
| ENSG00000151470 | *C4orf33* | rs1709422 | 4:130064878 | -0.56 | -5.54 | 1.03E-07 | 1.12E-04 |
| ENSG00000228716 | *DHFR* | rs6883090 | 5:79976388 | 0.54 | 5.54 | 1.03E-07 | 1.13E-04 |
| ENSG00000228716 | *DHFR* | rs32989 | 5:80116090 | 0.59 | 5.54 | 1.03E-07 | 1.13E-04 |
| ENSG00000228716 | *DHFR* | rs245357 | 5:80144041 | 0.59 | 5.54 | 1.03E-07 | 1.13E-04 |
| ENSG00000228716 | *DHFR* | rs245356 | 5:80145345 | 0.59 | 5.54 | 1.03E-07 | 1.13E-04 |
| ENSG00000228716 | *DHFR* | rs245355 | 5:80146387 | 0.59 | 5.54 | 1.03E-07 | 1.13E-04 |
| ENSG00000228716 | *DHFR* | rs26279 | 5:80168937 | 0.59 | 5.54 | 1.03E-07 | 1.13E-04 |
| ENSG00000228716 | *DHFR* | rs245360 | 5:80143343 | 0.59 | 5.54 | 1.03E-07 | 1.13E-04 |
| ENSG00000228716 | *DHFR* | rs32988 | 5:80116118 | 0.59 | 5.54 | 1.03E-07 | 1.13E-04 |
| ENSG00000228716 | *DHFR* | rs39626 | 5:80118794 | 0.59 | 5.54 | 1.03E-07 | 1.13E-04 |
| ENSG00000228716 | *DHFR* | rs40591 | 5:80113846 | 0.59 | 5.54 | 1.03E-07 | 1.13E-04 |
| ENSG00000228716 | *DHFR* | rs26278 | 5:80122122 | 0.59 | 5.54 | 1.03E-07 | 1.13E-04 |
| ENSG00000228716 | *DHFR* | rs27887 | 5:80169477 | 0.59 | 5.54 | 1.03E-07 | 1.13E-04 |
| ENSG00000228716 | *DHFR* | rs1679115 | 5:80122570 | 0.59 | 5.54 | 1.04E-07 | 1.13E-04 |
| ENSG00000228716 | *DHFR* | rs27494 | 5:80169543 | 0.59 | 5.54 | 1.04E-07 | 1.13E-04 |
| ENSG00000228716 | *DHFR* | rs245385 | 5:80128605 | 0.59 | 5.54 | 1.04E-07 | 1.14E-04 |
| ENSG00000228716 | *DHFR* | rs245407 | 5:80096734 | 0.60 | 5.54 | 1.04E-07 | 1.14E-04 |
| ENSG00000228716 | *DHFR* | rs245380 | 5:80130390 | 0.59 | 5.54 | 1.04E-07 | 1.14E-04 |
| ENSG00000228716 | *DHFR* | rs32963 | 5:80174728 | 0.59 | 5.54 | 1.04E-07 | 1.14E-04 |
| ENSG00000228716 | *DHFR* | rs32964 | 5:80175797 | 0.59 | 5.54 | 1.04E-07 | 1.14E-04 |
| ENSG00000228716 | *DHFR* | rs245374 | 5:80133109 | 0.59 | 5.54 | 1.04E-07 | 1.14E-04 |
| ENSG00000228716 | *DHFR* | rs245373 | 5:80133142 | 0.59 | 5.54 | 1.04E-07 | 1.14E-04 |
| ENSG00000228716 | *DHFR* | rs32967 | 5:80178256 | 0.59 | 5.54 | 1.05E-07 | 1.14E-04 |
| ENSG00000228716 | *DHFR* | rs27366 | 5:80147881 | 0.59 | 5.53 | 1.05E-07 | 1.15E-04 |
| ENSG00000228716 | *DHFR* | rs32968 | 5:80178884 | 0.59 | 5.53 | 1.06E-07 | 1.15E-04 |
| ENSG00000228716 | *DHFR* | rs1011290419 | 5:80122663 | 0.59 | 5.53 | 1.10E-07 | 1.20E-04 |
| ENSG00000251504 | *LINC01099* | rs1381695 | 4:178964396 | -0.51 | -5.52 | 1.12E-07 | 1.22E-04 |
| ENSG00000171522 | *PTGER4* | rs1445009 | 5:40401689 | 0.51 | 5.52 | 1.13E-07 | 1.23E-04 |
| ENSG00000228716 | *DHFR* | rs35868974 | 5:80179301 | 0.59 | 5.52 | 1.13E-07 | 1.23E-04 |
| ENSG00000228716 | *DHFR* | rs245348 | 5:80155595 | 0.59 | 5.51 | 1.16E-07 | 1.26E-04 |
| ENSG00000228716 | *DHFR* | rs245340 | 5:80161500 | 0.59 | 5.51 | 1.17E-07 | 1.27E-04 |
| ENSG00000228716 | *DHFR* | rs245341 | 5:80160393 | 0.58 | 5.51 | 1.19E-07 | 1.29E-04 |
| ENSG00000228716 | *DHFR* | rs168653 | 5:80154165 | 0.58 | 5.51 | 1.19E-07 | 1.29E-04 |
| ENSG00000251504 | *LINC01099* | rs28834138 | 4:178937795 | -0.58 | -5.51 | 1.21E-07 | 1.31E-04 |
| ENSG00000228716 | *DHFR* | rs2115014 | 5:79899922 | -0.53 | -5.50 | 1.22E-07 | 1.32E-04 |
| ENSG00000228716 | *DHFR* | rs1679120 | 5:80112001 | 0.57 | 5.49 | 1.28E-07 | 1.38E-04 |
| ENSG00000149328 | *GLB1L2* | rs481252 | 11:134188542 | -0.48 | -5.49 | 1.29E-07 | 1.39E-04 |
| ENSG00000251504 | *LINC01099* | rs10660369 | 4:178986473 | -0.51 | -5.48 | 1.40E-07 | 1.49E-04 |
| ENSG00000228716 | *DHFR* | rs245372 | 5:80164172 | 0.59 | 5.47 | 1.44E-07 | 1.53E-04 |
| ENSG00000251504 | *LINC01099* | rs1676178 | 4:178968674 | -0.50 | -5.47 | 1.44E-07 | 1.54E-04 |
| ENSG00000251504 | *LINC01099* | rs1599603 | 4:178971901 | -0.50 | -5.47 | 1.47E-07 | 1.56E-04 |
| ENSG00000171522 | *PTGER4* | rs6889125 | 5:40412590 | 0.52 | 5.46 | 1.52E-07 | 1.61E-04 |
| ENSG00000251504 | *LINC01099* | rs200517927 | 4:178981189 | -0.50 | -5.46 | 1.54E-07 | 1.63E-04 |
| ENSG00000169071 | *ROR2* | rs7047326 | 9:94707277 | 0.53 | 5.45 | 1.55E-07 | 1.64E-04 |
| ENSG00000136108 | *CKAP2* | rs72440971 | 13:53029770 | -0.55 | -5.45 | 1.56E-07 | 1.65E-04 |
| ENSG00000169071 | *ROR2* | rs149783420 | 9:94666332 | 0.57 | 5.45 | 1.57E-07 | 1.66E-04 |
| ENSG00000251504 | *LINC01099* | rs1381697 | 4:178970017 | -0.60 | -5.45 | 1.61E-07 | 1.70E-04 |
| ENSG00000228716 | *DHFR* | rs245389 | 5:80104368 | 0.58 | 5.45 | 1.62E-07 | 1.71E-04 |
| ENSG00000228716 | *DHFR* | rs36223115 | 5:80115189 | 0.56 | 5.44 | 1.66E-07 | 1.74E-04 |
| ENSG00000228716 | *DHFR* | rs32995 | 5:80113394 | 0.53 | 5.43 | 1.76E-07 | 1.84E-04 |
| ENSG00000251504 | *LINC01099* | rs2378810 | 4:178972443 | -0.50 | -5.43 | 1.77E-07 | 1.85E-04 |
| ENSG00000136108 | *CKAP2* | rs1287024424 | 13:53043732 | -0.55 | -5.43 | 1.77E-07 | 1.85E-04 |
| ENSG00000228716 | *DHFR* | rs32999 | 5:80106765 | 0.58 | 5.43 | 1.78E-07 | 1.86E-04 |
| ENSG00000169071 | *ROR2* | rs11789730 | 9:94631730 | 0.53 | 5.43 | 1.79E-07 | 1.86E-04 |
| ENSG00000251504 | *LINC01099* | rs1711366 | 4:178967267 | -0.50 | -5.42 | 1.87E-07 | 1.94E-04 |
| ENSG00000228716 | *DHFR* | rs245338 | 5:80162193 | 0.52 | 5.41 | 1.88E-07 | 1.95E-04 |
| ENSG00000251504 | *LINC01099* | rs1614976 | 4:178972564 | -0.50 | -5.41 | 1.88E-07 | 1.96E-04 |
| ENSG00000251504 | *LINC01099* | rs1186460439 | 4:178916395 | -0.61 | -5.41 | 1.89E-07 | 1.96E-04 |
| ENSG00000169071 | *ROR2* | rs34777436 | 9:94680533 | 0.56 | 5.41 | 1.89E-07 | 1.96E-04 |
| ENSG00000149328 | *GLB1L2* | rs1146189 | 11:134196442 | -0.53 | -5.41 | 1.91E-07 | 1.98E-04 |
| ENSG00000251504 | *LINC01099* | rs1711365 | 4:178968302 | -0.50 | -5.41 | 1.92E-07 | 1.99E-04 |
| ENSG00000228716 | *DHFR* | rs1223933898 | 5:80139376 | 0.57 | 5.41 | 1.92E-07 | 1.99E-04 |
| ENSG00000169071 | *ROR2* | rs10116249 | 9:94704033 | 0.53 | 5.40 | 2.01E-07 | 2.07E-04 |
| ENSG00000171522 | *PTGER4* | rs4526151 | 5:40435643 | 0.51 | 5.40 | 2.03E-07 | 2.09E-04 |
| ENSG00000171522 | *PTGER4* | rs6880809 | 5:40429250 | 0.51 | 5.40 | 2.05E-07 | 2.11E-04 |
| ENSG00000251504 | *LINC01099* | rs11729555 | 4:178975584 | -0.60 | -5.38 | 2.21E-07 | 2.25E-04 |
| ENSG00000251504 | *LINC01099* | rs1676175 | 4:178961278 | -0.49 | -5.38 | 2.22E-07 | 2.26E-04 |
| ENSG00000251504 | *LINC01099* | rs7688188 | 4:178976958 | -0.60 | -5.38 | 2.24E-07 | 2.29E-04 |
| ENSG00000228716 | *DHFR* | rs6151617 | 5:79961643 | 0.52 | 5.38 | 2.25E-07 | 2.30E-04 |
| ENSG00000228716 | *DHFR* | rs2081853 | 5:79964349 | 0.52 | 5.38 | 2.25E-07 | 2.30E-04 |
| ENSG00000171522 | *PTGER4* | rs4632848 | 5:40435886 | 0.51 | 5.38 | 2.27E-07 | 2.31E-04 |
| ENSG00000251504 | *LINC01099* | rs35684555 | 4:178977698 | -0.60 | -5.38 | 2.27E-07 | 2.31E-04 |
| ENSG00000251504 | *LINC01099* | rs1676182 | 4:178973899 | -0.49 | -5.38 | 2.27E-07 | 2.32E-04 |
| ENSG00000151470 | *C4orf33* | rs504968 | 4:130040437 | -0.63 | -5.37 | 2.28E-07 | 2.32E-04 |
| ENSG00000228716 | *DHFR* | rs443611 | 5:79919170 | -0.51 | -5.37 | 2.31E-07 | 2.35E-04 |
| ENSG00000151470 | *C4orf33* | rs1596963 | 4:129912966 | -0.66 | -5.37 | 2.31E-07 | 2.35E-04 |
| ENSG00000169071 | *ROR2* | rs10683668 | 9:94709046 | 0.52 | 5.37 | 2.32E-07 | 2.35E-04 |
| ENSG00000171522 | *PTGER4* | rs6880419 | 5:40428913 | 0.51 | 5.37 | 2.33E-07 | 2.37E-04 |
| ENSG00000228716 | *DHFR* | rs3958547 | 5:79995570 | 0.52 | 5.37 | 2.34E-07 | 2.38E-04 |
| ENSG00000251504 | *LINC01099* | rs1015093 | 4:178981044 | -0.50 | -5.37 | 2.35E-07 | 2.39E-04 |
| ENSG00000251504 | *LINC01099* | rs4690596 | 4:178979442 | -0.60 | -5.37 | 2.37E-07 | 2.40E-04 |
| ENSG00000228716 | *DHFR* | rs6151603 | 5:79960128 | 0.52 | 5.37 | 2.37E-07 | 2.40E-04 |
| ENSG00000149328 | *GLB1L2* | rs4937878 | 11:134235710 | 0.52 | 5.37 | 2.37E-07 | 2.41E-04 |
| ENSG00000169071 | *ROR2* | rs11790144 | 9:94631985 | 0.53 | 5.36 | 2.40E-07 | 2.43E-04 |
| ENSG00000228716 | *DHFR* | rs35100179 | 5:80121401 | 0.58 | 5.36 | 2.45E-07 | 2.48E-04 |
| ENSG00000136108 | *CKAP2* | rs200296410 | 13:52909772 | 0.55 | 5.36 | 2.46E-07 | 2.48E-04 |
| ENSG00000171522 | *PTGER4* | rs10042027 | 5:40428811 | 0.51 | 5.36 | 2.46E-07 | 2.49E-04 |
| ENSG00000251504 | *LINC01099* | rs35489440 | 4:178974212 | -0.60 | -5.35 | 2.50E-07 | 2.53E-04 |
| ENSG00000251504 | *LINC01099* | rs981454 | 4:178974641 | -0.60 | -5.35 | 2.51E-07 | 2.53E-04 |
| ENSG00000251504 | *LINC01099* | rs5864487 | 4:178981242 | -0.60 | -5.35 | 2.51E-07 | 2.53E-04 |
| ENSG00000251504 | *LINC01099* | rs11728674 | 4:178975311 | -0.60 | -5.35 | 2.52E-07 | 2.54E-04 |
| ENSG00000228716 | *DHFR* | rs9293824 | 5:80181748 | -0.58 | -5.35 | 2.52E-07 | 2.54E-04 |
| ENSG00000251504 | *LINC01099* | rs7687405 | 4:178976553 | -0.60 | -5.35 | 2.55E-07 | 2.57E-04 |
| ENSG00000171522 | *PTGER4* | rs6874500 | 5:40427883 | 0.51 | 5.35 | 2.55E-07 | 2.57E-04 |
| ENSG00000251504 | *LINC01099* | rs7666348 | 4:178976944 | -0.60 | -5.35 | 2.56E-07 | 2.58E-04 |
| ENSG00000171522 | *PTGER4* | rs10473183 | 5:40370251 | 0.50 | 5.35 | 2.57E-07 | 2.58E-04 |
| ENSG00000171522 | *PTGER4* | rs10472332 | 5:40368373 | 0.50 | 5.35 | 2.57E-07 | 2.58E-04 |
| ENSG00000171522 | *PTGER4* | rs2120855 | 5:40383620 | 0.50 | 5.35 | 2.57E-07 | 2.59E-04 |
| ENSG00000171522 | *PTGER4* | rs7445978 | 5:40381056 | 0.50 | 5.35 | 2.59E-07 | 2.60E-04 |
| ENSG00000169071 | *ROR2* | rs1212002858 | 9:94665435 | 0.97 | 5.35 | 2.59E-07 | 2.60E-04 |
| ENSG00000171522 | *PTGER4* | rs34300575 | 5:40371286 | 0.50 | 5.35 | 2.61E-07 | 2.62E-04 |
| ENSG00000171522 | *PTGER4* | rs1899982 | 5:40425045 | 0.51 | 5.34 | 2.65E-07 | 2.66E-04 |
| ENSG00000171522 | *PTGER4* | rs7730306 | 5:40423257 | 0.51 | 5.34 | 2.66E-07 | 2.67E-04 |
| ENSG00000171522 | *PTGER4* | rs12656644 | 5:40387432 | 0.50 | 5.34 | 2.66E-07 | 2.67E-04 |
| ENSG00000171522 | *PTGER4* | rs7705708 | 5:40420658 | 0.51 | 5.34 | 2.66E-07 | 2.67E-04 |
| ENSG00000171522 | *PTGER4* | rs12655827 | 5:40412335 | 0.50 | 5.34 | 2.66E-07 | 2.67E-04 |
| ENSG00000171522 | *PTGER4* | rs6891041 | 5:40406238 | 0.50 | 5.34 | 2.67E-07 | 2.67E-04 |
| ENSG00000171522 | *PTGER4* | rs6883302 | 5:40411414 | 0.50 | 5.34 | 2.67E-07 | 2.67E-04 |
| ENSG00000171522 | *PTGER4* | rs10473189 | 5:40402692 | 0.50 | 5.34 | 2.67E-07 | 2.68E-04 |
| ENSG00000171522 | *PTGER4* | rs10941505 | 5:40399246 | 0.50 | 5.34 | 2.67E-07 | 2.68E-04 |
| ENSG00000171522 | *PTGER4* | rs6891952 | 5:40400885 | 0.50 | 5.34 | 2.67E-07 | 2.68E-04 |
| ENSG00000169071 | *ROR2* | rs12376130 | 9:94620277 | 0.53 | 5.34 | 2.68E-07 | 2.69E-04 |
| ENSG00000228716 | *DHFR* | rs245386 | 5:80127665 | 0.57 | 5.34 | 2.69E-07 | 2.69E-04 |
| ENSG00000171522 | *PTGER4* | rs7730693 | 5:40373105 | 0.50 | 5.34 | 2.69E-07 | 2.70E-04 |
| ENSG00000251504 | *LINC01099* | rs1042863017 | 4:178982502 | -0.60 | -5.34 | 2.70E-07 | 2.70E-04 |
| ENSG00000136108 | *CKAP2* | rs9536069 | 13:52994756 | -0.53 | -5.34 | 2.70E-07 | 2.71E-04 |
| ENSG00000171522 | *PTGER4* | rs11291962 | 5:40396940 | 0.50 | 5.34 | 2.71E-07 | 2.71E-04 |
| ENSG00000171522 | *PTGER4* | rs10072596 | 5:40407033 | 0.50 | 5.34 | 2.75E-07 | 2.75E-04 |
| ENSG00000251504 | *LINC01099* | rs56352325 | 4:178980377 | -0.59 | -5.33 | 2.77E-07 | 2.76E-04 |
| ENSG00000251504 | *LINC01099* | rs1381705 | 4:178987226 | -0.49 | -5.33 | 2.77E-07 | 2.76E-04 |
| ENSG00000169071 | *ROR2* | rs6479389 | 9:94716442 | 0.51 | 5.33 | 2.77E-07 | 2.77E-04 |
| ENSG00000169071 | *ROR2* | rs7048699 | 9:94708403 | 0.51 | 5.33 | 2.77E-07 | 2.77E-04 |
| ENSG00000169071 | *ROR2* | rs7863167 | 9:94705498 | 0.51 | 5.33 | 2.78E-07 | 2.77E-04 |
| ENSG00000228716 | *DHFR* | rs201457721 | 5:79912891 | -0.56 | -5.33 | 2.81E-07 | 2.80E-04 |
| ENSG00000228716 | *DHFR* | rs2035256 | 5:79996632 | 0.51 | 5.33 | 2.84E-07 | 2.83E-04 |
| ENSG00000171522 | *PTGER4* | rs9292776 | 5:40437845 | 0.50 | 5.33 | 2.87E-07 | 2.86E-04 |
| ENSG00000251504 | *LINC01099* | rs6821187 | 4:178981923 | -0.59 | -5.33 | 2.87E-07 | 2.86E-04 |
| ENSG00000171522 | *PTGER4* | rs7718352 | 5:40441019 | 0.50 | 5.33 | 2.87E-07 | 2.86E-04 |
| ENSG00000151470 | *C4orf33* | rs337266 | 4:130044425 | -0.65 | -5.32 | 2.90E-07 | 2.88E-04 |
| ENSG00000171522 | *PTGER4* | rs12658567 | 5:40391932 | 0.50 | 5.32 | 2.92E-07 | 2.90E-04 |
| ENSG00000228716 | *DHFR* | rs67620227 | 5:80106202 | 0.57 | 5.32 | 2.92E-07 | 2.91E-04 |
| ENSG00000127325 | *BEST3* | rs59663283 | 12:70068823 | 0.53 | 5.31 | 3.07E-07 | 3.04E-04 |
| ENSG00000169071 | *ROR2* | rs7046430 | 9:94860578 | 0.51 | 5.31 | 3.08E-07 | 3.05E-04 |
| ENSG00000169071 | *ROR2* | rs6479388 | 9:94711672 | 0.51 | 5.31 | 3.09E-07 | 3.05E-04 |
| ENSG00000169071 | *ROR2* | rs7038017 | 9:94696852 | 0.51 | 5.30 | 3.27E-07 | 3.22E-04 |
| ENSG00000169071 | *ROR2* | rs7037255 | 9:94696954 | 0.51 | 5.30 | 3.27E-07 | 3.22E-04 |
| ENSG00000228716 | *DHFR* | rs40139 | 5:80033835 | 0.51 | 5.29 | 3.40E-07 | 3.34E-04 |
| ENSG00000149328 | *GLB1L2* | rs4937879 | 11:134235924 | 0.51 | 5.29 | 3.48E-07 | 3.40E-04 |
| ENSG00000251504 | *LINC01099* | rs62340561 | 4:178984340 | -0.59 | -5.28 | 3.50E-07 | 3.42E-04 |
| ENSG00000149328 | *GLB1L2* | rs2119297 | 11:134237620 | 0.51 | 5.28 | 3.52E-07 | 3.44E-04 |
| ENSG00000169071 | *ROR2* | rs368446555 | 9:94702986 | 0.52 | 5.28 | 3.54E-07 | 3.45E-04 |
| ENSG00000169071 | *ROR2* | rs11792319 | 9:94662657 | 0.53 | 5.28 | 3.55E-07 | 3.47E-04 |
| ENSG00000251504 | *LINC01099* | rs62340562 | 4:178984507 | -0.59 | -5.28 | 3.55E-07 | 3.47E-04 |
| ENSG00000169071 | *ROR2* | rs10992075 | 9:94501891 | 0.55 | 5.27 | 3.70E-07 | 3.59E-04 |
| ENSG00000136108 | *CKAP2* | rs7336679 | 13:53043333 | -0.52 | -5.27 | 3.70E-07 | 3.60E-04 |
| ENSG00000228716 | *DHFR* | rs140570977 | 5:80140546 | 0.53 | 5.27 | 3.71E-07 | 3.61E-04 |
| ENSG00000171522 | *PTGER4* | rs13189778 | 5:40421243 | 0.50 | 5.27 | 3.82E-07 | 3.70E-04 |
| ENSG00000228716 | *DHFR* | rs6151622 | 5:79961856 | -0.60 | -5.26 | 3.89E-07 | 3.77E-04 |
| ENSG00000169071 | *ROR2* | rs10820931 | 9:94754056 | 0.51 | 5.26 | 3.90E-07 | 3.78E-04 |
| ENSG00000149328 | *GLB1L2* | rs9783402 | 11:134245284 | 0.50 | 5.26 | 3.94E-07 | 3.81E-04 |
| ENSG00000251504 | *LINC01099* | rs1711386 | 4:178988148 | -0.48 | -5.26 | 3.98E-07 | 3.84E-04 |
| ENSG00000228716 | *DHFR* | rs32950 | 5:80018244 | 0.51 | 5.26 | 3.99E-07 | 3.85E-04 |
| ENSG00000136108 | *CKAP2* | rs7333451 | 13:53047709 | -0.52 | -5.25 | 4.09E-07 | 3.94E-04 |
| ENSG00000169071 | *ROR2* | rs7850702 | 9:94758904 | 0.51 | 5.25 | 4.21E-07 | 4.04E-04 |
| ENSG00000169071 | *ROR2* | rs1354561306 | 9:94516335 | -0.52 | -5.24 | 4.25E-07 | 4.07E-04 |
| ENSG00000169071 | *ROR2* | rs10739923 | 9:94746291 | 0.51 | 5.24 | 4.35E-07 | 4.16E-04 |
| ENSG00000169071 | *ROR2* | rs12340044 | 9:94770521 | 0.51 | 5.24 | 4.37E-07 | 4.18E-04 |
| ENSG00000169071 | *ROR2* | rs10733743 | 9:94768542 | 0.50 | 5.24 | 4.38E-07 | 4.19E-04 |
| ENSG00000169071 | *ROR2* | rs7034357 | 9:94740446 | 0.51 | 5.24 | 4.42E-07 | 4.23E-04 |
| ENSG00000136108 | *CKAP2* | rs4884354 | 13:53017474 | -0.52 | -5.23 | 4.45E-07 | 4.25E-04 |
| ENSG00000136108 | *CKAP2* | rs9379 | 13:53050479 | -0.52 | -5.23 | 4.45E-07 | 4.25E-04 |
| ENSG00000169071 | *ROR2* | rs9409654 | 9:94506614 | -0.53 | -5.23 | 4.48E-07 | 4.27E-04 |
| ENSG00000169071 | *ROR2* | rs4401931 | 9:94507454 | -0.53 | -5.23 | 4.50E-07 | 4.30E-04 |
| ENSG00000169071 | *ROR2* | rs7867072 | 9:94729227 | 0.51 | 5.23 | 4.53E-07 | 4.32E-04 |
| ENSG00000169071 | *ROR2* | rs10761138 | 9:94730341 | 0.51 | 5.23 | 4.53E-07 | 4.32E-04 |
| ENSG00000151470 | *C4orf33* | rs397715958 | 4:130025913 | -0.62 | -5.23 | 4.53E-07 | 4.32E-04 |
| ENSG00000169071 | *ROR2* | rs7852032 | 9:94720466 | 0.50 | 5.23 | 4.54E-07 | 4.33E-04 |
| ENSG00000169071 | *ROR2* | rs1316268 | 9:94734562 | 0.51 | 5.23 | 4.58E-07 | 4.37E-04 |
| ENSG00000228716 | *DHFR* | rs6864512 | 5:79985947 | 0.51 | 5.23 | 4.59E-07 | 4.37E-04 |
| ENSG00000228716 | *DHFR* | rs6868774 | 5:79985963 | 0.51 | 5.23 | 4.59E-07 | 4.37E-04 |
| ENSG00000136108 | *CKAP2* | rs875673 | 13:53323950 | -0.49 | -5.22 | 4.68E-07 | 4.45E-04 |
| ENSG00000171522 | *PTGER4* | rs7722414 | 5:40441343 | 0.50 | 5.22 | 4.72E-07 | 4.48E-04 |
| ENSG00000136108 | *CKAP2* | rs4886077 | 13:52998697 | -0.52 | -5.22 | 4.74E-07 | 4.49E-04 |
| ENSG00000136108 | *CKAP2* | rs7323666 | 13:53006058 | -0.52 | -5.22 | 4.74E-07 | 4.49E-04 |
| ENSG00000136108 | *CKAP2* | rs8001624 | 13:53002995 | -0.52 | -5.22 | 4.74E-07 | 4.49E-04 |
| ENSG00000136108 | *CKAP2* | rs1056335 | 13:52989863 | -0.52 | -5.22 | 4.75E-07 | 4.51E-04 |
| ENSG00000169071 | *ROR2* | rs2895201 | 9:94856495 | 0.51 | 5.22 | 4.78E-07 | 4.53E-04 |
| ENSG00000136108 | *CKAP2* | rs138123023 | 13:52835439 | -0.52 | -5.22 | 4.80E-07 | 4.55E-04 |
| ENSG00000169071 | *ROR2* | rs4372069 | 9:94515416 | -0.53 | -5.21 | 5.01E-07 | 4.73E-04 |
| ENSG00000169071 | *ROR2* | rs4585802 | 9:94515565 | -0.53 | -5.21 | 5.01E-07 | 4.73E-04 |
| ENSG00000228716 | *DHFR* | rs33002 | 5:80165378 | 0.50 | 5.21 | 5.09E-07 | 4.80E-04 |
| ENSG00000169071 | *ROR2* | rs7848874 | 9:94871720 | 0.51 | 5.20 | 5.12E-07 | 4.82E-04 |
| ENSG00000169071 | *ROR2* | rs10820909 | 9:94641723 | 0.55 | 5.20 | 5.19E-07 | 4.88E-04 |
| ENSG00000228716 | *DHFR* | rs1056460783 | 5:80012251 | -0.60 | -5.20 | 5.19E-07 | 4.88E-04 |
| ENSG00000169071 | *ROR2* | rs9409650 | 9:94490838 | -0.50 | -5.20 | 5.24E-07 | 4.92E-04 |
| ENSG00000228716 | *DHFR* | rs201279646 | 5:79896559 | -0.53 | -5.20 | 5.29E-07 | 4.97E-04 |
| ENSG00000228716 | *DHFR* | rs32969 | 5:80179156 | 0.55 | 5.19 | 5.37E-07 | 5.03E-04 |
| ENSG00000171522 | *PTGER4* | rs12697405 | 5:40323517 | 0.51 | 5.19 | 5.60E-07 | 5.23E-04 |
| ENSG00000169071 | *ROR2* | rs4467996 | 9:94517715 | -0.49 | -5.18 | 5.62E-07 | 5.24E-04 |
| ENSG00000149328 | *GLB1L2* | rs1144213 | 11:134188408 | -0.50 | -5.17 | 5.95E-07 | 5.52E-04 |
| ENSG00000251504 | *LINC01099* | rs143760895 | 4:178941639 | -0.60 | -5.17 | 5.96E-07 | 5.52E-04 |
| ENSG00000169071 | *ROR2* | rs62565678 | 9:94661796 | 0.53 | 5.17 | 6.02E-07 | 5.57E-04 |
| ENSG00000169071 | *ROR2* | rs7035867 | 9:94849855 | 0.53 | 5.16 | 6.19E-07 | 5.72E-04 |
| ENSG00000171522 | *PTGER4* | rs7712308 | 5:40351716 | 0.49 | 5.16 | 6.21E-07 | 5.73E-04 |
| ENSG00000169071 | *ROR2* | rs10820914 | 9:94655862 | 0.50 | 5.16 | 6.33E-07 | 5.83E-04 |
| ENSG00000169071 | *ROR2* | rs4500142 | 9:94503309 | -0.52 | -5.16 | 6.33E-07 | 5.84E-04 |
| ENSG00000169071 | *ROR2* | rs4517189 | 9:94657130 | 0.50 | 5.16 | 6.35E-07 | 5.85E-04 |
| ENSG00000169071 | *ROR2* | rs7871444 | 9:94855553 | 0.50 | 5.16 | 6.35E-07 | 5.85E-04 |
| ENSG00000169071 | *ROR2* | rs3935053 | 9:94508114 | -0.52 | -5.16 | 6.37E-07 | 5.86E-04 |
| ENSG00000169071 | *ROR2* | rs3935846 | 9:94506959 | -0.52 | -5.16 | 6.37E-07 | 5.86E-04 |
| ENSG00000169071 | *ROR2* | rs58431919 | 9:94654782 | 0.50 | 5.16 | 6.42E-07 | 5.91E-04 |
| ENSG00000169071 | *ROR2* | rs59723153 | 9:94654689 | 0.50 | 5.16 | 6.43E-07 | 5.92E-04 |
| ENSG00000169071 | *ROR2* | rs9409457 | 9:94495286 | -0.49 | -5.15 | 6.49E-07 | 5.96E-04 |
| ENSG00000169071 | *ROR2* | rs9409655 | 9:94510635 | -0.52 | -5.15 | 6.53E-07 | 6.00E-04 |
| ENSG00000169071 | *ROR2* | rs9409656 | 9:94510673 | -0.52 | -5.15 | 6.53E-07 | 6.00E-04 |
| ENSG00000169071 | *ROR2* | rs9409651 | 9:94498550 | -0.49 | -5.15 | 6.61E-07 | 6.06E-04 |
| ENSG00000169071 | *ROR2* | rs9409458 | 9:94498370 | -0.49 | -5.15 | 6.61E-07 | 6.06E-04 |
| ENSG00000169071 | *ROR2* | rs10992151 | 9:94652613 | 0.50 | 5.15 | 6.61E-07 | 6.06E-04 |
| ENSG00000169071 | *ROR2* | rs12683797 | 9:94649104 | 0.50 | 5.15 | 6.61E-07 | 6.07E-04 |
| ENSG00000169071 | *ROR2* | rs10992149 | 9:94648679 | 0.50 | 5.15 | 6.63E-07 | 6.08E-04 |
| ENSG00000169071 | *ROR2* | rs11789973 | 9:94651984 | 0.50 | 5.15 | 6.67E-07 | 6.11E-04 |
| ENSG00000169071 | *ROR2* | rs7034363 | 9:94740471 | 0.51 | 5.15 | 6.68E-07 | 6.12E-04 |
| ENSG00000171522 | *PTGER4* | rs12697408 | 5:40348573 | 0.49 | 5.14 | 6.77E-07 | 6.20E-04 |
| ENSG00000228716 | *DHFR* | rs34486413 | 5:80160596 | 0.49 | 5.14 | 6.77E-07 | 6.20E-04 |
| ENSG00000169071 | *ROR2* | rs10739918 | 9:94514292 | -0.52 | -5.14 | 6.85E-07 | 6.26E-04 |
| ENSG00000169071 | *ROR2* | rs9409653 | 9:94506153 | -0.52 | -5.14 | 6.86E-07 | 6.26E-04 |
| ENSG00000169071 | *ROR2* | rs10739919 | 9:94514322 | -0.52 | -5.14 | 6.86E-07 | 6.26E-04 |
| ENSG00000169071 | *ROR2* | rs7859689 | 9:94839649 | 0.50 | 5.14 | 6.92E-07 | 6.32E-04 |
| ENSG00000169071 | *ROR2* | rs7855522 | 9:94499618 | -0.49 | -5.14 | 6.94E-07 | 6.33E-04 |
| ENSG00000169071 | *ROR2* | rs3935602 | 9:94508663 | -0.52 | -5.14 | 7.00E-07 | 6.38E-04 |
| ENSG00000228716 | *DHFR* | rs1216198009 | 5:79934285 | -0.59 | -5.14 | 7.01E-07 | 6.38E-04 |
| ENSG00000136108 | *CKAP2* | rs7985262 | 13:52997161 | -0.51 | -5.14 | 7.01E-07 | 6.39E-04 |
| ENSG00000149328 | *GLB1L2* | rs1144219 | 11:134172031 | -0.51 | -5.14 | 7.01E-07 | 6.39E-04 |
| ENSG00000228716 | *DHFR* | rs408626 | 5:79951133 | 0.49 | 5.13 | 7.10E-07 | 6.46E-04 |
| ENSG00000151470 | *C4orf33* | rs337267 | 4:130043436 | -0.63 | -5.13 | 7.12E-07 | 6.48E-04 |
| ENSG00000169071 | *ROR2* | rs4744113 | 9:94648931 | 0.50 | 5.13 | 7.14E-07 | 6.49E-04 |
| ENSG00000169071 | *ROR2* | rs10118072 | 9:94840979 | 0.50 | 5.13 | 7.15E-07 | 6.50E-04 |
| ENSG00000136108 | *CKAP2* | rs13431 | 13:52987477 | -0.51 | -5.13 | 7.28E-07 | 6.61E-04 |
| ENSG00000171522 | *PTGER4* | rs10045016 | 5:40328325 | 0.49 | 5.13 | 7.30E-07 | 6.62E-04 |
| ENSG00000169071 | *ROR2* | rs2297568 | 9:94874651 | 0.50 | 5.13 | 7.39E-07 | 6.69E-04 |
| ENSG00000169071 | *ROR2* | rs9409456 | 9:94494561 | -0.49 | -5.12 | 7.43E-07 | 6.73E-04 |
| ENSG00000169071 | *ROR2* | rs2030992 | 9:94669239 | 0.53 | 5.12 | 7.48E-07 | 6.77E-04 |
| ENSG00000251504 | *LINC01099* | rs28771164 | 4:178939844 | -0.50 | -5.12 | 7.62E-07 | 6.88E-04 |
| ENSG00000171522 | *PTGER4* | rs6879428 | 5:40404227 | 0.50 | 5.12 | 7.62E-07 | 6.88E-04 |
| ENSG00000169071 | *ROR2* | rs4347043 | 9:94662787 | 0.53 | 5.12 | 7.67E-07 | 6.92E-04 |
| ENSG00000169071 | *ROR2* | rs4347044 | 9:94662817 | 0.53 | 5.12 | 7.67E-07 | 6.92E-04 |
| ENSG00000169071 | *ROR2* | rs138871026 | 9:94677249 | 0.53 | 5.12 | 7.68E-07 | 6.92E-04 |
| ENSG00000169071 | *ROR2* | rs10992158 | 9:94674927 | 0.53 | 5.12 | 7.72E-07 | 6.96E-04 |
| ENSG00000169071 | *ROR2* | rs3905385 | 9:94668733 | 0.53 | 5.12 | 7.74E-07 | 6.98E-04 |
| ENSG00000136108 | *CKAP2* | rs3803262 | 13:53036398 | -0.51 | -5.11 | 7.83E-07 | 7.04E-04 |
| ENSG00000169071 | *ROR2* | rs57688942 | 9:94664869 | 0.53 | 5.11 | 7.85E-07 | 7.06E-04 |
| ENSG00000170369 | *CST2* | rs201305936 | 20:23777058 | -0.52 | -5.11 | 7.90E-07 | 7.11E-04 |
| ENSG00000251504 | *LINC01099* | rs144875726 | 4:178939803 | -0.63 | -5.11 | 8.01E-07 | 7.19E-04 |
| ENSG00000169071 | *ROR2* | rs1244531635 | 9:94667336 | 0.53 | 5.11 | 8.05E-07 | 7.22E-04 |
| ENSG00000228716 | *DHFR* | rs10685456 | 5:79942318 | -0.51 | -5.11 | 8.06E-07 | 7.23E-04 |
| ENSG00000169071 | *ROR2* | rs6479400 | 9:94805306 | 0.49 | 5.10 | 8.25E-07 | 7.38E-04 |
| ENSG00000169071 | *ROR2* | rs10512220 | 9:94806331 | 0.49 | 5.10 | 8.25E-07 | 7.38E-04 |
| ENSG00000169071 | *ROR2* | rs10820915 | 9:94666958 | 0.53 | 5.10 | 8.37E-07 | 7.48E-04 |
| ENSG00000169071 | *ROR2* | rs1881384 | 9:94666993 | 0.53 | 5.10 | 8.37E-07 | 7.48E-04 |
| ENSG00000136108 | *CKAP2* | rs1836931 | 13:53185646 | -0.53 | -5.10 | 8.48E-07 | 7.57E-04 |
| ENSG00000136108 | *CKAP2* | rs1836932 | 13:53185648 | -0.53 | -5.10 | 8.48E-07 | 7.57E-04 |
| ENSG00000169071 | *ROR2* | rs2030991 | 9:94645339 | 0.51 | 5.09 | 8.65E-07 | 7.70E-04 |
| ENSG00000169071 | *ROR2* | rs1418185522 | 9:94667447 | 0.53 | 5.09 | 8.66E-07 | 7.71E-04 |
| ENSG00000169071 | *ROR2* | rs12555334 | 9:94644979 | 0.51 | 5.09 | 8.68E-07 | 7.72E-04 |
| ENSG00000169071 | *ROR2* | rs7041193 | 9:94820893 | 0.49 | 5.09 | 8.69E-07 | 7.73E-04 |
| ENSG00000169071 | *ROR2* | rs1892268 | 9:94820446 | 0.49 | 5.09 | 8.69E-07 | 7.73E-04 |
| ENSG00000169071 | *ROR2* | rs58202197 | 9:94643546 | 0.51 | 5.09 | 8.74E-07 | 7.77E-04 |
| ENSG00000169071 | *ROR2* | rs7853136 | 9:94685210 | 0.51 | 5.09 | 8.81E-07 | 7.83E-04 |
| ENSG00000169071 | *ROR2* | rs10820918 | 9:94686453 | 0.51 | 5.09 | 8.82E-07 | 7.83E-04 |
| ENSG00000169071 | *ROR2* | rs7020136 | 9:94831511 | 0.49 | 5.09 | 8.91E-07 | 7.90E-04 |
| ENSG00000169071 | *ROR2* | rs6479402 | 9:94829027 | 0.49 | 5.09 | 8.91E-07 | 7.90E-04 |
| ENSG00000169071 | *ROR2* | rs62565747 | 9:94678097 | 0.53 | 5.08 | 9.03E-07 | 8.00E-04 |
| ENSG00000127325 | *BEST3* | rs3741755 | 12:70072479 | 0.50 | 5.08 | 9.14E-07 | 8.08E-04 |
| ENSG00000169071 | *ROR2* | rs7033819 | 9:94638008 | 0.52 | 5.08 | 9.15E-07 | 8.08E-04 |
| ENSG00000170369 | *CST2* | rs6132663 | 20:23770738 | -0.52 | -5.08 | 9.16E-07 | 8.10E-04 |
| ENSG00000169071 | *ROR2* | rs10118816 | 9:94691478 | 0.50 | 5.08 | 9.23E-07 | 8.14E-04 |
| ENSG00000169071 | *ROR2* | rs10118792 | 9:94691380 | 0.50 | 5.08 | 9.23E-07 | 8.15E-04 |
| ENSG00000228716 | *DHFR* | rs380691 | 5:79952034 | 0.50 | 5.08 | 9.25E-07 | 8.17E-04 |
| ENSG00000228716 | *DHFR* | rs442767 | 5:79951496 | 0.50 | 5.08 | 9.28E-07 | 8.19E-04 |
| ENSG00000228716 | *DHFR* | rs1650713 | 5:79934417 | -0.59 | -5.08 | 9.31E-07 | 8.21E-04 |
| ENSG00000169071 | *ROR2* | rs62565674 | 9:94637165 | 0.52 | 5.07 | 9.35E-07 | 8.24E-04 |
| ENSG00000149328 | *GLB1L2* | rs1258842 | 11:134167225 | -0.51 | -5.07 | 9.37E-07 | 8.25E-04 |
| ENSG00000228716 | *DHFR* | rs1677702 | 5:79931247 | -0.59 | -5.07 | 9.45E-07 | 8.32E-04 |
| ENSG00000169071 | *ROR2* | rs10992136 | 9:94617709 | 0.52 | 5.07 | 9.52E-07 | 8.37E-04 |
| ENSG00000169071 | *ROR2* | rs7848987 | 9:94795992 | 0.49 | 5.07 | 9.62E-07 | 8.45E-04 |
| ENSG00000127325 | *BEST3* | rs73135873 | 12:70068747 | 0.53 | 5.07 | 9.68E-07 | 8.50E-04 |
| ENSG00000169071 | *ROR2* | rs62564599 | 9:94614206 | 0.53 | 5.07 | 9.69E-07 | 8.50E-04 |
| ENSG00000228716 | *DHFR* | rs142352906 | 5:79947863 | -0.62 | -5.07 | 9.71E-07 | 8.52E-04 |
| ENSG00000169071 | *ROR2* | rs1534530 | 9:94606212 | 0.51 | 5.07 | 9.76E-07 | 8.56E-04 |
| ENSG00000169071 | *ROR2* | rs7042102 | 9:94763790 | 0.49 | 5.06 | 9.88E-07 | 8.65E-04 |
| ENSG00000228716 | *DHFR* | rs13185915 | 5:79905411 | -0.60 | -5.06 | 9.91E-07 | 8.68E-04 |
| ENSG00000170369 | *CST2* | rs4319988 | 20:23775453 | -0.50 | -5.06 | 9.94E-07 | 8.70E-04 |
| ENSG00000170369 | *CST2* | rs4549173 | 20:23775488 | -0.50 | -5.06 | 9.94E-07 | 8.70E-04 |
| ENSG00000170369 | *CST2* | rs4583530 | 20:23775772 | -0.50 | -5.06 | 9.95E-07 | 8.71E-04 |
| ENSG00000171522 | *PTGER4* | rs13186168 | 5:40376047 | 0.48 | 5.06 | 9.96E-07 | 8.71E-04 |
| ENSG00000171522 | *PTGER4* | rs11952844 | 5:40384295 | 0.48 | 5.06 | 9.98E-07 | 8.73E-04 |
| ENSG00000149328 | *GLB1L2* | rs35022697 | 11:134151812 | -0.49 | -5.06 | 1.00E-06 | 8.78E-04 |
| ENSG00000169071 | *ROR2* | rs10992145 | 9:94636075 | 0.51 | 5.06 | 1.01E-06 | 8.81E-04 |
| ENSG00000228716 | *DHFR* | rs1611028 | 5:79953680 | 0.50 | 5.06 | 1.01E-06 | 8.84E-04 |
| ENSG00000136108 | *CKAP2* | rs6561682 | 13:53266297 | 0.54 | 5.06 | 1.01E-06 | 8.85E-04 |
| ENSG00000169071 | *ROR2* | rs7047937 | 9:94516768 | -0.49 | -5.05 | 1.03E-06 | 9.00E-04 |
| ENSG00000169071 | *ROR2* | rs67944481 | 9:94631759 | 0.52 | 5.05 | 1.04E-06 | 9.02E-04 |
| ENSG00000169071 | *ROR2* | rs56308784 | 9:94621508 | 0.52 | 5.05 | 1.04E-06 | 9.06E-04 |
| ENSG00000134278 | *SPIRE1* | rs72877242 | 18:12501428 | 0.87 | 5.05 | 1.04E-06 | 9.07E-04 |
| ENSG00000169071 | *ROR2* | rs34365495 | 9:94635690 | 0.51 | 5.05 | 1.04E-06 | 9.08E-04 |
| ENSG00000170369 | *CST2* | rs4438551 | 20:23774050 | -0.50 | -5.05 | 1.05E-06 | 9.11E-04 |
| ENSG00000169071 | *ROR2* | rs7031729 | 9:94506930 | -0.48 | -5.05 | 1.05E-06 | 9.14E-04 |
| ENSG00000169071 | *ROR2* | rs1881387 | 9:94633406 | 0.52 | 5.05 | 1.05E-06 | 9.15E-04 |
| ENSG00000169071 | *ROR2* | rs12552858 | 9:94629592 | 0.52 | 5.05 | 1.05E-06 | 9.15E-04 |
| ENSG00000169071 | *ROR2* | rs55956910 | 9:94619276 | 0.52 | 5.05 | 1.05E-06 | 9.15E-04 |
| ENSG00000149328 | *GLB1L2* | rs3132808 | 11:134201612 | -0.60 | -5.05 | 1.05E-06 | 9.17E-04 |
| ENSG00000169071 | *ROR2* | rs1013590765 | 9:94628401 | 0.52 | 5.05 | 1.06E-06 | 9.19E-04 |
| ENSG00000169071 | *ROR2* | rs3935601 | 9:94508700 | -0.48 | -5.05 | 1.06E-06 | 9.20E-04 |
| ENSG00000251504 | *LINC01099* | rs201911210 | 4:178951562 | -0.48 | -5.05 | 1.07E-06 | 9.28E-04 |
| ENSG00000228716 | *DHFR* | rs1650707 | 5:79939257 | -0.59 | -5.04 | 1.08E-06 | 9.32E-04 |
| ENSG00000228716 | *DHFR* | rs34268388 | 5:79941419 | -0.59 | -5.04 | 1.08E-06 | 9.32E-04 |
| ENSG00000171522 | *PTGER4* | rs1475357493 | 5:40332634 | 0.48 | 5.04 | 1.09E-06 | 9.41E-04 |
| ENSG00000171522 | *PTGER4* | rs1384183470 | 5:40438435 | 0.53 | 5.04 | 1.09E-06 | 9.43E-04 |
| ENSG00000169071 | *ROR2* | rs3847308 | 9:94800146 | 0.49 | 5.04 | 1.10E-06 | 9.53E-04 |
| ENSG00000169071 | *ROR2* | rs10992137 | 9:94619103 | 0.52 | 5.04 | 1.12E-06 | 9.67E-04 |
| ENSG00000169071 | *ROR2* | rs10992143 | 9:94628882 | 0.52 | 5.03 | 1.13E-06 | 9.72E-04 |
| ENSG00000169071 | *ROR2* | rs10992126 | 9:94596450 | 0.51 | 5.03 | 1.13E-06 | 9.74E-04 |
| ENSG00000169071 | *ROR2* | rs1881385 | 9:94596781 | 0.51 | 5.03 | 1.13E-06 | 9.74E-04 |
| ENSG00000169071 | *ROR2* | rs12378938 | 9:94594115 | 0.51 | 5.03 | 1.13E-06 | 9.74E-04 |
| ENSG00000169071 | *ROR2* | rs1028728977 | 9:94592511 | 0.51 | 5.03 | 1.13E-06 | 9.74E-04 |
| ENSG00000169071 | *ROR2* | rs1292778936 | 9:94592975 | 0.51 | 5.03 | 1.13E-06 | 9.75E-04 |
| ENSG00000169071 | *ROR2* | rs4744107 | 9:94628329 | 0.50 | 5.03 | 1.14E-06 | 9.82E-04 |
| ENSG00000171522 | *PTGER4* | rs7707395 | 5:40343533 | 0.48 | 5.03 | 1.15E-06 | 9.89E-04 |
| ENSG00000169071 | *ROR2* | rs2312735 | 9:94605908 | 0.51 | 5.03 | 1.16E-06 | 9.94E-04 |
| ENSG00000228716 | *DHFR* | rs11952744 | 5:79926897 | -0.58 | -5.02 | 1.18E-06 | 1.01E-03 |
| ENSG00000228716 | *DHFR* | rs1650714 | 5:79931112 | -0.58 | -5.02 | 1.18E-06 | 1.01E-03 |
| ENSG00000169071 | *ROR2* | rs2312733 | 9:94586200 | 0.51 | 5.02 | 1.18E-06 | 1.01E-03 |
| ENSG00000169071 | *ROR2* | rs10820908 | 9:94640935 | 0.52 | 5.02 | 1.19E-06 | 1.01E-03 |
| ENSG00000169071 | *ROR2* | rs10992130 | 9:94611390 | 0.52 | 5.02 | 1.19E-06 | 1.01E-03 |
| ENSG00000228716 | *DHFR* | rs35086803 | 5:79954075 | -0.58 | -5.02 | 1.20E-06 | 1.02E-03 |
| ENSG00000228716 | *DHFR* | rs141508315 | 5:79933653 | -0.58 | -5.02 | 1.22E-06 | 1.03E-03 |
| ENSG00000149328 | *GLB1L2* | rs4936230 | 11:134235220 | 0.49 | 5.02 | 1.23E-06 | 1.04E-03 |
| ENSG00000149328 | *GLB1L2* | rs4937877 | 11:134235392 | 0.49 | 5.02 | 1.23E-06 | 1.04E-03 |
| ENSG00000169071 | *ROR2* | rs62564596 | 9:94610981 | 0.52 | 5.02 | 1.23E-06 | 1.04E-03 |
| ENSG00000169071 | *ROR2* | rs62564597 | 9:94610982 | 0.52 | 5.02 | 1.23E-06 | 1.04E-03 |
| ENSG00000169071 | *ROR2* | rs2840334 | 9:94733023 | 0.50 | 5.01 | 1.23E-06 | 1.05E-03 |
| ENSG00000169071 | *ROR2* | rs2312739 | 9:94733024 | 0.49 | 5.01 | 1.24E-06 | 1.05E-03 |
| ENSG00000169071 | *ROR2* | rs146512465 | 9:94612533 | 0.52 | 5.01 | 1.24E-06 | 1.05E-03 |
| ENSG00000169071 | *ROR2* | rs10820900 | 9:94495608 | -0.48 | -5.01 | 1.24E-06 | 1.05E-03 |
| ENSG00000170369 | *CST2* | rs6049126 | 20:23772654 | -0.50 | -5.01 | 1.24E-06 | 1.05E-03 |
| ENSG00000169071 | *ROR2* | rs148943824 | 9:94612694 | 0.52 | 5.01 | 1.24E-06 | 1.05E-03 |
| ENSG00000170369 | *CST2* | rs6132662 | 20:23770556 | -0.50 | -5.01 | 1.25E-06 | 1.05E-03 |
| ENSG00000170369 | *CST2* | rs6106716 | 20:23770462 | -0.50 | -5.01 | 1.25E-06 | 1.05E-03 |
| ENSG00000169071 | *ROR2* | rs7847061 | 9:94613202 | 0.51 | 5.01 | 1.27E-06 | 1.07E-03 |
| ENSG00000169071 | *ROR2* | rs10992139 | 9:94619798 | 0.52 | 5.01 | 1.27E-06 | 1.07E-03 |
| ENSG00000228716 | *DHFR* | rs10168 | 5:79950403 | -0.58 | -5.01 | 1.28E-06 | 1.08E-03 |
| ENSG00000228716 | *DHFR* | rs58063248 | 5:79940605 | -0.58 | -5.01 | 1.28E-06 | 1.08E-03 |
| ENSG00000228716 | *DHFR* | rs1650715 | 5:79930741 | -0.58 | -5.01 | 1.28E-06 | 1.08E-03 |
| ENSG00000228716 | *DHFR* | rs56147256 | 5:79940560 | -0.58 | -5.01 | 1.29E-06 | 1.09E-03 |
| ENSG00000170369 | *CST2* | rs6114281 | 20:23767453 | -0.50 | -5.01 | 1.29E-06 | 1.09E-03 |
| ENSG00000228716 | *DHFR* | rs1643649 | 5:79939449 | -0.58 | -5.00 | 1.29E-06 | 1.09E-03 |
| ENSG00000228716 | *DHFR* | rs1644201 | 5:80112002 | 0.49 | 5.00 | 1.30E-06 | 1.10E-03 |
| ENSG00000228716 | *DHFR* | rs59117994 | 5:79926340 | -0.58 | -5.00 | 1.32E-06 | 1.11E-03 |
| ENSG00000127325 | *BEST3* | rs111233078 | 12:70075718 | 0.49 | 5.00 | 1.32E-06 | 1.11E-03 |
| ENSG00000169071 | *ROR2* | rs10820905 | 9:94584121 | 0.51 | 5.00 | 1.33E-06 | 1.12E-03 |
| ENSG00000228716 | *DHFR* | rs112878595 | 5:79926274 | -0.58 | -5.00 | 1.33E-06 | 1.12E-03 |
| ENSG00000228716 | *DHFR* | rs57771284 | 5:79926423 | -0.58 | -5.00 | 1.33E-06 | 1.12E-03 |
| ENSG00000228716 | *DHFR* | rs60244599 | 5:79926427 | -0.58 | -5.00 | 1.33E-06 | 1.12E-03 |
| ENSG00000228716 | *DHFR* | rs58490646 | 5:79940534 | -0.58 | -5.00 | 1.34E-06 | 1.12E-03 |
| ENSG00000171522 | *PTGER4* | rs6897022 | 5:40336546 | 0.48 | 5.00 | 1.34E-06 | 1.12E-03 |
| ENSG00000169071 | *ROR2* | rs1195781142 | 9:94736276 | 0.50 | 5.00 | 1.35E-06 | 1.13E-03 |
| ENSG00000228716 | *DHFR* | rs1053136 | 5:79922349 | -0.58 | -4.99 | 1.36E-06 | 1.14E-03 |
| ENSG00000228716 | *DHFR* | rs7387 | 5:79924791 | -0.58 | -4.99 | 1.36E-06 | 1.14E-03 |
| ENSG00000228716 | *DHFR* | rs2618372 | 5:79925610 | -0.58 | -4.99 | 1.36E-06 | 1.14E-03 |
| ENSG00000228716 | *DHFR* | rs1623489 | 5:79926115 | -0.58 | -4.99 | 1.36E-06 | 1.14E-03 |
| ENSG00000228716 | *DHFR* | rs1677636 | 5:79926126 | -0.58 | -4.99 | 1.36E-06 | 1.14E-03 |
| ENSG00000228716 | *DHFR* | rs1650718 | 5:79930276 | -0.58 | -4.99 | 1.36E-06 | 1.14E-03 |
| ENSG00000228716 | *DHFR* | rs1677706 | 5:79930303 | -0.58 | -4.99 | 1.36E-06 | 1.14E-03 |
| ENSG00000228716 | *DHFR* | rs1677704 | 5:79931061 | -0.58 | -4.99 | 1.36E-06 | 1.14E-03 |
| ENSG00000228716 | *DHFR* | rs1677701 | 5:79931332 | -0.58 | -4.99 | 1.36E-06 | 1.14E-03 |
| ENSG00000228716 | *DHFR* | rs1403747583 | 5:79931419 | -0.58 | -4.99 | 1.36E-06 | 1.14E-03 |
| ENSG00000228716 | *DHFR* | rs67837398 | 5:79932496 | -0.58 | -4.99 | 1.36E-06 | 1.14E-03 |
| ENSG00000228716 | *DHFR* | rs71578847 | 5:79932504 | -0.58 | -4.99 | 1.36E-06 | 1.14E-03 |
| ENSG00000228716 | *DHFR* | rs1643662 | 5:79933235 | -0.58 | -4.99 | 1.36E-06 | 1.14E-03 |
| ENSG00000228716 | *DHFR* | rs1643661 | 5:79933576 | -0.58 | -4.99 | 1.36E-06 | 1.14E-03 |
| ENSG00000228716 | *DHFR* | rs1643660 | 5:79934027 | -0.58 | -4.99 | 1.36E-06 | 1.14E-03 |
| ENSG00000228716 | *DHFR* | rs1643659 | 5:79934836 | -0.58 | -4.99 | 1.36E-06 | 1.14E-03 |
| ENSG00000228716 | *DHFR* | rs1677696 | 5:79935017 | -0.58 | -4.99 | 1.36E-06 | 1.14E-03 |
| ENSG00000228716 | *DHFR* | rs1650712 | 5:79935513 | -0.58 | -4.99 | 1.36E-06 | 1.14E-03 |
| ENSG00000228716 | *DHFR* | rs1650710 | 5:79936161 | -0.58 | -4.99 | 1.36E-06 | 1.14E-03 |
| ENSG00000228716 | *DHFR* | rs1677634 | 5:79928324 | -0.58 | -4.99 | 1.36E-06 | 1.14E-03 |
| ENSG00000228716 | *DHFR* | rs1643636 | 5:79928767 | -0.58 | -4.99 | 1.36E-06 | 1.14E-03 |
| ENSG00000228716 | *DHFR* | rs1643637 | 5:79928909 | -0.58 | -4.99 | 1.36E-06 | 1.14E-03 |
| ENSG00000228716 | *DHFR* | rs2560424 | 5:79925305 | -0.58 | -4.99 | 1.36E-06 | 1.14E-03 |
| ENSG00000228716 | *DHFR* | rs1677633 | 5:79928224 | -0.58 | -4.99 | 1.37E-06 | 1.14E-03 |
| ENSG00000228716 | *DHFR* | rs1650711 | 5:79935843 | -0.58 | -4.99 | 1.37E-06 | 1.15E-03 |
| ENSG00000228716 | *DHFR* | rs1643634 | 5:79927899 | -0.58 | -4.99 | 1.37E-06 | 1.15E-03 |
| ENSG00000228716 | *DHFR* | rs1471777243 | 5:79929174 | -0.58 | -4.99 | 1.37E-06 | 1.15E-03 |
| ENSG00000228716 | *DHFR* | rs1643655 | 5:79953776 | -0.58 | -4.99 | 1.37E-06 | 1.15E-03 |
| ENSG00000228716 | *DHFR* | rs1677684 | 5:79939053 | -0.58 | -4.99 | 1.37E-06 | 1.15E-03 |
| ENSG00000228716 | *DHFR* | rs1677666 | 5:79921825 | -0.58 | -4.99 | 1.37E-06 | 1.15E-03 |
| ENSG00000228716 | *DHFR* | rs1643650 | 5:79940143 | -0.58 | -4.99 | 1.37E-06 | 1.15E-03 |
| ENSG00000228716 | *DHFR* | rs1643657 | 5:79936417 | -0.58 | -4.99 | 1.37E-06 | 1.15E-03 |
| ENSG00000228716 | *DHFR* | rs966762 | 5:79936799 | -0.58 | -4.99 | 1.37E-06 | 1.15E-03 |
| ENSG00000228716 | *DHFR* | rs1677691 | 5:79937028 | -0.58 | -4.99 | 1.37E-06 | 1.15E-03 |
| ENSG00000228716 | *DHFR* | rs1650709 | 5:79937174 | -0.58 | -4.99 | 1.37E-06 | 1.15E-03 |
| ENSG00000228716 | *DHFR* | rs1677689 | 5:79937722 | -0.58 | -4.99 | 1.37E-06 | 1.15E-03 |
| ENSG00000228716 | *DHFR* | rs1677688 | 5:79938007 | -0.58 | -4.99 | 1.37E-06 | 1.15E-03 |
| ENSG00000228716 | *DHFR* | rs1677686 | 5:79938528 | -0.58 | -4.99 | 1.37E-06 | 1.15E-03 |
| ENSG00000228716 | *DHFR* | rs1643647 | 5:79938568 | -0.58 | -4.99 | 1.37E-06 | 1.15E-03 |
| ENSG00000228716 | *DHFR* | rs1643648 | 5:79938615 | -0.58 | -4.99 | 1.37E-06 | 1.15E-03 |
| ENSG00000228716 | *DHFR* | rs1677685 | 5:79938941 | -0.58 | -4.99 | 1.37E-06 | 1.15E-03 |
| ENSG00000228716 | *DHFR* | rs844370 | 5:79943002 | -0.58 | -4.99 | 1.37E-06 | 1.15E-03 |
| ENSG00000228716 | *DHFR* | rs1643646 | 5:79948641 | -0.58 | -4.99 | 1.37E-06 | 1.15E-03 |
| ENSG00000228716 | *DHFR* | rs1677670 | 5:79948654 | -0.58 | -4.99 | 1.37E-06 | 1.15E-03 |
| ENSG00000228716 | *DHFR* | rs1677626 | 5:79949445 | -0.58 | -4.99 | 1.37E-06 | 1.15E-03 |
| ENSG00000228716 | *DHFR* | rs1478835 | 5:79949600 | -0.58 | -4.99 | 1.37E-06 | 1.15E-03 |
| ENSG00000228716 | *DHFR* | rs2250063 | 5:79950497 | -0.58 | -4.99 | 1.37E-06 | 1.15E-03 |
| ENSG00000228716 | *DHFR* | rs1272567903 | 5:79948005 | -0.58 | -4.99 | 1.37E-06 | 1.15E-03 |
| ENSG00000228716 | *DHFR* | rs1478834 | 5:79949575 | -0.58 | -4.99 | 1.37E-06 | 1.15E-03 |
| ENSG00000228716 | *DHFR* | rs1677694 | 5:79936297 | -0.58 | -4.99 | 1.37E-06 | 1.15E-03 |
| ENSG00000228716 | *DHFR* | rs1677693 | 5:79936318 | -0.58 | -4.99 | 1.37E-06 | 1.15E-03 |
| ENSG00000228716 | *DHFR* | rs1643658 | 5:79936341 | -0.58 | -4.99 | 1.37E-06 | 1.15E-03 |
| ENSG00000228716 | *DHFR* | rs13161245 | 5:79944733 | -0.58 | -4.99 | 1.37E-06 | 1.15E-03 |
| ENSG00000228716 | *DHFR* | rs836821 | 5:79945711 | -0.58 | -4.99 | 1.37E-06 | 1.15E-03 |
| ENSG00000228716 | *DHFR* | rs865647 | 5:79942276 | -0.58 | -4.99 | 1.37E-06 | 1.15E-03 |
| ENSG00000228716 | *DHFR* | rs1677692 | 5:79937014 | -0.58 | -4.99 | 1.38E-06 | 1.15E-03 |
| ENSG00000228716 | *DHFR* | rs861373 | 5:79948062 | -0.58 | -4.99 | 1.38E-06 | 1.15E-03 |
| ENSG00000228716 | *DHFR* | rs1643638 | 5:79930256 | -0.58 | -4.99 | 1.38E-06 | 1.15E-03 |
| ENSG00000136108 | *CKAP2* | rs61958050 | 13:52994427 | -0.52 | -4.99 | 1.39E-06 | 1.16E-03 |
| ENSG00000251504 | *LINC01099* | rs1711384 | 4:178990324 | -0.49 | -4.99 | 1.39E-06 | 1.16E-03 |
| ENSG00000136108 | *CKAP2* | rs4884320 | 13:53006914 | -0.52 | -4.99 | 1.39E-06 | 1.16E-03 |
| ENSG00000169071 | *ROR2* | rs4744110 | 9:94642450 | 0.51 | 4.99 | 1.39E-06 | 1.16E-03 |
| ENSG00000228716 | *DHFR* | rs1677687 | 5:79938336 | -0.58 | -4.99 | 1.40E-06 | 1.17E-03 |
| ENSG00000170369 | *CST2* | rs6049120 | 20:23765191 | -0.50 | -4.99 | 1.40E-06 | 1.17E-03 |
| ENSG00000228716 | *DHFR* | rs1255740960 | 5:79950389 | -0.58 | -4.99 | 1.41E-06 | 1.18E-03 |
| ENSG00000169071 | *ROR2* | rs60903425 | 9:94641791 | 0.51 | 4.99 | 1.41E-06 | 1.18E-03 |
| ENSG00000228716 | *DHFR* | rs865645 | 5:79944104 | -0.58 | -4.98 | 1.43E-06 | 1.19E-03 |
| ENSG00000228716 | *DHFR* | rs836823 | 5:79943714 | -0.58 | -4.98 | 1.43E-06 | 1.19E-03 |
| ENSG00000228716 | *DHFR* | rs836822 | 5:79943776 | -0.58 | -4.98 | 1.43E-06 | 1.19E-03 |
| ENSG00000169071 | *ROR2* | rs4237215 | 9:94497680 | -0.48 | -4.98 | 1.44E-06 | 1.20E-03 |
| ENSG00000136108 | *CKAP2* | rs7331730 | 13:52862914 | -0.48 | -4.98 | 1.46E-06 | 1.21E-03 |
| ENSG00000170369 | *CST2* | rs13045184 | 20:23764906 | -0.50 | -4.98 | 1.47E-06 | 1.22E-03 |
| ENSG00000228716 | *DHFR* | rs6870725 | 5:80186922 | -0.49 | -4.98 | 1.48E-06 | 1.23E-03 |
| ENSG00000228716 | *DHFR* | rs35707907 | 5:80188630 | -0.49 | -4.97 | 1.49E-06 | 1.24E-03 |
| ENSG00000228716 | *DHFR* | rs1287323682 | 5:79921897 | -0.58 | -4.97 | 1.50E-06 | 1.25E-03 |
| ENSG00000169071 | *ROR2* | rs4744109 | 9:94642340 | 0.51 | 4.97 | 1.50E-06 | 1.25E-03 |
| ENSG00000228716 | *DHFR* | rs1200017808 | 5:79931745 | -0.57 | -4.97 | 1.52E-06 | 1.26E-03 |
| ENSG00000136108 | *CKAP2* | rs11406358 | 13:52992422 | -0.51 | -4.97 | 1.52E-06 | 1.26E-03 |
| ENSG00000169071 | *ROR2* | rs10992144 | 9:94631630 | 0.50 | 4.97 | 1.54E-06 | 1.27E-03 |
| ENSG00000169071 | *ROR2* | rs7850118 | 9:94626543 | 0.50 | 4.96 | 1.55E-06 | 1.28E-03 |
| ENSG00000169071 | *ROR2* | rs7865855 | 9:94614602 | 0.51 | 4.96 | 1.55E-06 | 1.28E-03 |
| ENSG00000169071 | *ROR2* | rs7864311 | 9:94614074 | 0.51 | 4.96 | 1.55E-06 | 1.28E-03 |
| ENSG00000170369 | *CST2* | rs6114283 | 20:23767666 | -0.50 | -4.96 | 1.56E-06 | 1.29E-03 |
| ENSG00000169071 | *ROR2* | rs55864279 | 9:94627108 | 0.51 | 4.96 | 1.56E-06 | 1.29E-03 |
| ENSG00000170369 | *CST2* | rs766406679 | 20:23776795 | -0.52 | -4.96 | 1.60E-06 | 1.32E-03 |
| ENSG00000169071 | *ROR2* | rs10992132 | 9:94614626 | 0.52 | 4.96 | 1.61E-06 | 1.32E-03 |
| ENSG00000228716 | *DHFR* | rs1643630 | 5:79923292 | -0.69 | -4.95 | 1.63E-06 | 1.34E-03 |
| ENSG00000170369 | *CST2* | rs201973382 | 20:23777061 | -0.50 | -4.95 | 1.63E-06 | 1.34E-03 |
| ENSG00000228716 | *DHFR* | rs200868906 | 5:79926584 | -0.60 | -4.95 | 1.65E-06 | 1.36E-03 |
| ENSG00000169071 | *ROR2* | rs56088447 | 9:94616103 | 0.52 | 4.95 | 1.65E-06 | 1.36E-03 |
| ENSG00000228716 | *DHFR* | rs32982 | 5:80122493 | 0.48 | 4.95 | 1.66E-06 | 1.36E-03 |
| ENSG00000169071 | *ROR2* | rs7869504 | 9:94791735 | 0.49 | 4.95 | 1.67E-06 | 1.37E-03 |
| ENSG00000170369 | *CST2* | rs34571857 | 20:23764112 | -0.50 | -4.95 | 1.67E-06 | 1.37E-03 |
| ENSG00000169071 | *ROR2* | rs7847473 | 9:94600102 | 0.50 | 4.95 | 1.68E-06 | 1.37E-03 |
| ENSG00000170369 | *CST2* | rs4432530 | 20:23808534 | -0.52 | -4.94 | 1.71E-06 | 1.40E-03 |
| ENSG00000169071 | *ROR2* | rs1881392 | 9:94616030 | 0.52 | 4.94 | 1.73E-06 | 1.41E-03 |
| ENSG00000169071 | *ROR2* | rs1374207917 | 9:94589076 | 0.53 | 4.94 | 1.75E-06 | 1.43E-03 |
| ENSG00000141404 | *GNAL* | rs7237466 | 18:11698292 | 0.70 | 4.94 | 1.77E-06 | 1.44E-03 |
| ENSG00000141404 | *GNAL* | rs8087897 | 18:11697824 | 0.70 | 4.94 | 1.77E-06 | 1.44E-03 |
| ENSG00000136108 | *CKAP2* | rs9563116 | 13:53301136 | 0.53 | 4.93 | 1.78E-06 | 1.44E-03 |
| ENSG00000141404 | *GNAL* | rs10591897 | 18:11697771 | 0.70 | 4.93 | 1.78E-06 | 1.44E-03 |
| ENSG00000141404 | *GNAL* | rs7237945 | 18:11698555 | 0.70 | 4.93 | 1.79E-06 | 1.45E-03 |
| ENSG00000228716 | *DHFR* | rs1643635 | 5:79928216 | -0.57 | -4.93 | 1.79E-06 | 1.46E-03 |
| ENSG00000228716 | *DHFR* | rs836818 | 5:79947956 | -0.57 | -4.93 | 1.80E-06 | 1.46E-03 |
| ENSG00000169071 | *ROR2* | rs7039406 | 9:94617098 | 0.51 | 4.93 | 1.80E-06 | 1.46E-03 |
| ENSG00000169071 | *ROR2* | rs16907887 | 9:94607294 | 0.52 | 4.93 | 1.80E-06 | 1.46E-03 |
| ENSG00000228716 | *DHFR* | rs1222809 | 5:79917517 | -0.57 | -4.93 | 1.81E-06 | 1.47E-03 |
| ENSG00000169071 | *ROR2* | rs4744112 | 9:94648911 | 0.47 | 4.93 | 1.83E-06 | 1.48E-03 |
| ENSG00000169071 | *ROR2* | rs7037848 | 9:94866040 | 0.49 | 4.93 | 1.84E-06 | 1.49E-03 |
| ENSG00000141404 | *GNAL* | rs34487043 | 18:11698951 | 0.70 | 4.93 | 1.85E-06 | 1.50E-03 |
| ENSG00000251504 | *LINC01099* | rs1018475633 | 4:178916397 | -0.57 | -4.92 | 1.88E-06 | 1.52E-03 |
| ENSG00000228716 | *DHFR* | rs1650717 | 5:79930579 | -0.63 | -4.92 | 1.88E-06 | 1.52E-03 |
| ENSG00000170369 | *CST2* | rs6138082 | 20:23763354 | -0.49 | -4.92 | 1.90E-06 | 1.53E-03 |
| ENSG00000170369 | *CST2* | rs6138081 | 20:23763351 | -0.49 | -4.92 | 1.90E-06 | 1.53E-03 |
| ENSG00000169071 | *ROR2* | rs10761143 | 9:94835051 | 0.49 | 4.92 | 1.93E-06 | 1.55E-03 |
| ENSG00000169071 | *ROR2* | rs10739924 | 9:94835241 | 0.48 | 4.92 | 1.94E-06 | 1.56E-03 |
| ENSG00000169071 | *ROR2* | rs7850636 | 9:94613534 | 0.52 | 4.91 | 1.95E-06 | 1.57E-03 |
| ENSG00000251504 | *LINC01099* | rs11393588 | 4:178982396 | -0.52 | -4.91 | 1.97E-06 | 1.58E-03 |
| ENSG00000151470 | *C4orf33* | rs145714234 | 4:130034197 | -0.49 | -4.91 | 1.99E-06 | 1.60E-03 |
| ENSG00000228716 | *DHFR* | rs138395509 | 5:79934294 | -0.57 | -4.91 | 2.01E-06 | 1.61E-03 |
| ENSG00000170369 | *CST2* | rs6036532 | 20:23763089 | -0.49 | -4.91 | 2.03E-06 | 1.63E-03 |
| ENSG00000149328 | *GLB1L2* | rs1146190 | 11:134192256 | -0.48 | -4.90 | 2.03E-06 | 1.63E-03 |
| ENSG00000149328 | *GLB1L2* | rs1146191 | 11:134191585 | -0.48 | -4.90 | 2.06E-06 | 1.64E-03 |
| ENSG00000228716 | *DHFR* | rs4704688 | 5:80191705 | -0.48 | -4.90 | 2.06E-06 | 1.64E-03 |
| ENSG00000251504 | *LINC01099* | rs1341157227 | 4:178970020 | -0.55 | -4.90 | 2.07E-06 | 1.65E-03 |
| ENSG00000170369 | *CST2* | rs6138127 | 20:23809107 | -0.52 | -4.90 | 2.11E-06 | 1.68E-03 |
| ENSG00000136108 | *CKAP2* | rs11148246 | 13:52797537 | -0.48 | -4.89 | 2.19E-06 | 1.74E-03 |
| ENSG00000169071 | *ROR2* | rs10992134 | 9:94615230 | 0.50 | 4.89 | 2.20E-06 | 1.74E-03 |
| ENSG00000169071 | *ROR2* | rs55663665 | 9:94584273 | 0.51 | 4.89 | 2.21E-06 | 1.76E-03 |
| ENSG00000169071 | *ROR2* | rs55962255 | 9:94584275 | 0.51 | 4.89 | 2.21E-06 | 1.76E-03 |
| ENSG00000169071 | *ROR2* | rs12685595 | 9:94669125 | 0.47 | 4.89 | 2.22E-06 | 1.76E-03 |
| ENSG00000151470 | *C4orf33* | rs1167150261 | 4:130015456 | -0.50 | -4.88 | 2.23E-06 | 1.77E-03 |
| ENSG00000169071 | *ROR2* | rs11420421 | 9:94601529 | 0.50 | 4.88 | 2.29E-06 | 1.81E-03 |
| ENSG00000141404 | *GNAL* | rs1013459 | 18:11700534 | 0.67 | 4.88 | 2.29E-06 | 1.81E-03 |
| ENSG00000169071 | *ROR2* | rs10992124 | 9:94584452 | 0.51 | 4.88 | 2.31E-06 | 1.82E-03 |
| ENSG00000228716 | *DHFR* | rs245362 | 5:80141214 | 0.46 | 4.88 | 2.32E-06 | 1.83E-03 |
| ENSG00000169071 | *ROR2* | rs7870902 | 9:94597812 | 0.50 | 4.87 | 2.35E-06 | 1.85E-03 |
| ENSG00000170369 | *CST2* | rs6049119 | 20:23762616 | -0.49 | -4.87 | 2.39E-06 | 1.88E-03 |
| ENSG00000149328 | *GLB1L2* | rs1146188 | 11:134197635 | -0.48 | -4.87 | 2.41E-06 | 1.89E-03 |
| ENSG00000170369 | *CST2* | rs62207515 | 20:23768776 | -0.51 | -4.86 | 2.47E-06 | 1.94E-03 |
| ENSG00000170369 | *CST2* | rs58314206 | 20:23806929 | -0.51 | -4.86 | 2.48E-06 | 1.94E-03 |
| ENSG00000170369 | *CST2* | rs6138077 | 20:23762380 | -0.49 | -4.85 | 2.56E-06 | 2.00E-03 |
| ENSG00000151470 | *C4orf33* | rs530686 | 4:130039884 | -0.49 | -4.85 | 2.58E-06 | 2.01E-03 |
| ENSG00000149328 | *GLB1L2* | rs4262740 | 11:134234406 | 0.57 | 4.85 | 2.58E-06 | 2.01E-03 |
| ENSG00000170369 | *CST2* | rs6132659 | 20:23763211 | -0.48 | -4.85 | 2.64E-06 | 2.05E-03 |
| ENSG00000148344 | *PTGES* | rs1382622533 | 9:132045384 | 0.58 | 4.85 | 2.64E-06 | 2.05E-03 |
| ENSG00000169071 | *ROR2* | rs7045226 | 9:94621055 | 0.49 | 4.84 | 2.67E-06 | 2.07E-03 |
| ENSG00000169071 | *ROR2* | rs7030661 | 9:94621265 | 0.49 | 4.84 | 2.69E-06 | 2.09E-03 |
| ENSG00000169071 | *ROR2* | rs12552292 | 9:94589441 | 0.51 | 4.84 | 2.76E-06 | 2.14E-03 |
| ENSG00000149328 | *GLB1L2* | rs7113199 | 11:134247187 | 0.47 | 4.84 | 2.77E-06 | 2.14E-03 |
| ENSG00000149328 | *GLB1L2* | rs11223774 | 11:134247315 | 0.47 | 4.83 | 2.84E-06 | 2.19E-03 |
| ENSG00000169071 | *ROR2* | rs10992140 | 9:94621866 | 0.50 | 4.83 | 2.90E-06 | 2.23E-03 |
| ENSG00000169071 | *ROR2* | rs7859570 | 9:94869687 | 0.48 | 4.82 | 2.92E-06 | 2.25E-03 |
| ENSG00000169071 | *ROR2* | rs7855417 | 9:94594626 | 0.50 | 4.82 | 2.94E-06 | 2.26E-03 |
| ENSG00000169071 | *ROR2* | rs4639579 | 9:94595355 | 0.50 | 4.82 | 2.94E-06 | 2.26E-03 |
| ENSG00000169071 | *ROR2* | rs7867707 | 9:94597646 | 0.50 | 4.82 | 2.94E-06 | 2.26E-03 |
| ENSG00000169071 | *ROR2* | rs10992128 | 9:94599967 | 0.50 | 4.82 | 2.98E-06 | 2.29E-03 |
| ENSG00000170369 | *CST2* | rs6138080 | 20:23763219 | -0.49 | -4.82 | 3.01E-06 | 2.31E-03 |
| ENSG00000169071 | *ROR2* | rs10992129 | 9:94600956 | 0.50 | 4.82 | 3.01E-06 | 2.31E-03 |
| ENSG00000169071 | *ROR2* | rs56167549 | 9:94600994 | 0.50 | 4.82 | 3.02E-06 | 2.31E-03 |
| ENSG00000169071 | *ROR2* | rs7863061 | 9:94601472 | 0.50 | 4.82 | 3.04E-06 | 2.33E-03 |
| ENSG00000169071 | *ROR2* | rs6479376 | 9:94609030 | 0.50 | 4.81 | 3.07E-06 | 2.35E-03 |
| ENSG00000169071 | *ROR2* | rs1534531 | 9:94607154 | 0.50 | 4.81 | 3.10E-06 | 2.37E-03 |
| ENSG00000228716 | *DHFR* | rs245343 | 5:80157824 | 0.46 | 4.81 | 3.10E-06 | 2.37E-03 |
| ENSG00000228716 | *DHFR* | rs1391360576 | 5:79912887 | -0.52 | -4.81 | 3.12E-06 | 2.38E-03 |
| ENSG00000228716 | *DHFR* | rs1340311881 | 5:79899375 | -0.58 | -4.81 | 3.13E-06 | 2.38E-03 |
| ENSG00000228716 | *DHFR* | rs245345 | 5:80156304 | 0.46 | 4.81 | 3.14E-06 | 2.39E-03 |
| ENSG00000149328 | *GLB1L2* | rs7112912 | 11:134246900 | 0.46 | 4.81 | 3.14E-06 | 2.39E-03 |
| ENSG00000228716 | *DHFR* | rs245351 | 5:80153541 | 0.46 | 4.81 | 3.14E-06 | 2.39E-03 |
| ENSG00000151470 | *C4orf33* | rs337276 | 4:130030944 | -0.49 | -4.81 | 3.15E-06 | 2.40E-03 |
| ENSG00000228716 | *DHFR* | rs245378 | 5:80132033 | 0.46 | 4.80 | 3.22E-06 | 2.45E-03 |
| ENSG00000151470 | *C4orf33* | rs139907008 | 4:129948304 | -0.55 | -4.80 | 3.22E-06 | 2.45E-03 |
| ENSG00000169071 | *ROR2* | rs397723900 | 9:94616290 | 0.51 | 4.80 | 3.26E-06 | 2.48E-03 |
| ENSG00000228716 | *DHFR* | rs245346 | 5:80156059 | 0.46 | 4.80 | 3.27E-06 | 2.48E-03 |
| ENSG00000228716 | *DHFR* | rs32993 | 5:80114131 | 0.46 | 4.80 | 3.27E-06 | 2.48E-03 |
| ENSG00000228716 | *DHFR* | rs864133 | 5:79942207 | -0.59 | -4.80 | 3.32E-06 | 2.51E-03 |
| ENSG00000171522 | *PTGER4* | rs10043093 | 5:40324220 | 0.46 | 4.79 | 3.37E-06 | 2.54E-03 |
| ENSG00000169071 | *ROR2* | rs2841691 | 9:94605469 | 0.50 | 4.79 | 3.39E-06 | 2.56E-03 |
| ENSG00000228716 | *DHFR* | rs958575 | 5:80206473 | -0.47 | -4.79 | 3.41E-06 | 2.57E-03 |
| ENSG00000127325 | *BEST3* | rs57897082 | 12:70075626 | 0.48 | 4.79 | 3.42E-06 | 2.58E-03 |
| ENSG00000169071 | *ROR2* | rs12683181 | 9:94518328 | -0.48 | -4.79 | 3.43E-06 | 2.58E-03 |
| ENSG00000228716 | *DHFR* | rs3839309 | 5:80073140 | 0.49 | 4.79 | 3.44E-06 | 2.60E-03 |
| ENSG00000127325 | *BEST3* | rs58715753 | 12:70075501 | 0.48 | 4.79 | 3.46E-06 | 2.60E-03 |
| ENSG00000169071 | *ROR2* | rs12684533 | 9:94638962 | 0.48 | 4.78 | 3.52E-06 | 2.65E-03 |
| ENSG00000127325 | *BEST3* | rs775434 | 12:70094505 | -0.49 | -4.78 | 3.54E-06 | 2.66E-03 |
| ENSG00000228716 | *DHFR* | rs32991 | 5:80114402 | 0.46 | 4.78 | 3.58E-06 | 2.69E-03 |
| ENSG00000149328 | *GLB1L2* | rs548465 | 11:134196849 | -0.49 | -4.77 | 3.64E-06 | 2.72E-03 |
| ENSG00000151470 | *C4orf33* | rs10529563 | 4:129738454 | -0.63 | -4.77 | 3.68E-06 | 2.75E-03 |
| ENSG00000251504 | *LINC01099* | rs1676197 | 4:178982549 | -0.51 | -4.77 | 3.70E-06 | 2.77E-03 |
| ENSG00000169071 | *ROR2* | rs6479379 | 9:94648302 | 0.47 | 4.77 | 3.73E-06 | 2.79E-03 |
| ENSG00000169071 | *ROR2* | rs7867567 | 9:94648219 | 0.47 | 4.77 | 3.74E-06 | 2.79E-03 |
| ENSG00000170369 | *CST2* | rs7264667 | 20:23761395 | -0.48 | -4.77 | 3.74E-06 | 2.79E-03 |
| ENSG00000067798 | *NAV3* | rs17792562 | 12:77986460 | 0.54 | 4.77 | 3.75E-06 | 2.80E-03 |
| ENSG00000067798 | *NAV3* | rs17792514 | 12:77986403 | 0.54 | 4.77 | 3.75E-06 | 2.80E-03 |
| ENSG00000171522 | *PTGER4* | rs6883686 | 5:40402533 | -0.44 | -4.77 | 3.79E-06 | 2.83E-03 |
| ENSG00000228716 | *DHFR* | rs33013 | 5:80060016 | 0.48 | 4.76 | 3.81E-06 | 2.84E-03 |
| ENSG00000169071 | *ROR2* | rs10992123 | 9:94582873 | 0.51 | 4.76 | 3.82E-06 | 2.84E-03 |
| ENSG00000067798 | *NAV3* | rs11105822 | 12:77986239 | 0.54 | 4.76 | 3.88E-06 | 2.88E-03 |
| ENSG00000136108 | *CKAP2* | rs6561664 | 13:52912099 | 0.44 | 4.76 | 3.95E-06 | 2.93E-03 |
| ENSG00000228716 | *DHFR* | rs372735391 | 5:80203678 | -0.61 | -4.76 | 3.96E-06 | 2.94E-03 |
| ENSG00000169071 | *ROR2* | rs6479377 | 9:94640884 | 0.48 | 4.75 | 3.97E-06 | 2.94E-03 |
| ENSG00000228716 | *DHFR* | rs33014 | 5:80061689 | 0.48 | 4.75 | 4.05E-06 | 2.99E-03 |
| ENSG00000067798 | *NAV3* | rs11105832 | 12:77988123 | 0.54 | 4.75 | 4.07E-06 | 3.01E-03 |
| ENSG00000228716 | *DHFR* | rs26778 | 5:80035750 | 0.48 | 4.75 | 4.07E-06 | 3.01E-03 |
| ENSG00000228716 | *DHFR* | rs245012 | 5:80048211 | 0.48 | 4.75 | 4.07E-06 | 3.01E-03 |
| ENSG00000228716 | *DHFR* | rs151887 | 5:80049520 | 0.48 | 4.75 | 4.07E-06 | 3.01E-03 |
| ENSG00000169071 | *ROR2* | rs10761135 | 9:94647024 | 0.46 | 4.75 | 4.10E-06 | 3.03E-03 |
| ENSG00000228716 | *DHFR* | rs245011 | 5:80049626 | 0.48 | 4.75 | 4.11E-06 | 3.03E-03 |
| ENSG00000151470 | *C4orf33* | rs4975265 | 4:129738210 | -0.62 | -4.75 | 4.11E-06 | 3.03E-03 |
| ENSG00000171522 | *PTGER4* | rs10512732 | 5:40319919 | 0.46 | 4.75 | 4.13E-06 | 3.04E-03 |
| ENSG00000169071 | *ROR2* | rs62565672 | 9:94629940 | 0.57 | 4.75 | 4.13E-06 | 3.05E-03 |
| ENSG00000169071 | *ROR2* | rs200750836 | 9:94666331 | 0.62 | 4.74 | 4.15E-06 | 3.06E-03 |
| ENSG00000228716 | *DHFR* | rs1017574 | 5:80211179 | -0.47 | -4.74 | 4.17E-06 | 3.07E-03 |
| ENSG00000228716 | *DHFR* | rs33015 | 5:80063548 | 0.48 | 4.74 | 4.24E-06 | 3.11E-03 |
| ENSG00000228716 | *DHFR* | rs10067194 | 5:80211574 | -0.47 | -4.74 | 4.25E-06 | 3.12E-03 |
| ENSG00000169071 | *ROR2* | rs1528363 | 9:94639795 | 0.47 | 4.73 | 4.45E-06 | 3.25E-03 |
| ENSG00000169071 | *ROR2* | rs11795130 | 9:94664004 | 0.46 | 4.73 | 4.45E-06 | 3.25E-03 |
| ENSG00000169071 | *ROR2* | rs62565675 | 9:94639421 | 0.47 | 4.73 | 4.47E-06 | 3.26E-03 |
| ENSG00000228716 | *DHFR* | rs1349209473 | 5:79931282 | -0.56 | -4.73 | 4.49E-06 | 3.27E-03 |
| ENSG00000169071 | *ROR2* | rs1528364 | 9:94639829 | 0.47 | 4.73 | 4.52E-06 | 3.29E-03 |
| ENSG00000169071 | *ROR2* | rs12684524 | 9:94638804 | 0.47 | 4.73 | 4.52E-06 | 3.30E-03 |
| ENSG00000169071 | *ROR2* | rs1534533 | 9:94645971 | 0.48 | 4.72 | 4.55E-06 | 3.31E-03 |
| ENSG00000149328 | *GLB1L2* | rs201665159 | 11:134199507 | -0.50 | -4.72 | 4.61E-06 | 3.35E-03 |
| ENSG00000151470 | *C4orf33* | rs140060997 | 4:129937502 | -0.66 | -4.72 | 4.62E-06 | 3.36E-03 |
| ENSG00000251504 | *LINC01099* | rs1676199 | 4:178984427 | -0.50 | -4.72 | 4.67E-06 | 3.39E-03 |
| ENSG00000169071 | *ROR2* | rs10761132 | 9:94642129 | 0.47 | 4.71 | 4.75E-06 | 3.44E-03 |
| ENSG00000169071 | *ROR2* | rs7870049 | 9:94645758 | 0.47 | 4.71 | 4.75E-06 | 3.44E-03 |
| ENSG00000127325 | *BEST3* | rs12425422 | 12:70078575 | 0.47 | 4.71 | 4.76E-06 | 3.45E-03 |
| ENSG00000127325 | *BEST3* | rs710711 | 12:70082357 | -0.47 | -4.71 | 4.76E-06 | 3.45E-03 |
| ENSG00000127325 | *BEST3* | rs60954668 | 12:70079387 | 0.47 | 4.71 | 4.76E-06 | 3.45E-03 |
| ENSG00000169071 | *ROR2* | rs202208617 | 9:94724875 | 0.49 | 4.71 | 4.76E-06 | 3.45E-03 |
| ENSG00000169071 | *ROR2* | rs1609292 | 9:94725872 | 0.49 | 4.71 | 4.81E-06 | 3.48E-03 |
| ENSG00000169071 | *ROR2* | rs10761137 | 9:94728959 | 0.49 | 4.71 | 4.82E-06 | 3.49E-03 |
| ENSG00000170369 | *CST2* | rs62207514 | 20:23768722 | -0.51 | -4.71 | 4.84E-06 | 3.50E-03 |
| ENSG00000169071 | *ROR2* | rs4744111 | 9:94642762 | 0.47 | 4.71 | 4.87E-06 | 3.52E-03 |
| ENSG00000169071 | *ROR2* | rs10820910 | 9:94644814 | 0.47 | 4.71 | 4.88E-06 | 3.52E-03 |
| ENSG00000251504 | *LINC01099* | rs1676187 | 4:178978140 | -0.51 | -4.70 | 4.96E-06 | 3.57E-03 |
| ENSG00000169071 | *ROR2* | rs72746246 | 9:94661537 | 0.54 | 4.70 | 4.97E-06 | 3.58E-03 |
| ENSG00000136108 | *CKAP2* | rs7334583 | 13:52930352 | 0.44 | 4.70 | 4.99E-06 | 3.59E-03 |
| ENSG00000251504 | *LINC01099* | rs1585573 | 4:178978446 | -0.51 | -4.70 | 4.99E-06 | 3.59E-03 |
| ENSG00000251504 | *LINC01099* | rs1585574 | 4:178978448 | -0.51 | -4.70 | 4.99E-06 | 3.59E-03 |
| ENSG00000251504 | *LINC01099* | rs1620511 | 4:178978661 | -0.51 | -4.70 | 5.01E-06 | 3.60E-03 |
| ENSG00000134278 | *SPIRE1* | rs202162952 | 18:12577243 | 0.56 | 4.70 | 5.06E-06 | 3.63E-03 |
| ENSG00000251504 | *LINC01099* | rs1676192 | 4:178980830 | -0.51 | -4.69 | 5.20E-06 | 3.72E-03 |
| ENSG00000141404 | *GNAL* | rs1491408169 | 18:11695920 | 0.57 | 4.69 | 5.23E-06 | 3.74E-03 |
| ENSG00000127325 | *BEST3* | rs775420 | 12:70084136 | -0.47 | -4.69 | 5.25E-06 | 3.76E-03 |
| ENSG00000151470 | *C4orf33* | rs145864533 | 4:129796194 | 0.63 | 4.69 | 5.30E-06 | 3.79E-03 |
| ENSG00000127325 | *BEST3* | rs775422 | 12:70084795 | -0.47 | -4.68 | 5.45E-06 | 3.88E-03 |
| ENSG00000151470 | *C4orf33* | rs2217023 | 4:129732099 | -0.64 | -4.68 | 5.57E-06 | 3.95E-03 |
| ENSG00000127325 | *BEST3* | rs11836914 | 12:70085444 | 0.47 | 4.68 | 5.57E-06 | 3.95E-03 |
| ENSG00000127325 | *BEST3* | rs11836953 | 12:70085445 | 0.47 | 4.68 | 5.57E-06 | 3.95E-03 |
| ENSG00000228716 | *DHFR* | rs7705816 | 5:80191800 | -0.56 | -4.68 | 5.58E-06 | 3.96E-03 |
| ENSG00000127325 | *BEST3* | rs138223084 | 12:70092269 | 0.48 | 4.67 | 5.64E-06 | 3.99E-03 |
| ENSG00000228716 | *DHFR* | rs1643645 | 5:79948540 | -0.58 | -4.67 | 5.78E-06 | 4.08E-03 |
| ENSG00000148344 | *PTGES* | rs17456931 | 9:132046373 | 0.54 | 4.67 | 5.85E-06 | 4.12E-03 |
| ENSG00000151470 | *C4orf33* | rs10008927 | 4:129729239 | -0.64 | -4.67 | 5.86E-06 | 4.13E-03 |
| ENSG00000169071 | *ROR2* | rs1919103 | 9:94736589 | 0.49 | 4.66 | 5.93E-06 | 4.17E-03 |
| ENSG00000148344 | *PTGES* | rs17456938 | 9:132046491 | 0.54 | 4.66 | 6.01E-06 | 4.22E-03 |
| ENSG00000127325 | *BEST3* | rs775425 | 12:70086890 | -0.47 | -4.66 | 6.01E-06 | 4.22E-03 |
| ENSG00000151470 | *C4orf33* | rs6857659 | 4:129763370 | -0.64 | -4.66 | 6.02E-06 | 4.23E-03 |
| ENSG00000170369 | *CST2* | rs6036530 | 20:23759983 | -0.47 | -4.66 | 6.03E-06 | 4.23E-03 |
| ENSG00000067798 | *NAV3* | rs10506755 | 12:77993785 | 0.55 | 4.66 | 6.11E-06 | 4.28E-03 |
| ENSG00000067798 | *NAV3* | rs75130357 | 12:77993717 | 0.55 | 4.65 | 6.15E-06 | 4.31E-03 |
| ENSG00000067798 | *NAV3* | rs11105859 | 12:77993606 | 0.55 | 4.65 | 6.22E-06 | 4.35E-03 |
| ENSG00000067798 | *NAV3* | rs11105857 | 12:77993568 | 0.55 | 4.65 | 6.24E-06 | 4.37E-03 |
| ENSG00000170369 | *CST2* | rs1248490673 | 20:23759878 | -0.47 | -4.65 | 6.26E-06 | 4.38E-03 |
| ENSG00000151470 | *C4orf33* | rs7687562 | 4:129739899 | -0.63 | -4.65 | 6.28E-06 | 4.39E-03 |
| ENSG00000151470 | *C4orf33* | rs4975269 | 4:129752134 | -0.63 | -4.65 | 6.35E-06 | 4.43E-03 |
| ENSG00000228716 | *DHFR* | rs1643664 | 5:79931771 | -0.54 | -4.64 | 6.52E-06 | 4.54E-03 |
| ENSG00000169071 | *ROR2* | rs10820907 | 9:94628846 | 0.49 | 4.64 | 6.54E-06 | 4.55E-03 |
| ENSG00000169071 | *ROR2* | rs7047587 | 9:94655021 | 0.44 | 4.64 | 6.61E-06 | 4.59E-03 |
| ENSG00000169071 | *ROR2* | rs3935603 | 9:94508505 | -0.44 | -4.63 | 6.71E-06 | 4.65E-03 |
| ENSG00000186340 | *THBS2* | rs55762318 | 6:169158402 | 1.82 | 4.63 | 6.72E-06 | 4.65E-03 |
| ENSG00000136108 | *CKAP2* | rs56033750 | 13:52747278 | -0.44 | -4.63 | 6.81E-06 | 4.71E-03 |
| ENSG00000184160 | *ADRA2C* | rs74499803 | 4:4367086 | -0.86 | -4.63 | 6.89E-06 | 4.76E-03 |
| ENSG00000136108 | *CKAP2* | rs61957257 | 13:52769702 | -0.44 | -4.63 | 6.93E-06 | 4.78E-03 |
| ENSG00000149328 | *GLB1L2* | rs483244 | 11:134164712 | -0.46 | -4.63 | 6.94E-06 | 4.79E-03 |
| ENSG00000136108 | *CKAP2* | rs1815669 | 13:52968645 | -0.46 | -4.62 | 7.10E-06 | 4.89E-03 |
| ENSG00000169071 | *ROR2* | rs10116962 | 9:94651871 | 0.44 | 4.62 | 7.10E-06 | 4.89E-03 |
| ENSG00000169071 | *ROR2* | rs7858435 | 9:94651415 | 0.44 | 4.62 | 7.22E-06 | 4.96E-03 |
| ENSG00000136108 | *CKAP2* | rs9568720 | 13:52836444 | -0.46 | -4.62 | 7.25E-06 | 4.98E-03 |
| ENSG00000067798 | *NAV3* | rs77792425 | 12:77992281 | 0.55 | 4.62 | 7.29E-06 | 5.00E-03 |
| ENSG00000170369 | *CST2* | rs6049111 | 20:23759439 | -0.47 | -4.61 | 7.36E-06 | 5.04E-03 |
| ENSG00000127325 | *BEST3* | rs112331929 | 12:70094642 | 0.47 | 4.61 | 7.42E-06 | 5.08E-03 |
| ENSG00000127325 | *BEST3* | rs775431 | 12:70093206 | -0.47 | -4.61 | 7.46E-06 | 5.10E-03 |
| ENSG00000127325 | *BEST3* | rs775430 | 12:70092210 | -0.47 | -4.61 | 7.50E-06 | 5.12E-03 |
| ENSG00000151470 | *C4orf33* | rs502319 | 4:130040702 | -0.46 | -4.61 | 7.50E-06 | 5.12E-03 |
| ENSG00000251504 | *LINC01099* | rs1381699 | 4:178983792 | -0.48 | -4.61 | 7.54E-06 | 5.15E-03 |
| ENSG00000127325 | *BEST3* | rs17225559 | 12:70090463 | 0.46 | 4.61 | 7.55E-06 | 5.15E-03 |
| ENSG00000067798 | *NAV3* | rs76978241 | 12:77992009 | 0.55 | 4.61 | 7.58E-06 | 5.17E-03 |
| ENSG00000169071 | *ROR2* | rs10992135 | 9:94617450 | 0.49 | 4.61 | 7.58E-06 | 5.17E-03 |
| ENSG00000151470 | *C4orf33* | rs337273 | 4:130041532 | -0.46 | -4.61 | 7.60E-06 | 5.18E-03 |
| ENSG00000170369 | *CST2* | rs4640452 | 20:23756613 | -0.47 | -4.60 | 7.93E-06 | 5.37E-03 |
| ENSG00000169071 | *ROR2* | rs6479378 | 9:94647600 | 0.45 | 4.60 | 7.94E-06 | 5.38E-03 |
| ENSG00000136108 | *CKAP2* | rs9535895 | 13:52765847 | -0.45 | -4.60 | 7.94E-06 | 5.38E-03 |
| ENSG00000169071 | *ROR2* | rs10761134 | 9:94646983 | 0.45 | 4.59 | 7.98E-06 | 5.40E-03 |
| ENSG00000169071 | *ROR2* | rs10761133 | 9:94646958 | 0.45 | 4.59 | 8.01E-06 | 5.42E-03 |
| ENSG00000151470 | *C4orf33* | rs789982 | 4:129999071 | -0.57 | -4.59 | 8.09E-06 | 5.47E-03 |
| ENSG00000151470 | *C4orf33* | rs492565 | 4:130046137 | -0.45 | -4.59 | 8.17E-06 | 5.51E-03 |
| ENSG00000228716 | *DHFR* | rs1650676 | 5:79959398 | -0.53 | -4.59 | 8.24E-06 | 5.56E-03 |
| ENSG00000186340 | *THBS2* | rs117833626 | 6:169217159 | 1.96 | 4.59 | 8.26E-06 | 5.57E-03 |
| ENSG00000149328 | *GLB1L2* | rs4936229 | 11:134171497 | 0.45 | 4.59 | 8.28E-06 | 5.58E-03 |
| ENSG00000136108 | *CKAP2* | rs7981732 | 13:52912217 | 0.44 | 4.58 | 8.32E-06 | 5.61E-03 |
| ENSG00000153823 | *PID1* | rs17621000 | 2:230437933 | -0.89 | -4.58 | 8.35E-06 | 5.62E-03 |
| ENSG00000169071 | *ROR2* | rs12380829 | 9:94724877 | 0.50 | 4.58 | 8.44E-06 | 5.67E-03 |
| ENSG00000170369 | *CST2* | rs6132658 | 20:23758976 | -0.46 | -4.58 | 8.45E-06 | 5.68E-03 |
| ENSG00000149328 | *GLB1L2* | rs1455215275 | 11:134199508 | -0.49 | -4.58 | 8.51E-06 | 5.71E-03 |
| ENSG00000153823 | *PID1* | rs111209772 | 2:230428250 | -1.01 | -4.58 | 8.60E-06 | 5.77E-03 |
| ENSG00000169071 | *ROR2* | rs1919102 | 9:94736561 | 0.47 | 4.58 | 8.62E-06 | 5.78E-03 |
| ENSG00000136108 | *CKAP2* | rs7993748 | 13:52941631 | -0.43 | -4.57 | 8.70E-06 | 5.83E-03 |
| ENSG00000151470 | *C4orf33* | rs10028974 | 4:129871442 | 0.47 | 4.57 | 8.71E-06 | 5.83E-03 |
| ENSG00000169071 | *ROR2* | rs62564598 | 9:94611329 | 0.48 | 4.57 | 8.87E-06 | 5.92E-03 |
| ENSG00000169071 | *ROR2* | rs10992131 | 9:94611554 | 0.48 | 4.57 | 8.92E-06 | 5.95E-03 |
| ENSG00000228716 | *DHFR* | rs111692241 | 5:79895438 | -0.56 | -4.57 | 9.02E-06 | 6.01E-03 |
| ENSG00000170369 | *CST2* | rs6049106 | 20:23758700 | -0.46 | -4.57 | 9.02E-06 | 6.01E-03 |
| ENSG00000228716 | *DHFR* | rs2405875 | 5:79950708 | -0.55 | -4.56 | 9.12E-06 | 6.07E-03 |
| ENSG00000149328 | *GLB1L2* | rs1144214 | 11:134184141 | -0.45 | -4.56 | 9.14E-06 | 6.07E-03 |
| ENSG00000136108 | *CKAP2* | rs9596649 | 13:52934610 | 0.43 | 4.56 | 9.18E-06 | 6.10E-03 |
| ENSG00000169071 | *ROR2* | rs7048756 | 9:94612391 | 0.48 | 4.56 | 9.19E-06 | 6.11E-03 |
| ENSG00000153823 | *PID1* | rs17677491 | 2:230430854 | -0.90 | -4.56 | 9.21E-06 | 6.12E-03 |
| ENSG00000151470 | *C4orf33* | rs10857131 | 4:129735906 | -0.62 | -4.56 | 9.25E-06 | 6.14E-03 |
| ENSG00000169071 | *ROR2* | rs202038013 | 9:94502883 | -0.45 | -4.56 | 9.27E-06 | 6.15E-03 |
| ENSG00000136108 | *CKAP2* | rs7987115 | 13:52962097 | -0.45 | -4.56 | 9.28E-06 | 6.15E-03 |
| ENSG00000136108 | *CKAP2* | rs6561666 | 13:52930045 | 0.43 | 4.56 | 9.31E-06 | 6.17E-03 |
| ENSG00000153823 | *PID1* | rs72985999 | 2:230432470 | -0.91 | -4.56 | 9.32E-06 | 6.17E-03 |
| ENSG00000228716 | *DHFR* | rs1677695 | 5:79936276 | -0.54 | -4.56 | 9.36E-06 | 6.20E-03 |
| ENSG00000067798 | *NAV3* | rs78689967 | 12:77988826 | 0.54 | 4.55 | 9.54E-06 | 6.30E-03 |
| ENSG00000151470 | *C4orf33* | rs35408367 | 4:130052358 | -0.45 | -4.55 | 9.56E-06 | 6.31E-03 |
| ENSG00000136108 | *CKAP2* | rs4884452 | 13:52949784 | -0.45 | -4.55 | 9.61E-06 | 6.34E-03 |
| ENSG00000136108 | *CKAP2* | rs4885953 | 13:52976491 | -0.45 | -4.55 | 9.62E-06 | 6.34E-03 |
| ENSG00000228716 | *DHFR* | rs1643644 | 5:79948532 | -0.57 | -4.55 | 9.63E-06 | 6.35E-03 |
| ENSG00000251504 | *LINC01099* | rs1676191 | 4:178979527 | -0.49 | -4.55 | 9.67E-06 | 6.37E-03 |
| ENSG00000228716 | *DHFR* | rs701388 | 5:80136296 | 0.52 | 4.55 | 9.70E-06 | 6.39E-03 |
| ENSG00000136108 | *CKAP2* | rs9568728 | 13:52946593 | -0.45 | -4.55 | 9.74E-06 | 6.41E-03 |
| ENSG00000136108 | *CKAP2* | rs3866534 | 13:52786236 | -0.44 | -4.55 | 9.78E-06 | 6.44E-03 |
| ENSG00000251504 | *LINC01099* | rs1305043 | 4:178973065 | -0.43 | -4.55 | 9.79E-06 | 6.44E-03 |
| ENSG00000228716 | *DHFR* | rs6887400 | 5:79958700 | 0.44 | 4.55 | 9.82E-06 | 6.46E-03 |
| ENSG00000228716 | *DHFR* | rs7712332 | 5:79957835 | 0.44 | 4.55 | 9.82E-06 | 6.46E-03 |
| ENSG00000136108 | *CKAP2* | rs7400602 | 13:52916665 | 0.43 | 4.55 | 9.86E-06 | 6.48E-03 |
| ENSG00000136108 | *CKAP2* | rs9536066 | 13:52983981 | -0.43 | -4.54 | 9.97E-06 | 6.54E-03 |
| ENSG00000149328 | *GLB1L2* | rs4937881 | 11:134250818 | 0.44 | 4.54 | 1.00E-05 | 6.57E-03 |
| ENSG00000134278 | *SPIRE1* | rs1491449941 | 18:12659265 | 0.40 | 4.54 | 1.01E-05 | 6.59E-03 |
| ENSG00000136108 | *CKAP2* | rs7399631 | 13:52975580 | -0.45 | -4.54 | 1.01E-05 | 6.60E-03 |
| ENSG00000151470 | *C4orf33* | rs28711799 | 4:129874496 | 0.56 | 4.54 | 1.02E-05 | 6.68E-03 |
| ENSG00000151470 | *C4orf33* | rs337265 | 4:130061181 | -0.45 | -4.54 | 1.02E-05 | 6.68E-03 |
| ENSG00000169071 | *ROR2* | rs10992142 | 9:94625960 | 0.47 | 4.54 | 1.02E-05 | 6.70E-03 |
| ENSG00000151470 | *C4orf33* | rs234264 | 4:130056276 | -0.45 | -4.54 | 1.03E-05 | 6.71E-03 |
| ENSG00000151470 | *C4orf33* | rs560341 | 4:130057958 | -0.45 | -4.54 | 1.03E-05 | 6.71E-03 |
| ENSG00000151470 | *C4orf33* | rs6824219 | 4:129876836 | 0.56 | 4.54 | 1.03E-05 | 6.72E-03 |
| ENSG00000151470 | *C4orf33* | rs189236 | 4:130028412 | -0.47 | -4.53 | 1.04E-05 | 6.76E-03 |
| ENSG00000151470 | *C4orf33* | rs337279 | 4:130028426 | -0.47 | -4.53 | 1.04E-05 | 6.76E-03 |
| ENSG00000169071 | *ROR2* | rs72748313 | 9:94727100 | 0.52 | 4.53 | 1.05E-05 | 6.86E-03 |
| ENSG00000149328 | *GLB1L2* | rs7951640 | 11:134251252 | 0.44 | 4.53 | 1.07E-05 | 6.97E-03 |
| ENSG00000141404 | *GNAL* | rs28620345 | 18:11696651 | 0.61 | 4.53 | 1.07E-05 | 6.97E-03 |
| ENSG00000067798 | *NAV3* | rs11105884 | 12:78001009 | 0.54 | 4.53 | 1.07E-05 | 6.97E-03 |
| ENSG00000067798 | *NAV3* | rs11105883 | 12:78000924 | 0.54 | 4.53 | 1.07E-05 | 6.97E-03 |
| ENSG00000067798 | *NAV3* | rs11105881 | 12:78000729 | 0.54 | 4.53 | 1.07E-05 | 6.97E-03 |
| ENSG00000067798 | *NAV3* | rs11105880 | 12:78000384 | 0.54 | 4.53 | 1.07E-05 | 6.97E-03 |
| ENSG00000067798 | *NAV3* | rs11105879 | 12:77999772 | 0.54 | 4.53 | 1.07E-05 | 6.98E-03 |
| ENSG00000067798 | *NAV3* | rs12368030 | 12:77997280 | 0.54 | 4.52 | 1.08E-05 | 6.99E-03 |
| ENSG00000151470 | *C4orf33* | rs12512715 | 4:129772286 | -0.62 | -4.52 | 1.09E-05 | 7.06E-03 |
| ENSG00000151470 | *C4orf33* | rs4975274 | 4:129778076 | -0.62 | -4.52 | 1.11E-05 | 7.15E-03 |
| ENSG00000228716 | *DHFR* | rs1291022408 | 5:79902408 | -0.55 | -4.52 | 1.11E-05 | 7.15E-03 |
| ENSG00000151470 | *C4orf33* | rs1757929 | 4:130022200 | -0.47 | -4.52 | 1.11E-05 | 7.19E-03 |
| ENSG00000151470 | *C4orf33* | rs1757926 | 4:130021892 | -0.47 | -4.52 | 1.11E-05 | 7.19E-03 |
| ENSG00000151470 | *C4orf33* | rs1756011 | 4:130019663 | -0.47 | -4.52 | 1.12E-05 | 7.22E-03 |
| ENSG00000141404 | *GNAL* | rs8099486 | 18:11696329 | 0.67 | 4.51 | 1.13E-05 | 7.26E-03 |
| ENSG00000148344 | *PTGES* | rs913768 | 9:132044370 | 0.53 | 4.51 | 1.15E-05 | 7.38E-03 |
| ENSG00000136108 | *CKAP2* | rs61957304 | 13:52798423 | -0.44 | -4.51 | 1.16E-05 | 7.46E-03 |
| ENSG00000174080 | *CTSF* | rs2444846 | 11:66266706 | 0.49 | 4.50 | 1.18E-05 | 7.57E-03 |
| ENSG00000228716 | *DHFR* | rs3776967 | 5:80002350 | 0.44 | 4.50 | 1.18E-05 | 7.58E-03 |
| ENSG00000251504 | *LINC01099* | rs1430182530 | 4:178952215 | -0.47 | -4.50 | 1.19E-05 | 7.58E-03 |
| ENSG00000228716 | *DHFR* | rs11743909 | 5:80002425 | 0.44 | 4.50 | 1.19E-05 | 7.59E-03 |
| ENSG00000228716 | *DHFR* | rs7709909 | 5:80001170 | 0.44 | 4.50 | 1.19E-05 | 7.60E-03 |
| ENSG00000228716 | *DHFR* | rs1271705081 | 5:79912849 | -0.49 | -4.50 | 1.19E-05 | 7.63E-03 |
| ENSG00000228716 | *DHFR* | rs6151704 | 5:80004718 | 0.44 | 4.50 | 1.20E-05 | 7.68E-03 |
| ENSG00000151470 | *C4orf33* | rs11934740 | 4:129882748 | 0.55 | 4.50 | 1.20E-05 | 7.69E-03 |
| ENSG00000151470 | *C4orf33* | rs280600 | 4:129946283 | -0.47 | -4.50 | 1.21E-05 | 7.70E-03 |
| ENSG00000153823 | *PID1* | rs10490184 | 2:230440104 | -0.89 | -4.50 | 1.21E-05 | 7.70E-03 |
| ENSG00000170369 | *CST2* | rs11480527 | 20:23755579 | -0.47 | -4.50 | 1.21E-05 | 7.71E-03 |
| ENSG00000169071 | *ROR2* | rs7031562 | 9:94615986 | 0.48 | 4.50 | 1.21E-05 | 7.73E-03 |
| ENSG00000228716 | *DHFR* | rs245365 | 5:80139358 | 0.48 | 4.49 | 1.23E-05 | 7.83E-03 |
| ENSG00000228716 | *DHFR* | rs6151661 | 5:79973195 | 0.44 | 4.49 | 1.23E-05 | 7.84E-03 |
| ENSG00000149328 | *GLB1L2* | rs893952 | 11:134249349 | 0.44 | 4.49 | 1.23E-05 | 7.84E-03 |
| ENSG00000136108 | *CKAP2* | rs9526845 | 13:52781763 | -0.44 | -4.49 | 1.23E-05 | 7.85E-03 |
| ENSG00000141404 | *GNAL* | rs1392176326 | 18:11695912 | 0.55 | 4.49 | 1.25E-05 | 7.93E-03 |
| ENSG00000169071 | *ROR2* | rs7019210 | 9:94607341 | 0.48 | 4.49 | 1.25E-05 | 7.94E-03 |
| ENSG00000127328 | *RAB3IP* | rs3730607 | 12:69222809 | 1.56 | 4.48 | 1.29E-05 | 8.15E-03 |
| ENSG00000149328 | *GLB1L2* | rs893953 | 11:134249321 | 0.44 | 4.48 | 1.29E-05 | 8.15E-03 |
| ENSG00000228716 | *DHFR* | rs3776968 | 5:80006558 | 0.44 | 4.48 | 1.30E-05 | 8.22E-03 |
| ENSG00000149328 | *GLB1L2* | rs3133073 | 11:134199915 | -0.49 | -4.48 | 1.32E-05 | 8.30E-03 |
| ENSG00000151470 | *C4orf33* | rs280594 | 4:129942287 | -0.46 | -4.48 | 1.33E-05 | 8.35E-03 |
| ENSG00000228716 | *DHFR* | rs11950480 | 5:79957113 | -0.53 | -4.47 | 1.33E-05 | 8.37E-03 |
| ENSG00000228716 | *DHFR* | rs1650685 | 5:79957117 | -0.53 | -4.47 | 1.33E-05 | 8.37E-03 |
| ENSG00000134278 | *SPIRE1* | rs12954079 | 18:12663353 | 0.39 | 4.47 | 1.33E-05 | 8.39E-03 |
| ENSG00000151470 | *C4orf33* | rs5861878 | 4:130034935 | -0.51 | -4.47 | 1.34E-05 | 8.40E-03 |
| ENSG00000228716 | *DHFR* | rs1677640 | 5:79962864 | -0.52 | -4.47 | 1.35E-05 | 8.46E-03 |
| ENSG00000169071 | *ROR2* | rs2312734 | 9:94586256 | 0.47 | 4.47 | 1.37E-05 | 8.58E-03 |
| ENSG00000171522 | *PTGER4* | rs12655997 | 5:40291064 | 0.44 | 4.46 | 1.39E-05 | 8.67E-03 |
| ENSG00000179431 | *FJX1* | rs111430211 | 11:36397982 | 1.20 | 4.46 | 1.39E-05 | 8.69E-03 |
| ENSG00000151470 | *C4orf33* | rs318503 | 4:129807236 | 0.61 | 4.46 | 1.39E-05 | 8.69E-03 |
| ENSG00000151470 | *C4orf33* | rs7671508 | 4:129977667 | -0.55 | -4.46 | 1.40E-05 | 8.75E-03 |
| ENSG00000148344 | *PTGES* | rs1203223407 | 9:132037560 | 0.60 | 4.46 | 1.40E-05 | 8.75E-03 |
| ENSG00000151470 | *C4orf33* | rs1757938 | 4:129987759 | -0.46 | -4.46 | 1.41E-05 | 8.78E-03 |
| ENSG00000151470 | *C4orf33* | rs1444156959 | 4:129965671 | -0.55 | -4.46 | 1.41E-05 | 8.79E-03 |
| ENSG00000151470 | *C4orf33* | rs318501 | 4:129802948 | 0.61 | 4.46 | 1.41E-05 | 8.80E-03 |
| ENSG00000151470 | *C4orf33* | rs318500 | 4:129804727 | 0.61 | 4.46 | 1.42E-05 | 8.82E-03 |
| ENSG00000151470 | *C4orf33* | rs2162126 | 4:129779076 | -0.61 | -4.46 | 1.42E-05 | 8.82E-03 |
| ENSG00000151470 | *C4orf33* | rs318502 | 4:129801495 | 0.61 | 4.46 | 1.42E-05 | 8.83E-03 |
| ENSG00000151470 | *C4orf33* | rs1757937 | 4:129995171 | -0.46 | -4.46 | 1.42E-05 | 8.83E-03 |
| ENSG00000151470 | *C4orf33* | rs1757942 | 4:130001593 | -0.46 | -4.46 | 1.42E-05 | 8.83E-03 |
| ENSG00000151470 | *C4orf33* | rs1757936 | 4:130011891 | -0.55 | -4.46 | 1.42E-05 | 8.86E-03 |
| ENSG00000151470 | *C4orf33* | rs1838911 | 4:129991636 | -0.46 | -4.46 | 1.42E-05 | 8.87E-03 |
| ENSG00000151470 | *C4orf33* | rs2592948 | 4:129994690 | -0.46 | -4.46 | 1.42E-05 | 8.87E-03 |
| ENSG00000151470 | *C4orf33* | rs796886 | 4:129996889 | -0.46 | -4.46 | 1.42E-05 | 8.87E-03 |
| ENSG00000151470 | *C4orf33* | rs62316978 | 4:129992058 | -0.46 | -4.46 | 1.43E-05 | 8.87E-03 |
| ENSG00000151470 | *C4orf33* | rs2118045 | 4:129979793 | -0.46 | -4.46 | 1.43E-05 | 8.89E-03 |
| ENSG00000151470 | *C4orf33* | rs280597 | 4:129944209 | -0.46 | -4.46 | 1.43E-05 | 8.91E-03 |
| ENSG00000151470 | *C4orf33* | rs3099902 | 4:129963504 | -0.46 | -4.46 | 1.43E-05 | 8.91E-03 |
| ENSG00000151470 | *C4orf33* | rs62316971 | 4:129976373 | -0.46 | -4.46 | 1.44E-05 | 8.92E-03 |
| ENSG00000151470 | *C4orf33* | rs7654405 | 4:129977906 | -0.46 | -4.46 | 1.44E-05 | 8.93E-03 |
| ENSG00000151470 | *C4orf33* | rs2655311 | 4:129986299 | -0.46 | -4.46 | 1.44E-05 | 8.93E-03 |
| ENSG00000136108 | *CKAP2* | rs9535914 | 13:52791751 | -0.44 | -4.46 | 1.44E-05 | 8.94E-03 |
| ENSG00000151470 | *C4orf33* | rs280596 | 4:129942678 | -0.46 | -4.46 | 1.44E-05 | 8.95E-03 |
| ENSG00000151470 | *C4orf33* | rs3113488 | 4:129929138 | -0.46 | -4.46 | 1.44E-05 | 8.95E-03 |
| ENSG00000151470 | *C4orf33* | rs3113487 | 4:129924977 | -0.46 | -4.46 | 1.44E-05 | 8.95E-03 |
| ENSG00000228716 | *DHFR* | rs1677635 | 5:79962168 | -0.53 | -4.45 | 1.45E-05 | 9.00E-03 |
| ENSG00000228716 | *DHFR* | rs6151709 | 5:80012969 | 0.44 | 4.45 | 1.46E-05 | 9.03E-03 |
| ENSG00000141404 | *GNAL* | rs2035195 | 18:11693813 | 0.52 | 4.45 | 1.46E-05 | 9.04E-03 |
| ENSG00000169071 | *ROR2* | rs1997286 | 9:94585666 | 0.47 | 4.45 | 1.46E-05 | 9.07E-03 |
| ENSG00000151470 | *C4orf33* | rs2777822 | 4:130009131 | -0.46 | -4.45 | 1.47E-05 | 9.12E-03 |
| ENSG00000149328 | *GLB1L2* | rs1405802149 | 11:134166758 | 0.44 | 4.45 | 1.48E-05 | 9.14E-03 |
| ENSG00000151470 | *C4orf33* | rs11098994 | 4:129888651 | 0.46 | 4.45 | 1.48E-05 | 9.15E-03 |
| ENSG00000228716 | *DHFR* | rs32952 | 5:80021445 | 0.44 | 4.45 | 1.50E-05 | 9.26E-03 |
| ENSG00000171522 | *PTGER4* | rs2100001 | 5:40355376 | 0.42 | 4.45 | 1.50E-05 | 9.27E-03 |
| ENSG00000228716 | *DHFR* | rs28027 | 5:80052803 | 0.52 | 4.44 | 1.51E-05 | 9.32E-03 |
| ENSG00000136108 | *CKAP2* | rs9535947 | 13:52839225 | -0.47 | -4.44 | 1.53E-05 | 9.43E-03 |
| ENSG00000151470 | *C4orf33* | rs2597837 | 4:129904050 | -0.46 | -4.44 | 1.54E-05 | 9.44E-03 |
| ENSG00000149328 | *GLB1L2* | rs3741099 | 11:134240524 | 0.51 | 4.44 | 1.54E-05 | 9.45E-03 |
| ENSG00000228716 | *DHFR* | rs73765838 | 5:79947822 | -0.56 | -4.44 | 1.54E-05 | 9.46E-03 |
| ENSG00000228716 | *DHFR* | rs1677708 | 5:79958039 | -0.52 | -4.44 | 1.54E-05 | 9.46E-03 |
| ENSG00000151470 | *C4orf33* | rs1376202 | 4:129907917 | -0.46 | -4.44 | 1.55E-05 | 9.51E-03 |
| ENSG00000169071 | *ROR2* | rs12551140 | 9:94589135 | 0.46 | 4.44 | 1.55E-05 | 9.53E-03 |
| ENSG00000171522 | *PTGER4* | rs13186880 | 5:40429429 | 0.54 | 4.44 | 1.56E-05 | 9.55E-03 |
| ENSG00000136108 | *CKAP2* | rs9536006 | 13:52887707 | -0.44 | -4.44 | 1.56E-05 | 9.57E-03 |
| ENSG00000228716 | *DHFR* | rs1650683 | 5:79957147 | -0.52 | -4.44 | 1.56E-05 | 9.59E-03 |
| ENSG00000228716 | *DHFR* | rs1650675 | 5:79959524 | -0.52 | -4.44 | 1.57E-05 | 9.59E-03 |
| ENSG00000151470 | *C4orf33* | rs280601 | 4:129914268 | -0.46 | -4.44 | 1.57E-05 | 9.62E-03 |
| ENSG00000228716 | *DHFR* | rs26282 | 5:80050981 | 0.52 | 4.43 | 1.58E-05 | 9.69E-03 |
| ENSG00000228716 | *DHFR* | rs1643651 | 5:79955924 | -0.52 | -4.43 | 1.59E-05 | 9.71E-03 |
| ENSG00000228716 | *DHFR* | rs56357417 | 5:79958336 | -0.52 | -4.43 | 1.59E-05 | 9.71E-03 |
| ENSG00000228716 | *DHFR* | rs1677647 | 5:79964816 | -0.52 | -4.43 | 1.59E-05 | 9.72E-03 |
| ENSG00000228716 | *DHFR* | rs1677625 | 5:79960448 | -0.52 | -4.43 | 1.59E-05 | 9.73E-03 |
| ENSG00000228716 | *DHFR* | rs1677690 | 5:79957099 | -0.52 | -4.43 | 1.59E-05 | 9.73E-03 |
| ENSG00000228716 | *DHFR* | rs1650666 | 5:79962439 | -0.52 | -4.43 | 1.59E-05 | 9.73E-03 |
| ENSG00000228716 | *DHFR* | rs1650674 | 5:79959683 | -0.52 | -4.43 | 1.59E-05 | 9.73E-03 |
| ENSG00000228716 | *DHFR* | rs1650671 | 5:79960268 | -0.52 | -4.43 | 1.59E-05 | 9.73E-03 |
| ENSG00000228716 | *DHFR* | rs375102283 | 5:79960301 | -0.52 | -4.43 | 1.59E-05 | 9.73E-03 |
| ENSG00000228716 | *DHFR* | rs1650670 | 5:79960849 | -0.52 | -4.43 | 1.59E-05 | 9.73E-03 |
| ENSG00000228716 | *DHFR* | rs1382543 | 5:79960955 | -0.52 | -4.43 | 1.59E-05 | 9.73E-03 |
| ENSG00000228716 | *DHFR* | rs1824837 | 5:79961241 | -0.52 | -4.43 | 1.59E-05 | 9.73E-03 |
| ENSG00000228716 | *DHFR* | rs1824838 | 5:79961312 | -0.52 | -4.43 | 1.59E-05 | 9.73E-03 |
| ENSG00000228716 | *DHFR* | rs6151614 | 5:79961335 | -0.52 | -4.43 | 1.59E-05 | 9.73E-03 |
| ENSG00000228716 | *DHFR* | rs1824839 | 5:79961362 | -0.52 | -4.43 | 1.59E-05 | 9.73E-03 |
| ENSG00000228716 | *DHFR* | rs6151618 | 5:79961673 | -0.52 | -4.43 | 1.59E-05 | 9.73E-03 |
| ENSG00000228716 | *DHFR* | rs6151619 | 5:79961675 | -0.52 | -4.43 | 1.59E-05 | 9.73E-03 |
| ENSG00000228716 | *DHFR* | rs1677629 | 5:79961841 | -0.52 | -4.43 | 1.59E-05 | 9.73E-03 |
| ENSG00000228716 | *DHFR* | rs1650667 | 5:79962226 | -0.52 | -4.43 | 1.59E-05 | 9.73E-03 |
| ENSG00000228716 | *DHFR* | rs1677638 | 5:79962545 | -0.52 | -4.43 | 1.59E-05 | 9.73E-03 |
| ENSG00000228716 | *DHFR* | rs1677639 | 5:79962548 | -0.52 | -4.43 | 1.59E-05 | 9.73E-03 |
| ENSG00000228716 | *DHFR* | rs1650665 | 5:79962578 | -0.52 | -4.43 | 1.59E-05 | 9.73E-03 |
| ENSG00000228716 | *DHFR* | rs2897262 | 5:79962676 | -0.52 | -4.43 | 1.59E-05 | 9.73E-03 |
| ENSG00000228716 | *DHFR* | rs1677703 | 5:79957737 | -0.52 | -4.43 | 1.59E-05 | 9.73E-03 |
| ENSG00000228716 | *DHFR* | rs1650682 | 5:79957931 | -0.52 | -4.43 | 1.59E-05 | 9.73E-03 |
| ENSG00000228716 | *DHFR* | rs1650681 | 5:79958258 | -0.52 | -4.43 | 1.59E-05 | 9.73E-03 |
| ENSG00000228716 | *DHFR* | rs1650679 | 5:79958434 | -0.52 | -4.43 | 1.59E-05 | 9.73E-03 |
| ENSG00000228716 | *DHFR* | rs1650678 | 5:79958711 | -0.52 | -4.43 | 1.59E-05 | 9.73E-03 |
| ENSG00000228716 | *DHFR* | rs1677623 | 5:79959103 | -0.52 | -4.43 | 1.59E-05 | 9.73E-03 |
| ENSG00000228716 | *DHFR* | rs1650677 | 5:79959253 | -0.52 | -4.43 | 1.59E-05 | 9.73E-03 |
| ENSG00000228716 | *DHFR* | rs1650668 | 5:79961955 | -0.52 | -4.43 | 1.59E-05 | 9.73E-03 |
| ENSG00000228716 | *DHFR* | rs1677641 | 5:79962919 | -0.52 | -4.43 | 1.59E-05 | 9.73E-03 |
| ENSG00000228716 | *DHFR* | rs1650663 | 5:79963197 | -0.52 | -4.43 | 1.59E-05 | 9.73E-03 |
| ENSG00000228716 | *DHFR* | rs1650662 | 5:79963287 | -0.52 | -4.43 | 1.59E-05 | 9.73E-03 |
| ENSG00000228716 | *DHFR* | rs1677642 | 5:79963318 | -0.52 | -4.43 | 1.59E-05 | 9.73E-03 |
| ENSG00000228716 | *DHFR* | rs1650661 | 5:79963363 | -0.52 | -4.43 | 1.59E-05 | 9.73E-03 |
| ENSG00000228716 | *DHFR* | rs140784251 | 5:79957390 | -0.52 | -4.43 | 1.59E-05 | 9.73E-03 |
| ENSG00000228716 | *DHFR* | rs1650658 | 5:79963651 | -0.52 | -4.43 | 1.59E-05 | 9.73E-03 |
| ENSG00000228716 | *DHFR* | rs1677698 | 5:79957483 | -0.52 | -4.43 | 1.59E-05 | 9.73E-03 |
| ENSG00000228716 | *DHFR* | rs1650664 | 5:79963023 | -0.52 | -4.43 | 1.59E-05 | 9.73E-03 |
| ENSG00000228716 | *DHFR* | rs1650659 | 5:79963537 | -0.52 | -4.43 | 1.59E-05 | 9.73E-03 |
| ENSG00000228716 | *DHFR* | rs1677643 | 5:79963940 | -0.52 | -4.43 | 1.59E-05 | 9.73E-03 |
| ENSG00000228716 | *DHFR* | rs1677644 | 5:79963976 | -0.52 | -4.43 | 1.59E-05 | 9.73E-03 |
| ENSG00000228716 | *DHFR* | rs1677682 | 5:79956593 | -0.52 | -4.43 | 1.59E-05 | 9.73E-03 |
| ENSG00000228716 | *DHFR* | rs1650657 | 5:79964249 | -0.52 | -4.43 | 1.59E-05 | 9.73E-03 |
| ENSG00000228716 | *DHFR* | rs1677645 | 5:79964419 | -0.52 | -4.43 | 1.59E-05 | 9.73E-03 |
| ENSG00000228716 | *DHFR* | rs1650654 | 5:79965020 | -0.52 | -4.43 | 1.59E-05 | 9.73E-03 |
| ENSG00000228716 | *DHFR* | rs1677648 | 5:79965091 | -0.52 | -4.43 | 1.59E-05 | 9.73E-03 |
| ENSG00000228716 | *DHFR* | rs1650656 | 5:79964800 | -0.52 | -4.43 | 1.59E-05 | 9.73E-03 |
| ENSG00000228716 | *DHFR* | rs1650653 | 5:79965257 | -0.52 | -4.43 | 1.59E-05 | 9.73E-03 |
| ENSG00000228716 | *DHFR* | rs1215805683 | 5:79958224 | -0.52 | -4.43 | 1.59E-05 | 9.73E-03 |
| ENSG00000228716 | *DHFR* | rs1650655 | 5:79964808 | -0.52 | -4.43 | 1.59E-05 | 9.73E-03 |
| ENSG00000228716 | *DHFR* | rs1650680 | 5:79958366 | -0.52 | -4.43 | 1.59E-05 | 9.74E-03 |
| ENSG00000228716 | *DHFR* | rs1631572 | 5:79958595 | -0.52 | -4.43 | 1.60E-05 | 9.75E-03 |
| ENSG00000228716 | *DHFR* | rs1650652 | 5:79965365 | -0.52 | -4.43 | 1.60E-05 | 9.76E-03 |
| ENSG00000228716 | *DHFR* | rs1650650 | 5:79965775 | -0.52 | -4.43 | 1.60E-05 | 9.76E-03 |
| ENSG00000228716 | *DHFR* | rs1677649 | 5:79966197 | -0.52 | -4.43 | 1.60E-05 | 9.76E-03 |
| ENSG00000228716 | *DHFR* | rs245009 | 5:80032002 | 0.49 | 4.43 | 1.61E-05 | 9.81E-03 |
| ENSG00000148344 | *PTGES* | rs913774 | 9:132033654 | -0.54 | -4.43 | 1.61E-05 | 9.81E-03 |
| ENSG00000004776 | *HSPB6* | rs75120684 | 19:36288595 | 1.13 | 4.43 | 1.62E-05 | 9.89E-03 |
| ENSG00000228716 | *DHFR* | rs245335 | 5:80029408 | 0.44 | 4.43 | 1.63E-05 | 9.92E-03 |
| ENSG00000136108 | *CKAP2* | rs9536278 | 13:53335943 | -0.42 | -4.43 | 1.63E-05 | 9.94E-03 |
| ENSG00000171522 | *PTGER4* | rs13165432 | 5:40441645 | 0.54 | 4.42 | 1.64E-05 | 9.99E-03 |
| ENSG00000169071 | *ROR2* | rs6479405 | 9:94874310 | 0.44 | 4.42 | 1.64E-05 | 1.00E-02 |
| ENSG00000170369 | *CST2* | rs11697006 | 20:23757966 | -0.46 | -4.42 | 1.64E-05 | 1.00E-02 |
| ENSG00000228716 | *DHFR* | rs6151632 | 5:79966679 | -0.52 | -4.42 | 1.65E-05 | 1.01E-02 |
| ENSG00000148344 | *PTGES* | rs867252 | 9:132033415 | -0.54 | -4.42 | 1.68E-05 | 1.02E-02 |
| ENSG00000169071 | *ROR2* | rs12376231 | 9:94593925 | 0.47 | 4.42 | 1.69E-05 | 1.02E-02 |
| ENSG00000151470 | *C4orf33* | rs546956 | 4:130046216 | -0.55 | -4.42 | 1.69E-05 | 1.02E-02 |
| ENSG00000004776 | *HSPB6* | rs60128322 | 19:36259810 | 1.06 | 4.42 | 1.69E-05 | 1.02E-02 |
| ENSG00000228716 | *DHFR* | rs397764797 | 5:79959368 | -0.52 | -4.42 | 1.69E-05 | 1.03E-02 |
| ENSG00000228716 | *DHFR* | rs73765839 | 5:79947823 | -0.55 | -4.42 | 1.70E-05 | 1.03E-02 |
| ENSG00000169071 | *ROR2* | rs10820911 | 9:94646797 | 0.43 | 4.42 | 1.70E-05 | 1.03E-02 |
| ENSG00000151470 | *C4orf33* | rs10032000 | 4:129863157 | 0.60 | 4.42 | 1.70E-05 | 1.03E-02 |
| ENSG00000141404 | *GNAL* | rs16976596 | 18:11696613 | 0.60 | 4.42 | 1.71E-05 | 1.03E-02 |
| ENSG00000171522 | *PTGER4* | rs1992661 | 5:40414989 | 0.47 | 4.41 | 1.73E-05 | 1.04E-02 |
| ENSG00000169071 | *ROR2* | rs10992071 | 9:94494471 | 0.52 | 4.41 | 1.74E-05 | 1.05E-02 |
| ENSG00000228716 | *DHFR* | rs1677650 | 5:79967164 | -0.52 | -4.41 | 1.76E-05 | 1.06E-02 |
| ENSG00000151470 | *C4orf33* | rs148945170 | 4:129827850 | 0.60 | 4.41 | 1.76E-05 | 1.06E-02 |
| ENSG00000171522 | *PTGER4* | rs13160782 | 5:40428061 | 0.53 | 4.41 | 1.76E-05 | 1.06E-02 |
| ENSG00000113657 | *DPYSL3* | rs11749030 | 5:146652392 | -0.64 | -4.41 | 1.76E-05 | 1.06E-02 |
| ENSG00000228716 | *DHFR* | rs1677628 | 5:79961781 | -0.52 | -4.41 | 1.76E-05 | 1.06E-02 |
| ENSG00000151470 | *C4orf33* | rs3099903 | 4:129962072 | -0.46 | -4.41 | 1.77E-05 | 1.06E-02 |
| ENSG00000134278 | *SPIRE1* | rs150778690 | 18:12547096 | 0.90 | 4.41 | 1.77E-05 | 1.07E-02 |
| ENSG00000134278 | *SPIRE1* | rs77991961 | 18:12551289 | 0.90 | 4.41 | 1.78E-05 | 1.07E-02 |
| ENSG00000151470 | *C4orf33* | rs4975267 | 4:129744992 | -0.60 | -4.40 | 1.78E-05 | 1.07E-02 |
| ENSG00000171522 | *PTGER4* | rs13186205 | 5:40289988 | 0.43 | 4.40 | 1.80E-05 | 1.08E-02 |
| ENSG00000136108 | *CKAP2* | rs235775 | 13:53336663 | 0.44 | 4.40 | 1.80E-05 | 1.08E-02 |
| ENSG00000151470 | *C4orf33* | rs13120702 | 4:129755980 | -0.60 | -4.40 | 1.81E-05 | 1.08E-02 |
| ENSG00000228716 | *DHFR* | rs1628627 | 5:79967262 | -0.52 | -4.40 | 1.81E-05 | 1.09E-02 |
| ENSG00000141404 | *GNAL* | rs9675415 | 18:11690019 | 0.53 | 4.40 | 1.81E-05 | 1.09E-02 |
| ENSG00000136108 | *CKAP2* | rs9596651 | 13:52938582 | 0.45 | 4.40 | 1.83E-05 | 1.10E-02 |
| ENSG00000228716 | *DHFR* | rs10563790 | 5:80004608 | -0.53 | -4.40 | 1.84E-05 | 1.10E-02 |
| ENSG00000251504 | *LINC01099* | rs140998986 | 4:178986710 | -0.51 | -4.40 | 1.84E-05 | 1.10E-02 |
| ENSG00000151470 | *C4orf33* | rs6812361 | 4:129899758 | 0.54 | 4.39 | 1.88E-05 | 1.12E-02 |
| ENSG00000113657 | *DPYSL3* | rs116143441 | 5:146663884 | -0.65 | -4.39 | 1.89E-05 | 1.12E-02 |
| ENSG00000228716 | *DHFR* | rs9686214 | 5:80186135 | -0.48 | -4.39 | 1.89E-05 | 1.13E-02 |
| ENSG00000151470 | *C4orf33* | rs202215815 | 4:130046217 | -0.55 | -4.39 | 1.90E-05 | 1.13E-02 |
| ENSG00000169071 | *ROR2* | rs10820906 | 9:94587274 | 0.47 | 4.39 | 1.91E-05 | 1.14E-02 |
| ENSG00000127325 | *BEST3* | rs12423593 | 12:70084049 | 0.45 | 4.39 | 1.91E-05 | 1.14E-02 |
| ENSG00000228716 | *DHFR* | rs245342 | 5:80159414 | 0.45 | 4.39 | 1.93E-05 | 1.14E-02 |
| ENSG00000153823 | *PID1* | rs17677612 | 2:230453927 | -0.87 | -4.38 | 1.96E-05 | 1.16E-02 |
| ENSG00000134278 | *SPIRE1* | rs11080581 | 18:12554672 | 0.44 | 4.38 | 1.98E-05 | 1.17E-02 |
| ENSG00000169071 | *ROR2* | rs12376539 | 9:94590770 | 0.46 | 4.38 | 1.98E-05 | 1.17E-02 |
| ENSG00000169071 | *ROR2* | rs7045333 | 9:94591789 | 0.46 | 4.38 | 1.98E-05 | 1.17E-02 |
| ENSG00000169071 | *ROR2* | rs4743857 | 9:94595578 | 0.46 | 4.38 | 1.98E-05 | 1.17E-02 |
| ENSG00000169071 | *ROR2* | rs72746206 | 9:94595494 | 0.46 | 4.38 | 1.98E-05 | 1.17E-02 |
| ENSG00000169071 | *ROR2* | rs4322068 | 9:94593679 | 0.46 | 4.38 | 1.98E-05 | 1.17E-02 |
| ENSG00000169071 | *ROR2* | rs6479374 | 9:94594731 | 0.46 | 4.38 | 1.98E-05 | 1.17E-02 |
| ENSG00000169071 | *ROR2* | rs7856144 | 9:94594505 | 0.46 | 4.38 | 1.98E-05 | 1.17E-02 |
| ENSG00000169071 | *ROR2* | rs7874148 | 9:94594375 | 0.46 | 4.38 | 1.98E-05 | 1.17E-02 |
| ENSG00000136108 | *CKAP2* | rs9527001 | 13:53334992 | -0.42 | -4.38 | 1.99E-05 | 1.18E-02 |
| ENSG00000169071 | *ROR2* | rs2312732 | 9:94582969 | 0.47 | 4.38 | 1.99E-05 | 1.18E-02 |
| ENSG00000113657 | *DPYSL3* | rs78417445 | 5:146659014 | -0.64 | -4.38 | 1.99E-05 | 1.18E-02 |
| ENSG00000134278 | *SPIRE1* | rs12458000 | 18:12561763 | 0.44 | 4.38 | 2.00E-05 | 1.18E-02 |
| ENSG00000136108 | *CKAP2* | rs9527006 | 13:53336212 | -0.42 | -4.38 | 2.02E-05 | 1.19E-02 |
| ENSG00000112874 | *NUDT12* | rs12654426 | 5:103368387 | 0.47 | 4.37 | 2.03E-05 | 1.19E-02 |
| ENSG00000141404 | *GNAL* | rs8099216 | 18:11696328 | 0.63 | 4.37 | 2.03E-05 | 1.19E-02 |
| ENSG00000228716 | *DHFR* | rs1677676 | 5:79954098 | -0.56 | -4.37 | 2.03E-05 | 1.20E-02 |
| ENSG00000228716 | *DHFR* | rs6871067 | 5:80220759 | 0.43 | 4.37 | 2.04E-05 | 1.20E-02 |
| ENSG00000228716 | *DHFR* | rs6151615 | 5:79961432 | -0.51 | -4.37 | 2.04E-05 | 1.20E-02 |
| ENSG00000228716 | *DHFR* | rs1650737 | 5:80001785 | -0.51 | -4.37 | 2.04E-05 | 1.20E-02 |
| ENSG00000228716 | *DHFR* | rs844369 | 5:79993502 | -0.51 | -4.37 | 2.05E-05 | 1.21E-02 |
| ENSG00000228716 | *DHFR* | rs836816 | 5:79994622 | -0.51 | -4.37 | 2.05E-05 | 1.21E-02 |
| ENSG00000228716 | *DHFR* | rs2560425 | 5:79991301 | -0.51 | -4.37 | 2.05E-05 | 1.21E-02 |
| ENSG00000228716 | *DHFR* | rs836795 | 5:80012071 | -0.51 | -4.37 | 2.05E-05 | 1.21E-02 |
| ENSG00000228716 | *DHFR* | rs245332 | 5:80026268 | -0.51 | -4.37 | 2.05E-05 | 1.21E-02 |
| ENSG00000228716 | *DHFR* | rs26267 | 5:80040674 | 0.51 | 4.37 | 2.05E-05 | 1.21E-02 |
| ENSG00000228716 | *DHFR* | rs32959 | 5:80042055 | 0.51 | 4.37 | 2.05E-05 | 1.21E-02 |
| ENSG00000228716 | *DHFR* | rs10044730 | 5:80014944 | 0.44 | 4.37 | 2.06E-05 | 1.21E-02 |
| ENSG00000169071 | *ROR2* | rs7029950 | 9:94607333 | 0.46 | 4.37 | 2.07E-05 | 1.22E-02 |
| ENSG00000140876 | *NUDT7* | rs3743756 | 16:77759136 | 0.64 | 4.37 | 2.07E-05 | 1.22E-02 |
| ENSG00000151470 | *C4orf33* | rs318517 | 4:129817440 | 0.59 | 4.37 | 2.08E-05 | 1.22E-02 |
| ENSG00000151470 | *C4orf33* | rs318521 | 4:129818192 | 0.59 | 4.37 | 2.08E-05 | 1.22E-02 |
| ENSG00000151470 | *C4orf33* | rs10010659 | 4:129856847 | 0.59 | 4.37 | 2.08E-05 | 1.22E-02 |
| ENSG00000151470 | *C4orf33* | rs318536 | 4:129831257 | 0.59 | 4.37 | 2.08E-05 | 1.22E-02 |
| ENSG00000169071 | *ROR2* | rs62564595 | 9:94608540 | 0.46 | 4.37 | 2.08E-05 | 1.22E-02 |
| ENSG00000169071 | *ROR2* | rs6479375 | 9:94608587 | 0.46 | 4.37 | 2.08E-05 | 1.22E-02 |
| ENSG00000169071 | *ROR2* | rs7859331 | 9:94608843 | 0.46 | 4.37 | 2.09E-05 | 1.22E-02 |
| ENSG00000151470 | *C4orf33* | rs318543 | 4:129836255 | 0.59 | 4.37 | 2.09E-05 | 1.22E-02 |
| ENSG00000228716 | *DHFR* | rs245331 | 5:80026377 | -0.51 | -4.37 | 2.09E-05 | 1.22E-02 |
| ENSG00000151470 | *C4orf33* | rs318552 | 4:129841122 | 0.59 | 4.37 | 2.09E-05 | 1.23E-02 |
| ENSG00000228716 | *DHFR* | rs35021383 | 5:80008501 | -0.51 | -4.37 | 2.10E-05 | 1.23E-02 |
| ENSG00000004776 | *HSPB6* | rs148417617 | 19:36276543 | 1.06 | 4.37 | 2.10E-05 | 1.23E-02 |
| ENSG00000004776 | *HSPB6* | rs79775485 | 19:36269652 | 1.06 | 4.37 | 2.10E-05 | 1.23E-02 |
| ENSG00000141404 | *GNAL* | rs8099179 | 18:11696168 | 0.51 | 4.36 | 2.12E-05 | 1.24E-02 |
| ENSG00000141404 | *GNAL* | rs8099016 | 18:11696064 | 0.51 | 4.36 | 2.12E-05 | 1.24E-02 |
| ENSG00000141404 | *GNAL* | rs8096345 | 18:11695992 | 0.51 | 4.36 | 2.14E-05 | 1.25E-02 |
| ENSG00000169071 | *ROR2* | rs397819172 | 9:94704214 | 0.45 | 4.36 | 2.14E-05 | 1.25E-02 |
| ENSG00000228716 | *DHFR* | rs34965641 | 5:79942376 | -0.53 | -4.36 | 2.15E-05 | 1.25E-02 |
| ENSG00000151470 | *C4orf33* | rs3113407 | 4:129827145 | 0.59 | 4.36 | 2.16E-05 | 1.26E-02 |
| ENSG00000151470 | *C4orf33* | rs1668289 | 4:129827039 | 0.59 | 4.36 | 2.16E-05 | 1.26E-02 |
| ENSG00000141404 | *GNAL* | rs8099342 | 18:11696268 | 0.51 | 4.36 | 2.18E-05 | 1.27E-02 |
| ENSG00000113657 | *DPYSL3* | rs11950257 | 5:146662190 | -0.64 | -4.36 | 2.19E-05 | 1.27E-02 |
| ENSG00000170369 | *CST2* | rs6132657 | 20:23758190 | -0.45 | -4.36 | 2.19E-05 | 1.28E-02 |
| ENSG00000228716 | *DHFR* | rs1650688 | 5:79956129 | -0.51 | -4.35 | 2.21E-05 | 1.28E-02 |
| ENSG00000113657 | *DPYSL3* | rs80248170 | 5:146667352 | -0.64 | -4.35 | 2.21E-05 | 1.29E-02 |
| ENSG00000171522 | *PTGER4* | rs6864103 | 5:40302452 | 0.41 | 4.35 | 2.24E-05 | 1.30E-02 |
| ENSG00000141404 | *GNAL* | rs1863122 | 18:11694882 | 0.51 | 4.35 | 2.24E-05 | 1.30E-02 |
| ENSG00000151470 | *C4orf33* | rs4975289 | 4:129873281 | 0.45 | 4.35 | 2.25E-05 | 1.31E-02 |
| ENSG00000141404 | *GNAL* | rs113260127 | 18:11693072 | 0.51 | 4.35 | 2.26E-05 | 1.31E-02 |
| ENSG00000141404 | *GNAL* | rs1028279098 | 18:11693065 | 0.51 | 4.35 | 2.26E-05 | 1.31E-02 |
| ENSG00000136108 | *CKAP2* | rs55966473 | 13:52834850 | -0.52 | -4.35 | 2.26E-05 | 1.31E-02 |
| ENSG00000141404 | *GNAL* | rs8090096 | 18:11694008 | 0.51 | 4.35 | 2.27E-05 | 1.32E-02 |
| ENSG00000141404 | *GNAL* | rs8085488 | 18:11693956 | 0.51 | 4.35 | 2.28E-05 | 1.32E-02 |
| ENSG00000067798 | *NAV3* | rs201096535 | 12:77973998 | 0.52 | 4.35 | 2.28E-05 | 1.32E-02 |
| ENSG00000171522 | *PTGER4* | rs10601166 | 5:40302251 | 0.42 | 4.35 | 2.28E-05 | 1.32E-02 |
| ENSG00000171522 | *PTGER4* | rs17226632 | 5:40311005 | 0.42 | 4.35 | 2.29E-05 | 1.32E-02 |
| ENSG00000171522 | *PTGER4* | rs10462010 | 5:40311568 | 0.42 | 4.35 | 2.29E-05 | 1.32E-02 |
| ENSG00000171522 | *PTGER4* | rs4957127 | 5:40316010 | 0.42 | 4.34 | 2.29E-05 | 1.33E-02 |
| ENSG00000141404 | *GNAL* | rs61670374 | 18:11693363 | 0.51 | 4.34 | 2.30E-05 | 1.33E-02 |
| ENSG00000141404 | *GNAL* | rs57892223 | 18:11693194 | 0.51 | 4.34 | 2.31E-05 | 1.33E-02 |
| ENSG00000141404 | *GNAL* | rs2035194 | 18:11693808 | 0.51 | 4.34 | 2.32E-05 | 1.34E-02 |
| ENSG00000184160 | *ADRA2C* | rs62287909 | 4:4370126 | -1.56 | -4.34 | 2.32E-05 | 1.34E-02 |
| ENSG00000112874 | *NUDT12* | rs11242515 | 5:103331818 | 0.46 | 4.34 | 2.34E-05 | 1.35E-02 |
| ENSG00000141404 | *GNAL* | rs375511994 | 18:11692817 | 0.51 | 4.34 | 2.34E-05 | 1.35E-02 |
| ENSG00000141404 | *GNAL* | rs8098596 | 18:11692773 | 0.51 | 4.34 | 2.34E-05 | 1.35E-02 |
| ENSG00000251504 | *LINC01099* | rs12649872 | 4:178919164 | 0.47 | 4.34 | 2.34E-05 | 1.35E-02 |
| ENSG00000251504 | *LINC01099* | rs12641431 | 4:178918074 | 0.47 | 4.34 | 2.35E-05 | 1.35E-02 |
| ENSG00000171522 | *PTGER4* | rs6859310 | 5:40301922 | 0.41 | 4.34 | 2.35E-05 | 1.35E-02 |
| ENSG00000136108 | *CKAP2* | rs4545703 | 13:52802822 | -0.43 | -4.34 | 2.37E-05 | 1.36E-02 |
| ENSG00000169071 | *ROR2* | rs1534534 | 9:94701825 | 0.45 | 4.34 | 2.38E-05 | 1.37E-02 |
| ENSG00000148344 | *PTGES* | rs3824537 | 9:132045333 | 0.52 | 4.34 | 2.38E-05 | 1.37E-02 |
| ENSG00000136108 | *CKAP2* | rs3886077 | 13:52949053 | -0.42 | -4.33 | 2.42E-05 | 1.39E-02 |
| ENSG00000228716 | *DHFR* | rs1650701 | 5:79944431 | -0.55 | -4.33 | 2.42E-05 | 1.39E-02 |
| ENSG00000228716 | *DHFR* | rs1650700 | 5:79944432 | -0.55 | -4.33 | 2.42E-05 | 1.39E-02 |
| ENSG00000148344 | *PTGES* | rs17456945 | 9:132046625 | 0.52 | 4.33 | 2.43E-05 | 1.39E-02 |
| ENSG00000228716 | *DHFR* | rs1346464 | 5:79967618 | -0.51 | -4.33 | 2.44E-05 | 1.40E-02 |
| ENSG00000148344 | *PTGES* | rs1008440438 | 9:132046043 | 0.52 | 4.33 | 2.44E-05 | 1.40E-02 |
| ENSG00000228716 | *DHFR* | rs1346463 | 5:79967652 | -0.51 | -4.33 | 2.45E-05 | 1.40E-02 |
| ENSG00000228716 | *DHFR* | rs1677652 | 5:79967954 | -0.51 | -4.33 | 2.45E-05 | 1.40E-02 |
| ENSG00000228716 | *DHFR* | rs1650648 | 5:79968258 | -0.51 | -4.33 | 2.45E-05 | 1.40E-02 |
| ENSG00000228716 | *DHFR* | rs1677654 | 5:79968330 | -0.51 | -4.33 | 2.45E-05 | 1.40E-02 |
| ENSG00000228716 | *DHFR* | rs1677655 | 5:79968496 | -0.51 | -4.33 | 2.45E-05 | 1.40E-02 |
| ENSG00000228716 | *DHFR* | rs1677653 | 5:79968271 | -0.51 | -4.33 | 2.46E-05 | 1.40E-02 |
| ENSG00000228716 | *DHFR* | rs836804 | 5:79976446 | -0.51 | -4.33 | 2.46E-05 | 1.40E-02 |
| ENSG00000228716 | *DHFR* | rs836813 | 5:79971513 | -0.51 | -4.33 | 2.46E-05 | 1.41E-02 |
| ENSG00000141404 | *GNAL* | rs8087266 | 18:11688979 | 0.50 | 4.33 | 2.46E-05 | 1.41E-02 |
| ENSG00000228716 | *DHFR* | rs836810 | 5:79972212 | -0.51 | -4.33 | 2.46E-05 | 1.41E-02 |
| ENSG00000228716 | *DHFR* | rs836809 | 5:79972760 | -0.51 | -4.33 | 2.46E-05 | 1.41E-02 |
| ENSG00000228716 | *DHFR* | rs836808 | 5:79974570 | -0.51 | -4.33 | 2.47E-05 | 1.41E-02 |
| ENSG00000067798 | *NAV3* | rs711108 | 12:77989880 | 0.49 | 4.33 | 2.47E-05 | 1.41E-02 |
| ENSG00000228716 | *DHFR* | rs836806 | 5:79974983 | -0.51 | -4.33 | 2.48E-05 | 1.41E-02 |
| ENSG00000171522 | *PTGER4* | rs4957126 | 5:40298449 | 0.42 | 4.33 | 2.48E-05 | 1.42E-02 |
| ENSG00000136108 | *CKAP2* | rs9526913 | 13:52975837 | -0.42 | -4.32 | 2.49E-05 | 1.42E-02 |
| ENSG00000228716 | *DHFR* | rs857016 | 5:79975981 | -0.51 | -4.32 | 2.49E-05 | 1.42E-02 |
| ENSG00000228716 | *DHFR* | rs861372 | 5:79976322 | -0.51 | -4.32 | 2.49E-05 | 1.42E-02 |
| ENSG00000251504 | *LINC01099* | rs201283685 | 4:178916406 | -0.47 | -4.32 | 2.49E-05 | 1.42E-02 |
| ENSG00000228716 | *DHFR* | rs844368 | 5:79976382 | -0.51 | -4.32 | 2.49E-05 | 1.42E-02 |
| ENSG00000169071 | *ROR2* | rs7038823 | 9:94697487 | 0.45 | 4.32 | 2.50E-05 | 1.43E-02 |
| ENSG00000169071 | *ROR2* | rs201228079 | 9:94736236 | 0.47 | 4.32 | 2.51E-05 | 1.43E-02 |
| ENSG00000107317 | *PTGDS* | rs28592848 | 9:140213790 | -1.20 | -4.32 | 2.52E-05 | 1.43E-02 |
| ENSG00000107317 | *PTGDS* | rs28375538 | 9:140213793 | -1.20 | -4.32 | 2.52E-05 | 1.43E-02 |
| ENSG00000228716 | *DHFR* | rs2618369 | 5:79978458 | -0.51 | -4.32 | 2.52E-05 | 1.43E-02 |
| ENSG00000228716 | *DHFR* | rs1677707 | 5:79979483 | -0.51 | -4.32 | 2.53E-05 | 1.44E-02 |
| ENSG00000228716 | *DHFR* | rs1650673 | 5:79959733 | -0.63 | -4.32 | 2.54E-05 | 1.44E-02 |
| ENSG00000228716 | *DHFR* | rs1677709 | 5:79980729 | -0.51 | -4.32 | 2.55E-05 | 1.45E-02 |
| ENSG00000228716 | *DHFR* | rs6151620 | 5:79961742 | -0.51 | -4.32 | 2.55E-05 | 1.45E-02 |
| ENSG00000228716 | *DHFR* | rs1650749 | 5:79981544 | -0.51 | -4.32 | 2.56E-05 | 1.45E-02 |
| ENSG00000228716 | *DHFR* | rs1620258 | 5:79982008 | -0.51 | -4.32 | 2.56E-05 | 1.46E-02 |
| ENSG00000113657 | *DPYSL3* | rs17106354 | 5:146647500 | -0.63 | -4.32 | 2.57E-05 | 1.46E-02 |
| ENSG00000171522 | *PTGER4* | rs2218465 | 5:40300291 | 0.41 | 4.32 | 2.57E-05 | 1.46E-02 |
| ENSG00000228716 | *DHFR* | rs1650672 | 5:79959734 | -0.63 | -4.32 | 2.57E-05 | 1.46E-02 |
| ENSG00000228716 | *DHFR* | rs1650747 | 5:79981593 | -0.51 | -4.32 | 2.58E-05 | 1.46E-02 |
| ENSG00000171522 | *PTGER4* | rs70985378 | 5:40365990 | 0.55 | 4.32 | 2.58E-05 | 1.46E-02 |
| ENSG00000228716 | *DHFR* | rs1677630 | 5:79983108 | -0.51 | -4.32 | 2.58E-05 | 1.46E-02 |
| ENSG00000170369 | *CST2* | rs7261217 | 20:23756855 | -0.44 | -4.32 | 2.58E-05 | 1.46E-02 |
| ENSG00000153823 | *PID1* | rs6751176 | 2:230435299 | 0.82 | 4.32 | 2.59E-05 | 1.47E-02 |
| ENSG00000170369 | *CST2* | rs7261342 | 20:23756827 | -0.44 | -4.31 | 2.59E-05 | 1.47E-02 |
| ENSG00000228716 | *DHFR* | rs836802 | 5:79983800 | -0.51 | -4.31 | 2.59E-05 | 1.47E-02 |
| ENSG00000067798 | *NAV3* | rs1491042 | 12:77977759 | 0.51 | 4.31 | 2.61E-05 | 1.48E-02 |
| ENSG00000067798 | *NAV3* | rs79649216 | 12:77975889 | 0.51 | 4.31 | 2.61E-05 | 1.48E-02 |
| ENSG00000067798 | *NAV3* | rs74756108 | 12:77972674 | 0.51 | 4.31 | 2.62E-05 | 1.48E-02 |
| ENSG00000067798 | *NAV3* | rs11105719 | 12:77969714 | 0.51 | 4.31 | 2.62E-05 | 1.48E-02 |
| ENSG00000067798 | *NAV3* | rs1126286 | 12:77982653 | 0.51 | 4.31 | 2.62E-05 | 1.48E-02 |
| ENSG00000067798 | *NAV3* | rs11105804 | 12:77982338 | 0.51 | 4.31 | 2.62E-05 | 1.48E-02 |
| ENSG00000067798 | *NAV3* | rs11105805 | 12:77982400 | 0.51 | 4.31 | 2.62E-05 | 1.48E-02 |
| ENSG00000067798 | *NAV3* | rs11105812 | 12:77983336 | 0.51 | 4.31 | 2.62E-05 | 1.48E-02 |
| ENSG00000067798 | *NAV3* | rs11105813 | 12:77983702 | 0.51 | 4.31 | 2.62E-05 | 1.48E-02 |
| ENSG00000134278 | *SPIRE1* | rs9959611 | 18:12664899 | 0.38 | 4.31 | 2.63E-05 | 1.48E-02 |
| ENSG00000113657 | *DPYSL3* | rs11744519 | 5:146675463 | -0.64 | -4.31 | 2.65E-05 | 1.49E-02 |
| ENSG00000251504 | *LINC01099* | rs6822857 | 4:178985883 | -0.50 | -4.31 | 2.65E-05 | 1.49E-02 |
| ENSG00000112874 | *NUDT12* | rs7443549 | 5:103330031 | 0.46 | 4.31 | 2.65E-05 | 1.50E-02 |
| ENSG00000134278 | *SPIRE1* | rs9950365 | 18:12656108 | 0.38 | 4.31 | 2.66E-05 | 1.50E-02 |
| ENSG00000169071 | *ROR2* | rs6479386 | 9:94694690 | 0.43 | 4.31 | 2.66E-05 | 1.50E-02 |
| ENSG00000169071 | *ROR2* | rs6479385 | 9:94694656 | 0.43 | 4.31 | 2.66E-05 | 1.50E-02 |
| ENSG00000251504 | *LINC01099* | rs10600244 | 4:178986011 | -0.50 | -4.31 | 2.68E-05 | 1.51E-02 |
| ENSG00000169071 | *ROR2* | rs10992154 | 9:94656057 | 0.41 | 4.31 | 2.68E-05 | 1.51E-02 |
| ENSG00000112874 | *NUDT12* | rs12513964 | 5:103338023 | 0.46 | 4.31 | 2.68E-05 | 1.51E-02 |
| ENSG00000171522 | *PTGER4* | rs200902473 | 5:40365989 | 0.55 | 4.31 | 2.68E-05 | 1.51E-02 |
| ENSG00000067798 | *NAV3* | rs139119355 | 12:77981959 | 0.51 | 4.30 | 2.70E-05 | 1.52E-02 |
| ENSG00000228716 | *DHFR* | rs1297578193 | 5:79950741 | -0.49 | -4.30 | 2.70E-05 | 1.52E-02 |
| ENSG00000170369 | *CST2* | rs6049103 | 20:23757164 | -0.43 | -4.30 | 2.74E-05 | 1.54E-02 |
| ENSG00000251504 | *LINC01099* | rs62340563 | 4:178986149 | -0.50 | -4.30 | 2.75E-05 | 1.54E-02 |
| ENSG00000112874 | *NUDT12* | rs66540229 | 5:103347726 | 0.46 | 4.30 | 2.75E-05 | 1.55E-02 |
| ENSG00000251504 | *LINC01099* | rs60892942 | 4:178986351 | -0.50 | -4.30 | 2.76E-05 | 1.55E-02 |
| ENSG00000136108 | *CKAP2* | rs3759509 | 13:53314278 | -0.41 | -4.30 | 2.76E-05 | 1.55E-02 |
| ENSG00000134278 | *SPIRE1* | rs9967437 | 18:12654976 | 0.38 | 4.30 | 2.77E-05 | 1.56E-02 |
| ENSG00000251504 | *LINC01099* | rs6822258 | 4:178985679 | -0.50 | -4.30 | 2.78E-05 | 1.56E-02 |
| ENSG00000134278 | *SPIRE1* | rs1062235 | 18:12658600 | 0.38 | 4.30 | 2.79E-05 | 1.56E-02 |
| ENSG00000154864 | *PIEZO2* | rs508816 | 18:9849620 | 0.47 | 4.30 | 2.79E-05 | 1.56E-02 |
| ENSG00000134278 | *SPIRE1* | rs201456533 | 18:12659266 | 0.38 | 4.30 | 2.79E-05 | 1.56E-02 |
| ENSG00000134278 | *SPIRE1* | rs9949293 | 18:12661279 | 0.38 | 4.30 | 2.80E-05 | 1.57E-02 |
| ENSG00000134278 | *SPIRE1* | rs1129214 | 18:12662148 | 0.38 | 4.30 | 2.81E-05 | 1.57E-02 |
| ENSG00000169071 | *ROR2* | rs1213658425 | 9:94502838 | -0.42 | -4.29 | 2.82E-05 | 1.58E-02 |
| ENSG00000251504 | *LINC01099* | rs1381706 | 4:178986632 | -0.50 | -4.29 | 2.83E-05 | 1.58E-02 |
| ENSG00000112874 | *NUDT12* | rs12654548 | 5:103333147 | 0.46 | 4.29 | 2.84E-05 | 1.59E-02 |
| ENSG00000136108 | *CKAP2* | rs9527003 | 13:53335354 | -0.44 | -4.29 | 2.86E-05 | 1.60E-02 |
| ENSG00000228716 | *DHFR* | rs1650694 | 5:79952426 | -0.49 | -4.29 | 2.86E-05 | 1.60E-02 |
| ENSG00000067798 | *NAV3* | rs76519564 | 12:77974660 | 0.51 | 4.29 | 2.87E-05 | 1.60E-02 |
| ENSG00000136108 | *CKAP2* | rs9316585 | 13:53332352 | -0.43 | -4.29 | 2.89E-05 | 1.61E-02 |
| ENSG00000112874 | *NUDT12* | rs4703279 | 5:103355087 | 0.46 | 4.29 | 2.90E-05 | 1.61E-02 |
| ENSG00000136108 | *CKAP2* | rs4885963 | 13:53331553 | -0.43 | -4.29 | 2.90E-05 | 1.61E-02 |
| ENSG00000134278 | *SPIRE1* | rs9945644 | 18:12663928 | 0.38 | 4.29 | 2.91E-05 | 1.62E-02 |
| ENSG00000228716 | *DHFR* | rs11333499 | 5:80198085 | -0.44 | -4.29 | 2.91E-05 | 1.62E-02 |
| ENSG00000136108 | *CKAP2* | rs1324991 | 13:53333352 | -0.43 | -4.29 | 2.91E-05 | 1.62E-02 |
| ENSG00000228716 | *DHFR* | rs836800 | 5:79985329 | -0.51 | -4.28 | 2.95E-05 | 1.63E-02 |
| ENSG00000228716 | *DHFR* | rs836799 | 5:79985768 | -0.51 | -4.28 | 2.96E-05 | 1.64E-02 |
| ENSG00000228716 | *DHFR* | rs836798 | 5:79985907 | -0.51 | -4.28 | 2.97E-05 | 1.64E-02 |
| ENSG00000170369 | *CST2* | rs4568017 | 20:23767776 | -0.50 | -4.28 | 2.97E-05 | 1.65E-02 |
| ENSG00000228716 | *DHFR* | rs11459232 | 5:80009321 | 0.47 | 4.28 | 2.98E-05 | 1.65E-02 |
| ENSG00000153823 | *PID1* | rs7574727 | 2:230449241 | 0.85 | 4.28 | 2.99E-05 | 1.65E-02 |
| ENSG00000153823 | *PID1* | rs72987903 | 2:230432550 | -0.83 | -4.28 | 3.00E-05 | 1.66E-02 |
| ENSG00000136108 | *CKAP2* | rs9527004 | 13:53335797 | -0.43 | -4.28 | 3.01E-05 | 1.66E-02 |
| ENSG00000228716 | *DHFR* | rs113333527 | 5:79989367 | -0.50 | -4.28 | 3.02E-05 | 1.67E-02 |
| ENSG00000136108 | *CKAP2* | rs4369550 | 13:52744910 | -0.42 | -4.27 | 3.06E-05 | 1.68E-02 |
| ENSG00000174080 | *CTSF* | rs8432 | 11:66299515 | 0.45 | 4.27 | 3.07E-05 | 1.69E-02 |
| ENSG00000112874 | *NUDT12* | rs68024865 | 5:103357634 | 0.46 | 4.27 | 3.07E-05 | 1.69E-02 |
| ENSG00000148344 | *PTGES* | rs11794563 | 9:132044938 | 0.51 | 4.27 | 3.13E-05 | 1.72E-02 |
| ENSG00000174080 | *CTSF* | rs1791686 | 11:66300463 | -0.45 | -4.27 | 3.17E-05 | 1.73E-02 |
| ENSG00000171522 | *PTGER4* | rs113054164 | 5:40330795 | 0.55 | 4.26 | 3.18E-05 | 1.74E-02 |
| ENSG00000228716 | *DHFR* | rs1034168800 | 5:80060355 | 0.53 | 4.26 | 3.20E-05 | 1.74E-02 |
| ENSG00000136108 | *CKAP2* | rs1324990 | 13:53330954 | -0.43 | -4.26 | 3.21E-05 | 1.75E-02 |
| ENSG00000170369 | *CST2* | rs6132672 | 20:23809681 | -0.46 | -4.26 | 3.21E-05 | 1.75E-02 |
| ENSG00000169071 | *ROR2* | rs10116351 | 9:94651702 | 0.41 | 4.26 | 3.22E-05 | 1.75E-02 |
| ENSG00000169071 | *ROR2* | rs4744114 | 9:94652581 | 0.41 | 4.26 | 3.23E-05 | 1.76E-02 |
| ENSG00000169071 | *ROR2* | rs10121787 | 9:94737496 | 0.45 | 4.26 | 3.24E-05 | 1.77E-02 |
| ENSG00000134278 | *SPIRE1* | rs34456643 | 18:12565250 | 0.39 | 4.26 | 3.28E-05 | 1.79E-02 |
| ENSG00000251504 | *LINC01099* | rs3033357 | 4:178986964 | -0.50 | -4.26 | 3.29E-05 | 1.79E-02 |
| ENSG00000228716 | *DHFR* | rs1650687 | 5:79956187 | -0.58 | -4.25 | 3.33E-05 | 1.80E-02 |
| ENSG00000151470 | *C4orf33* | rs318513 | 4:129816821 | 0.60 | 4.25 | 3.35E-05 | 1.82E-02 |
| ENSG00000148344 | *PTGES* | rs7849964 | 9:132050588 | 0.49 | 4.25 | 3.39E-05 | 1.83E-02 |
| ENSG00000067798 | *NAV3* | rs1964608 | 12:77982585 | 0.51 | 4.25 | 3.39E-05 | 1.83E-02 |
| ENSG00000228716 | *DHFR* | rs857263 | 5:80100352 | 0.44 | 4.25 | 3.39E-05 | 1.83E-02 |
| ENSG00000171522 | *PTGER4* | rs2084030 | 5:40355074 | 0.40 | 4.25 | 3.40E-05 | 1.84E-02 |
| ENSG00000153823 | *PID1* | rs67240011 | 2:230424669 | -0.80 | -4.25 | 3.41E-05 | 1.84E-02 |
| ENSG00000148344 | *PTGES* | rs11794624 | 9:132034591 | 0.54 | 4.25 | 3.41E-05 | 1.84E-02 |
| ENSG00000136111 | *TBC1D4* | rs139523306 | 13:75908601 | 0.45 | 4.25 | 3.43E-05 | 1.85E-02 |
| ENSG00000137033 | *IL33* | rs4452851 | 9:5288908 | -0.53 | -4.25 | 3.43E-05 | 1.85E-02 |
| ENSG00000127325 | *BEST3* | rs7137565 | 12:70036048 | 0.43 | 4.25 | 3.44E-05 | 1.85E-02 |
| ENSG00000228716 | *DHFR* | rs2431221 | 5:79934230 | -0.47 | -4.25 | 3.44E-05 | 1.85E-02 |
| ENSG00000112874 | *NUDT12* | rs1420870 | 5:103358752 | 0.46 | 4.25 | 3.44E-05 | 1.86E-02 |
| ENSG00000228716 | *DHFR* | rs73132417 | 5:79957038 | 0.45 | 4.24 | 3.46E-05 | 1.86E-02 |
| ENSG00000134278 | *SPIRE1* | rs12955693 | 18:12660381 | 0.40 | 4.24 | 3.51E-05 | 1.89E-02 |
| ENSG00000140876 | *NUDT7* | rs9929424 | 16:77757718 | 0.62 | 4.24 | 3.54E-05 | 1.90E-02 |
| ENSG00000251504 | *LINC01099* | rs112936976 | 4:178988712 | -0.51 | -4.24 | 3.55E-05 | 1.90E-02 |
| ENSG00000136108 | *CKAP2* | rs3742284 | 13:53175424 | -0.46 | -4.24 | 3.56E-05 | 1.91E-02 |
| ENSG00000228716 | *DHFR* | rs1650684 | 5:79957125 | -0.59 | -4.24 | 3.57E-05 | 1.91E-02 |
| ENSG00000257261 | *RP11-96H19.1* | rs4768697 | 12:46748965 | 0.52 | 4.24 | 3.58E-05 | 1.92E-02 |
| ENSG00000086730 | *LAT2* | rs200184967 | 7:74251390 | 0.72 | 4.24 | 3.59E-05 | 1.92E-02 |
| ENSG00000136108 | *CKAP2* | rs1296352042 | 13:52879236 | -0.45 | -4.23 | 3.62E-05 | 1.94E-02 |
| ENSG00000067798 | *NAV3* | rs1534251 | 12:77970603 | 0.50 | 4.23 | 3.66E-05 | 1.95E-02 |
| ENSG00000136108 | *CKAP2* | rs2016192 | 13:53327795 | -0.43 | -4.23 | 3.66E-05 | 1.96E-02 |
| ENSG00000140876 | *NUDT7* | rs79000636 | 16:77797038 | 0.66 | 4.23 | 3.67E-05 | 1.96E-02 |
| ENSG00000136108 | *CKAP2* | rs7992749 | 13:53327385 | -0.43 | -4.23 | 3.68E-05 | 1.96E-02 |
| ENSG00000136108 | *CKAP2* | rs7330282 | 13:53327139 | -0.43 | -4.23 | 3.69E-05 | 1.97E-02 |
| ENSG00000171522 | *PTGER4* | rs1545334 | 5:40430118 | 0.41 | 4.23 | 3.71E-05 | 1.98E-02 |
| ENSG00000136108 | *CKAP2* | rs7327223 | 13:53330377 | -0.43 | -4.23 | 3.73E-05 | 1.99E-02 |
| ENSG00000251504 | *LINC01099* | rs1585575 | 4:178925963 | 0.46 | 4.23 | 3.73E-05 | 1.99E-02 |
| ENSG00000251504 | *LINC01099* | rs1585576 | 4:178926024 | 0.46 | 4.22 | 3.74E-05 | 1.99E-02 |
| ENSG00000228716 | *DHFR* | rs1028255869 | 5:79944429 | -0.54 | -4.22 | 3.78E-05 | 2.01E-02 |
| ENSG00000069424 | *KCNAB2* | rs3789529 | 1:6153525 | -0.41 | -4.22 | 3.80E-05 | 2.02E-02 |
| ENSG00000228716 | *DHFR* | rs201874762 | 5:79950699 | -0.50 | -4.22 | 3.82E-05 | 2.03E-02 |
| ENSG00000136108 | *CKAP2* | rs5006649 | 13:53323486 | -0.43 | -4.22 | 3.84E-05 | 2.04E-02 |
| ENSG00000172020 | *GAP43* | rs28374180 | 3:116238060 | -0.61 | -4.22 | 3.85E-05 | 2.04E-02 |
| ENSG00000228716 | *DHFR* | rs6151604 | 5:79960175 | 0.44 | 4.22 | 3.85E-05 | 2.04E-02 |
| ENSG00000136108 | *CKAP2* | rs7330117 | 13:53327087 | -0.43 | -4.22 | 3.86E-05 | 2.04E-02 |
| ENSG00000136108 | *CKAP2* | rs1475297 | 13:53326722 | -0.43 | -4.22 | 3.87E-05 | 2.05E-02 |
| ENSG00000179331 | *RAB39A* | rs73000543 | 11:107976426 | 1.02 | 4.21 | 3.91E-05 | 2.07E-02 |
| ENSG00000127325 | *BEST3* | rs139915647 | 12:70094498 | 0.43 | 4.21 | 3.92E-05 | 2.07E-02 |
| ENSG00000136108 | *CKAP2* | rs11419658 | 13:53315445 | -0.43 | -4.21 | 3.92E-05 | 2.07E-02 |
| ENSG00000228716 | *DHFR* | rs245408 | 5:80072451 | 0.58 | 4.21 | 3.92E-05 | 2.07E-02 |
| ENSG00000137033 | *IL33* | rs35188966 | 9:5287734 | -0.52 | -4.21 | 3.93E-05 | 2.08E-02 |
| ENSG00000134278 | *SPIRE1* | rs11080584 | 18:12558095 | 0.38 | 4.21 | 3.93E-05 | 2.08E-02 |
| ENSG00000151470 | *C4orf33* | rs1674974 | 4:129838452 | 0.55 | 4.21 | 3.96E-05 | 2.09E-02 |
| ENSG00000169071 | *ROR2* | rs10992162 | 9:94688429 | 0.45 | 4.21 | 3.97E-05 | 2.09E-02 |
| ENSG00000228716 | *DHFR* | rs6151627 | 5:79965536 | 0.44 | 4.21 | 3.97E-05 | 2.09E-02 |
| ENSG00000228716 | *DHFR* | rs6151629 | 5:79965735 | 0.44 | 4.21 | 3.97E-05 | 2.09E-02 |
| ENSG00000153823 | *PID1* | rs11695348 | 2:230436865 | 0.77 | 4.21 | 3.98E-05 | 2.10E-02 |
| ENSG00000153823 | *PID1* | rs11893092 | 2:230441760 | -0.81 | -4.21 | 3.98E-05 | 2.10E-02 |
| ENSG00000136108 | *CKAP2* | rs9536272 | 13:53320600 | -0.42 | -4.21 | 3.98E-05 | 2.10E-02 |
| ENSG00000228716 | *DHFR* | rs245403 | 5:80069930 | 0.58 | 4.21 | 3.99E-05 | 2.10E-02 |
| ENSG00000127325 | *BEST3* | rs12817803 | 12:70020174 | 0.47 | 4.21 | 4.00E-05 | 2.11E-02 |
| ENSG00000136144 | *RCBTB1* | rs12184585 | 13:49986602 | 0.42 | 4.21 | 4.02E-05 | 2.11E-02 |
| ENSG00000137033 | *IL33* | rs7862237 | 9:5291111 | -0.52 | -4.21 | 4.03E-05 | 2.12E-02 |
| ENSG00000134278 | *SPIRE1* | rs12971041 | 18:12584074 | 0.42 | 4.21 | 4.03E-05 | 2.12E-02 |
| ENSG00000228716 | *DHFR* | rs40300 | 5:80067803 | 0.58 | 4.21 | 4.03E-05 | 2.12E-02 |
| ENSG00000251504 | *LINC01099* | rs55897234 | 4:178897890 | -0.44 | -4.21 | 4.04E-05 | 2.12E-02 |
| ENSG00000257261 | *RP11-96H19.1* | rs11613912 | 12:46739740 | 0.52 | 4.21 | 4.05E-05 | 2.13E-02 |
| ENSG00000151470 | *C4orf33* | rs4975287 | 4:129869058 | 0.43 | 4.20 | 4.05E-05 | 2.13E-02 |
| ENSG00000151470 | *C4orf33* | rs12650832 | 4:129872211 | 0.43 | 4.20 | 4.06E-05 | 2.13E-02 |
| ENSG00000174080 | *CTSF* | rs1671065 | 11:66286377 | 0.44 | 4.20 | 4.06E-05 | 2.13E-02 |
| ENSG00000228716 | *DHFR* | rs62365529 | 5:80225729 | 0.41 | 4.20 | 4.06E-05 | 2.13E-02 |
| ENSG00000151470 | *C4orf33* | rs10857134 | 4:129873433 | 0.43 | 4.20 | 4.07E-05 | 2.13E-02 |
| ENSG00000174080 | *CTSF* | rs10736656 | 11:66285268 | 0.44 | 4.20 | 4.07E-05 | 2.14E-02 |
| ENSG00000151470 | *C4orf33* | rs1288623233 | 4:129876588 | 0.43 | 4.20 | 4.08E-05 | 2.14E-02 |
| ENSG00000251504 | *LINC01099* | rs12152628 | 4:178987563 | -0.49 | -4.20 | 4.08E-05 | 2.14E-02 |
| ENSG00000151470 | *C4orf33* | rs10028124 | 4:129867280 | 0.43 | 4.20 | 4.09E-05 | 2.15E-02 |
| ENSG00000151470 | *C4orf33* | rs4975288 | 4:129873147 | 0.43 | 4.20 | 4.09E-05 | 2.15E-02 |
| ENSG00000174080 | *CTSF* | rs1671062 | 11:66281538 | 0.44 | 4.20 | 4.11E-05 | 2.15E-02 |
| ENSG00000067798 | *NAV3* | rs9669318 | 12:78005051 | 0.51 | 4.20 | 4.11E-05 | 2.15E-02 |
| ENSG00000137033 | *IL33* | rs13301911 | 9:5290749 | -0.52 | -4.20 | 4.17E-05 | 2.18E-02 |
| ENSG00000151470 | *C4orf33* | rs1947269 | 4:129881331 | 0.43 | 4.20 | 4.18E-05 | 2.18E-02 |
| ENSG00000257261 | *RP11-96H19.1* | rs1492888 | 12:46660508 | 0.46 | 4.20 | 4.18E-05 | 2.18E-02 |
| ENSG00000153823 | *PID1* | rs764408 | 2:230451967 | -0.83 | -4.20 | 4.21E-05 | 2.20E-02 |
| ENSG00000153823 | *PID1* | rs6759455 | 2:230437241 | 0.77 | 4.20 | 4.22E-05 | 2.20E-02 |
| ENSG00000228716 | *DHFR* | rs10942935 | 5:80225924 | 0.41 | 4.19 | 4.22E-05 | 2.20E-02 |
| ENSG00000153823 | *PID1* | rs6759476 | 2:230437369 | 0.77 | 4.19 | 4.23E-05 | 2.20E-02 |
| ENSG00000137033 | *IL33* | rs2208687 | 9:5288452 | -0.52 | -4.19 | 4.24E-05 | 2.21E-02 |
| ENSG00000137033 | *IL33* | rs1407374 | 9:5292257 | -0.52 | -4.19 | 4.24E-05 | 2.21E-02 |
| ENSG00000137033 | *IL33* | rs7847238 | 9:5289607 | -0.52 | -4.19 | 4.25E-05 | 2.21E-02 |
| ENSG00000137033 | *IL33* | rs7866280 | 9:5289617 | -0.52 | -4.19 | 4.25E-05 | 2.21E-02 |
| ENSG00000174080 | *CTSF* | rs1671063 | 11:66272142 | 0.44 | 4.19 | 4.25E-05 | 2.21E-02 |
| ENSG00000134278 | *SPIRE1* | rs58215411 | 18:12554125 | 0.38 | 4.19 | 4.25E-05 | 2.21E-02 |
| ENSG00000137033 | *IL33* | rs113899175 | 9:5288916 | -0.52 | -4.19 | 4.26E-05 | 2.21E-02 |
| ENSG00000149328 | *GLB1L2* | rs1144215 | 11:134183450 | -0.42 | -4.19 | 4.27E-05 | 2.22E-02 |
| ENSG00000228716 | *DHFR* | rs1643643 | 5:79948411 | -0.57 | -4.19 | 4.28E-05 | 2.22E-02 |
| ENSG00000151470 | *C4orf33* | rs4975290 | 4:129881527 | 0.43 | 4.19 | 4.30E-05 | 2.23E-02 |
| ENSG00000228716 | *DHFR* | rs1643653 | 5:79954790 | -0.50 | -4.19 | 4.33E-05 | 2.25E-02 |
| ENSG00000228716 | *DHFR* | rs2405880 | 5:79953793 | -0.50 | -4.19 | 4.34E-05 | 2.25E-02 |
| ENSG00000134278 | *SPIRE1* | rs34178879 | 18:12549738 | 0.38 | 4.19 | 4.34E-05 | 2.25E-02 |
| ENSG00000134278 | *SPIRE1* | rs11080582 | 18:12555802 | 0.38 | 4.19 | 4.35E-05 | 2.26E-02 |
| ENSG00000134278 | *SPIRE1* | rs10445428 | 18:12555127 | 0.38 | 4.19 | 4.39E-05 | 2.27E-02 |
| ENSG00000136108 | *CKAP2* | rs370509980 | 13:52954465 | -0.41 | -4.18 | 4.39E-05 | 2.27E-02 |
| ENSG00000127328 | *RAB3IP* | rs147794356 | 12:69239736 | 1.52 | 4.18 | 4.42E-05 | 2.28E-02 |
| ENSG00000134278 | *SPIRE1* | rs12961966 | 18:12552632 | 0.38 | 4.18 | 4.54E-05 | 2.34E-02 |
| ENSG00000069424 | *KCNAB2* | rs1056866 | 1:6161075 | -0.39 | -4.18 | 4.55E-05 | 2.34E-02 |
| ENSG00000228716 | *DHFR* | rs6151616 | 5:79961470 | -0.57 | -4.18 | 4.56E-05 | 2.34E-02 |
| ENSG00000053108 | *FSTL4* | rs17491643 | 5:133167979 | -0.50 | -4.18 | 4.56E-05 | 2.35E-02 |
| ENSG00000134278 | *SPIRE1* | rs10678393 | 18:12672553 | 0.37 | 4.17 | 4.61E-05 | 2.36E-02 |
| ENSG00000228716 | *DHFR* | rs1650660 | 5:79963463 | -0.57 | -4.17 | 4.61E-05 | 2.37E-02 |
| ENSG00000151470 | *C4orf33* | rs6844446 | 4:129745425 | -0.42 | -4.17 | 4.63E-05 | 2.37E-02 |
| ENSG00000171522 | *PTGER4* | rs72257654 | 5:40414965 | 0.46 | 4.17 | 4.64E-05 | 2.38E-02 |
| ENSG00000134278 | *SPIRE1* | rs12955161 | 18:12542121 | 0.38 | 4.17 | 4.64E-05 | 2.38E-02 |
| ENSG00000134278 | *SPIRE1* | rs12953406 | 18:12550908 | 0.38 | 4.17 | 4.65E-05 | 2.38E-02 |
| ENSG00000176533 | *GNG7* | rs12608919 | 19:3543397 | 0.46 | 4.17 | 4.65E-05 | 2.38E-02 |
| ENSG00000148344 | *PTGES* | rs4836651 | 9:132049329 | 0.48 | 4.17 | 4.68E-05 | 2.39E-02 |
| ENSG00000112874 | *NUDT12* | rs11747480 | 5:103358108 | 0.45 | 4.17 | 4.69E-05 | 2.40E-02 |
| ENSG00000151470 | *C4orf33* | rs13132428 | 4:129870798 | 0.43 | 4.17 | 4.71E-05 | 2.40E-02 |
| ENSG00000228716 | *DHFR* | rs34099716 | 5:79955360 | -0.49 | -4.17 | 4.72E-05 | 2.41E-02 |
| ENSG00000148344 | *PTGES* | rs10988290 | 9:132035452 | 0.49 | 4.17 | 4.72E-05 | 2.41E-02 |
| ENSG00000228716 | *DHFR* | rs1643652 | 5:79955079 | -0.49 | -4.17 | 4.73E-05 | 2.41E-02 |
| ENSG00000228716 | *DHFR* | rs1677680 | 5:79955035 | -0.49 | -4.17 | 4.73E-05 | 2.41E-02 |
| ENSG00000170369 | *CST2* | rs6114296 | 20:23777130 | -0.46 | -4.17 | 4.74E-05 | 2.42E-02 |
| ENSG00000228716 | *DHFR* | rs1382542 | 5:79953581 | -0.49 | -4.17 | 4.75E-05 | 2.42E-02 |
| ENSG00000228716 | *DHFR* | rs1047451300 | 5:79953484 | -0.49 | -4.17 | 4.75E-05 | 2.42E-02 |
| ENSG00000228716 | *DHFR* | rs1650692 | 5:79953393 | -0.49 | -4.17 | 4.76E-05 | 2.43E-02 |
| ENSG00000228716 | *DHFR* | rs1650693 | 5:79952827 | -0.49 | -4.17 | 4.76E-05 | 2.43E-02 |
| ENSG00000228716 | *DHFR* | rs1439387644 | 5:79952856 | -0.49 | -4.17 | 4.76E-05 | 2.43E-02 |
| ENSG00000228716 | *DHFR* | rs1650696 | 5:79951280 | -0.49 | -4.17 | 4.76E-05 | 2.43E-02 |
| ENSG00000228716 | *DHFR* | rs1643641 | 5:79951491 | -0.49 | -4.17 | 4.76E-05 | 2.43E-02 |
| ENSG00000228716 | *DHFR* | rs1382539 | 5:79952154 | -0.49 | -4.17 | 4.76E-05 | 2.43E-02 |
| ENSG00000228716 | *DHFR* | rs1382540 | 5:79952165 | -0.49 | -4.17 | 4.76E-05 | 2.43E-02 |
| ENSG00000228716 | *DHFR* | rs1643639 | 5:79952390 | -0.49 | -4.17 | 4.76E-05 | 2.43E-02 |
| ENSG00000228716 | *DHFR* | rs1677667 | 5:79952541 | -0.49 | -4.17 | 4.76E-05 | 2.43E-02 |
| ENSG00000153823 | *PID1* | rs4972903 | 2:230438525 | 0.77 | 4.16 | 4.76E-05 | 2.43E-02 |
| ENSG00000153823 | *PID1* | rs58430280 | 2:230439073 | -0.77 | -4.16 | 4.78E-05 | 2.44E-02 |
| ENSG00000153823 | *PID1* | rs6710000 | 2:230439184 | 0.77 | 4.16 | 4.78E-05 | 2.44E-02 |
| ENSG00000153823 | *PID1* | rs2396693 | 2:230439332 | 0.77 | 4.16 | 4.79E-05 | 2.44E-02 |
| ENSG00000112874 | *NUDT12* | rs460651 | 5:102872552 | -0.40 | -4.16 | 4.80E-05 | 2.44E-02 |
| ENSG00000137033 | *IL33* | rs7851952 | 9:5291086 | -0.51 | -4.16 | 4.80E-05 | 2.44E-02 |
| ENSG00000134278 | *SPIRE1* | rs1330800677 | 18:12658191 | 0.37 | 4.16 | 4.81E-05 | 2.45E-02 |
| ENSG00000148344 | *PTGES* | rs1156726968 | 9:132037440 | 0.53 | 4.16 | 4.82E-05 | 2.45E-02 |
| ENSG00000137033 | *IL33* | rs13293161 | 9:5292743 | -0.52 | -4.16 | 4.84E-05 | 2.46E-02 |
| ENSG00000112874 | *NUDT12* | rs456940 | 5:102874062 | -0.40 | -4.16 | 4.86E-05 | 2.47E-02 |
| ENSG00000112874 | *NUDT12* | rs458304 | 5:102874719 | -0.40 | -4.16 | 4.87E-05 | 2.47E-02 |
| ENSG00000228716 | *DHFR* | rs1650651 | 5:79965580 | -0.50 | -4.16 | 4.89E-05 | 2.48E-02 |
| ENSG00000228716 | *DHFR* | rs6151706 | 5:80004794 | 0.44 | 4.16 | 4.91E-05 | 2.49E-02 |
| ENSG00000124151 | *NCOA3* | rs17484330 | 20:45423221 | -0.64 | -4.16 | 4.94E-05 | 2.50E-02 |
| ENSG00000140876 | *NUDT7* | rs117911381 | 16:77447255 | -1.42 | -4.16 | 4.94E-05 | 2.50E-02 |
| ENSG00000228716 | *DHFR* | rs6151636 | 5:79966797 | -0.57 | -4.15 | 5.05E-05 | 2.55E-02 |
| ENSG00000153823 | *PID1* | rs2052310 | 2:230440448 | 0.77 | 4.15 | 5.05E-05 | 2.55E-02 |
| ENSG00000134121 | *CHL1* | rs2063886 | 3:222904 | -0.48 | -4.15 | 5.06E-05 | 2.55E-02 |
| ENSG00000151470 | *C4orf33* | rs1425618 | 4:129740487 | -0.42 | -4.15 | 5.09E-05 | 2.56E-02 |
| ENSG00000151470 | *C4orf33* | rs1170728180 | 4:129741269 | -0.42 | -4.15 | 5.09E-05 | 2.56E-02 |
| ENSG00000151470 | *C4orf33* | rs6833391 | 4:129742066 | -0.42 | -4.15 | 5.09E-05 | 2.56E-02 |
| ENSG00000228716 | *DHFR* | rs6151651 | 5:79971464 | 0.43 | 4.15 | 5.13E-05 | 2.58E-02 |
| ENSG00000228716 | *DHFR* | rs6151653 | 5:79971612 | 0.43 | 4.15 | 5.13E-05 | 2.58E-02 |
| ENSG00000174080 | *CTSF* | rs1700189 | 11:66258916 | -0.43 | -4.15 | 5.15E-05 | 2.59E-02 |
| ENSG00000228716 | *DHFR* | rs71720287 | 5:79977504 | 0.43 | 4.14 | 5.16E-05 | 2.59E-02 |
| ENSG00000136144 | *RCBTB1* | rs2181328 | 13:49986118 | 0.41 | 4.14 | 5.21E-05 | 2.61E-02 |
| ENSG00000228716 | *DHFR* | rs6151670 | 5:79983215 | 0.44 | 4.14 | 5.22E-05 | 2.62E-02 |
| ENSG00000151470 | *C4orf33* | rs6821814 | 4:129883052 | 0.43 | 4.14 | 5.22E-05 | 2.62E-02 |
| ENSG00000069424 | *KCNAB2* | rs2229002 | 1:6158562 | -0.39 | -4.14 | 5.25E-05 | 2.63E-02 |
| ENSG00000121898 | *CPXM2* | rs28705282 | 10:125642428 | -0.51 | -4.14 | 5.25E-05 | 2.63E-02 |
| ENSG00000228716 | *DHFR* | rs6861850 | 5:79982350 | 0.44 | 4.14 | 5.31E-05 | 2.65E-02 |
| ENSG00000228716 | *DHFR* | rs199896299 | 5:80171132 | 0.46 | 4.13 | 5.37E-05 | 2.68E-02 |
| ENSG00000069424 | *KCNAB2* | rs6696489 | 1:6162054 | -0.48 | -4.13 | 5.40E-05 | 2.69E-02 |
| ENSG00000151470 | *C4orf33* | rs4975264 | 4:129738176 | -0.41 | -4.13 | 5.42E-05 | 2.70E-02 |
| ENSG00000151470 | *C4orf33* | rs4975291 | 4:129883221 | 0.43 | 4.13 | 5.42E-05 | 2.70E-02 |
| ENSG00000134853 | *PDGFRA* | rs12642334 | 4:55952299 | 0.53 | 4.13 | 5.44E-05 | 2.70E-02 |
| ENSG00000121898 | *CPXM2* | rs7893701 | 10:125640142 | 0.64 | 4.13 | 5.46E-05 | 2.71E-02 |
| ENSG00000112874 | *NUDT12* | rs251163 | 5:102866217 | -0.40 | -4.13 | 5.48E-05 | 2.72E-02 |
| ENSG00000153823 | *PID1* | rs6706536 | 2:230438663 | 0.77 | 4.13 | 5.49E-05 | 2.73E-02 |
| ENSG00000228716 | *DHFR* | rs3776972 | 5:80007751 | 0.44 | 4.13 | 5.56E-05 | 2.75E-02 |
| ENSG00000228716 | *DHFR* | rs6864493 | 5:79985702 | 0.44 | 4.13 | 5.57E-05 | 2.76E-02 |
| ENSG00000141404 | *GNAL* | rs6505679 | 18:11890168 | 0.55 | 4.12 | 5.59E-05 | 2.77E-02 |
| ENSG00000151470 | *C4orf33* | rs318555 | 4:129798627 | 0.43 | 4.12 | 5.61E-05 | 2.77E-02 |
| ENSG00000228716 | *DHFR* | rs245404 | 5:80071071 | 0.44 | 4.12 | 5.74E-05 | 2.83E-02 |
| ENSG00000127325 | *BEST3* | rs7308313 | 12:70037906 | 0.42 | 4.12 | 5.76E-05 | 2.83E-02 |
| ENSG00000228716 | *DHFR* | rs6151707 | 5:80012872 | 0.44 | 4.12 | 5.77E-05 | 2.84E-02 |
| ENSG00000127325 | *BEST3* | rs7958416 | 12:70063970 | 0.42 | 4.12 | 5.78E-05 | 2.84E-02 |
| ENSG00000228716 | *DHFR* | rs35756839 | 5:79959600 | -0.49 | -4.12 | 5.80E-05 | 2.85E-02 |
| ENSG00000127325 | *BEST3* | rs3847773 | 12:70065072 | 0.42 | 4.12 | 5.81E-05 | 2.86E-02 |
| ENSG00000127325 | *BEST3* | rs3847772 | 12:70064295 | 0.42 | 4.11 | 5.83E-05 | 2.86E-02 |
| ENSG00000127325 | *BEST3* | rs17813545 | 12:70064146 | 0.42 | 4.11 | 5.84E-05 | 2.87E-02 |
| ENSG00000127325 | *BEST3* | rs4761164 | 12:70063938 | 0.42 | 4.11 | 5.84E-05 | 2.87E-02 |
| ENSG00000148344 | *PTGES* | rs10819484 | 9:132032637 | -0.47 | -4.11 | 5.85E-05 | 2.87E-02 |
| ENSG00000148344 | *PTGES* | rs10760602 | 9:132032717 | -0.47 | -4.11 | 5.85E-05 | 2.87E-02 |
| ENSG00000228716 | *DHFR* | rs26283 | 5:80051463 | 0.57 | 4.11 | 5.93E-05 | 2.90E-02 |
| ENSG00000228716 | *DHFR* | rs3836866 | 5:80017342 | 0.44 | 4.11 | 5.95E-05 | 2.91E-02 |
| ENSG00000257261 | *RP11-96H19.1* | rs10880962 | 12:46773444 | 0.40 | 4.11 | 5.97E-05 | 2.92E-02 |
| ENSG00000228716 | *DHFR* | rs140127524 | 5:80040727 | 0.44 | 4.11 | 5.97E-05 | 2.92E-02 |
| ENSG00000228716 | *DHFR* | rs32960 | 5:80041918 | 0.44 | 4.11 | 5.97E-05 | 2.92E-02 |
| ENSG00000228716 | *DHFR* | rs41097 | 5:80019172 | 0.44 | 4.11 | 5.99E-05 | 2.92E-02 |
| ENSG00000228716 | *DHFR* | rs26780 | 5:80053255 | 0.44 | 4.11 | 6.01E-05 | 2.93E-02 |
| ENSG00000136108 | *CKAP2* | rs9536275 | 13:53323618 | -0.39 | -4.11 | 6.02E-05 | 2.94E-02 |
| ENSG00000148344 | *PTGES* | rs883343 | 9:132033730 | -0.47 | -4.11 | 6.02E-05 | 2.94E-02 |
| ENSG00000153823 | *PID1* | rs2396691 | 2:230438057 | 0.72 | 4.11 | 6.02E-05 | 2.94E-02 |
| ENSG00000153823 | *PID1* | rs2396692 | 2:230438058 | 0.72 | 4.11 | 6.02E-05 | 2.94E-02 |
| ENSG00000228716 | *DHFR* | rs26781 | 5:80052766 | 0.44 | 4.11 | 6.02E-05 | 2.94E-02 |
| ENSG00000228716 | *DHFR* | rs26782 | 5:80052419 | 0.44 | 4.11 | 6.02E-05 | 2.94E-02 |
| ENSG00000228716 | *DHFR* | rs28059 | 5:80038965 | 0.44 | 4.11 | 6.02E-05 | 2.94E-02 |
| ENSG00000228716 | *DHFR* | rs245398 | 5:80046374 | 0.44 | 4.11 | 6.02E-05 | 2.94E-02 |
| ENSG00000228716 | *DHFR* | rs6151747 | 5:80037636 | 0.44 | 4.11 | 6.02E-05 | 2.94E-02 |
| ENSG00000228716 | *DHFR* | rs245336 | 5:80028301 | 0.44 | 4.11 | 6.02E-05 | 2.94E-02 |
| ENSG00000169071 | *ROR2* | rs7866775 | 9:94684859 | 0.44 | 4.11 | 6.02E-05 | 2.94E-02 |
| ENSG00000148344 | *PTGES* | rs883342 | 9:132033760 | -0.47 | -4.11 | 6.03E-05 | 2.94E-02 |
| ENSG00000228716 | *DHFR* | rs245014 | 5:80024434 | 0.44 | 4.11 | 6.03E-05 | 2.94E-02 |
| ENSG00000228716 | *DHFR* | rs151886 | 5:80024077 | 0.44 | 4.11 | 6.03E-05 | 2.94E-02 |
| ENSG00000228716 | *DHFR* | rs32951 | 5:80021168 | 0.44 | 4.11 | 6.03E-05 | 2.94E-02 |
| ENSG00000169071 | *ROR2* | rs10820917 | 9:94685761 | 0.44 | 4.11 | 6.03E-05 | 2.94E-02 |
| ENSG00000169071 | *ROR2* | rs10820919 | 9:94686470 | 0.44 | 4.11 | 6.04E-05 | 2.95E-02 |
| ENSG00000169071 | *ROR2* | rs10992165 | 9:94689777 | 0.44 | 4.10 | 6.06E-05 | 2.95E-02 |
| ENSG00000151470 | *C4orf33* | rs13127734 | 4:129882023 | 0.54 | 4.10 | 6.10E-05 | 2.97E-02 |
| ENSG00000148344 | *PTGES* | rs10819483 | 9:132031837 | -0.47 | -4.10 | 6.10E-05 | 2.97E-02 |
| ENSG00000148344 | *PTGES* | rs10988288 | 9:132031764 | -0.47 | -4.10 | 6.10E-05 | 2.97E-02 |
| ENSG00000148344 | *PTGES* | rs7046889 | 9:132031742 | -0.47 | -4.10 | 6.10E-05 | 2.97E-02 |
| ENSG00000228716 | *DHFR* | rs245400 | 5:80047758 | 0.57 | 4.10 | 6.11E-05 | 2.97E-02 |
| ENSG00000148344 | *PTGES* | rs7046279 | 9:132031535 | -0.47 | -4.10 | 6.11E-05 | 2.97E-02 |
| ENSG00000148344 | *PTGES* | rs7870137 | 9:132031138 | -0.47 | -4.10 | 6.12E-05 | 2.98E-02 |
| ENSG00000148344 | *PTGES* | rs913773 | 9:132033466 | -0.47 | -4.10 | 6.12E-05 | 2.98E-02 |
| ENSG00000148344 | *PTGES* | rs10119824 | 9:132030687 | -0.47 | -4.10 | 6.13E-05 | 2.98E-02 |
| ENSG00000173918 | *C1QTNF1* | rs62063816 | 17:77073237 | 0.76 | 4.10 | 6.13E-05 | 2.98E-02 |
| ENSG00000228716 | *DHFR* | rs245399 | 5:80046904 | 0.57 | 4.10 | 6.13E-05 | 2.98E-02 |
| ENSG00000148344 | *PTGES* | rs10819481 | 9:132029868 | -0.47 | -4.10 | 6.14E-05 | 2.99E-02 |
| ENSG00000154864 | *PIEZO2* | rs592170 | 18:9849009 | 0.42 | 4.10 | 6.16E-05 | 2.99E-02 |
| ENSG00000121898 | *CPXM2* | rs28658074 | 10:125631631 | 0.50 | 4.10 | 6.18E-05 | 3.00E-02 |
| ENSG00000174348 | *PODN* | rs41294760 | 1:53814186 | 1.04 | 4.10 | 6.19E-05 | 3.00E-02 |
| ENSG00000228716 | *DHFR* | rs150234858 | 5:79942375 | -0.56 | -4.10 | 6.21E-05 | 3.01E-02 |
| ENSG00000137033 | *IL33* | rs1327403746 | 9:5296098 | -0.51 | -4.10 | 6.22E-05 | 3.01E-02 |
| ENSG00000148344 | *PTGES* | rs17517009 | 9:132038331 | 0.50 | 4.10 | 6.25E-05 | 3.02E-02 |
| ENSG00000184220 | *CMSS1* | rs62283523 | 3:99551325 | -0.40 | -4.10 | 6.25E-05 | 3.02E-02 |
| ENSG00000148344 | *PTGES* | rs4836648 | 9:132048664 | 0.48 | 4.10 | 6.25E-05 | 3.02E-02 |
| ENSG00000228716 | *DHFR* | rs32958 | 5:80043076 | 0.57 | 4.10 | 6.25E-05 | 3.02E-02 |
| ENSG00000004776 | *HSPB6* | rs199534880 | 19:36211374 | 0.97 | 4.10 | 6.27E-05 | 3.03E-02 |
| ENSG00000257261 | *RP11-96H19.1* | rs11284537 | 12:46786939 | 0.40 | 4.10 | 6.27E-05 | 3.03E-02 |
| ENSG00000134278 | *SPIRE1* | rs12957151 | 18:12576001 | 0.37 | 4.09 | 6.30E-05 | 3.04E-02 |
| ENSG00000101463 | *SYNDIG1* | rs13038938 | 20:24601943 | -0.53 | -4.09 | 6.32E-05 | 3.05E-02 |
| ENSG00000228716 | *DHFR* | rs26266 | 5:80040694 | 0.57 | 4.09 | 6.33E-05 | 3.05E-02 |
| ENSG00000166387 | *PPFIBP2* | rs10839805 | 11:7572567 | -0.43 | -4.09 | 6.33E-05 | 3.05E-02 |
| ENSG00000134278 | *SPIRE1* | rs12967204 | 18:12648084 | 0.41 | 4.09 | 6.35E-05 | 3.06E-02 |
| ENSG00000148344 | *PTGES* | rs35980170 | 9:132038380 | 0.50 | 4.09 | 6.36E-05 | 3.07E-02 |
| ENSG00000004776 | *HSPB6* | rs59531859 | 19:36292754 | 0.83 | 4.09 | 6.37E-05 | 3.07E-02 |
| ENSG00000112874 | *NUDT12* | rs10057908 | 5:102884933 | 0.40 | 4.09 | 6.37E-05 | 3.07E-02 |
| ENSG00000228716 | *DHFR* | rs836803 | 5:79977569 | -0.57 | -4.09 | 6.38E-05 | 3.07E-02 |
| ENSG00000253669 | *KB-1732A1.1* | rs2513911 | 8:103817865 | -0.44 | -4.09 | 6.39E-05 | 3.08E-02 |
| ENSG00000251504 | *LINC01099* | rs62340565 | 4:178988575 | -0.48 | -4.09 | 6.41E-05 | 3.08E-02 |
| ENSG00000228716 | *DHFR* | rs26268 | 5:80036469 | -0.57 | -4.09 | 6.41E-05 | 3.09E-02 |
| ENSG00000171522 | *PTGER4* | rs6882977 | 5:40471460 | 0.40 | 4.09 | 6.41E-05 | 3.09E-02 |
| ENSG00000121898 | *CPXM2* | rs28741041 | 10:125650709 | -0.42 | -4.09 | 6.42E-05 | 3.09E-02 |
| ENSG00000151470 | *C4orf33* | rs4975268 | 4:129751768 | -0.42 | -4.09 | 6.44E-05 | 3.10E-02 |
| ENSG00000127325 | *BEST3* | rs7309007 | 12:70038290 | 0.41 | 4.09 | 6.44E-05 | 3.10E-02 |
| ENSG00000228716 | *DHFR* | rs28026 | 5:80036668 | -0.57 | -4.09 | 6.46E-05 | 3.11E-02 |
| ENSG00000151470 | *C4orf33* | rs201645085 | 4:129884000 | 0.43 | 4.09 | 6.48E-05 | 3.11E-02 |
| ENSG00000127325 | *BEST3* | rs57066354 | 12:70021771 | 0.45 | 4.09 | 6.54E-05 | 3.13E-02 |
| ENSG00000251504 | *LINC01099* | rs74271914 | 4:178988715 | -0.48 | -4.09 | 6.54E-05 | 3.13E-02 |
| ENSG00000228716 | *DHFR* | rs836820 | 5:79946380 | -0.52 | -4.08 | 6.55E-05 | 3.14E-02 |
| ENSG00000127325 | *BEST3* | rs55744725 | 12:70024547 | 0.45 | 4.08 | 6.56E-05 | 3.14E-02 |
| ENSG00000151470 | *C4orf33* | rs2777823 | 4:129990743 | -0.43 | -4.08 | 6.56E-05 | 3.14E-02 |
| ENSG00000053108 | *FSTL4* | rs4958254 | 5:133180095 | -0.49 | -4.08 | 6.57E-05 | 3.15E-02 |
| ENSG00000228716 | *DHFR* | rs27365 | 5:80034940 | -0.57 | -4.08 | 6.58E-05 | 3.15E-02 |
| ENSG00000067798 | *NAV3* | rs11105493 | 12:77909438 | 0.49 | 4.08 | 6.59E-05 | 3.16E-02 |
| ENSG00000067798 | *NAV3* | rs10506751 | 12:77911322 | 0.49 | 4.08 | 6.59E-05 | 3.16E-02 |
| ENSG00000169071 | *ROR2* | rs58953970 | 9:94571233 | 0.74 | 4.08 | 6.60E-05 | 3.16E-02 |
| ENSG00000151470 | *C4orf33* | rs371740799 | 4:129780717 | -0.42 | -4.08 | 6.61E-05 | 3.16E-02 |
| ENSG00000121898 | *CPXM2* | rs1996432 | 10:125649185 | -0.43 | -4.08 | 6.62E-05 | 3.17E-02 |
| ENSG00000257261 | *RP11-96H19.1* | rs79350141 | 12:46792423 | 0.40 | 4.08 | 6.64E-05 | 3.17E-02 |
| ENSG00000169071 | *ROR2* | rs76397354 | 9:94574872 | 0.86 | 4.08 | 6.64E-05 | 3.18E-02 |
| ENSG00000136108 | *CKAP2* | rs1815349 | 13:52785881 | -0.43 | -4.08 | 6.65E-05 | 3.18E-02 |
| ENSG00000228716 | *DHFR* | rs1650716 | 5:79930580 | -0.61 | -4.08 | 6.65E-05 | 3.18E-02 |
| ENSG00000169071 | *ROR2* | rs73513287 | 9:94573919 | 0.85 | 4.08 | 6.75E-05 | 3.22E-02 |
| ENSG00000140876 | *NUDT7* | rs147576392 | 16:77458451 | -1.28 | -4.08 | 6.78E-05 | 3.23E-02 |
| ENSG00000115590 | *IL1R2* | rs4850993 | 2:102622466 | -0.38 | -4.08 | 6.78E-05 | 3.23E-02 |
| ENSG00000115590 | *IL1R2* | rs4851527 | 2:102622376 | -0.38 | -4.08 | 6.78E-05 | 3.23E-02 |
| ENSG00000115590 | *IL1R2* | rs719248 | 2:102623875 | -0.38 | -4.08 | 6.78E-05 | 3.23E-02 |
| ENSG00000137033 | *IL33* | rs2381217 | 9:5289943 | -0.50 | -4.08 | 6.80E-05 | 3.24E-02 |
| ENSG00000112874 | *NUDT12* | rs159168 | 5:102865205 | -0.43 | -4.07 | 6.82E-05 | 3.24E-02 |
| ENSG00000148344 | *PTGES* | rs3928781 | 9:132025297 | -0.47 | -4.07 | 6.84E-05 | 3.25E-02 |
| ENSG00000141404 | *GNAL* | rs9303742 | 18:11689522 | 0.48 | 4.07 | 6.86E-05 | 3.26E-02 |
| ENSG00000115590 | *IL1R2* | rs2110563 | 2:102628433 | -0.38 | -4.07 | 6.86E-05 | 3.26E-02 |
| ENSG00000151470 | *C4orf33* | rs72933964 | 4:130133550 | 0.68 | 4.07 | 6.90E-05 | 3.28E-02 |
| ENSG00000127325 | *BEST3* | rs3886392 | 12:70021380 | 0.45 | 4.07 | 6.90E-05 | 3.28E-02 |
| ENSG00000121898 | *CPXM2* | rs28513406 | 10:125650793 | -0.42 | -4.07 | 6.91E-05 | 3.28E-02 |
| ENSG00000004776 | *HSPB6* | rs2272537 | 19:36195586 | 0.96 | 4.07 | 6.91E-05 | 3.28E-02 |
| ENSG00000134278 | *SPIRE1* | rs12962340 | 18:12272923 | 0.47 | 4.07 | 6.91E-05 | 3.28E-02 |
| ENSG00000121898 | *CPXM2* | rs61861909 | 10:125641025 | -0.50 | -4.07 | 6.91E-05 | 3.28E-02 |
| ENSG00000121898 | *CPXM2* | rs28673572 | 10:125641862 | -0.50 | -4.07 | 6.92E-05 | 3.28E-02 |
| ENSG00000228716 | *DHFR* | rs193499 | 5:80098240 | 0.58 | 4.07 | 6.94E-05 | 3.29E-02 |
| ENSG00000174080 | *CTSF* | rs3018318 | 11:66340968 | 0.41 | 4.07 | 6.94E-05 | 3.29E-02 |
| ENSG00000112874 | *NUDT12* | rs294098 | 5:102875560 | -0.42 | -4.07 | 6.96E-05 | 3.30E-02 |
| ENSG00000134278 | *SPIRE1* | rs34537593 | 18:12727596 | 0.40 | 4.07 | 7.00E-05 | 3.31E-02 |
| ENSG00000186340 | *THBS2* | rs116978104 | 6:169092400 | 1.74 | 4.07 | 7.00E-05 | 3.31E-02 |
| ENSG00000134278 | *SPIRE1* | rs34040888 | 18:12727143 | 0.40 | 4.07 | 7.00E-05 | 3.31E-02 |
| ENSG00000253669 | *KB-1732A1.1* | rs2436850 | 8:103827380 | -0.44 | -4.07 | 7.01E-05 | 3.32E-02 |
| ENSG00000136108 | *CKAP2* | rs1865877 | 13:52782894 | -0.42 | -4.07 | 7.01E-05 | 3.32E-02 |
| ENSG00000153823 | *PID1* | rs6760456 | 2:230425104 | -0.74 | -4.07 | 7.01E-05 | 3.32E-02 |
| ENSG00000251504 | *LINC01099* | rs62340566 | 4:178988922 | -0.48 | -4.07 | 7.07E-05 | 3.34E-02 |
| ENSG00000134278 | *SPIRE1* | rs35322088 | 18:12653940 | 0.39 | 4.07 | 7.08E-05 | 3.34E-02 |
| ENSG00000121898 | *CPXM2* | rs28445838 | 10:125650370 | -0.43 | -4.06 | 7.09E-05 | 3.35E-02 |
| ENSG00000148344 | *PTGES* | rs913769 | 9:132044584 | 0.48 | 4.06 | 7.10E-05 | 3.35E-02 |
| ENSG00000112874 | *NUDT12* | rs251159 | 5:102869509 | -0.42 | -4.06 | 7.12E-05 | 3.36E-02 |
| ENSG00000171522 | *PTGER4* | rs1483675582 | 5:40445550 | -0.40 | -4.06 | 7.13E-05 | 3.36E-02 |
| ENSG00000149328 | *GLB1L2* | rs579409 | 11:134184715 | -0.38 | -4.06 | 7.15E-05 | 3.37E-02 |
| ENSG00000115590 | *IL1R2* | rs3819369 | 2:102632582 | -0.38 | -4.06 | 7.16E-05 | 3.37E-02 |
| ENSG00000180875 | *GREM2* | rs149509706 | 1:240288098 | -0.75 | -4.06 | 7.17E-05 | 3.38E-02 |
| ENSG00000053108 | *FSTL4* | rs13189397 | 5:133184083 | -0.48 | -4.06 | 7.18E-05 | 3.38E-02 |
| ENSG00000174080 | *CTSF* | rs487444 | 11:66319313 | 0.41 | 4.06 | 7.18E-05 | 3.38E-02 |
| ENSG00000115590 | *IL1R2* | rs1978330 | 2:102768855 | -0.39 | -4.06 | 7.19E-05 | 3.38E-02 |
| ENSG00000127325 | *BEST3* | rs12812948 | 12:70022696 | 0.44 | 4.06 | 7.20E-05 | 3.39E-02 |
| ENSG00000149328 | *GLB1L2* | rs512958 | 11:134185832 | -0.38 | -4.06 | 7.20E-05 | 3.39E-02 |
| ENSG00000228716 | *DHFR* | rs1355340077 | 5:80122658 | 0.41 | 4.06 | 7.24E-05 | 3.40E-02 |
| ENSG00000153822 | *KCNJ16* | rs138740926 | 17:68891029 | -1.45 | -4.06 | 7.25E-05 | 3.41E-02 |
| ENSG00000127325 | *BEST3* | rs11177756 | 12:70021508 | 0.44 | 4.06 | 7.25E-05 | 3.41E-02 |
| ENSG00000149328 | *GLB1L2* | rs578825 | 11:134188414 | -0.39 | -4.06 | 7.29E-05 | 3.42E-02 |
| ENSG00000148344 | *PTGES* | rs1556143 | 9:132022788 | -0.47 | -4.06 | 7.30E-05 | 3.43E-02 |
| ENSG00000149328 | *GLB1L2* | rs61908675 | 11:134220859 | -0.46 | -4.06 | 7.31E-05 | 3.43E-02 |
| ENSG00000151470 | *C4orf33* | rs141532432 | 4:130134840 | 0.67 | 4.06 | 7.34E-05 | 3.44E-02 |
| ENSG00000134278 | *SPIRE1* | rs12970964 | 18:12408230 | 0.42 | 4.06 | 7.36E-05 | 3.45E-02 |
| ENSG00000142677 | *IL22RA1* | rs11805096 | 1:24828929 | -0.74 | -4.06 | 7.36E-05 | 3.45E-02 |
| ENSG00000171522 | *PTGER4* | rs6874571 | 5:40418741 | 0.40 | 4.05 | 7.39E-05 | 3.46E-02 |
| ENSG00000112874 | *NUDT12* | rs72773269 | 5:103390171 | 0.59 | 4.05 | 7.39E-05 | 3.46E-02 |
| ENSG00000257261 | *RP11-96H19.1* | rs2279559 | 12:46767175 | 0.39 | 4.05 | 7.40E-05 | 3.47E-02 |
| ENSG00000134278 | *SPIRE1* | rs948319 | 18:12717272 | 0.36 | 4.05 | 7.42E-05 | 3.47E-02 |
| ENSG00000253669 | *KB-1732A1.1* | rs36201829 | 8:103821467 | -0.43 | -4.05 | 7.43E-05 | 3.48E-02 |
| ENSG00000228716 | *DHFR* | rs201170046 | 5:80098239 | 0.53 | 4.05 | 7.45E-05 | 3.48E-02 |
| ENSG00000180011 | *ZADH2* | rs9964091 | 18:73744389 | -1.15 | -4.05 | 7.45E-05 | 3.48E-02 |
| ENSG00000166387 | *PPFIBP2* | rs6578871 | 11:7571717 | -0.42 | -4.05 | 7.45E-05 | 3.48E-02 |
| ENSG00000151470 | *C4orf33* | rs280598 | 4:129944940 | -0.42 | -4.05 | 7.47E-05 | 3.49E-02 |
| ENSG00000142677 | *IL22RA1* | rs6687456 | 1:24827282 | -0.74 | -4.05 | 7.47E-05 | 3.49E-02 |
| ENSG00000127325 | *BEST3* | rs59589108 | 12:70022172 | 0.44 | 4.05 | 7.49E-05 | 3.50E-02 |
| ENSG00000142677 | *IL22RA1* | rs85063 | 1:24857503 | -0.39 | -4.05 | 7.50E-05 | 3.50E-02 |
| ENSG00000076513 | *ANKRD13A* | rs146769225 | 12:111328361 | 1.48 | 4.05 | 7.52E-05 | 3.51E-02 |
| ENSG00000151470 | *C4orf33* | rs6830229 | 4:129884609 | 0.42 | 4.05 | 7.53E-05 | 3.51E-02 |
| ENSG00000142677 | *IL22RA1* | rs9424375 | 1:24831643 | -0.74 | -4.05 | 7.54E-05 | 3.52E-02 |
| ENSG00000142677 | *IL22RA1* | rs80007720 | 1:24831663 | -0.75 | -4.05 | 7.58E-05 | 3.53E-02 |
| ENSG00000142677 | *IL22RA1* | rs7540336 | 1:24832734 | -0.74 | -4.05 | 7.61E-05 | 3.54E-02 |
| ENSG00000251504 | *LINC01099* | rs7679755 | 4:178988248 | -0.47 | -4.05 | 7.63E-05 | 3.55E-02 |
| ENSG00000173918 | *C1QTNF1* | rs62063770 | 17:77063734 | 0.76 | 4.05 | 7.66E-05 | 3.56E-02 |
| ENSG00000127325 | *BEST3* | rs11177757 | 12:70021670 | 0.44 | 4.04 | 7.67E-05 | 3.56E-02 |
| ENSG00000112874 | *NUDT12* | rs463332 | 5:102870930 | -0.42 | -4.04 | 7.67E-05 | 3.56E-02 |
| ENSG00000134278 | *SPIRE1* | rs1344894992 | 18:12670649 | 0.39 | 4.04 | 7.70E-05 | 3.57E-02 |
| ENSG00000112874 | *NUDT12* | rs1356519347 | 5:103358106 | 0.44 | 4.04 | 7.71E-05 | 3.58E-02 |
| ENSG00000228716 | *DHFR* | rs857287 | 5:80098957 | 0.49 | 4.04 | 7.71E-05 | 3.58E-02 |
| ENSG00000149328 | *GLB1L2* | rs513412 | 11:134194937 | -0.38 | -4.04 | 7.71E-05 | 3.58E-02 |
| ENSG00000148344 | *PTGES* | rs7035167 | 9:132037505 | 0.50 | 4.04 | 7.72E-05 | 3.58E-02 |
| ENSG00000148344 | *PTGES* | rs1201598199 | 9:132049842 | 0.48 | 4.04 | 7.73E-05 | 3.59E-02 |
| ENSG00000173918 | *C1QTNF1* | rs57024531 | 17:77060103 | 0.76 | 4.04 | 7.77E-05 | 3.60E-02 |
| ENSG00000153823 | *PID1* | rs2216250 | 2:230439906 | 0.72 | 4.04 | 7.79E-05 | 3.61E-02 |
| ENSG00000166387 | *PPFIBP2* | rs7122137 | 11:7570607 | -0.42 | -4.04 | 7.81E-05 | 3.62E-02 |
| ENSG00000166387 | *PPFIBP2* | rs7125735 | 11:7570972 | -0.42 | -4.04 | 7.82E-05 | 3.62E-02 |
| ENSG00000151470 | *C4orf33* | rs55829553 | 4:129755837 | -0.41 | -4.04 | 7.85E-05 | 3.63E-02 |
| ENSG00000136108 | *CKAP2* | rs4885955 | 13:53318387 | -0.43 | -4.04 | 7.87E-05 | 3.64E-02 |
| ENSG00000168566 | *SNRNP48* | rs72817847 | 6:7989018 | -1.76 | -4.04 | 7.88E-05 | 3.64E-02 |
| ENSG00000127325 | *BEST3* | rs11177758 | 12:70021696 | 0.44 | 4.04 | 7.89E-05 | 3.64E-02 |
| ENSG00000173918 | *C1QTNF1* | rs115925079 | 17:77061305 | 0.76 | 4.04 | 7.89E-05 | 3.64E-02 |
| ENSG00000134278 | *SPIRE1* | rs7234697 | 18:12576512 | 0.37 | 4.04 | 7.91E-05 | 3.65E-02 |
| ENSG00000186340 | *THBS2* | rs117693877 | 6:169129773 | 1.73 | 4.04 | 7.91E-05 | 3.65E-02 |
| ENSG00000112874 | *NUDT12* | rs159165 | 5:102866121 | -0.42 | -4.04 | 7.92E-05 | 3.65E-02 |
| ENSG00000151470 | *C4orf33* | rs1709416 | 4:130031650 | -0.42 | -4.04 | 7.92E-05 | 3.66E-02 |
| ENSG00000053372 | *MRTO4* | rs6677903 | 1:18767738 | 0.61 | 4.04 | 7.93E-05 | 3.66E-02 |
| ENSG00000053372 | *MRTO4* | rs17490601 | 1:18768577 | 0.61 | 4.04 | 7.93E-05 | 3.66E-02 |
| ENSG00000134278 | *SPIRE1* | rs12963325 | 18:12577424 | 0.36 | 4.04 | 7.94E-05 | 3.66E-02 |
| ENSG00000053372 | *MRTO4* | rs6660438 | 1:18767827 | 0.61 | 4.04 | 7.95E-05 | 3.66E-02 |
| ENSG00000228716 | *DHFR* | rs857290 | 5:80099066 | 0.49 | 4.03 | 7.97E-05 | 3.67E-02 |
| ENSG00000228716 | *DHFR* | rs857289 | 5:80099055 | 0.49 | 4.03 | 7.98E-05 | 3.68E-02 |
| ENSG00000134278 | *SPIRE1* | rs12957688 | 18:12576133 | 0.37 | 4.03 | 7.99E-05 | 3.68E-02 |
| ENSG00000124151 | *NCOA3* | rs6018020 | 20:45370172 | -0.67 | -4.03 | 8.00E-05 | 3.68E-02 |
| ENSG00000186340 | *THBS2* | rs202237913 | 6:169115117 | 1.73 | 4.03 | 8.04E-05 | 3.70E-02 |
| ENSG00000228716 | *DHFR* | rs192228465 | 5:79988320 | 0.50 | 4.03 | 8.04E-05 | 3.70E-02 |
| ENSG00000186340 | *THBS2* | rs200843859 | 6:169115125 | 1.73 | 4.03 | 8.04E-05 | 3.70E-02 |
| ENSG00000127325 | *BEST3* | rs7131770 | 12:70039958 | 0.41 | 4.03 | 8.05E-05 | 3.70E-02 |
| ENSG00000173918 | *C1QTNF1* | rs55979313 | 17:77059498 | 0.76 | 4.03 | 8.07E-05 | 3.71E-02 |
| ENSG00000228716 | *DHFR* | rs42290 | 5:80089022 | 0.55 | 4.03 | 8.08E-05 | 3.71E-02 |
| ENSG00000151470 | *C4orf33* | rs79088961 | 4:130136923 | 0.66 | 4.03 | 8.08E-05 | 3.71E-02 |
| ENSG00000251504 | *LINC01099* | rs4690597 | 4:178989256 | -0.48 | -4.03 | 8.09E-05 | 3.72E-02 |
| ENSG00000174080 | *CTSF* | rs560556 | 11:66338118 | 0.41 | 4.03 | 8.09E-05 | 3.72E-02 |
| ENSG00000174080 | *CTSF* | rs615205 | 11:66337987 | 0.41 | 4.03 | 8.09E-05 | 3.72E-02 |
| ENSG00000121898 | *CPXM2* | rs1996431 | 10:125645781 | -0.49 | -4.03 | 8.10E-05 | 3.72E-02 |
| ENSG00000253669 | *KB-1732A1.1* | rs34352733 | 8:103831508 | -0.43 | -4.03 | 8.11E-05 | 3.72E-02 |
| ENSG00000137033 | *IL33* | rs35042847 | 9:5294495 | -0.50 | -4.03 | 8.13E-05 | 3.73E-02 |
| ENSG00000257261 | *RP11-96H19.1* | rs146808132 | 12:46842122 | 1.03 | 4.03 | 8.14E-05 | 3.73E-02 |
| ENSG00000134278 | *SPIRE1* | rs12971256 | 18:12672008 | 0.36 | 4.03 | 8.14E-05 | 3.73E-02 |
| ENSG00000170369 | *CST2* | rs6106714 | 20:23768931 | -0.43 | -4.03 | 8.17E-05 | 3.75E-02 |
| ENSG00000173918 | *C1QTNF1* | rs56115702 | 17:77058111 | 0.76 | 4.03 | 8.19E-05 | 3.75E-02 |
| ENSG00000173918 | *C1QTNF1* | rs74780160 | 17:77057626 | 0.76 | 4.03 | 8.20E-05 | 3.76E-02 |
| ENSG00000174080 | *CTSF* | rs630021 | 11:66336989 | 0.41 | 4.03 | 8.21E-05 | 3.76E-02 |
| ENSG00000228716 | *DHFR* | rs6151716 | 5:80016301 | -0.56 | -4.03 | 8.22E-05 | 3.76E-02 |
| ENSG00000134278 | *SPIRE1* | rs1272000530 | 18:12672554 | 0.38 | 4.03 | 8.22E-05 | 3.76E-02 |
| ENSG00000173918 | *C1QTNF1* | rs57377371 | 17:77058436 | 0.76 | 4.03 | 8.23E-05 | 3.76E-02 |
| ENSG00000228716 | *DHFR* | rs33012 | 5:80087276 | 0.55 | 4.03 | 8.24E-05 | 3.77E-02 |
| ENSG00000169071 | *ROR2* | rs7862013 | 9:94687785 | 0.43 | 4.03 | 8.24E-05 | 3.77E-02 |
| ENSG00000142677 | *IL22RA1* | rs72882488 | 1:24819678 | -0.74 | -4.03 | 8.24E-05 | 3.77E-02 |
| ENSG00000112874 | *NUDT12* | rs251162 | 5:102867875 | -0.42 | -4.03 | 8.25E-05 | 3.77E-02 |
| ENSG00000174080 | *CTSF* | rs509556 | 11:66326362 | 0.41 | 4.02 | 8.30E-05 | 3.79E-02 |
| ENSG00000174080 | *CTSF* | rs677488 | 11:66324762 | 0.41 | 4.02 | 8.30E-05 | 3.79E-02 |
| ENSG00000174080 | *CTSF* | rs572846 | 11:66331458 | 0.41 | 4.02 | 8.30E-05 | 3.79E-02 |
| ENSG00000174080 | *CTSF* | rs2000939 | 11:66325830 | 0.41 | 4.02 | 8.30E-05 | 3.79E-02 |
| ENSG00000174080 | *CTSF* | rs678397 | 11:66324583 | 0.41 | 4.02 | 8.36E-05 | 3.81E-02 |
| ENSG00000173918 | *C1QTNF1* | rs62063767 | 17:77056604 | 0.76 | 4.02 | 8.36E-05 | 3.81E-02 |
| ENSG00000174080 | *CTSF* | rs545009 | 11:66333598 | 0.41 | 4.02 | 8.38E-05 | 3.82E-02 |
| ENSG00000142677 | *IL22RA1* | rs196400 | 1:24837599 | -0.41 | -4.02 | 8.41E-05 | 3.83E-02 |
| ENSG00000151470 | *C4orf33* | rs2218814 | 4:129885298 | 0.42 | 4.02 | 8.42E-05 | 3.83E-02 |
| ENSG00000228716 | *DHFR* | rs1650734 | 5:80004545 | -0.56 | -4.02 | 8.42E-05 | 3.83E-02 |
| ENSG00000228716 | *DHFR* | rs36204523 | 5:80082907 | 0.55 | 4.02 | 8.43E-05 | 3.84E-02 |
| ENSG00000134278 | *SPIRE1* | rs62096048 | 18:12733948 | 0.39 | 4.02 | 8.45E-05 | 3.84E-02 |
| ENSG00000151470 | *C4orf33* | rs6814109 | 4:129887584 | 0.42 | 4.02 | 8.48E-05 | 3.85E-02 |
| ENSG00000169071 | *ROR2* | rs10119342 | 9:94873767 | 0.42 | 4.02 | 8.49E-05 | 3.86E-02 |
| ENSG00000151470 | *C4orf33* | rs6820403 | 4:129888150 | 0.42 | 4.02 | 8.49E-05 | 3.86E-02 |
| ENSG00000151470 | *C4orf33* | rs6820637 | 4:129888428 | 0.42 | 4.02 | 8.50E-05 | 3.86E-02 |
| ENSG00000173918 | *C1QTNF1* | rs118101091 | 17:77055154 | 0.75 | 4.02 | 8.50E-05 | 3.86E-02 |
| ENSG00000151470 | *C4orf33* | rs4975270 | 4:129757215 | -0.41 | -4.02 | 8.50E-05 | 3.86E-02 |
| ENSG00000151470 | *C4orf33* | rs62317868 | 4:129725180 | -0.42 | -4.02 | 8.54E-05 | 3.88E-02 |
| ENSG00000053372 | *MRTO4* | rs55810339 | 1:18768291 | 0.60 | 4.02 | 8.57E-05 | 3.89E-02 |
| ENSG00000151470 | *C4orf33* | rs17013778 | 4:129759735 | -0.41 | -4.02 | 8.58E-05 | 3.89E-02 |
| ENSG00000112874 | *NUDT12* | rs159164 | 5:102866203 | -0.42 | -4.02 | 8.58E-05 | 3.89E-02 |
| ENSG00000170369 | *CST2* | rs6114290 | 20:23769817 | -0.47 | -4.02 | 8.58E-05 | 3.89E-02 |
| ENSG00000253669 | *KB-1732A1.1* | rs1002174896 | 8:103827352 | -0.41 | -4.02 | 8.59E-05 | 3.89E-02 |
| ENSG00000228716 | *DHFR* | rs33010 | 5:80083631 | 0.55 | 4.02 | 8.61E-05 | 3.90E-02 |
| ENSG00000173918 | *C1QTNF1* | rs62063766 | 17:77053912 | 0.75 | 4.01 | 8.63E-05 | 3.91E-02 |
| ENSG00000198542 | *ITGBL1* | rs56907128 | 13:101267201 | -5.71 | -4.01 | 8.65E-05 | 3.91E-02 |
| ENSG00000228716 | *DHFR* | rs1650736 | 5:80001990 | -0.56 | -4.01 | 8.68E-05 | 3.92E-02 |
| ENSG00000253669 | *KB-1732A1.1* | rs2679753 | 8:103867460 | -0.42 | -4.01 | 8.68E-05 | 3.92E-02 |
| ENSG00000253669 | *KB-1732A1.1* | rs35150478 | 8:103868292 | -0.42 | -4.01 | 8.68E-05 | 3.92E-02 |
| ENSG00000253669 | *KB-1732A1.1* | rs2513920 | 8:103830692 | -0.42 | -4.01 | 8.69E-05 | 3.93E-02 |
| ENSG00000253669 | *KB-1732A1.1* | rs1062048 | 8:103851052 | -0.42 | -4.01 | 8.69E-05 | 3.93E-02 |
| ENSG00000253669 | *KB-1732A1.1* | rs1019976 | 8:103864271 | -0.42 | -4.01 | 8.69E-05 | 3.93E-02 |
| ENSG00000253669 | *KB-1732A1.1* | rs2513917 | 8:103833327 | -0.42 | -4.01 | 8.69E-05 | 3.93E-02 |
| ENSG00000253669 | *KB-1732A1.1* | rs3018954 | 8:103833571 | -0.42 | -4.01 | 8.69E-05 | 3.93E-02 |
| ENSG00000253669 | *KB-1732A1.1* | rs2513915 | 8:103834540 | -0.42 | -4.01 | 8.69E-05 | 3.93E-02 |
| ENSG00000253669 | *KB-1732A1.1* | rs974758 | 8:103837491 | -0.42 | -4.01 | 8.69E-05 | 3.93E-02 |
| ENSG00000253669 | *KB-1732A1.1* | rs2513936 | 8:103844988 | -0.42 | -4.01 | 8.69E-05 | 3.93E-02 |
| ENSG00000253669 | *KB-1732A1.1* | rs2570941 | 8:103857481 | -0.42 | -4.01 | 8.69E-05 | 3.93E-02 |
| ENSG00000253669 | *KB-1732A1.1* | rs2679747 | 8:103858114 | -0.42 | -4.01 | 8.69E-05 | 3.93E-02 |
| ENSG00000253669 | *KB-1732A1.1* | rs2679750 | 8:103860392 | -0.42 | -4.01 | 8.69E-05 | 3.93E-02 |
| ENSG00000253669 | *KB-1732A1.1* | rs1897787 | 8:103862423 | -0.42 | -4.01 | 8.69E-05 | 3.93E-02 |
| ENSG00000253669 | *KB-1732A1.1* | rs2513876 | 8:103872521 | -0.42 | -4.01 | 8.69E-05 | 3.93E-02 |
| ENSG00000173918 | *C1QTNF1* | rs144821410 | 17:77053374 | 0.75 | 4.01 | 8.69E-05 | 3.93E-02 |
| ENSG00000253669 | *KB-1732A1.1* | rs2436851 | 8:103875613 | -0.42 | -4.01 | 8.69E-05 | 3.93E-02 |
| ENSG00000253669 | *KB-1732A1.1* | rs1991928 | 8:103873091 | -0.42 | -4.01 | 8.70E-05 | 3.93E-02 |
| ENSG00000228716 | *DHFR* | rs1650735 | 5:80002739 | -0.56 | -4.01 | 8.73E-05 | 3.94E-02 |
| ENSG00000148344 | *PTGES* | rs7041309 | 9:132043191 | 0.46 | 4.01 | 8.74E-05 | 3.94E-02 |
| ENSG00000180875 | *GREM2* | rs74149173 | 1:240754894 | 0.71 | 4.01 | 8.74E-05 | 3.95E-02 |
| ENSG00000228716 | *DHFR* | rs245382 | 5:80129958 | 0.54 | 4.01 | 8.79E-05 | 3.96E-02 |
| ENSG00000151470 | *C4orf33* | rs77331275 | 4:130137179 | 0.67 | 4.01 | 8.79E-05 | 3.96E-02 |
| ENSG00000142677 | *IL22RA1* | rs114258202 | 1:24814821 | -0.75 | -4.01 | 8.79E-05 | 3.96E-02 |
| ENSG00000151470 | *C4orf33* | rs6817901 | 4:129900134 | 0.42 | 4.01 | 8.80E-05 | 3.97E-02 |
| ENSG00000228716 | *DHFR* | rs39628 | 5:80075674 | 0.55 | 4.01 | 8.81E-05 | 3.97E-02 |
| ENSG00000134278 | *SPIRE1* | rs12959790 | 18:12613023 | 0.40 | 4.01 | 8.81E-05 | 3.97E-02 |
| ENSG00000151470 | *C4orf33* | rs4975292 | 4:129900477 | 0.42 | 4.01 | 8.81E-05 | 3.97E-02 |
| ENSG00000137033 | *IL33* | rs7022175 | 9:5286253 | -0.50 | -4.01 | 8.81E-05 | 3.97E-02 |
| ENSG00000151470 | *C4orf33* | rs77949011 | 4:130129256 | 0.69 | 4.01 | 8.82E-05 | 3.97E-02 |
| ENSG00000151470 | *C4orf33* | rs3099898 | 4:129902267 | -0.42 | -4.01 | 8.86E-05 | 3.99E-02 |
| ENSG00000136108 | *CKAP2* | rs9536265 | 13:53311557 | -0.40 | -4.01 | 8.86E-05 | 3.99E-02 |
| ENSG00000103187 | *COTL1* | rs4783067 | 16:84838975 | -0.43 | -4.01 | 8.87E-05 | 3.99E-02 |
| ENSG00000134278 | *SPIRE1* | rs4073592 | 18:12658045 | 0.35 | 4.01 | 8.89E-05 | 4.00E-02 |
| ENSG00000153823 | *PID1* | rs68150706 | 2:230424629 | -0.74 | -4.01 | 8.92E-05 | 4.01E-02 |
| ENSG00000228716 | *DHFR* | rs1011681711 | 5:79988600 | 0.63 | 4.01 | 8.92E-05 | 4.01E-02 |
| ENSG00000142677 | *IL22RA1* | rs9424323 | 1:24812795 | -0.74 | -4.01 | 8.92E-05 | 4.01E-02 |
| ENSG00000101463 | *SYNDIG1* | rs6083563 | 20:24569897 | -0.52 | -4.01 | 8.95E-05 | 4.02E-02 |
| ENSG00000112874 | *NUDT12* | rs251160 | 5:102869123 | -0.42 | -4.00 | 9.01E-05 | 4.04E-02 |
| ENSG00000148344 | *PTGES* | rs7041313 | 9:132043158 | 0.46 | 4.00 | 9.04E-05 | 4.05E-02 |
| ENSG00000127325 | *BEST3* | rs10506568 | 12:70043390 | 0.41 | 4.00 | 9.04E-05 | 4.05E-02 |
| ENSG00000151470 | *C4orf33* | rs2592953 | 4:129909196 | -0.42 | -4.00 | 9.05E-05 | 4.05E-02 |
| ENSG00000137033 | *IL33* | rs12344259 | 9:5280904 | -0.49 | -4.00 | 9.14E-05 | 4.09E-02 |
| ENSG00000070159 | *PTPN3* | rs75466648 | 9:113152877 | -0.76 | -4.00 | 9.18E-05 | 4.10E-02 |
| ENSG00000151470 | *C4orf33* | rs280602 | 4:129915017 | -0.42 | -4.00 | 9.20E-05 | 4.11E-02 |
| ENSG00000121898 | *CPXM2* | rs28568391 | 10:125628540 | -0.43 | -4.00 | 9.20E-05 | 4.11E-02 |
| ENSG00000124151 | *NCOA3* | rs77899196 | 20:45424110 | -0.60 | -4.00 | 9.22E-05 | 4.11E-02 |
| ENSG00000070159 | *PTPN3* | rs10817000 | 9:113143538 | -0.78 | -4.00 | 9.24E-05 | 4.12E-02 |
| ENSG00000228716 | *DHFR* | rs32994 | 5:80113468 | 0.51 | 4.00 | 9.24E-05 | 4.12E-02 |
| ENSG00000228716 | *DHFR* | rs245358 | 5:80143791 | 0.54 | 3.99 | 9.34E-05 | 4.16E-02 |
| ENSG00000124151 | *NCOA3* | rs8120796 | 20:45373055 | -0.70 | -3.99 | 9.34E-05 | 4.16E-02 |
| ENSG00000134278 | *SPIRE1* | rs112089575 | 18:12319995 | 0.73 | 3.99 | 9.39E-05 | 4.18E-02 |
| ENSG00000169071 | *ROR2* | rs185659380 | 9:95178183 | 0.98 | 3.99 | 9.40E-05 | 4.18E-02 |
| ENSG00000180011 | *ZADH2* | rs80349957 | 18:73751408 | -1.08 | -3.99 | 9.41E-05 | 4.18E-02 |
| ENSG00000180011 | *ZADH2* | rs78761481 | 18:73754292 | -1.08 | -3.99 | 9.41E-05 | 4.18E-02 |
| ENSG00000151470 | *C4orf33* | rs112499393 | 4:129732799 | -0.56 | -3.99 | 9.44E-05 | 4.19E-02 |
| ENSG00000196177 | *ACADSB* | rs118161107 | 10:125011707 | 1.31 | 3.99 | 9.53E-05 | 4.22E-02 |
| ENSG00000170369 | *CST2* | rs6049138 | 20:23783442 | -0.46 | -3.99 | 9.54E-05 | 4.23E-02 |
| ENSG00000115590 | *IL1R2* | rs7569218 | 2:102690578 | -0.40 | -3.99 | 9.62E-05 | 4.25E-02 |
| ENSG00000151470 | *C4orf33* | rs10518544 | 4:130029206 | 0.66 | 3.99 | 9.62E-05 | 4.26E-02 |
| ENSG00000153823 | *PID1* | rs56156768 | 2:230456232 | -0.97 | -3.99 | 9.63E-05 | 4.26E-02 |
| ENSG00000151470 | *C4orf33* | rs6854308 | 4:130139735 | 0.66 | 3.99 | 9.65E-05 | 4.27E-02 |
| ENSG00000151470 | *C4orf33* | rs9684807 | 4:130104062 | 0.67 | 3.99 | 9.65E-05 | 4.27E-02 |
| ENSG00000151470 | *C4orf33* | rs175964 | 4:129921238 | -0.41 | -3.99 | 9.67E-05 | 4.27E-02 |
| ENSG00000067798 | *NAV3* | rs74886250 | 12:77995533 | 0.46 | 3.99 | 9.67E-05 | 4.27E-02 |
| ENSG00000140876 | *NUDT7* | rs8063281 | 16:77274701 | 0.43 | 3.99 | 9.68E-05 | 4.27E-02 |
| ENSG00000171522 | *PTGER4* | rs10596585 | 5:40414968 | 0.44 | 3.98 | 9.70E-05 | 4.28E-02 |
| ENSG00000136108 | *CKAP2* | rs7983971 | 13:52790700 | -0.41 | -3.98 | 9.76E-05 | 4.30E-02 |
| ENSG00000134278 | *SPIRE1* | rs9957880 | 18:12676349 | 0.35 | 3.98 | 9.77E-05 | 4.31E-02 |
| ENSG00000137033 | *IL33* | rs13293588 | 9:5278596 | -0.49 | -3.98 | 9.77E-05 | 4.31E-02 |
| ENSG00000151470 | *C4orf33* | rs76983300 | 4:129776983 | -0.42 | -3.98 | 9.78E-05 | 4.31E-02 |
| ENSG00000113657 | *DPYSL3* | rs17464441 | 5:146438829 | 0.82 | 3.98 | 9.84E-05 | 4.33E-02 |
| ENSG00000151470 | *C4orf33* | rs62318666 | 4:129924438 | -0.41 | -3.98 | 9.85E-05 | 4.33E-02 |
| ENSG00000053108 | *FSTL4* | rs4958253 | 5:133179997 | -0.47 | -3.98 | 9.87E-05 | 4.34E-02 |
| ENSG00000169071 | *ROR2* | rs1005788884 | 9:94684400 | 0.43 | 3.98 | 9.88E-05 | 4.35E-02 |
| ENSG00000169071 | *ROR2* | rs7862779 | 9:94705255 | 0.40 | 3.98 | 9.90E-05 | 4.35E-02 |
| ENSG00000053108 | *FSTL4* | rs10073113 | 5:132473485 | 0.58 | 3.98 | 9.91E-05 | 4.35E-02 |
| ENSG00000169071 | *ROR2* | rs10125466 | 9:94704474 | 0.40 | 3.98 | 9.92E-05 | 4.36E-02 |
| ENSG00000169071 | *ROR2* | rs10125384 | 9:94704110 | 0.40 | 3.98 | 9.96E-05 | 4.37E-02 |
| ENSG00000127325 | *BEST3* | rs4367985 | 12:70048185 | 0.41 | 3.98 | 9.97E-05 | 4.38E-02 |
| ENSG00000117399 | *CDC20* | rs113997331 | 1:44570879 | -0.89 | -3.98 | 9.97E-05 | 4.38E-02 |
| ENSG00000169071 | *ROR2* | rs200083737 | 9:94684397 | 0.43 | 3.98 | 1.00E-04 | 4.39E-02 |
| ENSG00000253669 | *KB-1732A1.1* | rs2436849 | 8:103827548 | -0.42 | -3.98 | 1.00E-04 | 4.39E-02 |
| ENSG00000154864 | *PIEZO2* | rs575420 | 18:9848371 | 0.41 | 3.98 | 1.00E-04 | 4.40E-02 |
| ENSG00000134278 | *SPIRE1* | rs8083217 | 18:12666749 | 0.38 | 3.98 | 1.00E-04 | 4.40E-02 |
| ENSG00000151470 | *C4orf33* | rs398064029 | 4:129925309 | -0.41 | -3.98 | 1.00E-04 | 4.40E-02 |
| ENSG00000151470 | *C4orf33* | rs280592 | 4:129939725 | -0.41 | -3.97 | 1.01E-04 | 4.41E-02 |
| ENSG00000253669 | *KB-1732A1.1* | rs2916559 | 8:103827084 | -0.42 | -3.97 | 1.01E-04 | 4.41E-02 |
| ENSG00000117399 | *CDC20* | rs4660777 | 1:44572706 | -0.89 | -3.97 | 1.01E-04 | 4.41E-02 |
| ENSG00000134278 | *SPIRE1* | rs12964660 | 18:12556646 | 0.36 | 3.97 | 1.01E-04 | 4.42E-02 |
| ENSG00000151470 | *C4orf33* | rs2893267 | 4:129965914 | -0.41 | -3.97 | 1.01E-04 | 4.42E-02 |
| ENSG00000151470 | *C4orf33* | rs62316963 | 4:129963946 | -0.41 | -3.97 | 1.01E-04 | 4.42E-02 |
| ENSG00000151470 | *C4orf33* | rs1450727 | 4:129965026 | -0.41 | -3.97 | 1.01E-04 | 4.42E-02 |
| ENSG00000151470 | *C4orf33* | rs2592955 | 4:129960040 | -0.41 | -3.97 | 1.01E-04 | 4.43E-02 |
| ENSG00000151470 | *C4orf33* | rs2592956 | 4:129927852 | -0.41 | -3.97 | 1.01E-04 | 4.43E-02 |
| ENSG00000151470 | *C4orf33* | rs3119696 | 4:130017135 | -0.41 | -3.97 | 1.01E-04 | 4.44E-02 |
| ENSG00000151470 | *C4orf33* | rs789986 | 4:130015752 | -0.41 | -3.97 | 1.01E-04 | 4.44E-02 |
| ENSG00000151470 | *C4orf33* | rs4165 | 4:129942088 | -0.41 | -3.97 | 1.01E-04 | 4.44E-02 |
| ENSG00000151470 | *C4orf33* | rs1699390 | 4:130005022 | -0.41 | -3.97 | 1.02E-04 | 4.45E-02 |
| ENSG00000151470 | *C4orf33* | rs1757941 | 4:130000793 | -0.41 | -3.97 | 1.02E-04 | 4.45E-02 |
| ENSG00000127325 | *BEST3* | rs4761254 | 12:70051908 | 0.41 | 3.97 | 1.02E-04 | 4.45E-02 |
| ENSG00000112874 | *NUDT12* | rs7735978 | 5:103376332 | 0.48 | 3.97 | 1.02E-04 | 4.45E-02 |
| ENSG00000137033 | *IL33* | rs34315379 | 9:5279082 | -0.49 | -3.97 | 1.02E-04 | 4.46E-02 |
| ENSG00000151470 | *C4orf33* | rs542391 | 4:130045700 | -0.66 | -3.97 | 1.02E-04 | 4.46E-02 |
| ENSG00000151470 | *C4orf33* | rs1699396 | 4:130010603 | -0.41 | -3.97 | 1.02E-04 | 4.47E-02 |
| ENSG00000118922 | *KLF12* | rs17218228 | 13:74565579 | 1.53 | 3.97 | 1.02E-04 | 4.47E-02 |
| ENSG00000134121 | *CHL1* | rs4685395 | 3:88011 | 0.44 | 3.97 | 1.03E-04 | 4.48E-02 |
| ENSG00000137033 | *IL33* | rs35231446 | 9:5279019 | -0.49 | -3.97 | 1.03E-04 | 4.48E-02 |
| ENSG00000112874 | *NUDT12* | rs67686311 | 5:103375913 | 0.48 | 3.97 | 1.03E-04 | 4.49E-02 |
| ENSG00000134121 | *CHL1* | rs9845270 | 3:88643 | 0.44 | 3.97 | 1.03E-04 | 4.49E-02 |
| ENSG00000103187 | *COTL1* | rs12051056 | 16:84633436 | -0.51 | -3.97 | 1.03E-04 | 4.50E-02 |
| ENSG00000228716 | *DHFR* | rs11741562 | 5:79893662 | 0.43 | 3.97 | 1.03E-04 | 4.50E-02 |
| ENSG00000127325 | *BEST3* | rs7980708 | 12:70052654 | 0.41 | 3.97 | 1.03E-04 | 4.50E-02 |
| ENSG00000070159 | *PTPN3* | rs1028867 | 9:112528882 | 0.45 | 3.97 | 1.03E-04 | 4.50E-02 |
| ENSG00000160695 | *VPS11* | rs1237344592 | 11:119694508 | 0.73 | 3.97 | 1.04E-04 | 4.53E-02 |
| ENSG00000137033 | *IL33* | rs13294941 | 9:5278285 | -0.49 | -3.97 | 1.04E-04 | 4.54E-02 |
| ENSG00000228716 | *DHFR* | rs33009 | 5:80081885 | 0.54 | 3.96 | 1.05E-04 | 4.56E-02 |
| ENSG00000137033 | *IL33* | rs13294478 | 9:5278343 | -0.49 | -3.96 | 1.05E-04 | 4.56E-02 |
| ENSG00000134278 | *SPIRE1* | rs8094172 | 18:12603801 | 0.37 | 3.96 | 1.05E-04 | 4.56E-02 |
| ENSG00000136108 | *CKAP2* | rs7987349 | 13:52734858 | -0.46 | -3.96 | 1.05E-04 | 4.56E-02 |
| ENSG00000127325 | *BEST3* | rs2197359 | 12:70057616 | 0.41 | 3.96 | 1.05E-04 | 4.56E-02 |
| ENSG00000137033 | *IL33* | rs35756661 | 9:5278241 | -0.49 | -3.96 | 1.05E-04 | 4.57E-02 |
| ENSG00000180011 | *ZADH2* | rs11877228 | 18:73759541 | -1.08 | -3.96 | 1.06E-04 | 4.58E-02 |
| ENSG00000180011 | *ZADH2* | rs117304074 | 18:73759647 | -1.08 | -3.96 | 1.06E-04 | 4.58E-02 |
| ENSG00000184640 | *SEPT9* | rs4788925 | 17:74487582 | 0.79 | 3.96 | 1.06E-04 | 4.59E-02 |
| ENSG00000134278 | *SPIRE1* | rs2051262 | 18:12693655 | 0.35 | 3.96 | 1.06E-04 | 4.60E-02 |
| ENSG00000151470 | *C4orf33* | rs11345554 | 4:129871139 | 0.43 | 3.96 | 1.06E-04 | 4.60E-02 |
| ENSG00000070159 | *PTPN3* | rs4978921 | 9:113149405 | -0.75 | -3.96 | 1.06E-04 | 4.60E-02 |
| ENSG00000070159 | *PTPN3* | rs2255007 | 9:113149952 | -0.75 | -3.96 | 1.06E-04 | 4.61E-02 |
| ENSG00000070159 | *PTPN3* | rs1410046 | 9:113150547 | -0.75 | -3.96 | 1.06E-04 | 4.61E-02 |
| ENSG00000127325 | *BEST3* | rs73150500 | 12:70055082 | 0.41 | 3.96 | 1.06E-04 | 4.61E-02 |
| ENSG00000127325 | *BEST3* | rs17813527 | 12:70055592 | 0.41 | 3.96 | 1.07E-04 | 4.62E-02 |
| ENSG00000134278 | *SPIRE1* | rs11874852 | 18:12520278 | 0.38 | 3.96 | 1.07E-04 | 4.62E-02 |
| ENSG00000070159 | *PTPN3* | rs10980367 | 9:113156828 | -0.75 | -3.96 | 1.07E-04 | 4.64E-02 |
| ENSG00000134278 | *SPIRE1* | rs9959536 | 18:12694985 | 0.35 | 3.96 | 1.07E-04 | 4.64E-02 |
| ENSG00000134278 | *SPIRE1* | rs9960555 | 18:12689910 | 0.35 | 3.96 | 1.07E-04 | 4.64E-02 |
| ENSG00000184220 | *CMSS1* | rs1021102 | 3:99603780 | 0.38 | 3.96 | 1.08E-04 | 4.66E-02 |
| ENSG00000128683 | *GAD1* | rs55758238 | 2:170840619 | 1.30 | 3.96 | 1.08E-04 | 4.67E-02 |
| ENSG00000070159 | *PTPN3* | rs77491701 | 9:113160658 | -0.75 | -3.96 | 1.08E-04 | 4.67E-02 |
| ENSG00000117399 | *CDC20* | rs7529373 | 1:44578268 | -0.89 | -3.96 | 1.08E-04 | 4.67E-02 |
| ENSG00000134278 | *SPIRE1* | rs57010148 | 18:12515912 | 0.38 | 3.96 | 1.08E-04 | 4.67E-02 |
| ENSG00000141404 | *GNAL* | rs9960175 | 18:11726074 | 0.82 | 3.96 | 1.09E-04 | 4.68E-02 |
| ENSG00000112874 | *NUDT12* | rs60305106 | 5:103373138 | 0.48 | 3.96 | 1.09E-04 | 4.69E-02 |
| ENSG00000134278 | *SPIRE1* | rs35634465 | 18:12735020 | 0.39 | 3.95 | 1.09E-04 | 4.69E-02 |
| ENSG00000134278 | *SPIRE1* | rs9945801 | 18:12690101 | 0.35 | 3.95 | 1.09E-04 | 4.69E-02 |
| ENSG00000112874 | *NUDT12* | rs1420862 | 5:103372943 | 0.48 | 3.95 | 1.09E-04 | 4.70E-02 |
| ENSG00000140876 | *NUDT7* | rs28584133 | 16:77829879 | 0.60 | 3.95 | 1.09E-04 | 4.71E-02 |
| ENSG00000141404 | *GNAL* | rs9955178 | 18:11890221 | 0.54 | 3.95 | 1.09E-04 | 4.71E-02 |
| ENSG00000112874 | *NUDT12* | rs294044 | 5:102888453 | 0.42 | 3.95 | 1.09E-04 | 4.71E-02 |
| ENSG00000228716 | *DHFR* | rs1650723 | 5:79922030 | -0.55 | -3.95 | 1.10E-04 | 4.73E-02 |
| ENSG00000228716 | *DHFR* | rs1677697 | 5:79933933 | -0.55 | -3.95 | 1.10E-04 | 4.73E-02 |
| ENSG00000228716 | *DHFR* | rs1643656 | 5:79937709 | -0.55 | -3.95 | 1.10E-04 | 4.73E-02 |
| ENSG00000228716 | *DHFR* | rs1238951 | 5:79915790 | -0.55 | -3.95 | 1.10E-04 | 4.73E-02 |
| ENSG00000142677 | *IL22RA1* | rs196432 | 1:24861704 | -0.39 | -3.95 | 1.10E-04 | 4.73E-02 |
| ENSG00000070159 | *PTPN3* | rs146419073 | 9:113152423 | -0.75 | -3.95 | 1.10E-04 | 4.73E-02 |
| ENSG00000127325 | *BEST3* | rs775506 | 12:70059036 | -0.40 | -3.95 | 1.10E-04 | 4.73E-02 |
| ENSG00000121898 | *CPXM2* | rs28624661 | 10:125653184 | -0.41 | -3.95 | 1.10E-04 | 4.74E-02 |
| ENSG00000136108 | *CKAP2* | rs2760772 | 13:52780373 | -0.41 | -3.95 | 1.10E-04 | 4.74E-02 |
| ENSG00000228716 | *DHFR* | rs245397 | 5:80101773 | 0.51 | 3.95 | 1.10E-04 | 4.74E-02 |
| ENSG00000186187 | *ZNRF1* | rs6564178 | 16:74889289 | 0.44 | 3.95 | 1.10E-04 | 4.75E-02 |
| ENSG00000228716 | *DHFR* | rs1650750 | 5:79981222 | -0.47 | -3.95 | 1.11E-04 | 4.75E-02 |
| ENSG00000151470 | *C4orf33* | rs1709417 | 4:130031847 | -0.41 | -3.95 | 1.11E-04 | 4.77E-02 |
| ENSG00000151470 | *C4orf33* | rs498435 | 4:130032031 | -0.41 | -3.95 | 1.11E-04 | 4.77E-02 |
| ENSG00000112874 | *NUDT12* | rs143249117 | 5:103371530 | 0.48 | 3.95 | 1.11E-04 | 4.77E-02 |
| ENSG00000134278 | *SPIRE1* | rs55634668 | 18:12560012 | 0.38 | 3.95 | 1.11E-04 | 4.78E-02 |
| ENSG00000136111 | *TBC1D4* | rs16932 | 13:76625863 | 0.38 | 3.95 | 1.12E-04 | 4.79E-02 |
| ENSG00000149328 | *GLB1L2* | rs139324903 | 11:134195396 | 0.45 | 3.95 | 1.12E-04 | 4.79E-02 |
| ENSG00000053372 | *MRTO4* | rs2946524 | 1:18768277 | 0.42 | 3.95 | 1.12E-04 | 4.79E-02 |
| ENSG00000141404 | *GNAL* | rs67076514 | 18:11698718 | 0.84 | 3.95 | 1.12E-04 | 4.80E-02 |
| ENSG00000180801 | *ARSJ* | rs55637110 | 4:115244088 | -0.49 | -3.95 | 1.12E-04 | 4.80E-02 |
| ENSG00000141404 | *GNAL* | rs200022782 | 18:11698720 | 0.84 | 3.95 | 1.12E-04 | 4.80E-02 |
| ENSG00000141404 | *GNAL* | rs34760751 | 18:11698721 | 0.84 | 3.95 | 1.12E-04 | 4.80E-02 |
| ENSG00000069424 | *KCNAB2* | rs3810989 | 1:6163925 | -0.43 | -3.95 | 1.12E-04 | 4.81E-02 |
| ENSG00000142677 | *IL22RA1* | rs196422 | 1:24867734 | -0.39 | -3.95 | 1.13E-04 | 4.83E-02 |
| ENSG00000184220 | *CMSS1* | rs4928232 | 3:99694217 | -0.37 | -3.94 | 1.13E-04 | 4.85E-02 |
| ENSG00000134278 | *SPIRE1* | rs12457026 | 18:12551818 | 0.37 | 3.94 | 1.13E-04 | 4.85E-02 |
| ENSG00000053372 | *MRTO4* | rs12407770 | 1:18770855 | 0.59 | 3.94 | 1.14E-04 | 4.85E-02 |
| ENSG00000170369 | *CST2* | rs6083261 | 20:23770704 | -0.42 | -3.94 | 1.14E-04 | 4.86E-02 |
| ENSG00000253669 | *KB-1732A1.1* | rs2513912 | 8:103821488 | -0.42 | -3.94 | 1.14E-04 | 4.86E-02 |
| ENSG00000184220 | *CMSS1* | rs6765027 | 3:99687730 | -0.37 | -3.94 | 1.14E-04 | 4.87E-02 |
| ENSG00000136108 | *CKAP2* | rs112762703 | 13:52959835 | -0.41 | -3.94 | 1.14E-04 | 4.88E-02 |
| ENSG00000118922 | *KLF12* | rs149900916 | 13:74575336 | 1.50 | 3.94 | 1.15E-04 | 4.88E-02 |
| ENSG00000137033 | *IL33* | rs1396363230 | 9:5282012 | -0.50 | -3.94 | 1.15E-04 | 4.91E-02 |
| ENSG00000110448 | *CD5* | rs511580 | 11:60835596 | 0.38 | 3.94 | 1.16E-04 | 4.92E-02 |
| ENSG00000164111 | *ANXA5* | rs72678628 | 4:122644716 | -0.39 | -3.94 | 1.16E-04 | 4.92E-02 |
| ENSG00000184220 | *CMSS1* | rs6809988 | 3:99656615 | -0.37 | -3.94 | 1.16E-04 | 4.92E-02 |
| ENSG00000151470 | *C4orf33* | rs6817004 | 4:129727677 | -0.50 | -3.94 | 1.16E-04 | 4.94E-02 |
| ENSG00000228716 | *DHFR* | rs1677658 | 5:79950859 | -0.58 | -3.94 | 1.16E-04 | 4.95E-02 |
| ENSG00000134278 | *SPIRE1* | rs12458377 | 18:12426378 | 0.38 | 3.94 | 1.17E-04 | 4.96E-02 |
| ENSG00000136108 | *CKAP2* | rs7399852 | 13:52900538 | 0.41 | 3.94 | 1.17E-04 | 4.97E-02 |
| ENSG00000134278 | *SPIRE1* | rs12963179 | 18:12577354 | 0.36 | 3.94 | 1.17E-04 | 4.98E-02 |
| ENSG00000184220 | *CMSS1* | rs17174947 | 3:99598884 | 0.37 | 3.93 | 1.18E-04 | 4.98E-02 |
| ENSG00000184220 | *CMSS1* | rs793447 | 3:99598278 | 0.37 | 3.93 | 1.18E-04 | 4.99E-02 |
| ENSG00000184220 | *CMSS1* | rs9817005 | 3:99682692 | -0.37 | -3.93 | 1.18E-04 | 5.00E-02 |
| ENSG00000151470 | *C4orf33* | rs6834108 | 4:129725894 | -0.50 | -3.93 | 1.18E-04 | 5.00E-02 |
