# Supplementary material for: Islet Gene View—a tool to facilitate islet research
Source: Life Sci Alliance. 2022 Aug 10;5(12):e202201376. doi: 10.26508/lsa.202201376 (PMC9366203; doi:10.26508/lsa.202201376)
Supplement: Supplementary file 9 [file LSA-2022-01376_TableS9.docx]

Supplementary table 9. Look up of eQTLs from Supplementary table S12 for association with indices of insulin secretion (corrected insulin response-CIR and discposition index-DI in MAGIC consortium data (Prokopenko et al, 2014).

| **Association with Corrected insulin response (CIR)** | | | |  |  |  |  |  |
| --- | --- | --- | --- | --- | --- | --- | --- | --- |
| **snp** | **effect_allele** | **other_allele** | **maf** | **effect** | **stderr** | **pvalue** | **Position** | **eGene** |
| rs11794563 | T | C | .252 | 5.5e-02 | 2.5e-02 | .030421 | 9:132044938 | *PTGES* |
| rs17456931 | G | A | .292 | 6.0e-02 | 2.5e-02 | .016209 | 9:132046373 | *PTGES* |
| rs17456938 | G | A | .292 | 6.0e-02 | 2.5e-02 | .015496 | 9:132046491 | *PTGES* |
| rs196432 | A | G | .465 | -5.3e-02 | 2.0e-02 | .008739 | 1:24861704 | *IL22RA1* |
| rs3824537 | G | A | .283 | 5.6e-02 | 2.5e-02 | .026207 | 9:132045333 | *PTGES* |
| rs4319988 | C | G | .408 | 5.3e-02 | 2.1e-02 | .010954 | 20:23775453 | *CST2* |
| rs4438551 | A | G | .408 | 5.3e-02 | 2.1e-02 | .010965 | 20:23774050 | *CST2* |
| rs4549173 | C | A | .408 | 5.3e-02 | 2.1e-02 | .010938 | 20:23775488 | *CST2* |
| rs4690588 | C | T | .319 | 5.6e-02 | 2.6e-02 | .030719 | 4:178907903 | *LINC01099* |
| rs4836648 | A | G | .248 | 4.8e-02 | 2.4e-02 | .048449 | 9:132048664 | *PTGES* |
| rs6049120 | T | C | .408 | 5.4e-02 | 2.1e-02 | .009863 | 20:23765191 | *CST2* |
| rs6049126 | A | G | .376 | 5.3e-02 | 2.1e-02 | .01126 | 20:23772654 | *CST2* |
| rs6132672 | C | T | .417 | -5.3e-02 | 2.2e-02 | .015697 | 20:23809681 | *CST2* |
| rs6138082 | C | T | .408 | 5.4e-02 | 2.1e-02 | .009681 | 20:23763354 | *CST2* |
| rs7041309 | G | A | .292 | 5.8e-02 | 2.5e-02 | .018122 | 9:132043191 | *PTGES* |
| rs7041313 | T | C | .284 | 5.8e-02 | 2.5e-02 | .017945 | 9:132043158 | *PTGES* |
| rs7849964 | A | G | .308 | 5.2e-02 | 2.5e-02 | .035239 | 9:132050588 | *PTGES* |
| rs913768 | T | C | .257 | 5.8e-02 | 2.4e-02 | .018117 | 9:132044370 | *PTGES* |
| rs913769 | A | C | .283 | 5.6e-02 | 2.5e-02 | .026614 | 9:132044584 | *PTGES* |
|  |  |  |  |  |  |  |  |  |
| **Association with Disposition index (DI)** | | | |  |  |  |  |  |
| **snp** | **effect_allele** | **other_allele** | **maf** | **effect** | **stderr** | **pvalue** | **Position** | **eGene** |
| rs11794563 | T | C | .252 | 6.0e-02 | 2.5e-02 | .01727 | 9:132044938 | *PTGES* |
| rs17456931 | G | A | .292 | 6.9e-02 | 2.5e-02 | .005366 | 9:132046373 | *PTGES* |
| rs17456938 | G | A | .292 | 7.0e-02 | 2.5e-02 | .005103 | 9:132046491 | *PTGES* |
| rs17517009 | G | C | .267 | 5.0e-02 | 2.5e-02 | .048896 | 9:132038331 | *PTGES* |
| rs3824537 | G | A | .283 | 6.3e-02 | 2.5e-02 | .013427 | 9:132045333 | *PTGES* |
| rs3892337 | G | C | .457 | -5.3e-02 | 2.6e-02 | .04 | 13:53029485 | *CKAP2* |
| rs4319988 | C | G | .408 | 6.0e-02 | 2.1e-02 | .004394 | 20:23775453 | *CST2* |
| rs4438551 | A | G | .408 | 6.0e-02 | 2.1e-02 | .004396 | 20:23774050 | *CST2* |
| rs4549173 | C | A | .408 | 6.0e-02 | 2.1e-02 | .004383 | 20:23775488 | *CST2* |
| rs4704688 | C | T | .372 | 4.1e-02 | 2.0e-02 | .043051 | 5:80191705 | *DHFR* |
| rs4836648 | A | G | .248 | 6.5e-02 | 2.4e-02 | .007526 | 9:132048664 | *PTGES* |
| rs4836651 | C | G | .248 | 6.3e-02 | 2.4e-02 | .009626 | 9:132049329 | *PTGES* |
| rs4851527 | A | G | .456 | 4.0e-02 | 2.0e-02 | .049106 | 2:102622376 | *IL1R2* |
| rs6049120 | T | C | .408 | 6.0e-02 | 2.1e-02 | .004268 | 20:23765191 | *CST2* |
| rs6049126 | A | G | .376 | 6.0e-02 | 2.1e-02 | .004452 | 20:23772654 | *CST2* |
| rs6132672 | C | T | .417 | -6.2e-02 | 2.2e-02 | .004516 | 20:23809681 | *CST2* |
| rs6138082 | C | T | .408 | 6.0e-02 | 2.1e-02 | .00425 | 20:23763354 | *CST2* |
| rs7041309 | G | A | .292 | 6.6e-02 | 2.5e-02 | .00777 | 9:132043191 | *PTGES* |
| rs7041313 | T | C | .284 | 6.5e-02 | 2.5e-02 | .007808 | 9:132043158 | *PTGES* |
| rs7045226 | T | C | .305 | -4.3e-02 | 2.1e-02 | .043826 | 9:94621055 | *ROR2* |
| rs7849964 | A | G | .308 | 6.6e-02 | 2.5e-02 | .00764 | 9:132050588 | *PTGES* |
| rs913768 | T | C | .257 | 6.6e-02 | 2.5e-02 | .006934 | 9:132044370 | *PTGES* |
| rs913769 | A | C | .283 | 6.2e-02 | 2.5e-02 | .014335 | 9:132044584 | *PTGES* |
