# Supplementary material for: Islet Gene View—a tool to facilitate islet research
Source: Life Sci Alliance. 2022 Aug 10;5(12):e202201376. doi: 10.26508/lsa.202201376 (PMC9366203; doi:10.26508/lsa.202201376)
Supplement: Supplementary file 10 [file LSA-2022-01376_TableS10.docx]

Supplementary table 10. List of lead eQTLs per gene for the known T2D loci in islets (gene level quantifications). Genes showing differential expression are also shown in the adjacent columns

| Target gene(Ensembl ID) | SNP | Chr | Target gene | P(eQTL) | FDR(eQTL) | logFC(T2D) | P-value(T2D) | FDR(T2D) |
| --- | --- | --- | --- | --- | --- | --- | --- | --- |
| ENSG00000004534 | rs11715915 | 3 | RBM6 | 4.03E-02 | 7.72E-01 |  |  |  |
| ENSG00000005007 | rs3794991 | 19 | UPF1 | 3.84E-02 | 7.66E-01 |  |  |  |
| ENSG00000005007 | rs10401969 | 19 | UPF1 | 9.66E-03 | 5.69E-01 |  |  |  |
| ENSG00000007402 | rs11715915 | 3 | CACNA2D2 | 3.45E-02 | 7.53E-01 | -0.26 | 4.74E-02 | 2.70E-01 |
| ENSG00000010310 | rs8108269 | 19 | GIPR | 1.01E-03 | 2.18E-01 |  |  |  |
| ENSG00000011478 | rs10423928 | 19 | QPCTL | 6.81E-03 | 5.13E-01 |  |  |  |
| ENSG00000011478 | rs11672660 | 19 | QPCTL | 6.84E-03 | 5.13E-01 |  |  |  |
| ENSG00000011478 | rs2302593 | 19 | QPCTL | 9.55E-03 | 5.67E-01 |  |  |  |
| ENSG00000012061 | rs2302593 | 19 | ERCC1 | 2.61E-02 | 7.16E-01 | 0.19 | 2.26E-02 | 2.00E-01 |
| ENSG00000022840 | rs7957197 | 12 | RNF10 | 3.08E-02 | 7.39E-01 |  |  |  |
| ENSG00000022840 | rs12427353 | 12 | RNF10 | 3.20E-02 | 7.44E-01 |  |  |  |
| ENSG00000022840 | rs2650000 | 12 | RNF10 | 4.14E-02 | 7.75E-01 |  |  |  |
| ENSG00000049089 | rs2296172 | 1 | COL9A2 | 2.45E-03 | 3.43E-01 |  |  |  |
| ENSG00000053918 | rs8181588 | 11 | KCNQ1 | 4.06E-02 | 7.73E-01 |  |  |  |
| ENSG00000054219 | rs7593730 | 2 | LY75 | 3.60E-04 | 1.13E-01 |  |  |  |
| ENSG00000060339 | rs1802295 | 10 | CCAR1 | 2.73E-02 | 7.23E-01 |  |  |  |
| ENSG00000065060 | rs6912327 | 6 | UHRF1BP1 | 6.13E-04 | 1.62E-01 |  |  |  |
| ENSG00000065060 | rs4646949 | 6 | UHRF1BP1 | 4.52E-03 | 4.43E-01 |  |  |  |
| ENSG00000068028 | rs11715915 | 3 | RASSF1 | 4.84E-02 | 7.93E-01 |  |  |  |
| ENSG00000069399 | rs10423928 | 19 | BCL3 | 4.83E-02 | 7.93E-01 | 0.35 | 1.45E-02 | 1.68E-01 |
| ENSG00000069399 | rs11672660 | 19 | BCL3 | 4.82E-02 | 7.93E-01 | 0.35 | 1.45E-02 | 1.68E-01 |
| ENSG00000075975 | rs1797912 | 3 | MKRN2 | 3.96E-02 | 7.70E-01 |  |  |  |
| ENSG00000078668 | rs516946 | 8 | VDAC3 | 4.85E-02 | 7.93E-01 |  |  |  |
| ENSG00000078668 | rs515071 | 8 | VDAC3 | 4.86E-02 | 7.94E-01 |  |  |  |
| ENSG00000078902 | rs2334499 | 11 | TOLLIP | 2.87E-02 | 7.29E-01 |  |  |  |
| ENSG00000078902 | rs7107784 | 11 | TOLLIP | 4.92E-02 | 7.95E-01 |  |  |  |
| ENSG00000079112 | rs896854 | 8 | CDH17 | 2.03E-02 | 6.82E-01 |  |  |  |
| ENSG00000079112 | rs7845219 | 8 | CDH17 | 1.80E-02 | 6.66E-01 |  |  |  |
| ENSG00000079332 | rs1802295 | 10 | SAR1A | 3.88E-02 | 7.67E-01 |  |  |  |
| ENSG00000079689 | rs1800562 | 6 | SCGN | 3.23E-02 | 7.45E-01 | -0.36 | 1.00E-02 | 1.41E-01 |
| ENSG00000079691 | rs1800562 | 6 | LRRC16A | 4.75E-02 | 7.91E-01 |  |  |  |
| ENSG00000080371 | rs7961581 | 12 | RAB21 | 4.07E-02 | 7.73E-01 |  |  |  |
| ENSG00000082269 | rs1048886 | 6 | FAM135A | 3.22E-06 | 2.45E-03 |  |  |  |
| ENSG00000084693 | rs1260326 | 2 | AGBL5 | 5.62E-03 | 4.80E-01 | -0.17 | 5.60E-03 | 1.11E-01 |
| ENSG00000084693 | rs780094 | 2 | AGBL5 | 7.68E-03 | 5.32E-01 | -0.17 | 5.60E-03 | 1.11E-01 |
| ENSG00000084734 | rs3736594 | 2 | GCKR | 3.79E-02 | 7.65E-01 |  |  |  |
| ENSG00000087448 | rs10842994 | 12 | KLHL42 | 1.13E-02 | 5.95E-01 |  |  |  |
| ENSG00000089053 | rs2650000 | 12 | ANAPC5 | 8.55E-03 | 5.50E-01 |  |  |  |
| ENSG00000089597 | rs174550 | 11 | GANAB | 4.77E-02 | 7.92E-01 |  |  |  |
| ENSG00000089597 | rs174576 | 11 | GANAB | 4.66E-02 | 7.89E-01 |  |  |  |
| ENSG00000090520 | rs6769511 | 3 | DNAJB11 | 1.41E-02 | 6.29E-01 | 0.19 | 2.12E-02 | 1.94E-01 |
| ENSG00000090520 | rs1470579 | 3 | DNAJB11 | 1.41E-02 | 6.29E-01 | 0.19 | 2.12E-02 | 1.94E-01 |
| ENSG00000090520 | rs4402960 | 3 | DNAJB11 | 1.41E-02 | 6.29E-01 | 0.19 | 2.12E-02 | 1.94E-01 |
| ENSG00000090520 | rs7651090 | 3 | DNAJB11 | 1.41E-02 | 6.29E-01 | 0.19 | 2.12E-02 | 1.94E-01 |
| ENSG00000092621 | rs10923931 | 1 | PHGDH | 9.44E-04 | 2.09E-01 |  |  |  |
| ENSG00000095564 | rs1111875 | 10 | BTAF1 | 9.51E-03 | 5.67E-01 |  |  |  |
| ENSG00000095564 | rs5015480 | 10 | BTAF1 | 1.04E-02 | 5.80E-01 |  |  |  |
| ENSG00000099869 | rs7107784 | 11 | IGF2-AS | 4.96E-02 | 7.96E-01 | -0.54 | 2.19E-02 | 1.97E-01 |
| ENSG00000099995 | rs41278853 | 22 | SF3A1 | 3.54E-02 | 7.56E-01 |  |  |  |
| ENSG00000099999 | rs41278853 | 22 | RNF215 | 3.63E-02 | 7.59E-01 |  |  |  |
| ENSG00000104856 | rs2302593 | 19 | RELB | 4.71E-02 | 7.90E-01 | 0.34 | 2.76E-02 | 2.18E-01 |
| ENSG00000104859 | rs8108269 | 19 | CLASRP | 1.07E-03 | 2.25E-01 |  |  |  |
| ENSG00000104881 | rs2302593 | 19 | PPP1R13L | 3.13E-03 | 3.82E-01 | 0.36 | 1.86E-02 | 1.84E-01 |
| ENSG00000104881 | rs11672660 | 19 | PPP1R13L | 1.12E-03 | 2.30E-01 | 0.36 | 1.86E-02 | 1.84E-01 |
| ENSG00000104881 | rs10423928 | 19 | PPP1R13L | 1.09E-03 | 2.27E-01 | 0.36 | 1.86E-02 | 1.84E-01 |
| ENSG00000104892 | rs2302593 | 19 | KLC3 | 1.59E-02 | 6.47E-01 | 0.45 | 4.53E-02 | 2.66E-01 |
| ENSG00000104936 | rs8108269 | 19 | DMPK | 4.79E-04 | 1.38E-01 |  |  |  |
| ENSG00000104967 | rs11672660 | 19 | NOVA2 | 4.16E-02 | 7.76E-01 |  |  |  |
| ENSG00000104967 | rs10423928 | 19 | NOVA2 | 4.19E-02 | 7.77E-01 |  |  |  |
| ENSG00000106070 | rs6943153 | 7 | GRB10 | 6.18E-03 | 4.96E-01 |  |  |  |
| ENSG00000106078 | rs6943153 | 7 | COBL | 1.20E-02 | 6.04E-01 | 0.16 | 1.24E-02 | 1.55E-01 |
| ENSG00000106829 | rs17791513 | 9 | TLE4 | 4.18E-02 | 7.76E-01 |  |  |  |
| ENSG00000106829 | rs13292136 | 9 | TLE4 | 4.64E-02 | 7.88E-01 |  |  |  |
| ENSG00000107099 | rs3824420 | 9 | DOCK8 | 1.28E-02 | 6.14E-01 |  |  |  |
| ENSG00000107331 | rs60980157 | 9 | ABCA2 | 4.38E-02 | 7.82E-01 |  |  |  |
| ENSG00000107581 | rs10886471 | 10 | EIF3A | 3.75E-02 | 7.63E-01 |  |  |  |
| ENSG00000108061 | rs553668 | 10 | SHOC2 | 1.24E-02 | 6.09E-01 |  |  |  |
| ENSG00000110514 | rs10838687 | 11 | MADD | 4.20E-02 | 7.77E-01 |  |  |  |
| ENSG00000110628 | rs163184 | 11 | SLC22A18 | 1.77E-02 | 6.63E-01 |  |  |  |
| ENSG00000110700 | rs5215 | 11 | RPS13 | 4.86E-02 | 7.94E-01 |  |  |  |
| ENSG00000110700 | rs5219 | 11 | RPS13 | 4.90E-02 | 7.94E-01 |  |  |  |
| ENSG00000110944 | rs2657879 | 12 | IL23A | 1.58E-03 | 2.76E-01 |  |  |  |
| ENSG00000110958 | rs2657879 | 12 | PTGES3 | 1.13E-02 | 5.94E-01 |  |  |  |
| ENSG00000111328 | rs1727313 | 12 | CDK2AP1 | 4.26E-04 | 1.27E-01 |  |  |  |
| ENSG00000111328 | rs4275659 | 12 | CDK2AP1 | 1.45E-02 | 6.33E-01 |  |  |  |
| ENSG00000111775 | rs7957197 | 12 | COX6A1 | 2.65E-02 | 7.19E-01 |  |  |  |
| ENSG00000111775 | rs12427353 | 12 | COX6A1 | 2.28E-02 | 6.98E-01 |  |  |  |
| ENSG00000115207 | rs780094 | 2 | GTF3C2 | 3.64E-02 | 7.59E-01 |  |  |  |
| ENSG00000115226 | rs1371614 | 2 | FNDC4 | 4.81E-02 | 7.92E-01 |  |  |  |
| ENSG00000115241 | rs1371614 | 2 | PPM1G | 3.05E-02 | 7.37E-01 | 0.16 | 6.80E-03 | 1.20E-01 |
| ENSG00000117000 | rs2296172 | 1 | RLF | 4.82E-02 | 7.92E-01 |  |  |  |
| ENSG00000117600 | rs9727115 | 1 | LPPR4 | 4.48E-02 | 7.84E-01 |  |  |  |
| ENSG00000117877 | rs11672660 | 19 | CD3EAP | 7.09E-03 | 5.19E-01 |  |  |  |
| ENSG00000117877 | rs2302593 | 19 | CD3EAP | 3.31E-03 | 3.91E-01 |  |  |  |
| ENSG00000117877 | rs10423928 | 19 | CD3EAP | 6.95E-03 | 5.16E-01 |  |  |  |
| ENSG00000117906 | rs7178572 | 15 | RCN2 | 4.15E-02 | 7.76E-01 |  |  |  |
| ENSG00000118985 | rs1019503 | 5 | ELL2 | 1.35E-03 | 2.55E-01 |  |  |  |
| ENSG00000119326 | rs16913693 | 9 | CTNNAL1 | 9.65E-03 | 5.69E-01 | 0.22 | 4.00E-02 | 2.54E-01 |
| ENSG00000121671 | rs11605924 | 11 | CRY2 | 4.40E-02 | 7.82E-01 |  |  |  |
| ENSG00000121671 | rs11607883 | 11 | CRY2 | 4.81E-02 | 7.92E-01 |  |  |  |
| ENSG00000121680 | rs11605924 | 11 | PEX16 | 4.92E-02 | 7.95E-01 | 0.26 | 4.73E-02 | 2.70E-01 |
| ENSG00000122958 | rs1802295 | 10 | VPS26A | 6.43E-04 | 1.66E-01 |  |  |  |
| ENSG00000123091 | rs17106184 | 1 | RNF11 | 4.89E-02 | 7.94E-01 |  |  |  |
| ENSG00000123384 | rs2657879 | 12 | LRP1 | 5.01E-03 | 4.61E-01 |  |  |  |
| ENSG00000123444 | rs10501320 | 11 | KBTBD4 | 3.89E-02 | 7.68E-01 |  |  |  |
| ENSG00000123444 | rs10838687 | 11 | KBTBD4 | 1.02E-02 | 5.78E-01 |  |  |  |
| ENSG00000123444 | rs11039182 | 11 | KBTBD4 | 3.41E-02 | 7.51E-01 |  |  |  |
| ENSG00000123444 | rs7944584 | 11 | KBTBD4 | 3.43E-02 | 7.52E-01 |  |  |  |
| ENSG00000123965 | rs1167800 | 7 | PMS2P5 | 2.50E-02 | 7.11E-01 |  |  |  |
| ENSG00000124107 | rs6017317 | 20 | SLPI | 4.63E-02 | 7.88E-01 |  |  |  |
| ENSG00000124191 | rs4812829 | 20 | TOX2 | 4.14E-02 | 7.75E-01 |  |  |  |
| ENSG00000124191 | rs6017317 | 20 | TOX2 | 4.14E-02 | 7.75E-01 |  |  |  |
| ENSG00000124302 | rs731839 | 19 | CHST8 | 3.30E-02 | 7.47E-01 |  |  |  |
| ENSG00000124766 | rs7754840 | 6 | SOX4 | 2.57E-02 | 7.14E-01 |  |  |  |
| ENSG00000124766 | rs10946398 | 6 | SOX4 | 2.57E-02 | 7.14E-01 |  |  |  |
| ENSG00000124766 | rs4712524 | 6 | SOX4 | 2.57E-02 | 7.14E-01 |  |  |  |
| ENSG00000124766 | rs4712523 | 6 | SOX4 | 2.57E-02 | 7.14E-01 |  |  |  |
| ENSG00000124780 | rs1535500 | 6 | KCNK17 | 1.58E-10 | 3.13E-07 | 0.36 | 3.12E-02 | 2.30E-01 |
| ENSG00000124942 | rs174576 | 11 | AHNAK | 9.65E-03 | 5.69E-01 |  |  |  |
| ENSG00000124942 | rs174550 | 11 | AHNAK | 1.52E-02 | 6.40E-01 |  |  |  |
| ENSG00000125484 | rs651007 | 9 | GTF3C4 | 4.56E-02 | 7.87E-01 |  |  |  |
| ENSG00000125744 | rs8108269 | 19 | RTN2 | 4.47E-02 | 7.84E-01 |  |  |  |
| ENSG00000125753 | rs2302593 | 19 | VASP | 2.48E-02 | 7.10E-01 | 0.18 | 3.06E-02 | 2.27E-01 |
| ENSG00000127957 | rs1167800 | 7 | PMS2P3 | 1.09E-04 | 4.71E-02 |  |  |  |
| ENSG00000130203 | rs2302593 | 19 | APOE | 2.95E-02 | 7.33E-01 |  |  |  |
| ENSG00000130921 | rs1727313 | 12 | C12orf65 | 2.97E-02 | 7.34E-01 | -0.11 | 4.51E-02 | 2.65E-01 |
| ENSG00000133116 | rs576674 | 13 | KL | 2.06E-02 | 6.84E-01 |  |  |  |
| ENSG00000134121 | rs9841287 | 3 | CHL1 | 2.83E-02 | 7.27E-01 | -1.02 | 1.38E-05 | 4.25E-03 |
| ENSG00000134250 | rs10923931 | 1 | NOTCH2 | 3.63E-02 | 7.59E-01 |  |  |  |
| ENSG00000134569 | rs10838687 | 11 | LRP4 | 7.84E-04 | 1.88E-01 |  |  |  |
| ENSG00000134569 | rs10501320 | 11 | LRP4 | 1.18E-03 | 2.37E-01 |  |  |  |
| ENSG00000134569 | rs7944584 | 11 | LRP4 | 2.69E-03 | 3.58E-01 |  |  |  |
| ENSG00000134569 | rs11039182 | 11 | LRP4 | 2.75E-03 | 3.61E-01 |  |  |  |
| ENSG00000134574 | rs10838687 | 11 | DDB2 | 5.65E-03 | 4.81E-01 |  |  |  |
| ENSG00000134575 | rs10838687 | 11 | ACP2 | 1.84E-02 | 6.68E-01 | 0.27 | 2.00E-04 | 1.94E-02 |
| ENSG00000134824 | rs174550 | 11 | FADS2 | 2.89E-02 | 7.30E-01 |  |  |  |
| ENSG00000134824 | rs174576 | 11 | FADS2 | 3.06E-02 | 7.38E-01 |  |  |  |
| ENSG00000135114 | rs7305618 | 12 | OASL | 1.79E-03 | 2.95E-01 |  |  |  |
| ENSG00000135114 | rs7957197 | 12 | OASL | 4.89E-03 | 4.57E-01 |  |  |  |
| ENSG00000135114 | rs12427353 | 12 | OASL | 5.66E-03 | 4.82E-01 |  |  |  |
| ENSG00000135404 | rs2657879 | 12 | CD63 | 3.94E-02 | 7.69E-01 | 0.12 | 4.89E-02 | 2.74E-01 |
| ENSG00000135414 | rs2657879 | 12 | GDF11 | 2.06E-04 | 7.63E-02 |  |  |  |
| ENSG00000135424 | rs2657879 | 12 | ITGA7 | 1.82E-02 | 6.67E-01 |  |  |  |
| ENSG00000135917 | rs4675095 | 2 | SLC19A3 | 8.83E-03 | 5.55E-01 |  |  |  |
| ENSG00000136267 | rs2191349 | 7 | DGKB | 7.26E-04 | 1.79E-01 | 0.41 | 1.63E-02 | 1.75E-01 |
| ENSG00000136628 | rs2820436 | 1 | EPRS | 2.62E-02 | 7.17E-01 |  |  |  |
| ENSG00000137337 | rs3132524 | 6 | MDC1 | 1.49E-02 | 6.37E-01 |  |  |  |
| ENSG00000137337 | rs3130501 | 6 | MDC1 | 1.76E-02 | 6.62E-01 |  |  |  |
| ENSG00000138083 | rs895636 | 2 | SIX3 | 1.59E-08 | 2.05E-05 |  |  |  |
| ENSG00000138085 | rs1260326 | 2 | ATRAID | 2.39E-03 | 3.39E-01 |  |  |  |
| ENSG00000138085 | rs780094 | 2 | ATRAID | 7.55E-03 | 5.29E-01 |  |  |  |
| ENSG00000138085 | rs3736594 | 2 | ATRAID | 1.05E-02 | 5.82E-01 |  |  |  |
| ENSG00000138463 | rs11717195 | 3 | DIRC2 | 1.36E-04 | 5.57E-02 |  |  |  |
| ENSG00000138463 | rs11708067 | 3 | DIRC2 | 5.55E-04 | 1.52E-01 |  |  |  |
| ENSG00000138463 | rs2877716 | 3 | DIRC2 | 7.62E-04 | 1.84E-01 |  |  |  |
| ENSG00000138496 | rs11717195 | 3 | PARP9 | 1.62E-02 | 6.50E-01 |  |  |  |
| ENSG00000138496 | rs11708067 | 3 | PARP9 | 7.36E-03 | 5.25E-01 |  |  |  |
| ENSG00000138496 | rs2877716 | 3 | PARP9 | 1.66E-02 | 6.54E-01 |  |  |  |
| ENSG00000139233 | rs1531343 | 12 | LLPH | 3.64E-02 | 7.60E-01 |  |  |  |
| ENSG00000139233 | rs2261181 | 12 | LLPH | 3.39E-02 | 7.51E-01 |  |  |  |
| ENSG00000139531 | rs2657879 | 12 | SUOX | 1.24E-02 | 6.09E-01 |  |  |  |
| ENSG00000139579 | rs2657879 | 12 | NABP2 | 1.35E-02 | 6.22E-01 |  |  |  |
| ENSG00000139613 | rs2657879 | 12 | SMARCC2 | 2.72E-02 | 7.22E-01 |  |  |  |
| ENSG00000139641 | rs2657879 | 12 | ESYT1 | 4.11E-02 | 7.74E-01 |  |  |  |
| ENSG00000139725 | rs7305618 | 12 | RHOF | 4.91E-02 | 7.95E-01 |  |  |  |
| ENSG00000139746 | rs1359790 | 13 | RBM26 | 3.45E-02 | 7.53E-01 |  |  |  |
| ENSG00000140382 | rs7178572 | 15 | HMG20A | 6.19E-04 | 1.62E-01 |  |  |  |
| ENSG00000140382 | rs7177055 | 15 | HMG20A | 2.72E-03 | 3.59E-01 |  |  |  |
| ENSG00000140521 | rs2028299 | 15 | POLG | 3.46E-02 | 7.53E-01 |  |  |  |
| ENSG00000140575 | rs2028299 | 15 | IQGAP1 | 1.49E-02 | 6.37E-01 | -0.15 | 2.42E-02 | 2.05E-01 |
| ENSG00000141560 | rs1046896 | 17 | FN3KRP | 1.12E-10 | 2.27E-07 |  |  |  |
| ENSG00000141562 | rs1046896 | 17 | NARF | 3.90E-02 | 7.68E-01 |  |  |  |
| ENSG00000142252 | rs2302593 | 19 | GEMIN7 | 1.08E-03 | 2.26E-01 | 0.23 | 1.57E-02 | 1.72E-01 |
| ENSG00000142252 | rs10423928 | 19 | GEMIN7 | 2.34E-02 | 7.02E-01 | 0.23 | 1.57E-02 | 1.72E-01 |
| ENSG00000142252 | rs11672660 | 19 | GEMIN7 | 2.34E-02 | 7.02E-01 | 0.23 | 1.57E-02 | 1.72E-01 |
| ENSG00000142273 | rs11672660 | 19 | CBLC | 1.12E-02 | 5.93E-01 | 0.51 | 5.63E-04 | 3.53E-02 |
| ENSG00000142273 | rs10423928 | 19 | CBLC | 1.12E-02 | 5.93E-01 | 0.51 | 5.63E-04 | 3.53E-02 |
| ENSG00000142273 | rs2302593 | 19 | CBLC | 2.22E-02 | 6.94E-01 | 0.51 | 5.63E-04 | 3.53E-02 |
| ENSG00000143537 | rs67156297 | 1 | ADAM15 | 4.77E-02 | 7.92E-01 |  |  |  |
| ENSG00000145335 | rs3822072 | 4 | SNCA | 2.72E-02 | 7.22E-01 |  |  |  |
| ENSG00000145725 | rs36046591 | 5 | PPIP5K2 | 4.60E-02 | 7.87E-01 |  |  |  |
| ENSG00000145725 | rs35658696 | 5 | PPIP5K2 | 4.55E-02 | 7.86E-01 |  |  |  |
| ENSG00000145996 | rs9348440 | 6 | CDKAL1 | 2.58E-04 | 8.99E-02 |  |  |  |
| ENSG00000145996 | rs742642 | 6 | CDKAL1 | 5.02E-04 | 1.42E-01 |  |  |  |
| ENSG00000145996 | rs2206734 | 6 | CDKAL1 | 9.27E-04 | 2.07E-01 |  |  |  |
| ENSG00000145996 | rs7747752 | 6 | CDKAL1 | 1.08E-03 | 2.25E-01 |  |  |  |
| ENSG00000145996 | rs7754840 | 6 | CDKAL1 | 1.53E-02 | 6.41E-01 |  |  |  |
| ENSG00000145996 | rs6931514 | 6 | CDKAL1 | 4.41E-03 | 4.39E-01 |  |  |  |
| ENSG00000145996 | rs7766070 | 6 | CDKAL1 | 2.21E-02 | 6.94E-01 |  |  |  |
| ENSG00000145996 | rs4712523 | 6 | CDKAL1 | 1.52E-02 | 6.40E-01 |  |  |  |
| ENSG00000145996 | rs4712524 | 6 | CDKAL1 | 1.52E-02 | 6.40E-01 |  |  |  |
| ENSG00000145996 | rs10946398 | 6 | CDKAL1 | 1.53E-02 | 6.41E-01 |  |  |  |
| ENSG00000145996 | rs7756992 | 6 | CDKAL1 | 1.79E-02 | 6.64E-01 |  |  |  |
| ENSG00000145996 | rs9368222 | 6 | CDKAL1 | 1.85E-02 | 6.69E-01 |  |  |  |
| ENSG00000145996 | rs10440833 | 6 | CDKAL1 | 2.00E-02 | 6.80E-01 |  |  |  |
| ENSG00000146109 | rs1800562 | 6 | ABT1 | 4.02E-02 | 7.72E-01 |  |  |  |
| ENSG00000147883 | rs564398 | 9 | CDKN2B | 8.85E-03 | 5.55E-01 |  |  |  |
| ENSG00000147883 | rs944801 | 9 | CDKN2B | 1.22E-02 | 6.06E-01 |  |  |  |
| ENSG00000148248 | rs505922 | 9 | SURF4 | 4.31E-02 | 7.80E-01 |  |  |  |
| ENSG00000148426 | rs10906115 | 10 | PROSER2 | 4.00E-02 | 7.71E-01 |  |  |  |
| ENSG00000148985 | rs163182 | 11 | PGAP2 | 3.28E-02 | 7.47E-01 |  |  |  |
| ENSG00000148985 | rs163184 | 11 | PGAP2 | 3.28E-02 | 7.47E-01 |  |  |  |
| ENSG00000149177 | rs7944584 | 11 | PTPRJ | 1.98E-03 | 3.09E-01 |  |  |  |
| ENSG00000149177 | rs11039182 | 11 | PTPRJ | 1.98E-03 | 3.10E-01 |  |  |  |
| ENSG00000149177 | rs10501320 | 11 | PTPRJ | 3.70E-03 | 4.10E-01 |  |  |  |
| ENSG00000149489 | rs174576 | 11 | ROM1 | 4.05E-02 | 7.73E-01 | 0.30 | 7.43E-03 | 1.25E-01 |
| ENSG00000149541 | rs174576 | 11 | B3GAT3 | 2.72E-02 | 7.22E-01 |  |  |  |
| ENSG00000149541 | rs174550 | 11 | B3GAT3 | 4.82E-02 | 7.93E-01 |  |  |  |
| ENSG00000151090 | rs6780569 | 3 | THRB | 1.17E-02 | 6.00E-01 |  |  |  |
| ENSG00000151090 | rs7612463 | 3 | THRB | 1.71E-02 | 6.58E-01 |  |  |  |
| ENSG00000152253 | rs492594 | 2 | SPC25 | 1.67E-02 | 6.55E-01 |  |  |  |
| ENSG00000152969 | rs4689388 | 4 | JAKMIP1 | 3.18E-02 | 7.43E-01 |  |  |  |
| ENSG00000153253 | rs7607980 | 2 | SCN3A | 1.68E-02 | 6.55E-01 |  |  |  |
| ENSG00000153774 | rs7202877 | 16 | CFDP1 | 3.49E-03 | 4.00E-01 |  |  |  |
| ENSG00000154079 | rs1048886 | 6 | C6orf57 | 8.23E-04 | 1.93E-01 |  |  |  |
| ENSG00000154310 | rs11920090 | 3 | TNIK | 3.81E-02 | 7.65E-01 |  |  |  |
| ENSG00000154310 | rs1280 | 3 | TNIK | 3.81E-02 | 7.65E-01 |  |  |  |
| ENSG00000156162 | rs7845219 | 8 | DPY19L4 | 4.21E-02 | 7.77E-01 |  |  |  |
| ENSG00000156521 | rs1802295 | 10 | TYSND1 | 1.08E-02 | 5.87E-01 |  |  |  |
| ENSG00000157823 | rs2028299 | 15 | AP3S2 | 5.03E-22 | 6.88E-18 |  |  |  |
| ENSG00000160360 | rs3829109 | 9 | GPSM1 | 5.29E-05 | 2.65E-02 | 0.33 | 4.74E-02 | 2.70E-01 |
| ENSG00000160360 | rs11787792 | 9 | GPSM1 | 8.74E-05 | 3.95E-02 | 0.33 | 4.74E-02 | 2.70E-01 |
| ENSG00000160360 | rs60980157 | 9 | GPSM1 | 1.86E-04 | 7.05E-02 | 0.33 | 4.74E-02 | 2.70E-01 |
| ENSG00000160360 | rs1135314 | 9 | GPSM1 | 1.95E-03 | 3.07E-01 | 0.33 | 4.74E-02 | 2.70E-01 |
| ENSG00000160741 | rs67156297 | 1 | CRTC2 | 2.32E-02 | 7.01E-01 |  |  |  |
| ENSG00000162627 | rs9727115 | 1 | SNX7 | 4.17E-06 | 3.07E-03 |  |  |  |
| ENSG00000162924 | rs243088 | 2 | REL | 1.38E-02 | 6.25E-01 |  |  |  |
| ENSG00000162924 | rs243021 | 2 | REL | 6.12E-03 | 4.95E-01 |  |  |  |
| ENSG00000162927 | rs243088 | 2 | PUS10 | 4.29E-03 | 4.34E-01 |  |  |  |
| ENSG00000162927 | rs243021 | 2 | PUS10 | 7.59E-03 | 5.30E-01 |  |  |  |
| ENSG00000162928 | rs243021 | 2 | PEX13 | 4.54E-03 | 4.44E-01 |  |  |  |
| ENSG00000162928 | rs243088 | 2 | PEX13 | 4.91E-03 | 4.57E-01 |  |  |  |
| ENSG00000163635 | rs4607103 | 3 | ATXN7 | 2.74E-03 | 3.60E-01 |  |  |  |
| ENSG00000163798 | rs3736594 | 2 | SLC4A1AP | 7.15E-03 | 5.21E-01 |  |  |  |
| ENSG00000163840 | rs11708067 | 3 | DTX3L | 1.16E-03 | 2.35E-01 |  |  |  |
| ENSG00000163840 | rs11717195 | 3 | DTX3L | 2.17E-03 | 3.24E-01 |  |  |  |
| ENSG00000163840 | rs2877716 | 3 | DTX3L | 6.99E-03 | 5.17E-01 |  |  |  |
| ENSG00000163923 | rs16861329 | 3 | RPL39L | 4.64E-03 | 4.47E-01 |  |  |  |
| ENSG00000164062 | rs11715915 | 3 | APEH | 4.97E-02 | 7.96E-01 |  |  |  |
| ENSG00000164292 | rs13179048 | 5 | RHOBTB3 | 4.86E-02 | 7.94E-01 | -0.16 | 4.40E-02 | 2.63E-01 |
| ENSG00000164292 | rs4869272 | 5 | RHOBTB3 | 4.91E-02 | 7.94E-01 | -0.16 | 4.40E-02 | 2.63E-01 |
| ENSG00000164307 | rs1019503 | 5 | ERAP1 | 2.04E-03 | 3.14E-01 |  |  |  |
| ENSG00000164308 | rs1019503 | 5 | ERAP2 | 4.50E-40 | 1.75E-34 |  |  |  |
| ENSG00000165066 | rs516946 | 8 | NKX6-3 | 2.42E-04 | 8.57E-02 |  |  |  |
| ENSG00000165066 | rs515071 | 8 | NKX6-3 | 2.45E-04 | 8.66E-02 |  |  |  |
| ENSG00000165066 | rs12549902 | 8 | NKX6-3 | 3.16E-03 | 3.84E-01 |  |  |  |
| ENSG00000165609 | rs12779790 | 10 | NUDT5 | 2.57E-02 | 7.14E-01 |  |  |  |
| ENSG00000165689 | rs11787792 | 9 | SDCCAG3 | 3.80E-02 | 7.65E-01 | 0.24 | 8.92E-03 | 1.35E-01 |
| ENSG00000165689 | rs3829109 | 9 | SDCCAG3 | 2.66E-02 | 7.19E-01 | 0.24 | 8.92E-03 | 1.35E-01 |
| ENSG00000165689 | rs1135314 | 9 | SDCCAG3 | 4.45E-02 | 7.84E-01 | 0.24 | 8.92E-03 | 1.35E-01 |
| ENSG00000166012 | rs10830963 | 11 | TAF1D | 4.79E-02 | 7.92E-01 |  |  |  |
| ENSG00000166173 | rs1549318 | 15 | LARP6 | 1.82E-02 | 6.67E-01 |  |  |  |
| ENSG00000166816 | rs7202877 | 16 | LDHD | 7.77E-03 | 5.34E-01 |  |  |  |
| ENSG00000166886 | rs2657879 | 12 | NAB2 | 1.54E-03 | 2.74E-01 |  |  |  |
| ENSG00000167272 | rs2650000 | 12 | POP5 | 3.18E-03 | 3.85E-01 |  |  |  |
| ENSG00000167272 | rs12427353 | 12 | POP5 | 6.11E-03 | 4.95E-01 |  |  |  |
| ENSG00000167272 | rs7957197 | 12 | POP5 | 6.44E-03 | 5.03E-01 |  |  |  |
| ENSG00000167272 | rs7305618 | 12 | POP5 | 2.39E-02 | 7.05E-01 |  |  |  |
| ENSG00000167487 | rs10401969 | 19 | KLHL26 | 3.29E-02 | 7.47E-01 |  |  |  |
| ENSG00000167720 | rs4523957 | 17 | SRR | 4.29E-04 | 1.28E-01 |  |  |  |
| ENSG00000167720 | rs391300 | 17 | SRR | 1.88E-03 | 3.01E-01 |  |  |  |
| ENSG00000167720 | rs4790333 | 17 | SRR | 4.16E-02 | 7.76E-01 |  |  |  |
| ENSG00000167721 | rs4790333 | 17 | TSR1 | 3.09E-02 | 7.39E-01 | 0.15 | 2.46E-02 | 2.07E-01 |
| ENSG00000167986 | rs174576 | 11 | DDB1 | 1.69E-02 | 6.57E-01 |  |  |  |
| ENSG00000167986 | rs174550 | 11 | DDB1 | 2.41E-02 | 7.06E-01 |  |  |  |
| ENSG00000168216 | rs1048886 | 6 | LMBRD1 | 2.07E-02 | 6.85E-01 | -0.17 | 1.66E-03 | 6.52E-02 |
| ENSG00000168496 | rs174550 | 11 | FEN1 | 3.36E-02 | 7.50E-01 |  |  |  |
| ENSG00000168496 | rs174576 | 11 | FEN1 | 4.00E-02 | 7.71E-01 |  |  |  |
| ENSG00000168925 | rs7202877 | 16 | CTRB1 | 2.54E-02 | 7.13E-01 |  |  |  |
| ENSG00000168928 | rs7202877 | 16 | CTRB2 | 5.92E-03 | 4.89E-01 | 0.81 | 4.03E-02 | 2.55E-01 |
| ENSG00000168958 | rs4675095 | 2 | MFF | 2.79E-02 | 7.26E-01 |  |  |  |
| ENSG00000169020 | rs6815464 | 4 | ATP5I | 2.30E-02 | 6.99E-01 |  |  |  |
| ENSG00000169515 | rs2302593 | 19 | CCDC8 | 8.59E-03 | 5.51E-01 |  |  |  |
| ENSG00000170515 | rs2657879 | 12 | PA2G4 | 3.75E-02 | 7.63E-01 | 0.21 | 1.81E-02 | 1.83E-01 |
| ENSG00000170577 | rs895636 | 2 | SIX2 | 1.09E-03 | 2.27E-01 |  |  |  |
| ENSG00000170827 | rs505922 | 9 | CELP | 4.76E-03 | 4.52E-01 | 1.16 | 3.00E-04 | 2.54E-02 |
| ENSG00000170835 | rs505922 | 9 | CEL | 2.15E-03 | 3.22E-01 |  |  |  |
| ENSG00000171132 | rs895636 | 2 | PRKCE | 1.44E-02 | 6.32E-01 | 0.14 | 4.16E-02 | 2.58E-01 |
| ENSG00000171224 | rs7072268 | 10 | C10orf35 | 1.22E-02 | 6.07E-01 |  |  |  |
| ENSG00000173175 | rs11708067 | 3 | ADCY5 | 3.71E-04 | 1.16E-01 |  |  |  |
| ENSG00000173175 | rs11717195 | 3 | ADCY5 | 6.36E-04 | 1.65E-01 |  |  |  |
| ENSG00000173175 | rs2877716 | 3 | ADCY5 | 8.99E-04 | 2.03E-01 |  |  |  |
| ENSG00000173193 | rs11708067 | 3 | PARP14 | 2.17E-03 | 3.24E-01 | -0.22 | 2.18E-02 | 1.97E-01 |
| ENSG00000173193 | rs2877716 | 3 | PARP14 | 5.46E-03 | 4.76E-01 | -0.22 | 2.18E-02 | 1.97E-01 |
| ENSG00000173193 | rs11717195 | 3 | PARP14 | 4.89E-03 | 4.57E-01 | -0.22 | 2.18E-02 | 1.97E-01 |
| ENSG00000174238 | rs391300 | 17 | PITPNA | 2.36E-02 | 7.03E-01 |  |  |  |
| ENSG00000174238 | rs4523957 | 17 | PITPNA | 1.53E-02 | 6.41E-01 |  |  |  |
| ENSG00000174238 | rs4790333 | 17 | PITPNA | 1.54E-02 | 6.42E-01 |  |  |  |
| ENSG00000174672 | rs7107784 | 11 | BRSK2 | 3.77E-02 | 7.64E-01 |  |  |  |
| ENSG00000174721 | rs5015480 | 10 | FGFBP3 | 2.07E-02 | 6.85E-01 |  |  |  |
| ENSG00000174721 | rs1111875 | 10 | FGFBP3 | 2.44E-02 | 7.08E-01 |  |  |  |
| ENSG00000175216 | rs7944584 | 11 | CKAP5 | 5.72E-03 | 4.83E-01 |  |  |  |
| ENSG00000175216 | rs11039182 | 11 | CKAP5 | 5.76E-03 | 4.85E-01 |  |  |  |
| ENSG00000175216 | rs10501320 | 11 | CKAP5 | 7.77E-03 | 5.34E-01 |  |  |  |
| ENSG00000175220 | rs10501320 | 11 | ARHGAP1 | 3.60E-03 | 4.05E-01 |  |  |  |
| ENSG00000175220 | rs7944584 | 11 | ARHGAP1 | 7.88E-03 | 5.37E-01 |  |  |  |
| ENSG00000175220 | rs11039182 | 11 | ARHGAP1 | 7.99E-03 | 5.39E-01 |  |  |  |
| ENSG00000175220 | rs35233100 | 11 | ARHGAP1 | 1.31E-02 | 6.17E-01 |  |  |  |
| ENSG00000175220 | rs10838687 | 11 | ARHGAP1 | 4.32E-02 | 7.80E-01 |  |  |  |
| ENSG00000175224 | rs11039182 | 11 | ATG13 | 3.78E-02 | 7.64E-01 |  |  |  |
| ENSG00000175224 | rs7944584 | 11 | ATG13 | 3.83E-02 | 7.66E-01 |  |  |  |
| ENSG00000176182 | rs2302593 | 19 | MYPOP | 1.16E-02 | 5.99E-01 |  |  |  |
| ENSG00000177045 | rs10423928 | 19 | SIX5 | 3.09E-02 | 7.39E-01 |  |  |  |
| ENSG00000177045 | rs2302593 | 19 | SIX5 | 3.17E-02 | 7.43E-01 |  |  |  |
| ENSG00000177045 | rs11672660 | 19 | SIX5 | 3.13E-02 | 7.41E-01 |  |  |  |
| ENSG00000177239 | rs60980157 | 9 | MAN1B1 | 2.93E-02 | 7.32E-01 |  |  |  |
| ENSG00000178623 | rs3792267 | 2 | GPR35 | 2.87E-02 | 7.30E-01 |  |  |  |
| ENSG00000179562 | rs791595 | 7 | GCC1 | 1.68E-02 | 6.56E-01 |  |  |  |
| ENSG00000179912 | rs2657879 | 12 | R3HDM2 | 8.70E-03 | 5.53E-01 |  |  |  |
| ENSG00000180596 | rs1800562 | 6 | HIST1H2BC | 1.54E-02 | 6.42E-01 | 0.62 | 2.80E-02 | 2.19E-01 |
| ENSG00000181751 | rs36046591 | 5 | C5orf30 | 1.80E-02 | 6.65E-01 |  |  |  |
| ENSG00000181751 | rs35658696 | 5 | C5orf30 | 2.02E-02 | 6.81E-01 |  |  |  |
| ENSG00000182013 | rs10423928 | 19 | PNMAL1 | 9.03E-03 | 5.59E-01 | -0.25 | 1.97E-02 | 1.88E-01 |
| ENSG00000182013 | rs11672660 | 19 | PNMAL1 | 9.25E-03 | 5.63E-01 | -0.25 | 1.97E-02 | 1.88E-01 |
| ENSG00000182179 | rs11715915 | 3 | UBA7 | 8.88E-03 | 5.56E-01 |  |  |  |
| ENSG00000182196 | rs4275659 | 12 | ARL6IP4 | 3.46E-02 | 7.53E-01 |  |  |  |
| ENSG00000182196 | rs1727313 | 12 | ARL6IP4 | 2.61E-02 | 7.17E-01 |  |  |  |
| ENSG00000182247 | rs7612463 | 3 | UBE2E2 | 8.81E-03 | 5.55E-01 |  |  |  |
| ENSG00000182247 | rs1496653 | 3 | UBE2E2 | 1.10E-04 | 4.73E-02 |  |  |  |
| ENSG00000182247 | rs6780569 | 3 | UBE2E2 | 2.68E-03 | 3.57E-01 |  |  |  |
| ENSG00000183049 | rs11257655 | 10 | CAMK1D | 3.35E-12 | 8.97E-09 |  |  |  |
| ENSG00000183049 | rs12779790 | 10 | CAMK1D | 6.65E-09 | 9.51E-06 |  |  |  |
| ENSG00000183049 | rs10906115 | 10 | CAMK1D | 1.84E-07 | 1.91E-04 |  |  |  |
| ENSG00000183579 | rs41278853 | 22 | ZNRF3 | 2.38E-02 | 7.04E-01 |  |  |  |
| ENSG00000184209 | rs1727313 | 12 | SNRNP35 | 4.64E-02 | 7.88E-01 |  |  |  |
| ENSG00000184281 | rs2237892 | 11 | TSSC4 | 4.93E-02 | 7.95E-01 |  |  |  |
| ENSG00000184517 | rs7202877 | 16 | ZFP1 | 3.55E-03 | 4.03E-01 |  |  |  |
| ENSG00000185049 | rs6815464 | 4 | NELFA | 3.47E-02 | 7.53E-01 |  |  |  |
| ENSG00000185561 | rs391300 | 17 | TLCD2 | 3.28E-02 | 7.47E-01 |  |  |  |
| ENSG00000185561 | rs4790333 | 17 | TLCD2 | 3.50E-02 | 7.55E-01 |  |  |  |
| ENSG00000185561 | rs4523957 | 17 | TLCD2 | 2.61E-02 | 7.17E-01 |  |  |  |
| ENSG00000185633 | rs2657879 | 12 | NDUFA4L2 | 1.63E-02 | 6.51E-01 |  |  |  |
| ENSG00000186567 | rs10423928 | 19 | CEACAM19 | 4.28E-02 | 7.79E-01 |  |  |  |
| ENSG00000186567 | rs11672660 | 19 | CEACAM19 | 4.29E-02 | 7.79E-01 |  |  |  |
| ENSG00000187045 | rs855791 | 22 | TMPRSS6 | 2.08E-02 | 6.85E-01 |  |  |  |
| ENSG00000187609 | rs60980157 | 9 | EXD3 | 1.66E-02 | 6.53E-01 |  |  |  |
| ENSG00000196260 | rs3132524 | 6 | SFTA2 | 4.64E-02 | 7.88E-01 |  |  |  |
| ENSG00000196531 | rs2657879 | 12 | NACA | 1.68E-02 | 6.55E-01 |  |  |  |
| ENSG00000196981 | rs11717195 | 3 | WDR5B | 2.50E-02 | 7.11E-01 |  |  |  |
| ENSG00000196981 | rs2877716 | 3 | WDR5B | 2.20E-02 | 6.93E-01 |  |  |  |
| ENSG00000196981 | rs11708067 | 3 | WDR5B | 2.63E-02 | 7.18E-01 |  |  |  |
| ENSG00000198535 | rs1436958 | 15 | C2CD4A | 3.93E-02 | 7.69E-01 |  |  |  |
| ENSG00000204344 | rs2244020 | 6 | STK19 | 1.54E-02 | 6.42E-01 |  |  |  |
| ENSG00000204387 | rs2244020 | 6 | C6orf48 | 5.86E-03 | 4.87E-01 |  |  |  |
| ENSG00000204396 | rs2244020 | 6 | VWA7 | 3.20E-02 | 7.44E-01 |  |  |  |
| ENSG00000204520 | rs2244020 | 6 | MICA | 1.18E-03 | 2.37E-01 | 0.36 | 3.99E-02 | 2.54E-01 |
| ENSG00000204525 | rs2244020 | 6 | HLA-C | 9.91E-04 | 2.15E-01 |  |  |  |
| ENSG00000204525 | rs3130501 | 6 | HLA-C | 4.25E-03 | 4.33E-01 |  |  |  |
| ENSG00000204525 | rs3132524 | 6 | HLA-C | 5.54E-03 | 4.78E-01 |  |  |  |
| ENSG00000204536 | rs3132524 | 6 | CCHCR1 | 1.49E-03 | 2.69E-01 |  |  |  |
| ENSG00000204536 | rs3130501 | 6 | CCHCR1 | 1.51E-03 | 2.71E-01 |  |  |  |
| ENSG00000205502 | rs7163757 | 15 | C2CD4B | 2.23E-03 | 3.28E-01 | 0.39 | 1.48E-02 | 1.68E-01 |
| ENSG00000205502 | rs11071657 | 15 | C2CD4B | 1.14E-02 | 5.96E-01 | 0.39 | 1.48E-02 | 1.68E-01 |
| ENSG00000205502 | rs12440695 | 15 | C2CD4B | 9.12E-03 | 5.60E-01 | 0.39 | 1.48E-02 | 1.68E-01 |
| ENSG00000205502 | rs4502156 | 15 | C2CD4B | 6.09E-03 | 4.94E-01 | 0.39 | 1.48E-02 | 1.68E-01 |
| ENSG00000205502 | rs7172432 | 15 | C2CD4B | 2.23E-03 | 3.28E-01 | 0.39 | 1.48E-02 | 1.68E-01 |
| ENSG00000205531 | rs163182 | 11 | NAP1L4 | 9.06E-03 | 5.59E-01 |  |  |  |
| ENSG00000205531 | rs163184 | 11 | NAP1L4 | 1.83E-02 | 6.68E-01 |  |  |  |
| ENSG00000205583 | rs1167800 | 7 | STAG3L1 | 1.42E-06 | 1.18E-03 |  |  |  |
| ENSG00000213213 | rs60980157 | 9 | CCDC183 | 7.93E-03 | 5.38E-01 |  |  |  |
| ENSG00000213213 | rs11787792 | 9 | CCDC183 | 3.57E-02 | 7.57E-01 |  |  |  |
| ENSG00000213213 | rs3829109 | 9 | CCDC183 | 1.92E-02 | 6.74E-01 |  |  |  |
| ENSG00000213221 | rs3829109 | 9 | DNLZ | 5.04E-07 | 4.76E-04 |  |  |  |
| ENSG00000213221 | rs11787792 | 9 | DNLZ | 7.07E-06 | 4.87E-03 |  |  |  |
| ENSG00000213221 | rs60980157 | 9 | DNLZ | 1.41E-05 | 8.78E-03 |  |  |  |
| ENSG00000213221 | rs1135314 | 9 | DNLZ | 4.09E-03 | 4.26E-01 |  |  |  |
| ENSG00000214331 | rs7202877 | 16 | RP11-252A24.2 | 2.20E-02 | 6.93E-01 |  |  |  |
| ENSG00000214491 | rs41278853 | 22 | SEC14L6 | 6.01E-03 | 4.92E-01 | 0.22 | 4.83E-02 | 2.72E-01 |
| ENSG00000214530 | rs11603334 | 11 | STARD10 | 9.07E-10 | 1.58E-06 |  |  |  |
| ENSG00000214530 | rs1552224 | 11 | STARD10 | 9.07E-10 | 1.58E-06 |  |  |  |
| ENSG00000214753 | rs174576 | 11 | HNRNPUL2 | 4.60E-02 | 7.87E-01 |  |  |  |
| ENSG00000215375 | rs6815464 | 4 | MYL5 | 9.77E-03 | 5.71E-01 |  |  |  |
| ENSG00000221983 | rs10401969 | 19 | UBA52 | 1.43E-02 | 6.31E-01 |  |  |  |
| ENSG00000221983 | rs3794991 | 19 | UBA52 | 3.19E-03 | 3.85E-01 |  |  |  |
| ENSG00000223658 | rs10200833 | 2 | AC011242.6 | 4.63E-02 | 7.88E-01 |  |  |  |
| ENSG00000229117 | rs2657879 | 12 | RPL41 | 4.87E-04 | 1.39E-01 |  |  |  |
| ENSG00000232112 | rs11715915 | 3 | TMA7 | 7.83E-03 | 5.35E-01 |  |  |  |
| ENSG00000232645 | rs6048205 | 20 | LINC01431 | 4.65E-02 | 7.89E-01 |  |  |  |
| ENSG00000232645 | rs6113722 | 20 | LINC01431 | 4.15E-02 | 7.76E-01 |  |  |  |
| ENSG00000233276 | rs11715915 | 3 | GPX1 | 4.43E-02 | 7.83E-01 |  |  |  |
| ENSG00000234072 | rs3736594 | 2 | AC074117.10 | 2.62E-02 | 7.17E-01 |  |  |  |
| ENSG00000234127 | rs3132524 | 6 | TRIM26 | 1.19E-02 | 6.03E-01 |  |  |  |
| ENSG00000234127 | rs3130501 | 6 | TRIM26 | 1.47E-02 | 6.35E-01 |  |  |  |
| ENSG00000235423 | rs1727313 | 12 | RP11-282O18.3 | 1.51E-02 | 6.39E-01 | -0.30 | 4.09E-02 | 2.56E-01 |
| ENSG00000235423 | rs4275659 | 12 | RP11-282O18.3 | 1.76E-02 | 6.62E-01 | -0.30 | 4.09E-02 | 2.56E-01 |
| ENSG00000236502 | rs895636 | 2 | SIX3-AS1 | 2.30E-08 | 2.87E-05 |  |  |  |
| ENSG00000240338 | rs7202877 | 16 | RP11-331F4.4 | 1.69E-05 | 1.02E-02 | 0.97 | 4.73E-03 | 1.02E-01 |
| ENSG00000241370 | rs3132524 | 6 | RPP21 | 2.42E-02 | 7.07E-01 |  |  |  |
| ENSG00000241370 | rs3130501 | 6 | RPP21 | 3.28E-02 | 7.47E-01 |  |  |  |
| ENSG00000241549 | rs1800562 | 6 | GUSBP2 | 1.06E-02 | 5.85E-01 |  |  |  |
| ENSG00000243147 | rs3736594 | 2 | MRPL33 | 1.54E-02 | 6.42E-01 |  |  |  |
| ENSG00000250021 | rs2028299 | 15 | C15orf38-AP3S2 | 1.59E-23 | 2.58E-19 | -0.67 | 3.76E-02 | 2.47E-01 |
| ENSG00000250742 | rs1153188 | 12 | RP11-834C11.4 | 4.34E-02 | 7.81E-01 |  |  |  |
| ENSG00000258399 | rs3783347 | 14 | MEG8 | 3.66E-02 | 7.60E-01 |  |  |  |
| ENSG00000259974 | rs6113722 | 20 | LINC00261 | 1.36E-05 | 8.54E-03 | -0.21 | 4.58E-02 | 2.66E-01 |
| ENSG00000259974 | rs6048205 | 20 | LINC00261 | 1.23E-05 | 7.82E-03 | -0.21 | 4.58E-02 | 2.66E-01 |
| ENSG00000260563 | rs1046896 | 17 | RP13-516M14.1 | 3.53E-02 | 7.56E-01 |  |  |  |
| ENSG00000269176 | rs174576 | 11 | RP11-727F15.12 | 1.26E-03 | 2.45E-01 |  |  |  |
| ENSG00000269176 | rs174550 | 11 | RP11-727F15.12 | 1.59E-03 | 2.77E-01 |  |  |  |
| ENSG00000273033 | rs11717195 | 3 | RP11-67L2.2 | 3.42E-02 | 7.52E-01 |  |  |  |
